# Supplementary material for: Complete sequencing of ape genomes
Source: Nature. 2025 Apr 9;641(8062):401–18. doi: 10.1038/s41586-025-08816-3 (PMC12058530; doi:10.1038/s41586-025-08816-3)
Supplement: Supplementary file 1 — Supplementary Note and Methods 1–21, Supplementary Figs. 1–79 and Supplementary References. [file 41586_2025_8816_MOESM1_ESM.pdf]

nature portfolio

https://doi.org/10.1038/s41586-025-08816-3

Supplementary information

Complete sequencing of ape genomes

In the format provided by the authors and unedited

Complete sequencing of ape genomes

Supplementary Notes

Table of Contents

I. Sequencing and samples 2
II. Genome assemblies 8
III. Genome alignment and sequence divergence 19
IV. Sumatran vs. Bornean orangutan divergence 38
V. Structurally divergent regions 40
VI. Incomplete lineage sorting (ILS) and speciation times 42
VII. Selection analyses within NHP lineages 52
VIII. Gene annotation 65
IX. Repeat annotation 75
X. Immunoglobulin annotation and analysis 79
XI. MHC I and MHC II analyses 85
XII. Evolutionary rearrangements and inversion characterization 101
XIII. AQER 105
XIV. TOGA analysis 117
XV. Non-B DNA annotations 120
XVI. Methylation 121
XVII. Replication timing 127
XVIII. Acrocentric region analysis 134
XIX. Centromere analyses 147
XX. Subterminal satellite 151
XXI. Segmental duplications 153
REFERENCES 161

I. Sequencing and samples

Contributing authors:

Katherine M. Munson, Kendra Hoekzema, Richard E. Green, Samuel Sacco, Gage H. Garcia, Gerard G. Bouffard, Shelise Y. Brooks, Juyun Crawford, David Gilbert, Takayo Sasaki, Lucia Carbone, Laura Carrel, Marlys Houck, Oliver A. Ryder, Cynthia Steiner, Alexandra P. Lewis, Barbara McGrath, Joana L. Rocha, Kateryna D. Makova

Methods

Sample selection and sequencing data were mostly reported in Makova et al, 2024[1]; briefly, including the whole-genome sequencing data (PacBio HiFi [high-fidelity] and ONT [Oxford Nanopore Technologies] long-read/Illumina short-read) derived from fibroblast cell lines of bonobo, gorilla, Bornean/Sumatran orangutans, and that of chimpanzee and siamang derived from lymphoblast cell lines, used directly in genome assembly. Moreover, with the parent–child trio available samples, including bonobo and gorilla, parental Illumina data were used for haplotype phasing. For the remaining samples, including chimpanzee, two orangutans and siamang, Hi-C data were used to perform haplotype phasing. Iso-Seq data from testes were used for gene annotation. On top of these data, additional data were also generated to assist in assembly and annotation of the autosomes, including 1) additional Iso-Seq/short-read RNA-seq data to assist annotation and 2) additional HiFi (bonobo lymphoblast cell line) and ONT (gorilla fibroblast, and bonobo lymphoblast cell lines) to improve genome assembly (**Table 1, Supplementary Table I.1**).

PacBio Iso-Seq and RNA-seq at Penn State University: All male-derived cell lines were cultured, pelleted, and stored as described in Makova 2024[1]. Total RNA was isolated from approximately 5 million cells using the RNeasy Mini Kit (Qiagen) with on-column DNase digestion according to the manufacturer's protocol. RNA was eluted in nuclease-free water, snap-frozen in liquid nitrogen, and stored at -80°C while awaiting downstream analyses.

Uniquely indexed, short- and long-read SMRTbell templates were prepared for Iso-Seq (PacBio) transcriptome sequencing by the Huck Genomics Core Facility with the goal of achieving 3-4 million reads per SMRT cell. Size selection (~2 kbp and >3 kbp) was achieved by altering the volume of ProNex Beads after cDNA amplification. Samples were pooled in an equimolar pair-wise fashion and loaded onto SMRT cells for sequencing on the core facility's Sequel IIe System. Samples were pooled such that highly related species were not sequenced on the same flow cells.

For RNA-seq, uniquely indexed Illumina transcript libraries were prepared from cell line total RNA using the Illumina Stranded mRNA Prep Kit. An equimolar pool of all of the libraries was sequenced with a NextSeq 2000 P3; to achieve ~150 million pairs of 150 bp reads per sample (150 x 150 paired-end).

PacBio Iso-Seq and Kinnex Sequencing at the University of Washington (UW): Aliquots of the lymphoblast and fibroblast cell line RNA extracted by the Makova lab were shipped to the UW and prepared for PacBio Iso-Seq full-length transcriptome sequencing using the Iso-Seq Express Kit according to the manufacturer's protocol (PacBio, Preparing Iso-Seq Libraries using SMRTbell prep kit 3.0) with sample barcodes added during the cDNA PCR amplification step as in the protocol, and SMRTbell barcoded adapter plate 3.0 (PacBio P/N 102-009-200) used during library preparation with the SMRTbell prep kit 3.0 (PacBio P/N 102-182-700). Libraries were pooled in an equimolar fashion before sequencing on one SMRT Cell 8M on a PacBio Sequel II instrument using chemistry P3.1/C2.0 (PacBio P/Ns 102-333-400, 101-849-000).

Next, 5 ng of each cDNA were re-amplified for five cycles using barcoded primers compatible with a beta version of the Kinnex full-length RNA kit (PacBio P/N 103-072-000), then pooled in an equimolar fashion before Kinnex PCR and the remainder of the Kinnex full-length RNA protocol. The final library was sequenced on two SMRT Cell 25Ms on a PacBio Revio instrument using chemistry v1 and SMRT Link v12 (PacBio P/N 102-817-900).

After sequencing, data were analyzed using the Read Segmentation (Kinnex only) and Iso-Seq Analysis pipelines (Iso-Seq and Kinnex) in SMRT Link v12 to generate demultiplexed flnc.bam files.

PacBio HiFi at University of California, Berkeley: Lymphoblastoid cell lines available at the Coriell Institute for Medical Research were used for HiFi sequencing and expanded to a total culture size of 3×10e6 cells. The cell line expansions were derived from the original expansion culture to reduce the number of passages and minimize culturing time. Cells were washed in PBS and flash-frozen as dry cell pellets of 10e6 cells per vial. High-molecular weight (HMW) DNA was extracted on December 1-3, 2021, using the Circulomics CBB kit (102-573-600) from a frozen cell pellet (10e6 cells). DNA quantity, purity, and integrity were checked at different steps and at the end of the extraction protocol. DNA quantity was checked on a Qubit Fluorometer I with a dsDNA HS Assay kit (ThermoFisher) and sizes examined on a FEMTO pulse (Agilent Technologies) using a Genomic DNA 165 kb kit. Purity ratios were assessed with NanoDrop. A total of 54.8 micrograms of DNA (274 ng/uL in 200 uL volume, over 50 kbp length, and purity ratio 260/280: 1.82, 260/230:2.0) was used as input for library preparation.

A starting amount of 4-5 ug HMW gDNA was sheared to a target size of 20-30 kbp using a Megaruptor 3 instrument (Diagenode). The sheared DNA underwent size selection using a Pippin HT instrument (Sage Science) to target a size range of 15-22 kbp. Following size selection, the DNA was used for CCS (circular consensus sequencing) library preparation using the SMRTbell Express Template Prep Kit 2.0 and Enzyme Cleanup Kit 1.0 (PacBio). Each library was barcoded using PacBio Barcoded Overhang Adapters. Post-library preparation, the concentration of the DNA stock was measured using the DNA-HS Qubit assay, and the DNA size was estimated using the Fragment Analyzer or Femto Pulse. Sequencing was conducted on a PacBio Sequel IIe instrument, using version 2.0 sequencing reagents and operating on control software version 10.1.0.119549, with a movie collection time of 30 hours per 8M SMRT Cell.

UL-ONT Sequencing at the University of Washington (PR00251_PPA and Jim_GGO): Ultra-long-(UL-)ONT data were generated from the PR00251_PPA lymphoblast cell line and Jim_GGO fibroblast cell line according to a previously published protocol (Logsdon, protocols.io, 2020). Briefly, 3-5 x 10^7 cells were lysed in a buffer containing 10 mM Tris-Cl (pH 8.0), 0.1 M EDTA (pH 8.0), 0.5% w/v SDS, and 20 mg/mL RNase A (Qiagen, 19101) for 1 hour at 37°C. 200 ug/mL Proteinase K (Qiagen, 19131) was added, and the solution was incubated at 50°C for 2 hours. DNA was purified via two rounds of 25:24:1 phenol-chloroform-isoamyl alcohol extraction followed by ethanol precipitation. Precipitated DNA was solubilized in 10 mM Tris (pH 8.0) containing 0.02% Triton X-100 at 4°C for two days. Libraries were constructed using the Ultra-Long DNA Sequencing Kit (ONT, SQK-ULK001) with modifications to the manufacturer's protocol. Specifically, ~40 ug of DNA was mixed with FRA enzyme and FDB buffer as described in the protocol and incubated for 5 minutes at RT, followed by a 5-minute heat-inactivation at 75°C. RAP enzyme was mixed with the DNA solution and incubated at RT for 1 hour before the clean-up step. Clean-up was performed using the Nanobind UL Library Prep Kit (Circulomics, NB-900-601-01) and eluted in 225 uL EB. 75 uL of library was loaded onto a primed FLO-PRO002 R9.4.1 flow cell for sequencing on the PromethION, with two nuclease washes and reloads after 24 and 48 hours of sequencing.

The multidimensional scaling (MDS) analysis below using SNP distances between samples suggest the T2T ape samples, indicated by blue datapoint ("Ref"), are not necessarily outliers, at least among the samples used as part of the great ape genome diversity project[2-5] processed in **Supplementary Note VII** (**Supplementary Fig. I.1-5**). For the differences between Bornean and Sumatran orangutans, we computed the genome-wide average of pairwise identity between the genomes and found that between Bornean and Sumatran genomes we observe an average identity of 98.9%, which was 0.3-4% lower than within-species identities (**Supplementary Fig. I.6**): among Sumatran orangutans (99.2%) and between two haplotypes of Bornean orangutan (99.3%). The cell lines from two orangutans and chimpanzees were also authenticated as described in Makova et al. 2024[1].

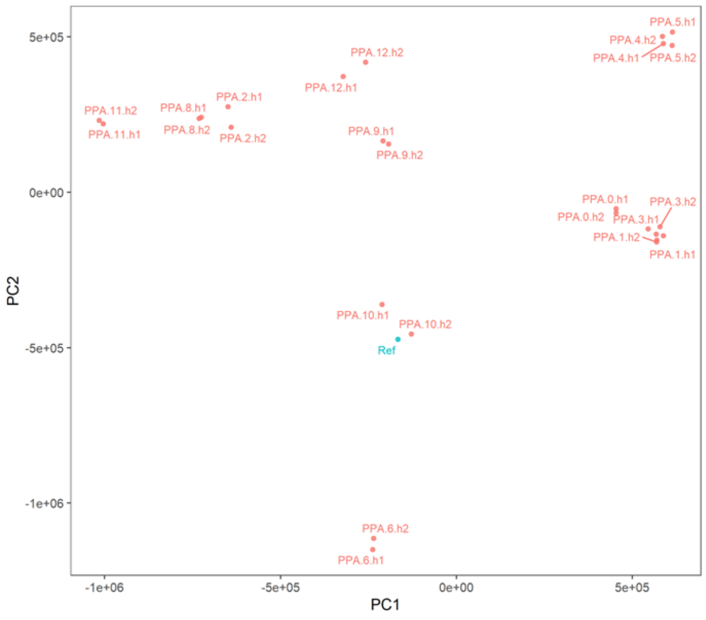

Supplementary Figure I.1. MDS plot using SNP distance matrix in bonobo (*Pan paniscus*).

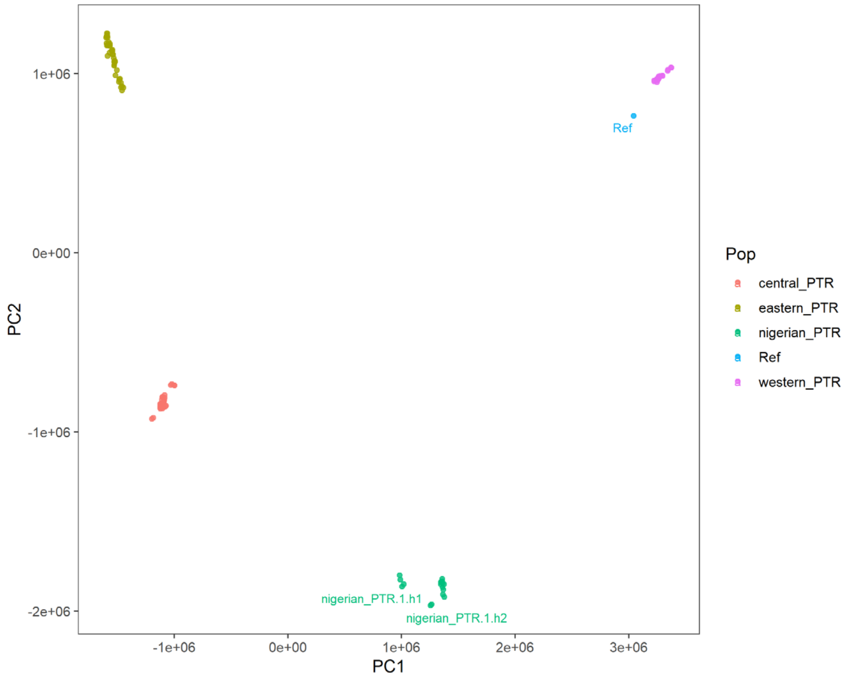

Supplementary Figure I.2. MDS plot using SNP distance matrix in chimpanzee (*Pan troglodytes*), suggesting the T2T chimpanzee genome is genetically related to western chimpanzee population (*Pan troglodytes verus*).

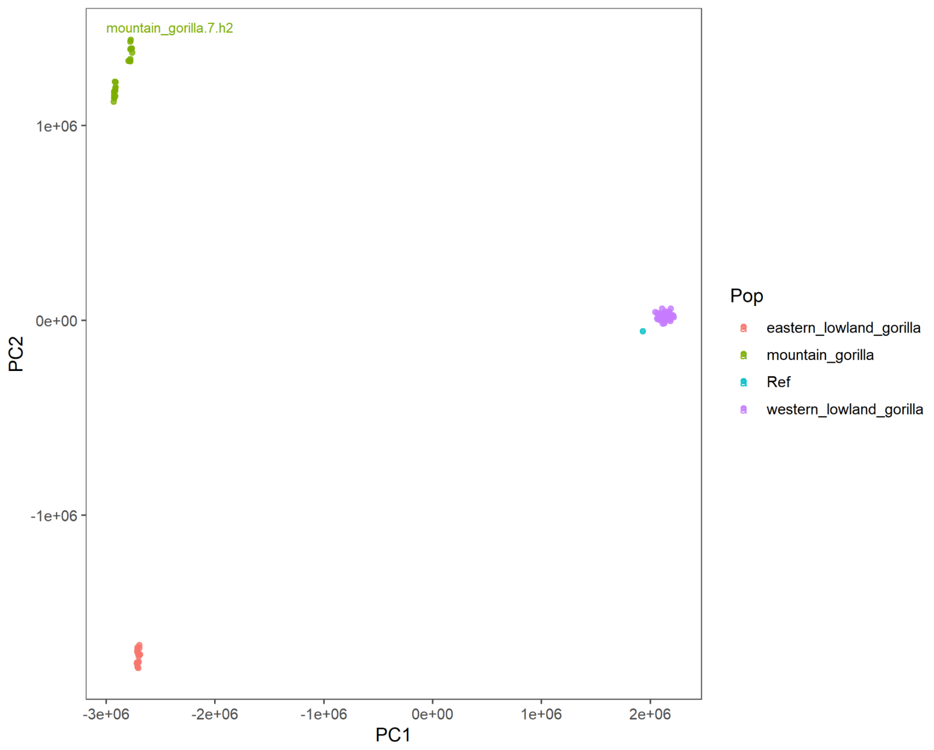

Supplementary Figure I.3. MDS plot using SNP distance matrix in gorilla, suggesting the T2T gorilla genome is closest to western lowland gorilla (*Gorilla gorilla gorilla*).

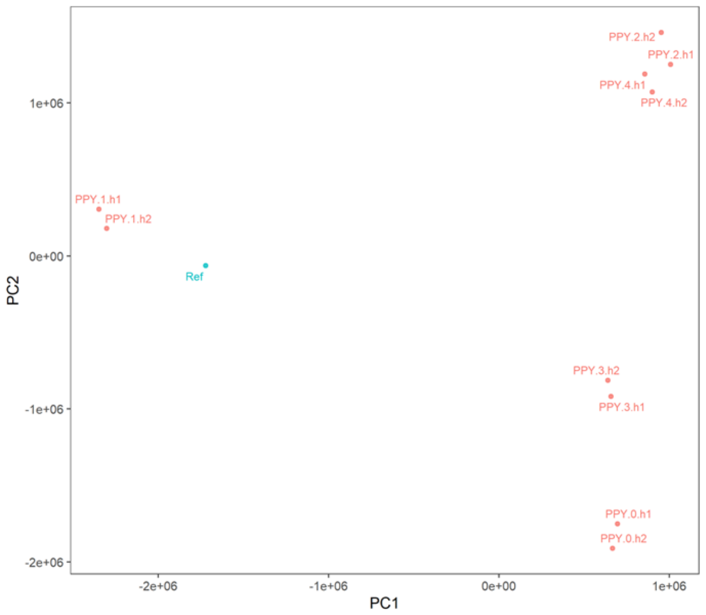

Supplementary Figure I.4. MDS plot using SNP distance matrix in Bornean orangutan (*Pongo pygmaeus*).

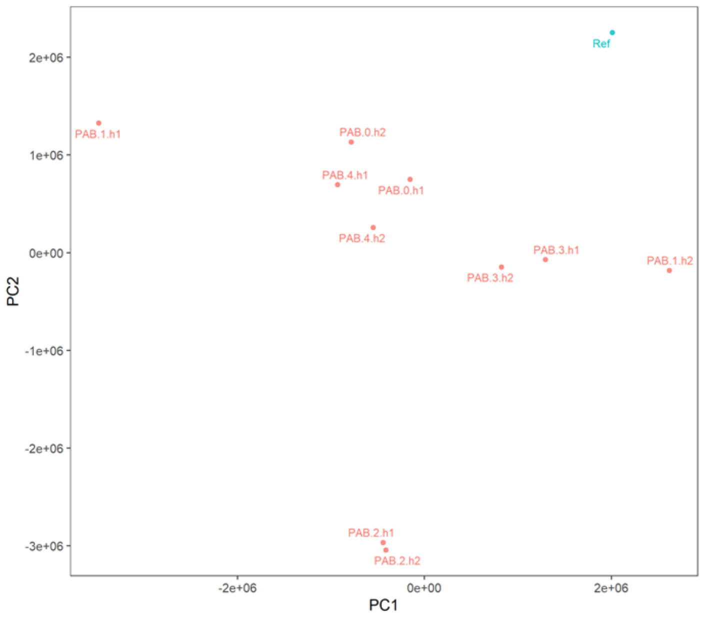

Supplementary Figure I.5. MDS plot using SNP distance matrix in Sumatran orangutan (*Pongo abelii*).

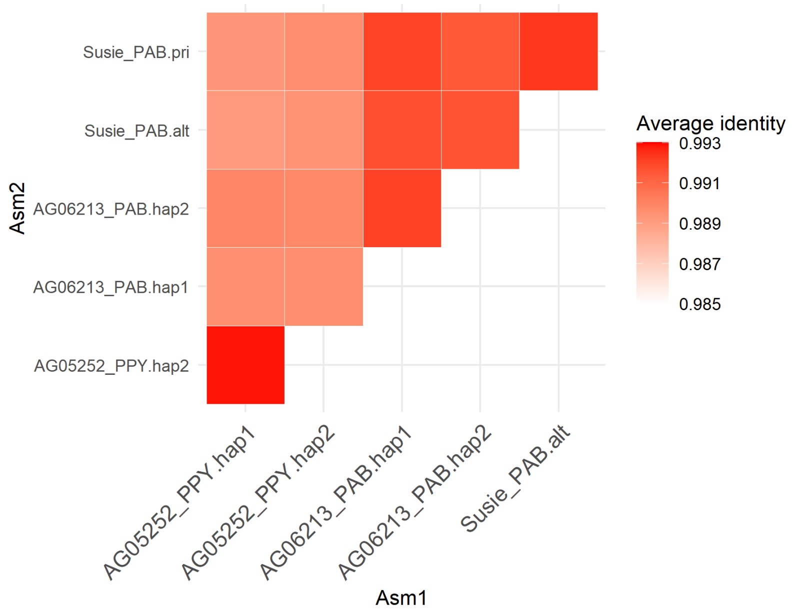

Supplementary Figure I.6. Genome-wide average identity between orangutan genomes.

II. Genome assemblies

Contributing authors:

Sergey Koren, Brandon Pickett, Arang Rhie, Dmitry Antipov, Julie Wertz, William T. Harvey, Sean McKinney, Mario Ventura, Adam M. Phillippy

Methods

The complete, haplotype-resolved assemblies were generated using a combination of Verkko[6] and expert manual curation. Parental-specific markers were generated using Merqury[7] with the commands:

```
cd maternal
ls *.fastq.gz > input.fofn
sh _submit_build.sh -c 30 input.fofn maternal
cd ../paternal
ls *.fastq.gz > input.fofn
sh _submit_build.sh -c 30 input.fofn paternal
cd ..
sh trio/hapmers.sh maternal/maternal.k30.meryl paternal/paternal.k30.meryl
```

Verkko v1.4.1 was run with the parameters --screen human and --trio maternal.hapmer.meryl paternal.hapmer.meryl for trios (bonobo and gorilla) using the *k*-mer databases built above or --hic1 *R1*fastq.gz --hic2 *R2*fastq.gz in the absence of trios (chimpanzee, orangutans, and siamang). Haplotype-consistent contigs and scaffolds were automatically extracted from the labeled Verkko graph, with unresolved gap sizes estimated directly from the graph structure (see Rautiainen et al.[6] for more details).

After the assembly was generated, manual interventions were employed to complete the Assembly. The ONT reads were re-aligned to the final graph using GraphAligner v1.0.17[8] with the command:

```
GraphAligner -t <cores> -g unitig-unrolled-unitig-unrolled-popped-unitig-normal-connected-tip.gfa -f split/ont<jobid>.fasta.gz -a aligned<jobid>.gaf --seeds-mxm-windowsize 5000 --seeds-mxm-length 30 --seeds-mem-count 10000 --bandwidth 15 --multimap-score-fraction 0.99 --precise-clipping 0.85 --min-alignment-score 5000 --hpc-collapse-reads --discard-cigar --clip-ambiguous-ends 100 --overlap-incompatible-cutoff 0.15 --max-trace-count 5 --mem-index-no-wavelet-tree
```

Using available information, such as parent-specific *k*-mer counts, depth of coverage, and node lengths, some artifactual edges could be removed and simple nonlinear structures resolved. For more complex cases, ONT reads aligned through the graph were used to select candidate resolutions consistent with the majority of alignments. In cases of unclear parental inheritance, nodes were arbitrarily assigned to a haplotype to avoid introducing gaps. Gaps filled by fewer candidate ONT sequences than Verkko's default of three were also patched at this stage after confirming the orientation and association of nodes via Hi-C links. The gap-filling sequences were added to the assembly graph as nodes using the same insert_aln_gaps.py script used internally by Verkko with parameters (minimum read support, distance from contig end) adjusted as appropriate to ensure a fill for each gap. A separate process was used to include assembly of the regions close to ribosomal DNA (rDNA) arrays. Initially, for each path ending at an rDNA sequence, the last reliable node present in only one path was selected. This was done manually, with the help of length, coverage, and graph structure. Then, Verkko's paths were extended with the help of improve_gaps_ont.py script. This script starts with unique nodes and extracts a set of ONT read alignments *S* that contain this node. Then, it iteratively adds the node most supported by alignments from *S*, if it is supported by 1.6-fold more reads than the second best, and the total number of supporting alignments is at least four. Graph structures which could not be resolved using the above methods were left as gaps in the assembly.

Once the paths for each chromosome were complete, Verkko was re-run to generate a new consensus with the commands:

```
cp  ../asm/6-layoutContigs/combined-alignments.gaf ./
cat ../asm/8-resolve/toalign_combined.gaf >> combined-alignments.gaf

cp  ../asm/6-layoutContigs/combined-edges.gfa ./
cat ../asm/8-resolve/gapfill_combined.gfa | grep '^L' >> combined-edges.gfa

ln -s ../asm/6-layoutContigs/combined-nodemap.txt

cp ../asm/6-layoutContigs/nodelens.txt ./
cat  ../asm/8-resolve/gapfill_combined.gfa | awk 'BEGIN \
     {
       FS="[ \t]+"; OFS="\t"; \
     } \
     ($1 == "S") && ($3 != "*") \
     { \
       print $2, length($3); \
     }' >> nodelens.txt

cat ../asm/8-resolve/gapfill_combined.gaf |awk '{print $1}' > ont.ids
```

```
grep -w -v -f ../asm/7-consensus/ont_subset.id tmp > ont.ids.extra
cp ../asm/7-consensus/ont_subset.fasta.gz ./
cat `ls ../asm/8-resolve/gapfill*fasta|grep -v hpc` | seqtk subseq - ont.ids.extra |pigz -c >>
ont_subset.fasta.gz

<path to verkko>/src/scripts/get_layout_from_mbg.py combined-nodemap.txt combined-
edges.gfa combined-alignments.gaf rukki.paths_with_short_arms.gaf nodelens.txt unitig-
popped.layout unitig-popped.layout.scfmap

<path to verkko>/lib/verkko/bin/layoutToPackage -layout unassigned-unitig.layout -
output packages/part###.cnspack -idmap  packages -partition 0.8 1.5 0.04 -reads
ont_subset.fasta.gz <hifi reads>  > packages.report touch packages.finished

# for each package in parallel
<path to verkko>/lib/verkko/bin/utgcns -V -V -V -import packages/part<jobid>.cnspack -
A packages/part<jobid>.fasta -C 2 -norealign -maxcoverage 50 -EM $MAXONT -e 0.05
-em 0.20 -l 3000 -threads 32 -edlib

<path to verkko>/lib/verkko/scripts/fasta_combine.py combine combined.fasta
packages.tigName_to_ID.map  unitig-popped.layout.scfmap  packages/part*.fasta
```

Lastly, short sequences, EBV, and mitochondrial sequences were identified using human reference and the Verkko screen-assembly.pl script with the identity threshold set to 90%. EBV was identified in bonobo, chimpanzee, and siamang.

Short arms of acrocentric chromosomes were scaffolded to the remaining chromosomes with Hi-C reads. Initially, potential short and long arms were discovered using length and proximity to rDNA containing nodes. Then, for each short arm *A*, we selected the corresponding long arm as the arm with the highest total number of Hi-C links connecting its nodes to *A*. Details of the algorithm are described in the Verkko 2.0 paper. The assembly was versioned as v1.4.1r and was subject for polishing and curation.

The initial assemblies were polished by adapting the process previously described in Mc Cartney et al.[9] and Rhie et al.[10] Briefly, short nucleotide variation (SNV) like errors were called from short and long reads with DeepVariant and filtered for correcting consensus and phasing errors using BCFtools and Merfin[11]. Pre- and post-polished assemblies were evaluated with *k*-mers from HiFi and Illumina reads with Merqury, along with read-coverage-based analysis using scripts from Mc Cartney et al.[9] Systematic errors were found close to the rDNA gap flanking sequences and were manually patched afterwards.

Read mapping

Unlike the X and Y chromosomes, the autosomes share more homology, making it difficult to uniquely map reads to the proper haplotype. This becomes challenging when phasing error persists in the underlying consensus, especially when collapsed haplotype errors exist in long stretches of a nearly homozygous region. Therefore, we added one more error type to target, in addition to the classic consensus errors—the phasing error.

First, HiFi and ONT read sets were aligned to the diploid genome (all-to-dip) as well as to each haploid genome (all-to-hap) with Winnowmap v2.03 using the pipeline from T2T-Polish (https://github.com/arangrhie/T2T-Polish/tree/master/winnowmap). The X was included to the paternal (or hap2) and Y to the maternal (or hap1) haploid genome in the all-to-hap alignment to prevent over-polishing of the highly diverged sex chromosomes. In brief, the top 0.02% repetitive 15-mers were collected using Meryl:

```
meryl count k=15 $ref output merylDB
meryl print greater-than distinct=0.9998 merylDB > repetitive_k15.txt
```

and mapped with Winnowmap and sorted, filtered for primary alignments with option -ax map-pb for HiFi and -ax map-ont for ONT reads:

```
winnowmap --MD -W repetitive_k15.txt -ax $map -I12g -t$cpus $ref $reads >
$tmp/$out.sam
samtools sort -@$cpus -m2G -T $tmp/$out.tmp -O bam -o $out.sort.bam $tmp/$out.sam
samtools view -F0x104 -@$cpus -hb $out.sort.bam > $out.pri.bam
```

The three reference versions (all-to-dip and two haplotypes for all-to-hap) were indexed for Illumina read mapping with BWA v0.7.17 (https://github.com/arangrhie/T2T-Polish/tree/master/bwa).

```
bwa index $ref
```

Each paired set of fastq files were provided for alignment, with duplicates removed with fixmate and SAMtools v1.17.

```
bwa mem -t $cpu $ref $r1 $r2 > $tmp/$out.sam
samtools fixmate -m -@$cpu $tmp/$out.sam $tmp/$out.fix.bam
samtools sort -@$cpu -O bam -o $out.bam -T $tmp/$out.tmp $tmp/$out.fix.bam
samtools index $out.bam
samtools markdup -r -@$cpu $out.bam $out.dedup.bam
samtools index $out.dedup.bam
```

The bam files were merged at the end for variant calling.

```
samtools merge -@ $cpu -O bam -b $lst $out.bam
samtools index $out.bam
```

Variant calling

Once the read alignment finished, Illumina and HiFi read alignments were used for variant calling with DeepVariant Hybrid mode (https://github.com/arangrhie/T2T-Polish/blob/master/deepvariant/_submit_mrg_hybrid_dv.sh). DeepVariant v1.5.0 was used in default mode.

```
samtools merge -@$cpu -O bam -o $BAM_HYBR $BAM_HIFI $BAM_ILMN
samtools index $BAM_HYBR
```

DeepVariant step 1 - make examples. For the all-to-dip alignment, MQ filter was lowered to 1 to include read alignments in the more homozygous region.

```
# for all-to-hap alignments
extra_args=""
# for all-to-dip alignments
extra_args="--min_mapping_quality 1"

seq 0 $((N_SHARD-1)) \
  | parallel -j ${SLURM_CPUS_PER_TASK} --eta --halt 2 \
  --joblog "logs/log" --res "logs" \
  make_examples    \
    --mode calling \
    --ref "${REF}" \
    --reads "${BAM}" \
    --examples $OUT/tfrecord@${N_SHARD}.gz $extra_args \
    --sample_name "$12" \
    --task {}
```

step 2 - call variants (this step was run on gpu nodes).

```
call_variants \
  --outfile "${CALL_VARIANTS_OUTPUT}" \
  --examples "$OUT/examples/tfrecord@${N_SHARD}.gz" \
  --checkpoint /opt/models/hybrid_pacbio_illumina/model.ckpt
```

step 3 - post process variants

```
postprocess_variants \
  --ref "${REF}" \
  --infile "${CALL_VARIANTS_OUTPUT}" \
  --outfile "$OUT/$OUT.vcf.gz"
```

For the ONT read alignments, PEPPER-MARGIN-DeepVariant v0.8 was used, as DeepVariant v1.5.0 was not available for R9 data. Similar to the hybrid mode, all-to-dip alignments were processed with mapping quality options --pepper_min_mapq 1 --dv_min_mapping_quality 1. For faster processing, this step was performed on each chromosome and the resulting VCF file was merged at the end with BCFtools v1.17.

```
# Per-chromosome
run_pepper_margin_deepvariant call_variant \
  -b $BAM -f $REF -o $OUTPUT_DIR \
  $MQ_OPT \
  -t $THREADS -r $REGION --ont_r9_guppy5_sup --gpu

# At the end, merge all per-chr VCFs
ls ${in}*/PEPPER_MARGIN_DEEPVARIANT_FINAL_OUTPUT.vcf.gz >
r9_files_to_mrg.list
bcftools concat -D -a --threads $cpus --no-version -Oz -o $out.vcf.gz -f
r9_files_to_mrg.list
bcftools index $out.vcf.gz
vcf_stats_report --input_vcf $out.vcf.gz --outfile_base $out
```

SNV correction

Variants were filtered with BCFtools v1.17 and Merfin v1.1 using *k*-mers from Illumina and HiFi. The overall diagram of the filtering is in **Supplementary Fig. II.7.** A custom script was made to run this filtering (https://github.com/arangrhie/T2T-Polish/blob/master/variant_call/snv_candidates.sh) and applied to each genome.

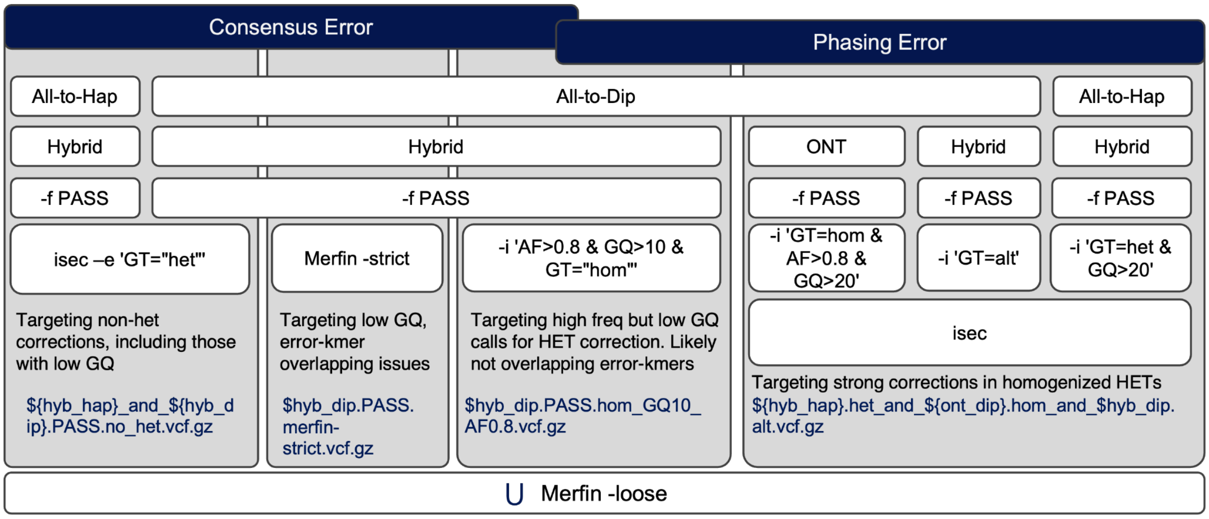

Supplementary Figure II.7. SNV-like error filtering criteria. All-to-Hap: Reads to each haplotype alignment. All-to-Dip: Reads to diploid (both haplotype) alignments. Hybrid: Merged HiFi and Illumina read alignments. U: Union. The rest are BCFtools and Merfin options to specify filtering conditions.

The final VCF file, snv_candidates.merfin-loose.vcf.gz, was used to make an internal 20231003 release (v1.4.2) with BCFtools consensus:

```
bcftools consensus -H1 --chain v1.4.1r_to_v1.4.2r.chain -f ${sp}_v1.4.1r.analysis-dip.fa
snv_candidates.merfin-loose.vcf.gz > ${sp}_v1.4.2r.analysis-dip.fa
```

Structural variant (SV) correction

While evaluating corrections, we identified regions flanking rDNA or gaps that contained clusters of needed corrections, which is difficult to properly correct with variant calling based polishing approaches. For correcting these errors, we revisited the assembly graph and manually created patch consensus to replace those regions. We primarily targeted regions around the rDNA, subtelomeric regions missing telomeres, and a few other regions flagged by the coverage-based analysis (described in the evaluation section).

Approximate regions containing the rDNA were identified in v1.4.1r assembly using a canonical human version of the 45S sequence[8] with MashMap3 v.3.1.1:

```
mashmap  -t $cpu --noSplit -q human_45S.fa \
  -r $sp_ver.analysis-dip.fa -s 13332 --pi 85 -f none -o 45S_to_$asm.mashmap.out

cat 45S_to_$asm.mashmap.out |\
  awk -v OFS='\t' '{print $6, $8, $9, $1, $(NF-1), $5}' |\
  awk -F ":" '{print $1"\t"$3}' |\
  awk -v OFS='\t' '{print $1, $2,$3, "45S", (100*$6), $7}' \
   > 45S_to_$asm.mashmap.bed
```

We identified an issue with Verkko consensus mis-assigning in regions with high-coverage repeats represented as gaps in the assembly. Once fixed, the paths were regenerated as described above to generate patch sequences. The patch sequences were trimmed down to contain a maximum of 500 kbp sequence and aligned to the target chromosome in the v1.4.1r version with wfmash. Alignments were compared to the SNV correction candidates and error *k*-mers from the hybrid *k*-mer database (described in the evaluation section). Patch sequences were further narrowed down manually to only contain target regions with less or no *k*-mers flagged as errors. Gaps flanking with rDNA have been resized to 1 Mbp in the new patch sequences.

wfmash v0.10.4 was run as follows:

```
wfmash --no-split -ad -t24 ${sp}_${ver}.$chr_hap.fa \
        patch.fa -s50000 -p95 > $out.sam
```

Once the target region to patch in v1.4.1 coordinates were identified, the corresponding query sequence was determined from the wfmash alignments. If the patch sequences had to be split at the gap, for later adjusting the gap size, both sides were considered to make one patch sequence (clip) and grouped. A custom script (https://github.com/arangrhie/T2T-Polish/blob/master/patch/samRefPos2QryPos.jar) was used to retrieve the corresponding query position from a given reference position:

```
if [[ $telo == "clip" ]]; then
  target1=`echo $target | awk -F "|" '{print $1}'`
  samtools view ${chr_hap}_fix_to_$ver.bam |\
    java -jar -Xmx1g samRefPos2QryPos.jar - $target1 > $chr_hap.patch.bed
  target2=`echo $target | awk -F "|" '{print $2}'`
  samtools view ${chr_hap}_fix_to_$ver.bam |\
    java -jar -Xmx1g samRefPos2QryPos.jar - $target2 >> $chr_hap.patch.bed
else
  samtools view ${chr_hap}_fix_to_$ver.bam |\
    java -jar -Xmx1g samRefPos2QryPos.jar - $target > $chr_hap.patch.bed
fi
```

Patch replacements were created in VCF format to contain target sequence in the REF field and the new patch sequence in ALT field:

```
## Per $chr_hap vcf
chr=`head -n1 $chr_hap.patch.bed | awk '{print $1}'`
pos=`head -n1 $chr_hap.patch.bed | awk '{print ($2+1)}'`
ref=`cat ref.seq`
alt=`cat qry.seq`
cat $sp_ver.header.vcf > $sp_ver.$chr_hap.rDNA_patch.vcf
echo -e "$chr\t$pos\t.\t$ref\t$alt\t1\t.\t.\tGT\t1/1" >> $sp_ver.$chr_hap.rDNA_patch.vcf
```

Missing telomeric sequences at the end of chromosomes were found using the 'telo -d 10000' function of seqtk v1.3, and patch sequences were made in the same way as the rDNA locus patches.

Lastly, SNV correction candidates overlapping the target region for patching were removed, and merged with the patch sequences. The resulting VCF was re-applied to the v1.4.1r version with BCFtools consensus as follows:

```
bcftools consensus -c $sp.${OLD}_to_$NEW.chain \
  -f ../$sp/$sp_ver.analysis-dip.fa -HA \
  $sp_ver.snv_sv_edits.vcf.gz > $sp_new.analysis-dip.fa
```

Scripts used for rDNA patch and telomere patch VCFs are available on https://github.com/arangrhie/T2T-Polish/tree/master/patch, make_rDNA_patch.sh and make_telo_patch.sh. Script for excluding SV edits and merging the SNV edits to make the final consensus is available as merge_snv.sh.

Haplotype assignment, chromosome orientation, and numbering

Chromosome numbers and orientation were identified using prior markers established for each species. Sequences were renamed to contain the species-specific chromosome number and human ortholog number and were reversed accordingly to have the p-arms at the beginning. For the non-trio species, haplotype numbers were reassigned to keep the rDNA containing haplotype as haplotype 1 if the partnering chromosome had no rDNA, or the more continuous (telomeres found on both ends), less gaps, less errors assigned as haplotype 1. For sex chromosomes, chrX was always assigned to haplotype 1, and chrY as haplotype 2, respectively. Haplotype 1 assemblies and the Y chromosome were regrouped as the primary assembly set for convenience if a linear representation of the species was needed. For the gorilla and chimpanzee, which had parental information available, haplotypes were assigned as mat or pat for maternal or paternal, respectively. Using the same criteria for choosing haplotype 1 and the primary haplotype in the non-trios, the haplotypes were reassigned to keep the more continuous, accurate haplotype in the primary assembly set.

Assessment of the genome assembly

Alignments were generated by mapping reads onto each assembly using the assembly_eval pipeline (https://github.com/EichlerLab/assembly_eval). Minimap2 (v2.26 for NucFreq) or Winnowmap (v2.03 for Flagger) was used for the alignment with standard parameters.

Flagger (v0.3.3) (https://github.com/mobinasri/flagger) was run using the alignment file containing HiFi reads mapped back to each assembly. The pipeline was run using flagger_end_to_end_with_mapping.wdl file deposited in the repository.

NucFreq was run using HiFi reads as a part of the assembly_eval pipeline (https://github.com/EichlerLab/assembly_eval). Collapses are defined as locations where the second most common base had a depth of coverage greater than 5, and Duplicated/HiFi-deplete are defined as regions of the assembly with 0 or decreased read coverage by HiFi reads.

QV was estimated using Meryl v1.4.1 and Merqury commit 01a39a6 using a hybrid database of 31-mers collected from Illumina and HiFi reads. Hybrid databases were made as described in https://github.com/arangrhie/T2T-Polish/tree/master/merqury. Switch error rate was estimated using parental Illumina 31-mers, if available.

Chromosome nomenclature

To accurately determine proper nomenclature for chromosomes based on the centromeric position, we employed three approaches: a ratio-based approach comparing the lengths of the long (l) and short (s) arms in base pairs (r=l/s), the centromeric index-based approach comparing short arm to the total chromosome length (c) (I=100s*c) and the centromeric index-based approach using the total chromosome length without the centromeric sequence length (c') (I'=100s*c') as per the classical definitions outlined by the Denver Study Group in 1960[13] and by Levan et al. in 1964[14]. To integrate new sequence data with traditional cytogenetic information to best define the arms lengths, the centromeric regions had to be excluded from the total chromosome length calculations for both the long and short arms. Traditionally, the centromere was not included in size measurements due to its over-condensed nature; rather, it was solely identified along the chromosomes. Consequently, we focused solely on the lengths of the q and p arms, thereby circumventing also the highly variable centromeric sequences present in each chromosome.

Additionally, we used the above-mentioned methods on human chromosomes and find the ratio criteria was the most reliable using the ranges: $0{,}7<r<1.58$ for metacentric (M), $1.58<r<3$ for submetacentric (S) and $r>3$ for acrocentric (A).

Employing this methodology (**Supplementary Fig. II.8** and **Supplementary Tables II.6-11**), we successfully categorized each chromosome and identified the acrocentric chromosomes in chimpanzees, bonobos, gorillas, and orangutans (red arrows).

To avoid confusion with nomenclature, we referred to great ape chromosomes considering the human synteny instead of species-specific nomenclature. For example, PTR chromosome 2, being homologous to human chromosome 3, was assigned as chromosome 3 (III in Roman numerals in **Supplementary Fig. II.8**). The only exception to this is chromosome 2 where we used 2A and 2B for 2p-2q and 2q chromosome syntenic blocks, respectively.

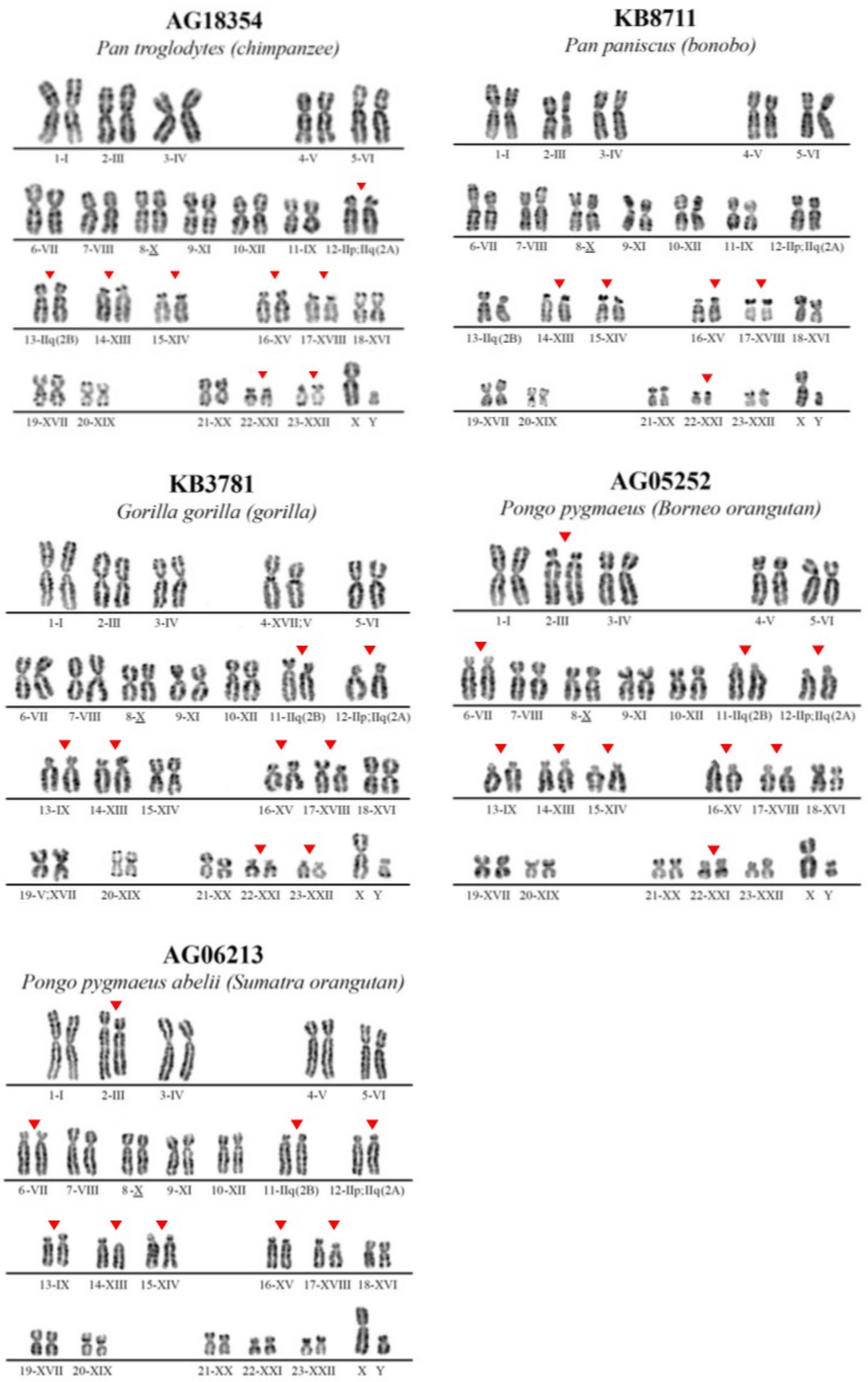

Supplementary Figure II.8. Karyotyping of the ape tissues.

III. Genome alignment and sequence divergence

Contributing authors:

Peter H. Sudmant, Giulio Formenti, Erin K. Molloy, Wenjie Wei, Andrea Guarracino, Bryce Kille, Erik Garrison, Wenjie Wei, Cole Shanks, Prajna Hebbar, Glenn Hickey, Benedict Paten

Methods

Pangenome alignment

To perform an all-vs-all alignment across the entire set of primate genomes, we applied an iterative all-vs-one approach using wfmash (https://github.com/waveygang/wfmash). We mapped all genomes against a single target genome at a time, repeating this process for each genome in the dataset as the target. This strategy breaks down the computationally intensive all-vs-all alignment into more manageable all-vs-one steps, enabling efficient parallel processing. For the mapping step, we used MashMap3 (https://github.com/marbl/MashMap), integrated into wfmash. Each iteration works by splitting each query genome into overlapping 5 kbp segments and mapping each segment to the current target. The mappings are then filtered to keep only those with >70% identity, merging those closer than 20 kbp. Next, we used the mappings as input to guide the alignment process in wfmash. This results in aligned PAF files for each target sequence that include base-level alignment as CIGAR strings (**Supplementary Fig. III.9a**). Finally, we used paf2chain (https://github.com/AndreaGuarracino/paf2chain) to convert the aligned PAF files into CHAIN format. We repeated this procedure to map and align the HPRC year 1 human genome assemblies against each primate target genome. This allows comparative analyses between the primate genomes and the diverse set of human haplotypes.

Implicit pangenome graph (impg)

Pangenome graphs condense many-way sequence relationships (similarities) into a graphical model that avoids redundancy. These graphs imply alignments: in principle you can extract alignments back out of the graph, which exactly encompass the relationships between individual genomes seen in the graph. And the reverse is true as well: a set of alignments between sequences imply a graph.

A common technique in algorithms is to avoid a hard problem by simulating its result. For instance, interval trees are data structures used to index and query range overlaps. They are used in BEDTools and alignment algorithms. But they are very heavy to instantiate. In practice, it is always better to use an implicit interval tree[15]. We build on this same data structure to create an implicit pangenome graph out of a set of base-level alignments between all pairs of haplotypes in the T2T-primates collection.

The implicit pangenome graph lets us produce specific products and query the pangenome without instantiating it. These include:

- Graph subset: using a compressed index of the alignments, the impg tool lets us extract all subsequences of genomes that match any locus on any genome in the pangenome. This matching is transitive and exactly equivalent to what would be obtained from subset operations on a full graph build. (Here we show the MHC and 8p23.1 inversion extracted from the implicit graph using this technique.)
- Reference-relative multiple alignments in a "pseudo-MAF" format usable by many downstream comparative genomics tools.
- Divergence: also from MAFs (multiple alignment formats) / flip side of conservation.
- Conservation: from the MAFs we build a track of conservation relative to each genome in the cohort.

Our alignment and pangenome analysis approach has some advantages over current standards (e.g., Cactus) in being tree-free, repeat-masker free, capable of considering all haplotypes, and easy to parallelize. The approach is fundamentally pangenomic in that we make results in any frame of reference.

Subgraph analysis from implicit graph

To efficiently extract subgraphs and analyze the primate pangenome without constructing a full graph, we used impg (implicit pangenome graph). impg is a tool that projects sequence ranges through many-way pairwise alignments, such as those built by wfmash and minimap2, to identify homologous loci across multiple genomes.

impg uses coitrees (implicit interval trees) to provide efficient range lookup over the input alignments. CIGAR strings are converted to a compact delta encoding, enabling fast and memory-efficient projection of sequence ranges through alignments. The output is provided in BED, BEDPE, and PAF formats, making it straightforward to extract FASTA sequences for downstream use in multiple sequence alignment or pangenome graph building.

To build the impg index, we first generated a compressed index of the wfmash alignments as a tiny auxiliary file that indexes a bgzipped PAF file. We then used impg to extract sequences corresponding to specific loci of interest, such as the MHC and 8p23.1 inversion regions (**Supplementary Fig. III.9**), from the primate genomes.

The resulting sequences were then used for downstream analyses, such as building local subgraphs with PGGB and visualizing them with odgi, as described in the previous sections. This approach allowed us to efficiently analyze specific regions of interest in the primate pangenome without the need to build and manipulate a full pangenome variation graph. Future work will enable distributed construction of large comparative genomic type pangenome graphs but is here applied in an exploratory way.

By using impg to extract homologous sequences and build local subgraphs, we were able to perform detailed comparative analyses of specific genomic regions across the primate genomes

in a computationally efficient manner. This approach demonstrates the utility of impg for managing and analyzing large collections of genomes in a pangenomic context.

8p23.1 subgraph generation

Human 8p23.1 is interesting due to its defensin gene content and high polymorphism in humans and across the primate lineage. To generate a subgraph of the 8p23.1 locus across multiple primate genomes, we first extracted the locus with flanking sequence from the human GRCh38 reference genome using coordinates grch38#1#chr8:5748405-13676927. We then used a wfmash whole-genome alignment of primate genomes to identify syntenic regions in the other primate genomes. The wfmash alignments were processed into an implicit graph (impg) data structure. We used impg to collect the sequences corresponding to the 8p23.1 locus from each primate genome:

```
impg -p primates16.20231205.paf.gz -t 64 -r grch38#1#chr8:5748405-13676927 -x |
bedtools sort | bedtools merge -d 1000000 | awk '$3 - $2 > 2000000 { print $1":"$2"-
"$3 }' > primates.8p23.1.merged-1m.gt2m.regions
```

```
samtools faidx -r primates.8p23.1.merged-1m.gt2m.regions ../primates16.20231205.fa.gz
| bgzip -@24 -l9 >../primates16.20231205.8p23.1.merged-1m.gt2m.fa.gz
```

These sequences were then built into a local sequence graph using PGGB with the following parameters: -c 10 -t 96 -p 90 -s 10k -k 19. -c 10 forces a high rate of multi-mapping between homologous sequences, which we found necessary to build a compact representation of the repetitive locus that modulates the recurrent polymorphic inversion.

To aid visualization in 1D, the graph was sorted using odgi sort, taking the chm13 sequence path as the reference order, with the following parameters: -t 96 -p Y -H <(odgi paths -i ${graph} -L | grep chm13) -x 500 -P -K 0.5 -v 10000000000000 -g 0.0000000000000001

The sorted graph was visualized using odgi viz (**Supplementary Fig. III.9b**) to generate a rendering showing the graph topology colored by path inversion status relative to the chm13 order and a rendering showing the graph topology colored by local graph depth.

MHC locus subgraph generation

To generate a subgraph of the MHC locus across multiple primate genomes, we first extracted the locus with flanking sequence from the chm13 reference genome using coordinates chm13#1#chr6:28385000-33300000. We then used the wfmash whole-genome alignment of primate genomes to identify syntenic regions in the other primate genomes. The wfmash alignments were processed into an implicit graph (impg) data structure using the following command:

```
impg -p primates16.20231205_wfmash-v0.12.5/chm13\#1.aln.paf -q
chm13#1#chr6:28385000-33300000 | tee mhc.bed
```

The resulting sequences were then built into a local sequence graph using PGGB with the following parameters:

> -i /lizardfs/erikg/primates/primates16.20231205.mhc.impg.merge-500kb.fa.gz -o mhc.primates.'$i' -t 96 -p 90 -s 10k -k 47

The resulting graph was visualized using odgi (**Supplementary Fig. III.9E-H**). The graph was sorted and visualized as described for the 8p23.1 locus above to generate renderings showing the graph topology colored by path inversion status and by local graph depth.

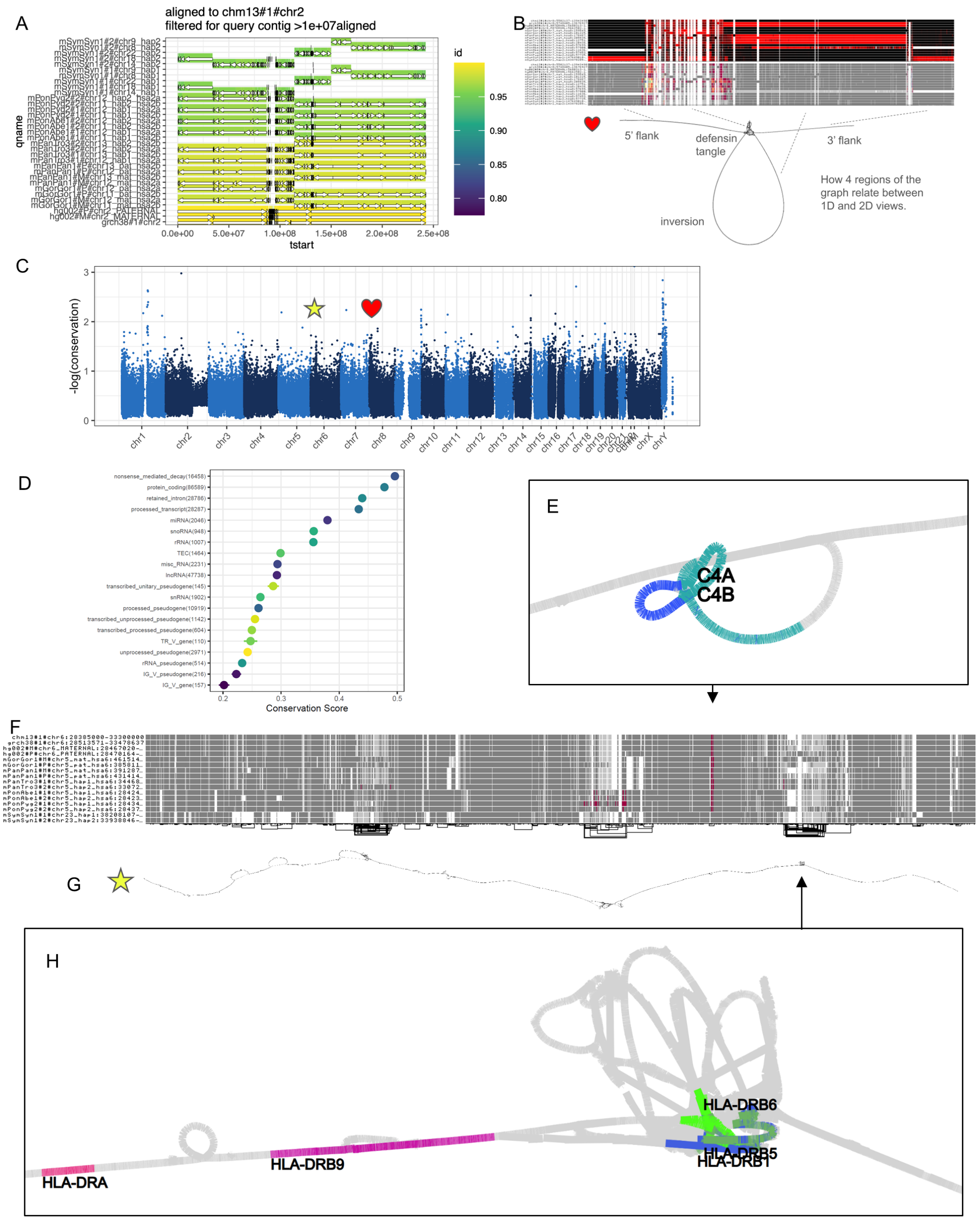

Supplementary Figure III.9. Analyses on the implicit pangenome graph. Analyses are based on a 16-way alignment of all T2T assemblies in the present study, CHM13, GRCh38, and HG002 to all others. Each genome serves as a reference, allowing for universal annotation of conservation and across all assemblies. (A) Alignments show the fusion of acrocentric chromosomes to form chromosome 2 in the human lineage. (B) Extraction of the pangenome graph around an inversion located on human 8p23.1. The inversion is polymorphic across great ape clade, as shown by visualizations with odgi viz (top, with black and red indicating relative orientation in the graph). (C) Conservation scores relative to CHM13, showing a drop in conservation at the MHC (star)

and also in the 8p23.1 locus (heart). Higher values indicate lower conservation. A single random haplotype per species, but including all sex chromosomes, was used for PhastCons analyses. (D) Conservation scores relative for specific gene annotation classes. Transcripts with retained introns, NMD (nonsense-mediated decay) transcripts, and protein-coding sequences are the most conserved, while pseudogenes and IGV genes are the among least conserved classes. The relatively low conservation observed for snRNA group is expected to be due to lack of power over short branch lengths. (E-H) Diverse views into an extraction of the MHC from the implicit pangenome graph. (E) A subgraph around the C4A/C4B locus suggests that there are distinct versions of the inserted endogenous retroviral sequence (nested loop). (F) A 1-D odgi viz rendering shows expected pattern of diversity in MHC, with structures mostly conserved with the exception of large deletions seen in gibbon. (G) A rendering of the entire region with odgi draw, demonstrating its collinearity across the clade. (H) MHC class II cell surface receptor genes show rapid structural evolution and diversity across the clade, as shown by the tangled structure around HLA-DRB1, HLA-DRB5, and HLA-DRB6.

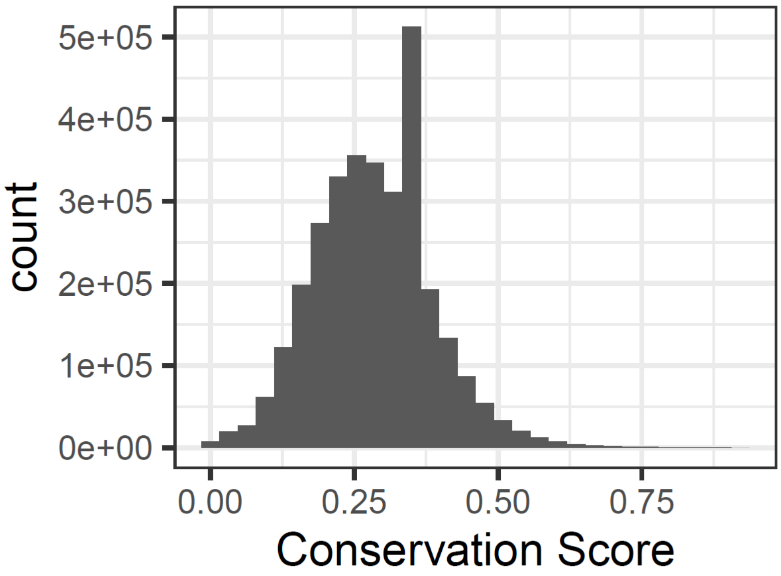

Supplementary Figure III.10. Conservation scores binned in 1000 base-pair windows.

wgatools pseudo-MAF (pMAF)

We generated all-to-all alignments using wfmash (https://github.com/waveygang/wfmash), producing one PAF with each assembly as a target/reference. Next, we used wgatools (github.com/wjwei-handsome/wgatools) to filter the PAF (16 PAFs) for min alignment len >10 Mbp and then convert PAF to MAF (384 MAFs).

SNP vs. gap divergence

We computed SNV and gap divergence from each pairwise alignment, estimated with wfmash v.0.13, for 1 Mbp segments running across the target haplotype. We report the mean and median SNV and gap divergence. We also binned segments based on SNV and gap divergence to create density plots (**Supplementary Fig. III.11-12**). SNV divergence is defined as the fraction of positions in the target haplotype where the two haplotypes are in different nucleotide states. Gap divergence is defined as the fraction of positions in the target haplotype that are not aligned to the other haplotype, which could be due to biological processes (e.g., gene loss/gain and insertions/deletions), missing data, or technical problems (e.g., alignment failure due to SVs, repetitive elements, etc.).

Autosome SNV divergence between human and nonhuman primates (NHPs) was lowest for human-chimpanzee and human-bonobo (0.15-0.16%), then human-gorilla (0.19-0.20%) and lastly human-Sumatran orangutan (0.36%). However, autosome gap divergence showed different trends, as it was highest for human-gorilla (17.9-27.3%). The within-species autosome gap divergence was also highest for gorilla (13.8%), which could be due to the assembly (larger number of contigs), SVs, or mobile elements. Within-species autosome SNV divergence was lowest for human (0.16%), followed by chimpanzee (0.27%), orangutan (0.35%), bonobo (0.36%), and lastly gorilla (0.58%). Trends are similar for X and Y chromosomes, with divergence values being lower for X and higher for Y.

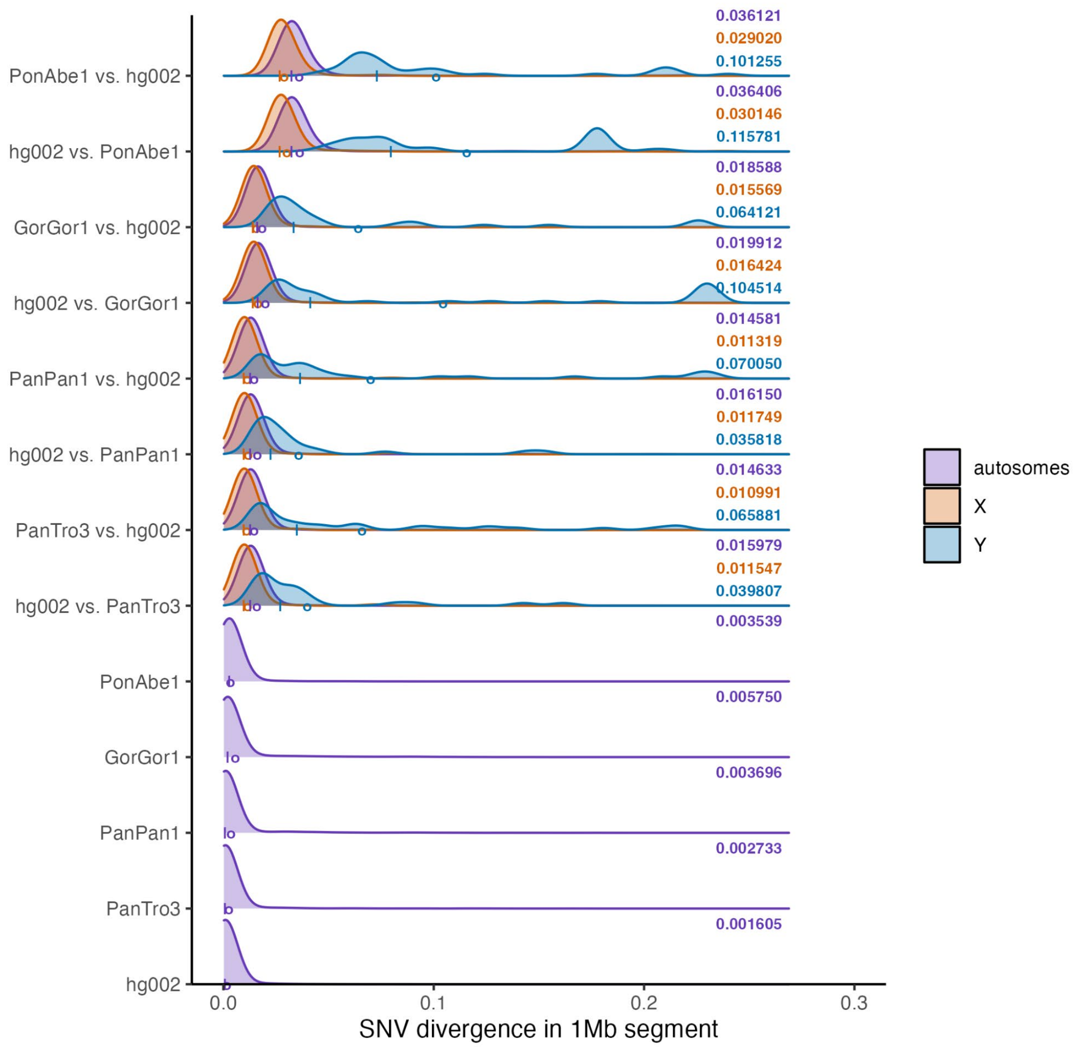

Supplementary Figure III.11. Plots show 1 Mbp segments binned by SNV divergence for each pairwise alignment (note that density, rather than counts, are shown). The maternal or primary haplotype is used for comparisons between human and NHP haplotypes. The second haplotype listed was aligned to the first/target haplotype (note that "A vs. B" and "B vs. A" are different pairwise alignments because the former includes the entire A haplotype with no gaps and the latter includes the entire B haplotype with no gaps). Density plots are broken down according to whether segments come from an autosome, the X chromosome, or the Y chromosome. Mean SNV divergence is reported for these three cases (numeric values and circles); medians are also shown (| characters).

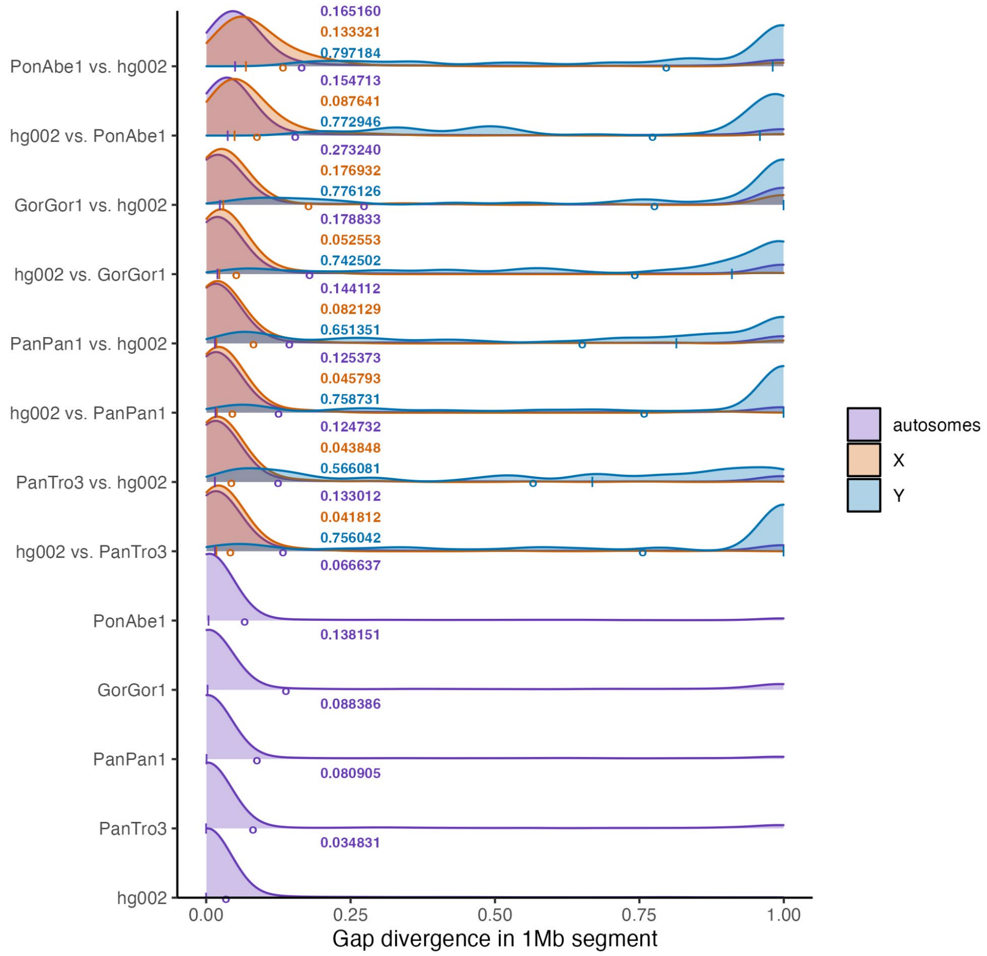

Supplementary Figure III.12. Plots show 1 Mbp segments binned by gap divergence for each pairwise alignment.

Conservation analyses

We generated per-base conservation scores and conserved elements tracks using PhastCons[16] v1_5 with two approaches, following the PhastCons HOWTO (http://compgen.cshl.edu/phast/phastCons-HOWTO.html) as described in Secomandi et al[17]. A single random haplotype aligned to CHM13 was selected for each species/chromosome pair, including all sex chromosomes. In the first approach, we used default parameters (unsupervised EM learning), splitting alignments into 1 Mbp chunks and combining predictions. Since this approach estimated high conservation scores for the majority of the genome (mean 0.964), suggesting that the EM algorithm could not distinguish between constrained and neutrally evolving regions for such closely related species and relatively small sample size, we devised a second approach. For the second approach, we applied a fourfold degenerate site model, using entire alignments for all chromosomes except for chromosome 2, which was split into 1 Mbp

chunks. In this approach, we inferred one model using all 4D sites across chromosomes and then applied it genome-wide. We provided strong priors to the model by fine-tuning PhastCons HMM model parameters --target-coverage and --expected-length using Grid Search to jointly maximize enrichment (Jaccard index) of CHM13 CDS sequences across all chromosomes. As the model was inferred genome-wide and the same final model parameters were applied to all chromosomes, conservation scores can be directly compared between chromosomes. The initial model was estimated with maximum likelihood and a tree from TimeTree (https://timetree.org). Exact software command and model parameters can be found in the GitHub repository (https://github.com/T2T-apes/ape_pangenome/tree/main/conservation).

We analyzed the conservation scores generated from the fourfold degenerate-sites-based model in several different ways. First, we assessed the distribution of conservation scores binned into 1000 base-pair windows (**Supplementary Fig. III.10**). Conservation scores obtained with the fourfold degenerate site model were strongly skewed towards 0 (mean 0.276) as expected given most of the genome should not be under purifying constraint. Conservation scores differ among genomic features indicating that the elements that we expect to be under purifying selection (e.g., protein-coding genes, rRNAs) are at a higher level of conservation than the background score (**Supplementary Fig. III.9D**). We next assessed conservation over coding and noncoding genes using the Comparative Annotation Toolkit (CAT) liftoff track from UCSC from T2T-CHM13 of GENCODE genes. Gene conservation was distributed as expected with nonsense-mediated decay-associated transcripts, protein-coding genes, and retained introns, exhibiting the highest conservation and various classes of pseudogene exhibiting the lowest. Conservation scores genome-wide revealed several hotspots of reduced conservation, including the rapidly evolving MHC locus. Hotspots additionally tended to correspond to the subtelomeric ends of chromosomes and the Y chromosome additionally showed reduced constraint (**Supplementary Fig. III.9C**).

Minigraph-Cactus pangenome graphs

We constructed two pangenome graphs using Minigraph-Cactus[18]. The first of 10 African ape haplotypes, includes diploid assemblies for chimpanzee, bonobo, gorilla and T2T-HG002, as well as hg38 and hs1 on which it is referenced. The second graph was constructed from diploid Bornean and Sumatran orangutan genomes (4 haplotypes total) and is referenced on the primary Bornean orangutan assembly. In both cases, the graphs were constructed all at once, rather than being split by reference chromosome, as was previously done for the HPRC[19], in order to better account inter-chromosomal alignments.

The graphs were constructed on a Slurm cluster using Cactus v2.7.1 and the following commands.

```
TOIL_SLURM_ARGS="--partition=long --time=8000" cactus-
pangenome ./js-pg ./10-t2t-apes-mc-2023v2.seqfile --outDir
10-t2t-apes-mc-2023v2 --outName 10-t2t-apes-mc-2023v2 --
reference hs1 hg38 --noSplit --gbz clip full --gfa clip
```

```
full --xg clip full --odgi --vcf --giraffe clip --haplo
clip --vcfReference hs1 hg38 --logFile 10-t2t-apes-mc-
2023v2.log --batchSystem slurm --coordinationDir /data/tmp
--caching false --batchLogsDir ./batch-logs --consMemory
1500Gi --indexMemory 1500Gi --mgMemory 500Gi --mgCores 72 -
-mapCores 8 --consCores 128 --indexCores 72 --giraffe clip


TOIL_SLURM_ARGS="--partition=long --time=8000" cactus-
pangenome ./js-pg ./4-t2t-orangs-mc-2023v2.seqfile --outDir
4-t2t-orangs-mc-2023v2 --outName 4-t2t-orangs-mc-2023v2 --
reference mPonAbe1_pri mPonAbe1_alt --noSplit --gbz clip
full --gfa clip full --xg clip full --odgi --vcf --giraffe
clip --haplo clip --vcfReference mPonAbe1_pri mPonAbe1_alt
--logFile 4-t2t-orangs-mc-2023v2.log --batchSystem slurm --
coordinationDir /data/tmp --batchLogsDir ./batch-logs --
consMemory 1500Gi --indexMemory 1500Gi --mgMemory 500Gi --
mgCores 72 --mapCores 8 --consCores 128 --indexCores 72 --
giraffe clip
```

Note that the input, output including UCSC Genome Browser track hubs, and all steps to reproduce Minigraph-Cactus pangenomes can be found at https://cglgenomics.ucsc.edu/february-2024-t2t-apes/.

The statistics of these graphs, alongside the HPRC v1.1 Minigraph-Cactus graph are as follows:

Supplementary Table III.15. Pangenome graph statistics. "Length" refers to the total length of all nodes in the graph and "Avg. Clipped" is the amount of sequence (bp), on average, clipped out of the graph for each non-reference genome.

Pangenome	Haplotypes	Reference	Ref. Length	Nodes	Edges	Length	Avg. Clipped
HPRC v1.1	90	T2T-CHM13	3117292070	93165628	128451813	3338032439	166761108
African Apes	10	T2T-CHM13	3117292070	264554139	361346441	3383639539	380296289
Orangutan	4	B. Orang (primary)	3259853530	69685551	94270798	3350249901	193691200

Minigraph-Cactus produces VCF output alongside the graph representations. We used `bcftools norm -f`, `vcf-bub -l 0 -a 100000` then `vcfwave -I 1000` from ghcr.io/pangenome/pggb:202402032147026ffe7f for normalization, then Truvari v4.2.2 to merge similar SVs: `truvari collapse -r 500 -p 0.95 -P 0.95 -s 50 -S 100000`. Note that multiallelic sites were split with `bcftools norm -m -any | bcftools`

`sort before truvari` and remerged with `bcftools norm -m +any | bcftools sort after truvari`. Note that these VCFs exclude sites with variants >100 kbp. The number of variants in each VCF, again including HPRC v1.1, for comparison is as follows:

Supplementary Table III.16. The number of variant sites in pangenome VCFs. SVs include sites with an allele of length >50 bp and ≤100 kbp.

Pangenome	SNPs	MNPs	Indels	SVs
HPRC v1.1	22237802	799337	5694302	231751
African Apes	66573699	3541028	8589725	310406
Orangutan	18381520	1374958	3161356	107778

MAF files were exported from the pangenome graphs using `cactus-hal2maf`.

```
for i in hs1 hg38 ; do TOIL_SLURM_ARGS="--partition=long --
time=8000" cactus-hal2maf ./js ./10-t2t-apes-mc-
2023v2.full.hal ./10-t2t-apes-mc-2023v2.${i}.maf.gz --
filterGapCausingDupes --refGenome $i --chunkSize 500000 --
batchCores 64 --noAncestors --batchCount 16 --batchSystem
slurm --caching false --logFile ./10-t2t-apes-mc-
2023v2.${i}.maf.gz.log --batchLogsDir batch-logs-16apes --
coordinationDir /data/tmp ;done
```

```
for i in GCA_028885655.2 GCA_028885685.2 ; do
TOIL_SLURM_ARGS="--partition=long --time=8000" cactus-
hal2maf ./js ./4-t2t-orangs-mc-2023v2.full.hal ./4-t2t-
orangs-mc-2023v2.${i}.maf.gz --filterGapCausingDupes --
refGenome $i --chunkSize 500000 --batchCores 64 --
noAncestors --batchCount 16 --batchSystem slurm --caching
false --logFile ./4-t2t-orangs-mc-2023v2.${i}.maf.gz.log --
batchLogsDir batch-logs-16apes --coordinationDir
/data/tmp ; done
```

We computed coverage statistics for these alignments using `taffy coverage` (as included in Cactus v2.7.1) and aggregated them into the following table:

Supplementary Table III.17. Alignment coverage of T2T-CHM13 (hs1) in the African ape pangenome.

Region	Length	Query	Aligned (pct)	Identical (pct)	Aligned 1:1 (pct)	Identical 1:1 (pct)
Autosomes	2900572475	GRCh38	92.34	92.24	92.15	92.04
Autosomes	5801128381	HG002	93.59	93.48	93.17	93.07
Autosomes	5801111812	Chimp	89.35	88.17	88.96	87.78
Autosomes	5801111812	Bonobo	89.3	88.12	88.88	87.71
Autosomes	5801111812	Gorilla	88.29	86.81	87.62	86.14
X	154259566	GRCh38	96.34	96.28	96.33	96.27
X	154259566	HG002 Mat	97.72	97.67	97.71	97.66
X	154259566	Chimp Pri	94.85	93.86	94.79	93.81
X	154259566	Bonobo Mat	94.94	93.95	94.83	93.84
X	154259566	Gorilla Mat	94.31	92.91	94.16	92.76
Y	62460029	GRCh38	34.7	34.67	34.64	34.6
Y	62460029	HG002 Pat	99.94	99.94	99.94	99.94
Y	62460029	Chimp Pri	19.86	15.41	16.83	12.59
Y	62460029	Bonobo Pat	18.17	14.26	17.61	13.8
Y	62460029	Gorilla Pat	12.81	7.09	11.04	6.26

Supplementary Table III.18. Alignment coverage of Bornean orangutan primary in the orangutan pangenome.

Region	Length	Query	Aligned (pct)	Identical (pct)	Aligned 1:1 (pct)	Identical 1:1 (pct)
Autosomes	3028670501	B.Orang	91.2	90.92	90.48	90.2
Autosomes	6057341002	S.Orang	90.07	89.68	89.1	88.72
X	162586321	S.Orang Pri	94.1	93.8	94.1	93.8
Y	67827326	B.Orang Alt	0.91	0.89	0.91	0.89
Y	67827326	S.Orang Alt	1.14	1.13	0.16	0.15
Y	67827326	S.Orang Pri	41.72	39.14	41.55	38.98

Progressive Cactus genome alignment

We used Progressive Cactus[20] to construct two genome alignments: An 8-way primary alignment of the six T2T apes plus hg38 and hg31, as well as a 16-way diploid alignment of the same samples, but also including HG002. We used MashTree v1.4.6[20] with default arguments to compute guide trees for the alignment, restricting to autosomes in the case of the diploid genome assemblies. The resulting guide tree for the 8-way primary alignment was:

> (((GCA_028885655.2:0.0017500000000000016,GCA_028885625.2:0.0017299999999999999):0.014950000000000001,(GCA_029281585.2:0.00877,((GCA_029289425.2:0.0019999999999999983,GCA_028858775.2:0.0022900000000000004):0.0043300000000000005,(hs1:5.000000000000004E-4,hg38:5.000000000000004E-4):0.0059899999999999999):0.0014300000000000007):0.0073599999999999985):0.011345,GCA_028878055.2:0.011345000000000003);

and the Cactus (v2.7.1) commands used to construct the alignments were:

```
TOIL_SLURM_ARGS="--partition=long --time=8000" cactus ./js-8apes ./8-t2t-apes-2023v2.seqfile ./8-t2t-apes-2023v2.hal --batchSystem slurm --caching false --consCores 64 --configFile ./config-slurm.xml --logFile 8-t2t-apes-2023v2.hal.log --batchLogsDir batch-logs-8apes --coordinationDir /data/tmp
```

```
TOIL_SLURM_ARGS="--partition=long --time=8000" cactus ./js-16apes ./16-t2t-apes-2023v2.seqfile ./16-t2t-apes-2023v2.hal --batchSystem slurm --caching false --consCores 64 --configFile ./config-slurm.xml --maxOutgroups 3 --chromInfo 16-t2t-apes-2023v2.chroms --logFile 16-t2t-apes-2023v2.hal.log --batchLogsDir batch-logs-16apes --coordinationDir /data/tmp
```

Note that the input, output including UCSC Genome Browser track hubs, and all steps to reproduce Progressive Cactus alignments can be found at https://cglgenomics.ucsc.edu/february-2024-t2t-apes/.

We extracted an MAF version of each alignment referenced on each species and computed the coverage, all as described above for the pangenome graphs. The coverage on T2T-CHM13/hs1 is described in these tables.

Supplementary Table III.19. Alignment coverage of T2T-CHM13 (hs1) in the 8-way primary Progressive Cactus alignment.

Region	Length	Query	Aligned (pct)	Identical (pct)	Aligned 1:1 (pct)	Identical 1:1 (pct)
Autosomes	2900555906	GRCh38	93.81	93.67	87.08	86.96
Autosomes	2900555906	Chimp	91.47	90.22	86.1	84.95
Autosomes	2900555906	Bonobo	91.48	90.22	85.98	84.83
Autosomes	2900555906	Gorilla	90.89	89.33	85.44	83.99
Autosomes	2900555906	B.Orang	88.49	85.51	83.41	80.62
Autosomes	2900555906	S.Orang	88.47	85.49	83.4	80.61
Autosomes	2900555906	Siamang	84.73	81.36	79.91	76.78
X	154259566	GRCh38	97.66	97.56	88.9	88.82
X	154259566	Chimp Pri	95.41	94.37	86.48	85.57
X	154259566	Bonobo Pri	95.44	94.4	86.49	85.59
X	1542595	Gorilla Pri	94.53	93.08	85.62	84.34

	66					
X	1542595 66	B.Orang Pri	90.69	88.05	81.78	79.44
X	1542595 66	S.Orang Pri	90.59	87.95	81.64	79.31
X	1542595 66	Siamang Pri	84.09	81.22	75.71	73.21
Y	6246002 9	GRCh38	40.22	40.12	12.21	12.14
Y	6246002 9	Chimp Pri	33.93	32.97	8.01	7.75
Y	6246002 9	Bonobo Pri	34.12	33.16	8.3	8.02
Y	6246002 9	Gorilla Pri	31.33	30.28	6.64	6.46
Y	6246002 9	B.Orang Pri	26.96	25.35	6.1	5.71
Y	6246002 9	S.Orang Pri	26.99	25.37	6.09	5.7
Y	6246002 9	Siamang Pri	21.18	19.65	3.91	3.66

Supplementary Table III.20. Alignment coverage of T2T-CHM13 (hs1) in the 16-way diploid Progressive Cactus alignment.

Region	Length	Query	Aligned (pct)	Identical (pct)	Aligned 1:1 (pct)	Identical 1:1 (pct)
Autosom es	2900572 475	GRCh38	93.57	93.42	87.99	87.87
Autosom es	5801144 950	HG002	95.07	94.83	88.99	88.8
Autosom es	5801128 381	Chimp	91.07	89.84	85.02	83.89
Autosom es	5801111 812	Bonobo	91.08	89.84	84.92	83.79
Autosom es	5801128 381	Gorilla	90.62	89.08	84.5	83.08
Autosom es	5801144 950	B.Orang	88.39	85.42	82.55	79.79

Autosomes	5801144950	S.Orang	88.4	85.42	82.58	79.82
Autosomes	5801111812	Siamang	84.87	81.5	79.05	75.96
X	154259566	GRCh38	97.26	97.11	86.19	86.13
X	154259566	HG002 Mat	96.99	96.91	85.39	85.34
X	154259566	HG002 Pat	7.48	6.96	0.12	0.09
X	154259566	Chimp Alt	1.67	1.42	0.24	0.19
X	154259566	Chimp Pri	91.66	90.5	80.71	79.88
X	154259566	Bonobo Mat	91.03	90	80.31	79.5
X	154259566	Bonobo Pat	4.93	4.39	0.38	0.32
X	154259566	Gorilla Mat	90.21	88.78	79.55	78.39
X	154259566	Gorilla Pat	4.36	3.9	0.32	0.28
X	154259566	B.Orang Alt	1.47	1.24	0.21	0.17
X	154259566	B.Orang Pri	86.95	84.36	75.91	73.76
X	154259566	S.Orang Alt	1.49	1.26	0.21	0.17
X	154259566	S.Orang Pri	86.14	83.54	75.2	73.07
X	154259566	Siamang Alt	1.81	1.6	0.68	0.64
X	154259566	Siamang Pri	79.55	76.76	69.73	67.46
Y	62460029	GRCh38	38.96	38.67	8.9	8.87
Y	62460029	HG002 Mat	20.66	19.39	0.3	0.26
Y	62460029	HG002 Pat	58.41	58.06	27.15	27.02

Y	62460029	Chimp Alt	5.25	4.82	0.19	0.16
Y	62460029	Chimp Pri	27.95	27.1	4.4	4.32
Y	62460029	Bonobo Mat	18.92	17.66	0.38	0.33
Y	62460029	Bonobo Pat	21.98	21.24	4.53	4.43
Y	62460029	Gorilla Mat	18.03	16.83	0.29	0.25
Y	62460029	Gorilla Pat	20.46	19.75	4.04	3.93
Y	62460029	B.Orang Alt	4.22	3.85	0.08	0.07
Y	62460029	B.Orang Pri	24.41	22.92	3.51	3.28
Y	62460029	S.Orang Alt	4.23	3.86	0.1	0.08
Y	62460029	S.Orang Pri	24.49	23.01	3.55	3.32
Y	62460029	Siamang Alt	3.64	3.31	0.05	0.04
Y	62460029	Siamang Pri	19.52	18.1	2.2	2.05

Annotation of the human-primate ancestral allele

We used the parsimony-like method used by the 1000 Genomes Project and Ensembl[21,22] with the following tree for this annotation, ((Gorilla,((Bonobo,Chimp)b,Human)a)c), where a, b, and c refer to the inferred ancestral sequences. Instead of using the EPO pipeline used by Ensembl, we used an 8-way alignment produced by Progressive Cactus available here https://cgl.gi.ucsc.edu/data/cactus/t2t-apes/8-t2t-apes-2023v2/. The Ensembl human-primate ancestor based in GRCh38 was downloaded from https://ftp.ensembl.org/pub/release-112/fasta/ancestral_alleles/.

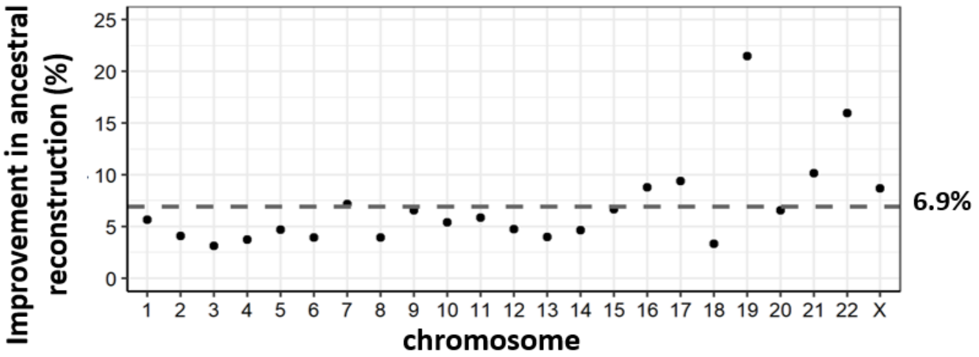

Supplementary Figure III.13. Improvement in the ancestral allele inference by Cactus alignment of the T2T ape genomes over the Ensembl/EPO alignment.

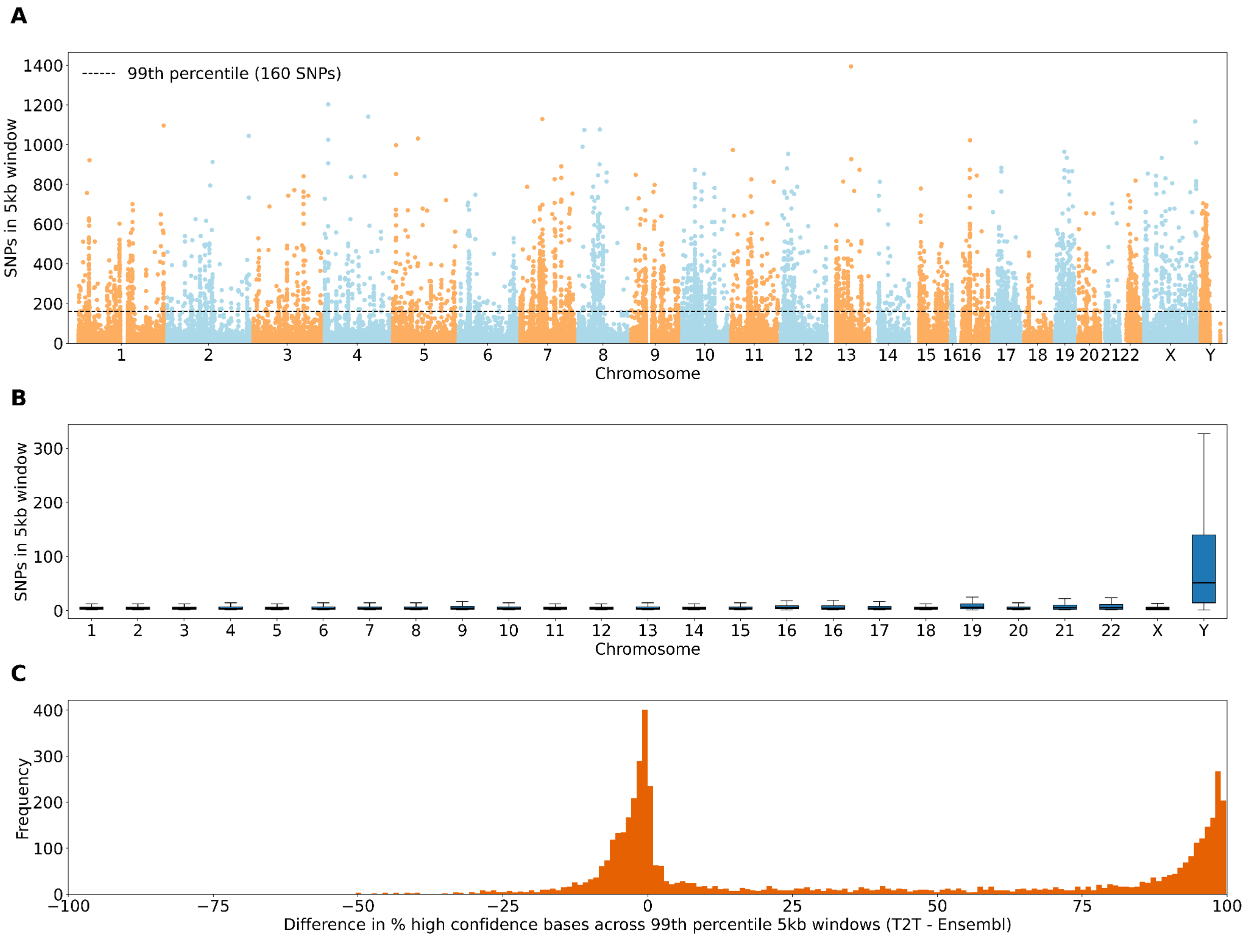

Supplementary Figure III.14. Annotation of ancestral allele. (A) SNPs per 5 kbp window between the T2T annotation and the Ensembl annotation of the human–primate ancestor, both based on GRCh38. (B) Boxplots showing the distribution of SNPs in 5 kbp windows for autosomes. The box and the horizontal line indicate interquartile range and median while whiskers show 1.5-fold the interquartile range. (C) Difference in the percentage of high-confidence bases in 5 kbp windows (n=4840, shown in panel A exceeding the 99th percentile in SNPs, between the T2T annotation and Ensembl annotation. The ancestral base is recorded in high confidence if all three ancestral sequences agree on the base, otherwise it is low confidence indicating partial agreement.

IV. Sumatran vs. Bornean orangutan divergence

Contributing authors:

Robert S. Harris, Saswat Mohanty, and Kateryna D. Makova (Penn State University)

Methods

Pairwise alignments between the two orangutan genomes were extracted from the 8-way cactus[20] alignment (8-t2t-apes-2023v2.hal):

> hal file → hal2maf → maf_filter_to_species_set → mafDuplicateFilter → pairwise alignments

Sequence identity statistics were collected from these alignments:

> pairwise alignments → maf_to_plain_pairwise_identity → stats.

In particular, we computed sequence identity over alignable bases, as well as blast identity over alignment length (**Supplementary Tables IV.21-23**) over each chromosome as well as weighted by alignment length average across the autosomes:

> identity m/(m+mm) = 99.63%

and

> blast identity m/(m+mm+i+d) = 99.38%

where m = match, mm = mismatch, i = insertion, d = deletion.

Separately, we aligned the two orangutan genomes using LASTZ[23] and computed the same metrics (m, mm, i, d, identity, and blast identity) for these alignments. The following parameters were used:

> --notransition
>
> --scores=scoring/human_chimp.v2_scoring
>
> --allocate:traceback=1.5G

We computed sequence identity over alignable bases, as well as blast identity over alignment length over each chromosome (**Supplementary Table IV.22**) as well as weighted by alignment length average across the autosomes:

> identity m/(m+mm) =97.17%

and

> blast identity m/(m+mm+i+d) =96.34%

Summary of results

Here, for the first time, we sequenced and assembled the genome of Bornean orangutan, significantly (i.e., to the T2T level) improved the genome of Sumatran orangutan, and performed their detailed comparison. The two species diverged very recently, only approximately 0.5-2 mya[24-26] and are the most closely related species in our dataset. The sequence identity of alignable bases between the two orangutan genomes was 99.63% from 8-way alignments (considering autosomes only, **Supplementary Table IV.21**; sequence identities for the sex chromosomes are reported in Makova et al.[1] and 97.17% from 2-way alignments (again, considering autosomes only; both autosomal and sex chromosome values are reported in **Supplementary Table IV.22**).

V. Structurally divergent regions

Contributing authors:

Jiadong Lin, Junmin Han, Shilong Zhang, Yafei Mao

Methods

The all-vs-all alignment created to detect lineage-specific segmental duplications (SDs) was used to identify lineage-specific structurally divergent regions (SDRs) on each haplotype. For primate lineages (i.e., PTR, PPAPAB, GGO, PAB, PPY, SSY), sequences that were not aligned or aligned of identity <85% were considered as divergent regions specifically to each species on the leaf node of the ape phylogeny. The human lineage-specific (HSA) SDRs were those conserved between human haplotypes (i.e., CHM13 and HG002) but divergent from other primate haplotypes. For the *Pan* lineage, we first obtained regions on PTR that are not aligned to other species except for PAB. We then subtract regions that are specific to PTR from the regions obtained in the previous step. We used PAB and applied the same approach to obtain *Pongo* lineage-specific SDRs. The SDRs were further annotated with centromere, acrocentric, subterminal, secondary constriction (qh) regions, and euchromatin. To count the SDR bases by genomic content, we classified SDRs in the order of centromere, acrocentric, subterminal, sex chromosome, and others. For the others category, we further examined whether it overlapped qh region and those non-overlapped parts were classified as euchromatin. Note that for centromere and subterminal, we also considered bases that are not in the centromere or subterminal as euchromatin.

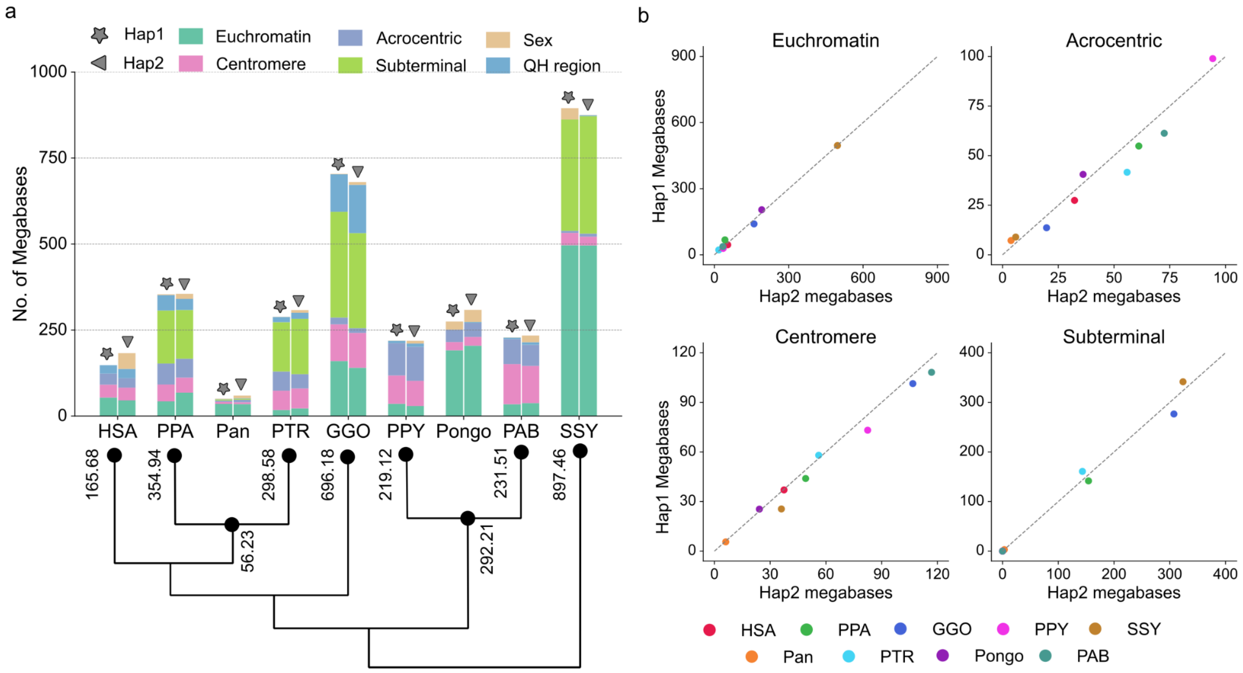

Supplementary Figure V.15. Lineage-specific structurally divergent regions (SDRs) on each haplotype. (a) The total megabases of SDRs detected on each haplotype. The average megabases of the two haplotypes were assigned to the phylogenetic tree. We used PTR and PAB to represent the ancestral node *Pan* and *Pongo*, respectively. (b) The comparison of SDR total megabases separated by euchromatin, acrocentric, centromere, and subterminal.

VI. Incomplete lineage sorting (ILS) and speciation times

Contributing authors:

Francesco Montinaro, Iker Rivas-González

Methods

Divergence time represents the average coalescent time between two sequences and can vary significantly across the genome. In contrast, speciation time refers to the minimum time at which two sequences can coalesce, reflecting when species become reproductively isolated.

We estimated ILS among different primates using TRAILS, which integrates a Hidden Markov Model of the ILS signal with a time discretization approach for the unbiased inference of demographic parameters, allowing the posterior decoding of both topology and coalescent times. In our analysis we considered any genomic region in ILS status when the reconstructed phylogeny as different from ((A,B)),C,D).

We performed the ILS estimation on the following four species (ABCD) phylogenies (**Supplementary Table VI.26**), selected in order to have at least one estimate for each branch across the analyzed species:

- *Homo sapiens; Pan troglodytes; Gorilla gorilla, Pongo abelii* (HCGO)
- *Pan troglodytes; Pan paniscus; Homo sapiens; Pongo abelii* (CBHO)
- *Homo sapiens, Pan troglodytes, Pongo abelii, Symphalangus syndactylus* (HCOS)
- *Homo sapiens, Gorilla gorilla, Pongo abelii, Symphalangus syndactylus* (HGOS)
- *Pongo Abelii, Pongo Pygmaeus, Homo sapiens, Symphalangus syndactylus* (OOHS)

We also harnessed msmc2[27] to infer the population size of single species; we started from the diploid multi-alignment, removed duplicates and created the multihet-step msmc2 input file using the MsmcOutput flag. Next, we estimated the between haplotype coalescence rates across time using default parameters and converted the inferred metrics to effective population size as in Schiffels[27] and Wang et al.[28] We used the same mutation rate of TRAILS analysis and the following generation times (extracted from Rivas-González et al. 2023, Table S1[29]):

1. Chimp: 24 years
2. Bonobo: 24 years
3. Human: 25 years
4. Gorilla: 19 years
5. Borneo and Sumatra Orangutan: 25 years
6. Gibbon: 15 years

Summary of results

We started from the haploid or diploid cactus alignment of the eight primate species and extracted the four relevant species for each phylogeny. We then merged syntenic regions separated by less than 200 bp and retained only blocks longer than 2 kbp. Every analysis was repeated three times, alternatively including the maternal/primary or paternal/alternative haplotype of the analyzed individuals. For Homo sapiens, we also included the t2t hs1 T2T-CHM13 haplotype as detailed in **Supplementary Table VI.27**. For all the analyses we used a mutation rate of 1.25×10^{-8} and the average generation time across the species for specific node:

1. Chimp-Bonobo: 24 years.
2. Homo-Chimp-Bonobo: 24.5 years.
3. Homo-Chimp-Gorilla: 21.75 years.
4. Homo-Chimp-Gorilla-Orangutan: 23.375 years.
5. Homo-Chimp-Gorilla-Orangutan-Gibbon: 19.2 years.
6. Orangutan Borneo-Orangutan Sumatra: 25 years.

For each of the 15 trees, we performed two optimization steps for the parameter and posterior probability estimations using the starting values as in Rivas-Gonzalez et al.[29] The first and second optimization steps were carried out by setting three discrete time intervals for both AB and ABC species.

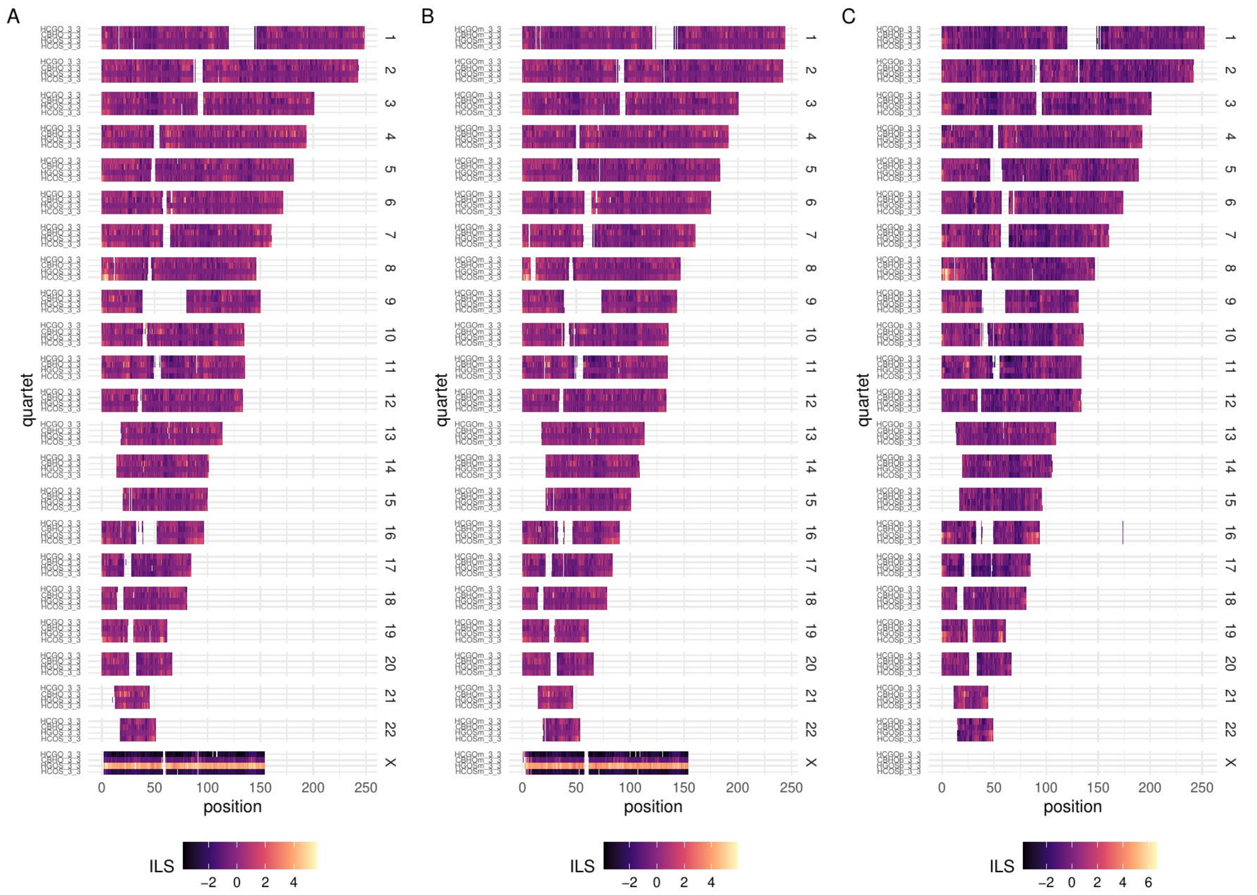

Supplementary Figure VI.16. Inference of ILS and demographic parameters. Inference of ILS proportion for 500 kbp windows among four phylogenies using chm/primary (A), maternal/primary (B), and paternal/alternative (C) haplotypes.

The parameter inference results across the five species' trees are summarized in **Supplementary Table VI.26**. The estimated time and population size parameters across the three replicates of each phylogeny are consistent. This is also confirmed when the correlation is assessed across genes for phylogenies using primary, maternal, or paternal haplotypes (**Supplementary Fig. VI.17**). In fact, for HCGO, considering the ILS proportion in windows that overlap with genes, a high correlation across replicates has been observed (R^2 HCGO vs. HCGOm = 0.88; R^2 HCGO vs. HCGOp = 0.89).

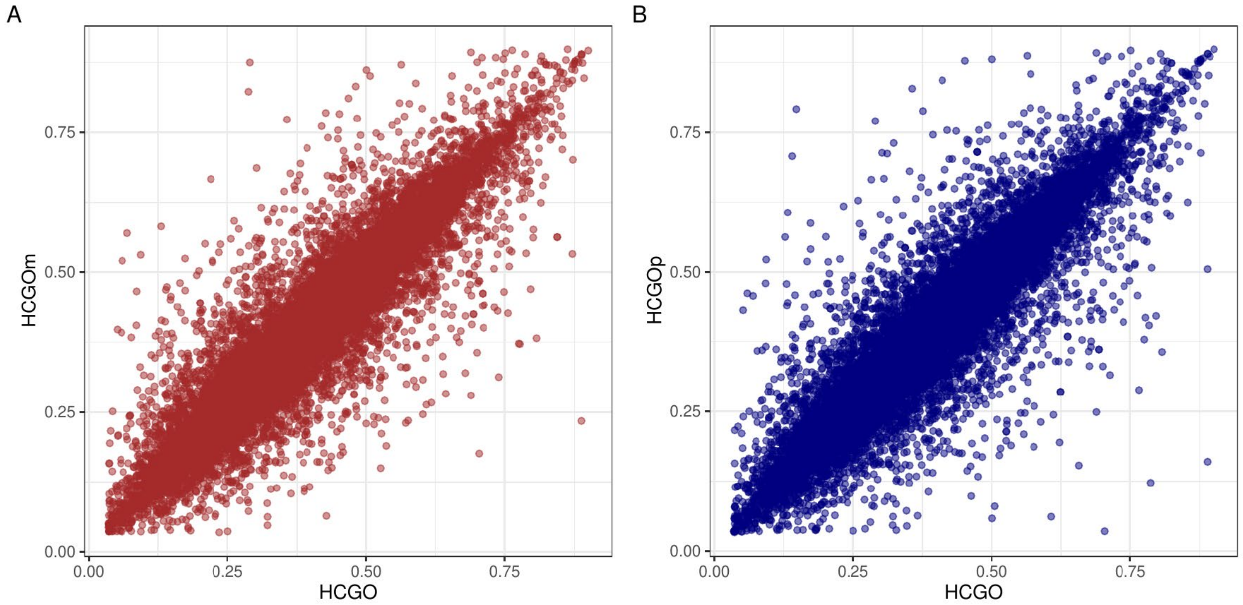

Supplementary Figure VI.17. Comparison of genic ILS proportion for the HCGO phylogeny across different replicates. (A) HCGO vs. HCGOm. (B) HCGO vs. HCGOp.

For the HCGO tree, we inferred the presence of 39.5% of autosomal genome and 24% of chromosome X in ILS, with an increase of approximately 7.5% compared to previous estimates[29]. Human and chimpanzee speciation time from the ancestral species has been estimated between 5.6 and 6.3 mya, respectively, in line with previous research. The speciation time from gorilla to the HGO ancestral species was estimated to be 10.6-10.9 mya, and the orangutan speciation to 18.2-19.6 mya. The population size of the ancestral population of human and chimpanzee (N=198,000) is larger than that estimated for human, chimpanzee, and gorilla (Ne=132,000), suggesting an increase of the population size between 6 and 12 mya. Moreover, we confirm the substantially reduced diversity for chromosome X for the HC ancestral population (Ne=76,700) but not for the population ancestral to HCG (N=115,600). This pattern can be explained by multiple population dynamics, such as strong selective sweeps on the X chromosome or, alternatively, reduced size for the female founder population.

For the CBHO phylogeny, we inferred approximately 5.8% of ILS, with an X chromosome estimate of 3.4%. The speciation time of the split is 1.58 mya, in line with previous research[30]. We estimated a speciation time between human and CB to approximately 6.8 mya and the O to CBHO 17.7 mya, confirming the robustness of the inference irrespectively of the phylogeny analyzed. The ancestral population size for the CB population (N=46,800) is about a third of that estimated for CBH (N= 115,600). We inferred 0.7% of ILS for the HCOS topology across the autosomal genome and 0.5% on the X chromosome. For HGOS, we inferred approximately 1.8% and 1.4% ILS across autosomal and X chromosomes, respectively.

Compared to previously reconstructed maps, the T2T assemblies allow us to compute ILS in previously inaccessible genomic regions such as that encompassing the HLA genes

(**Supplementary Fig. VI.18**), which shows relatively high levels of ILS for the HCGO phylogeny. In fact, many HLA genes show an ILS proportion higher than 0.6, with high concordance across replicates (**Supplementary Table VI.28**).

To further explore the locus, we analyzed the chr6:25Mbp-40Mbp region, both at window and base-pair posterior decoding level. When 100 kbp windows are analyzed, the region harboring HLA genes shows an increased ILS proportion between 30 and 33 Mbp, with a very similar pattern in all the considered phylogenies (**Supplementary Fig. VI.18**).

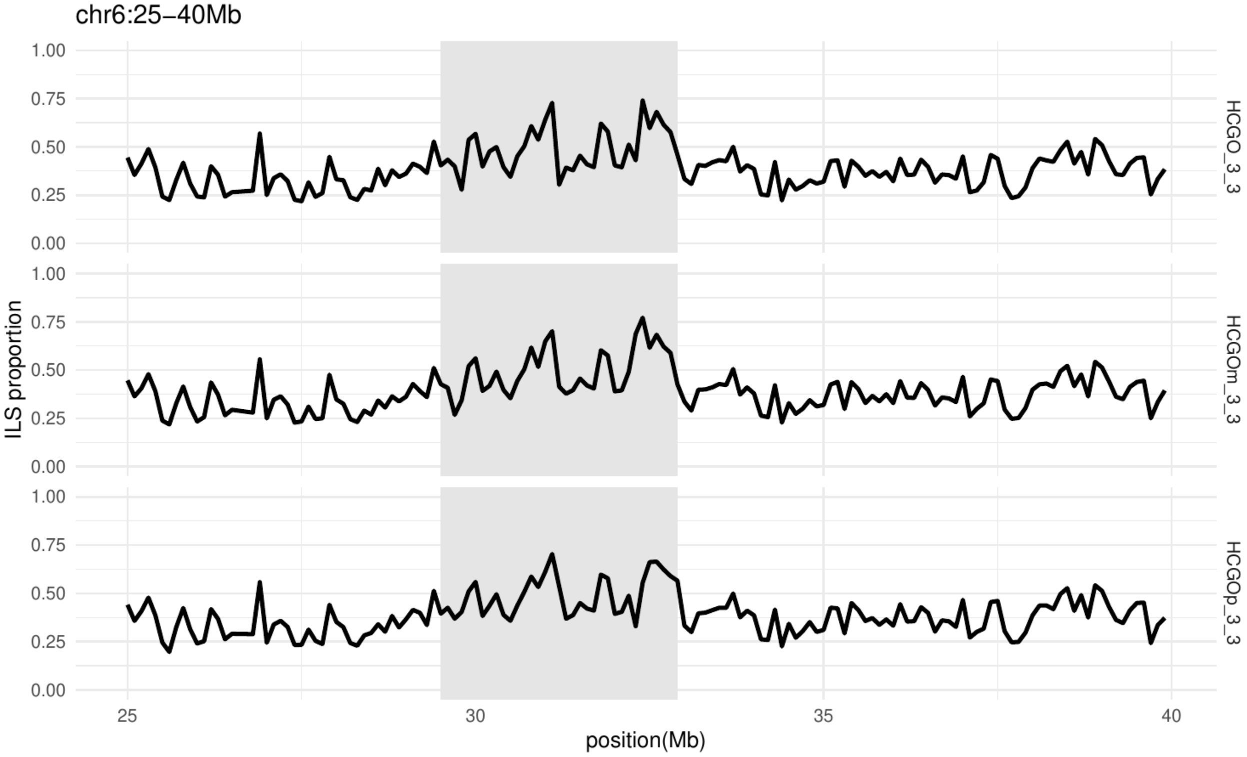

Supplementary Figure VI.18. ILS proportion in the chr6:25-40Mbp for HCGO phylogeny considering 100 kbp windows.

The posterior decoding gives per-base-pair posterior probabilities of observing the hidden states and, thus, we can build an ILS map at the highest resolution. **Supplementary Fig. VI.19** reveals that there are stretches of the genome that favor one of the two alternative topologies that do not follow the canonical species tree. For example, at around chr6:32.85Mbp, there is a region favoring the chimpanzee–gorilla topology, while chr6:29.30Mbp favors a human–gorilla topology.

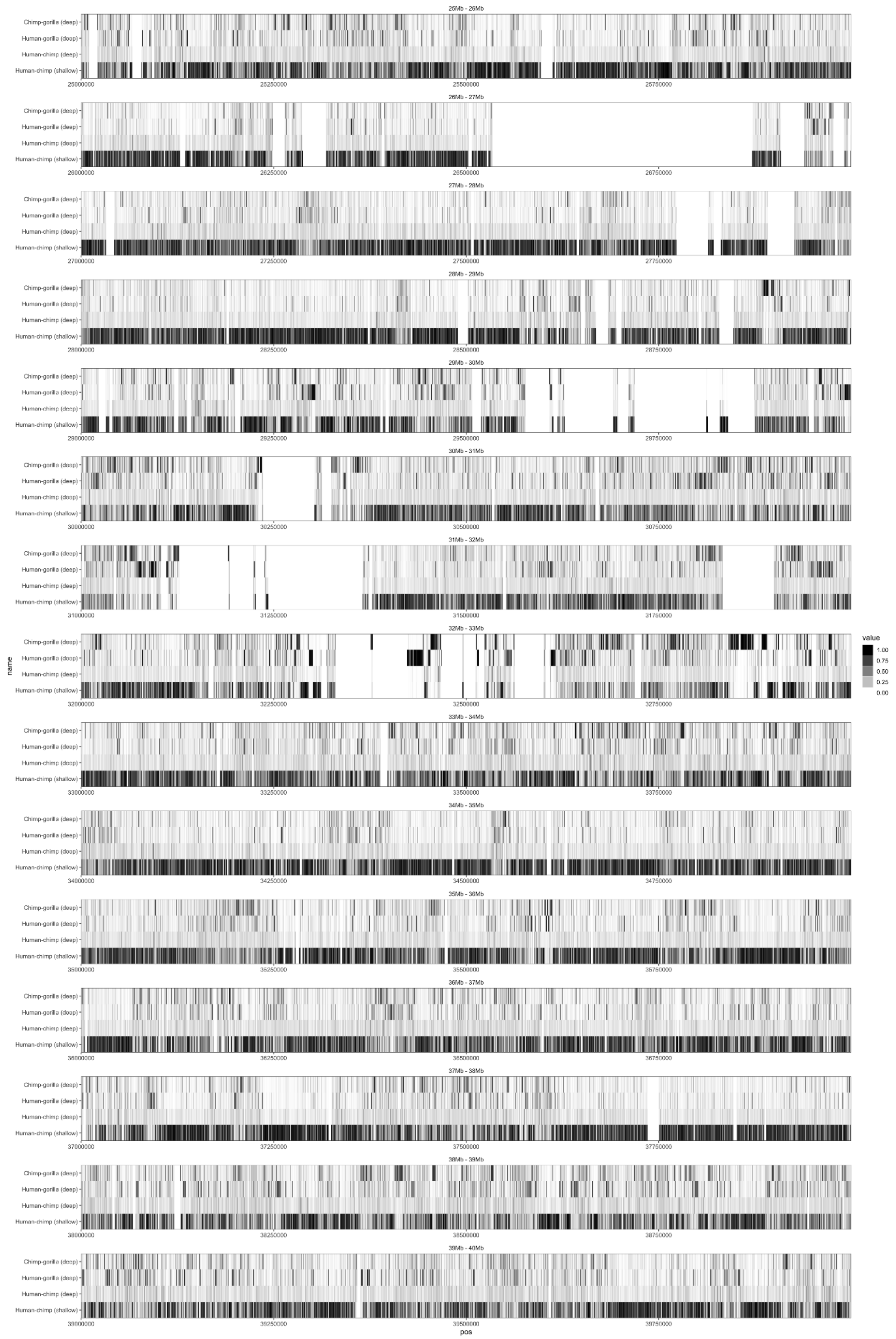

Supplementary Figure VI.19. ILS proportion in the chr6:25-40Mbp for HCGO phylogeny at base-pair level.

Moreover, we focused on the four HLA variable regions at a base-pair scale considering the tree topology and the first coalescent event (**Supplementary Fig. VI.20**). Three of the variable regions (I, II, and III) show elevated levels of ILS, with the only exception of region IV, confirming their high variability. For region I, 88% of the analyzed sequence was compatible with a topology in which human and gorilla are sister groups, and the first coalescent is shallow, consistent with the reconstructed phylogeny in **Extended Fig. 3c** and **Supplementary Fig. XI.42**, suggesting a possible balancing selection event. In region II, 36% of the sequence is consistent with an HC topology, with the remaining regions equally distributed. In the remaining two regions, we observed 55% (region III) and 71% (region IV) of the region topology consistent with HCGO.

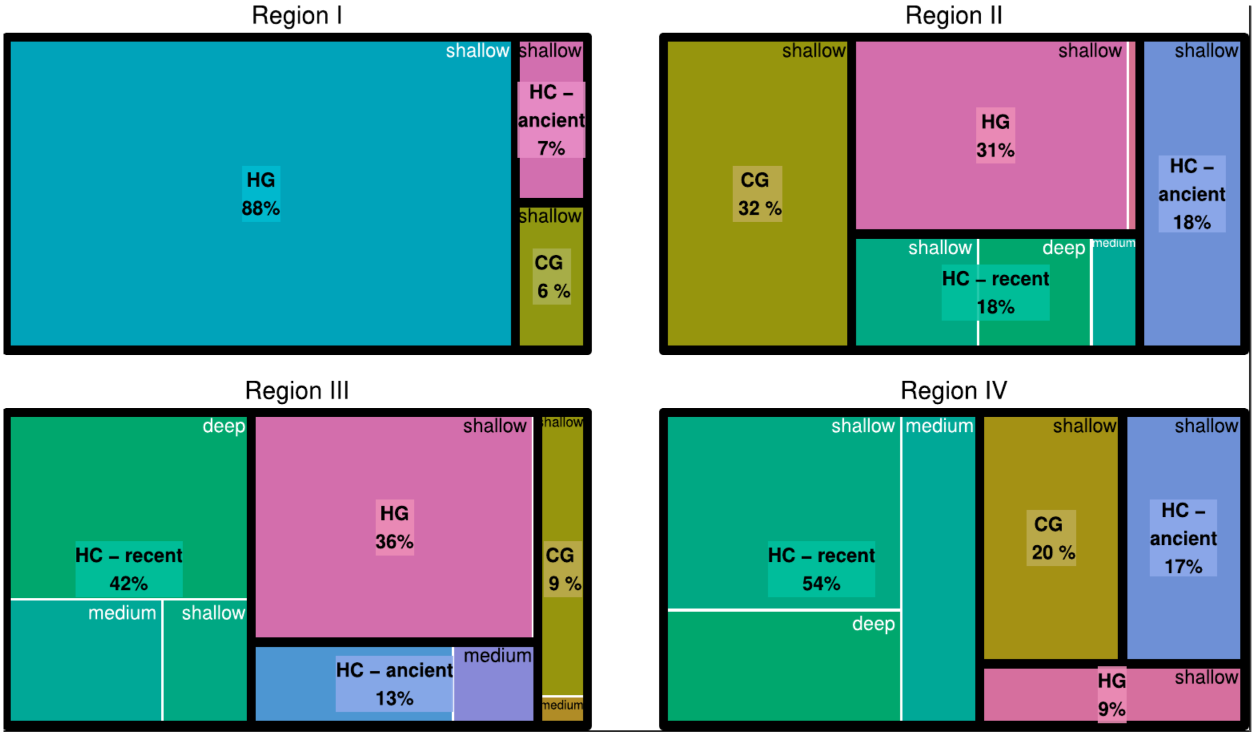

Supplementary Figure VI.20. Treemaps of the topology inferred for the four HLA variable regions. The topology is indicated in the middle of each rectangle. We show four possible topologies: human-chimp (HG recent) with shallow first coalescent, human-chimp with deep first coalescent (HG ancient), chimp-gorilla (CG) and human-gorilla (HG). Labels on the right corner refer to the first coalescent deepness.

Furthermore, we explored the role of repetitive regions in increased ILS estimation in HCGO, when compared with previous estimates[29]. In doing so, we iteratively excluded 1000 bp windows overlapping with the T2T-CHM13 repeat track, considering different repeat sizes. When all the repeats-containing windows are excluded, the inferred ILS is 37.7%, which is consistent with those estimated in Rivas-Gonzales et al. 2023[29] using the alignments from Shao et al. 2023[26].

When we excluded repeats longer than 100 bp and 1000 bp, the inferred ILS proportion is 37.6% and 38.5%, respectively, increasing to 39.4 when repeats longer than 10^5 bp are removed (**Supplementary Fig. VI.21**). These results suggest that repetitive regions account for ~2% of the ILS increase, consistent with selectively neutrally evolving DNA.

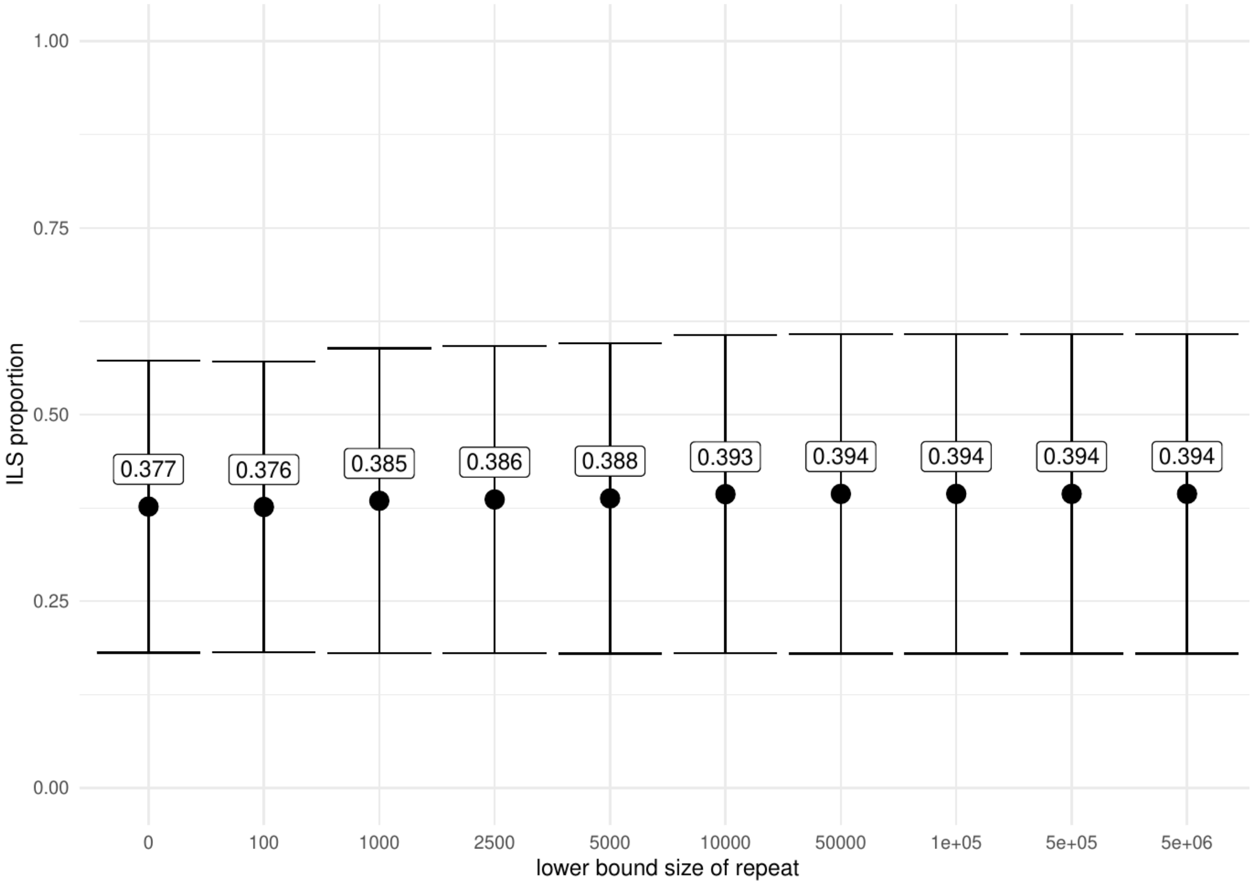

Supplementary Figure VI.21. Estimates of ILS proportion (x-axis) in HCGO when excluding 1000 bp windows overlapping with repetitive regions of different sizes (y-axis). Numbers in the y-axis refer to the lower bound of the excluded repeats. Confidence intervals refer to standard deviations inferred across 1000 bp windows.

To explore the association between inferred ILS in HCGO and adaptation we intersected ILS proportion and selective sweeps at the gene level for bonobo, chimpanzee, and gorilla (**Supplementary Fig. VI.22**). We observed that genes putatively under selection have significantly lower ILS proportion than those with no evidence of selective pressure (one-sided Wilcoxon rank sum test, p-value=5.8^{-12}). This signal is driven by the putatively selected genes identified by SweepFinder2, which is designed to detect predominantly distorted Site Frequency Spectrum pattern due to hard sweeps. In fact, the ILS proportion for selected genes with saltiLASSI, which is tuned to detect both hard and partial sweeps is significantly larger than

those with no evidence of selection (p-value = 6.04^{-6}). These results confirm that ILS patterns are directly affected by selective trajectories.

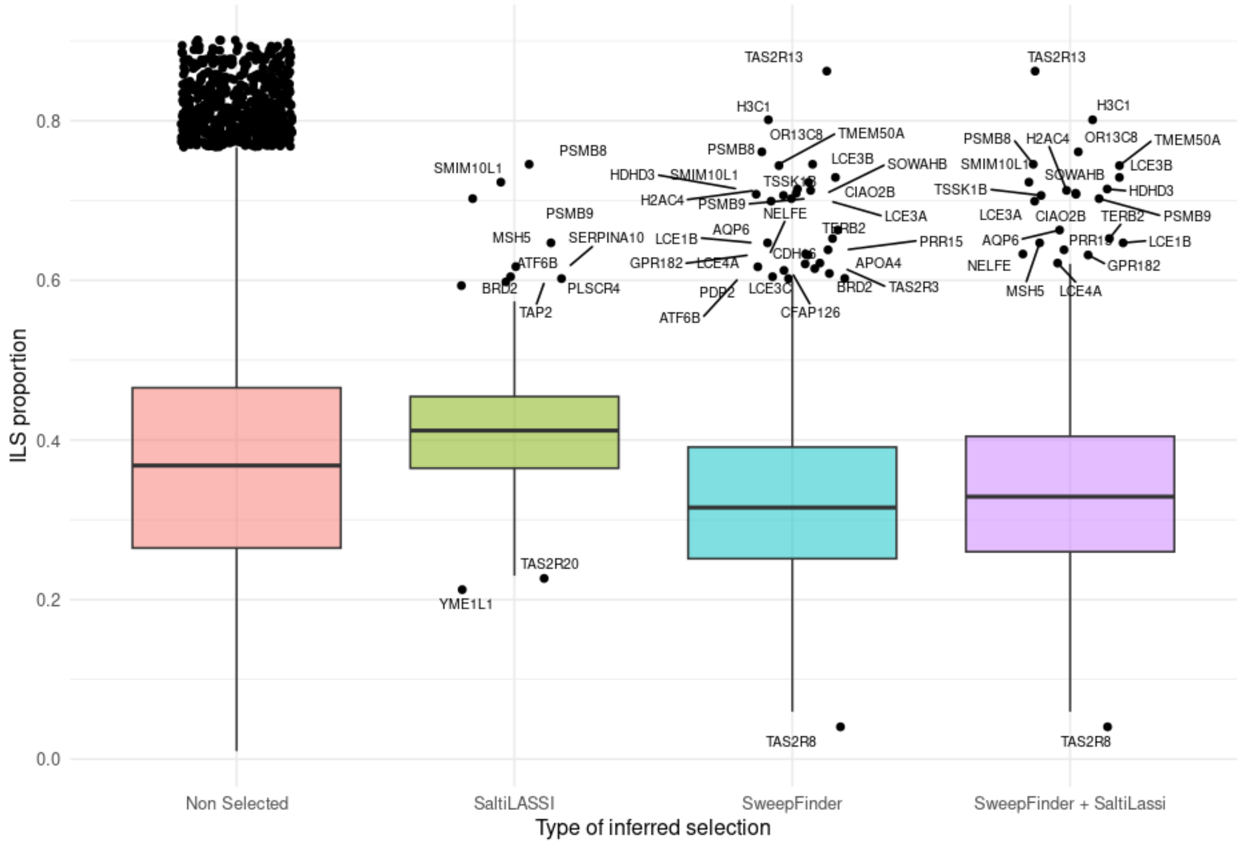

Supplementary Figure VI.22. Proportion and inferred selective pressure. We intersected ILS proportion and selection scans results at gene level. The box and the horizontal line indicate interquartile range and median while whiskers show 1.5-fold the interquartile range. Names showing an excess or defect of ILS (exceeding 1.5-fold the interquartile range) are annotated.

Changes in the effective population sizes through time are shown in **Supplementary Fig. VI.23.** We confirm a general decline of *Pan* genus population size starting approximately 5 mya, and a subsequent increase between 1 and 2 mya. For gorilla, we inferred a relatively low constant sample size, possibly reflecting an inbreeding history[2]. For orangutans, we observe a similar trend until 1 mya, confirming the estimates obtained with TRAILS, followed by a steep decline in the Bornean orangutan. Notably, the effective population size for the Sumatran orangutan is the historically highest before 100,000-200,000 years ago, as reflected by the broad geographic distribution of Pleistocene fossil remains in mainland Asia.

When species-specific mutation rates were used[29,31] a reduced population size for *Pongo abelii* and an increased one for *symphalangus syndactylus* were observed.

A.

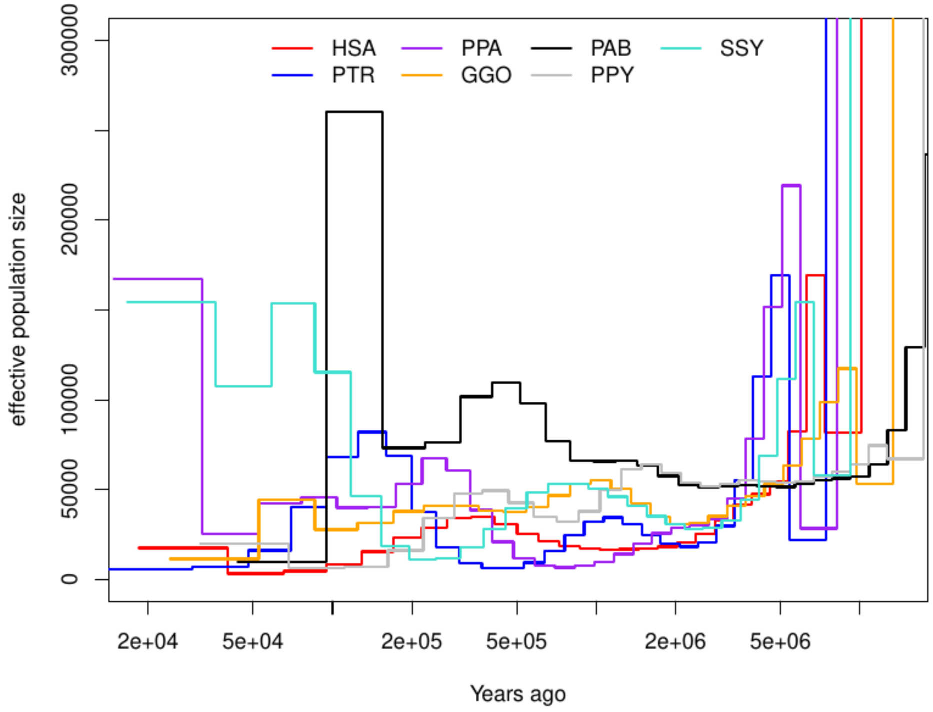

B.

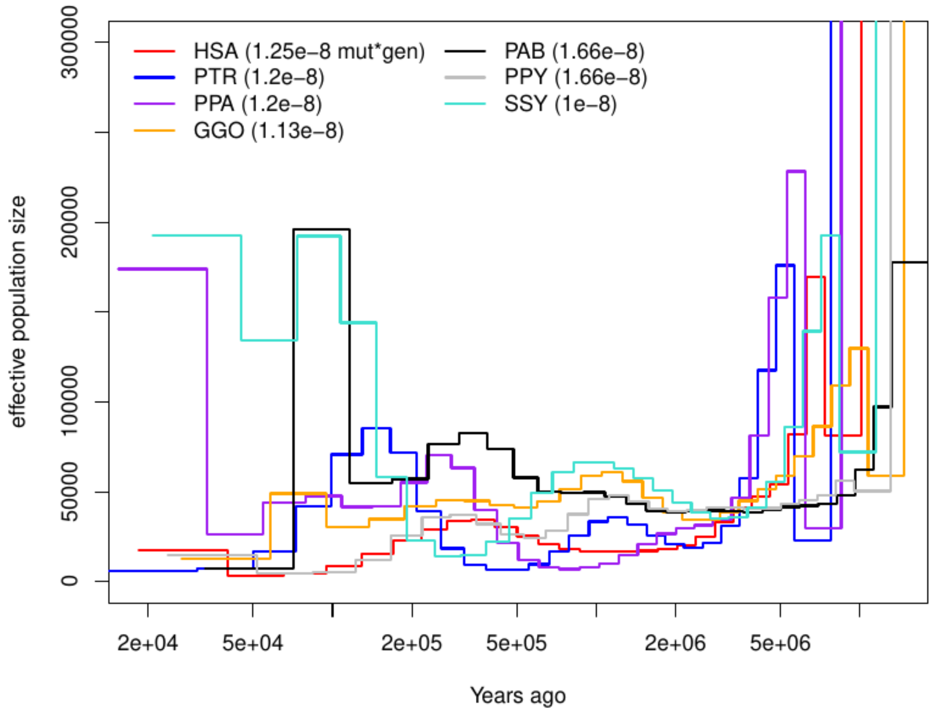

Supplementary Figure VI.23. Effective population size trajectories through time as inferred by msmc2. (A) Constant mutation rate 1.25 x 10-8 bp*gen. (B) Species-specific mutation rates were used, as indicated in legend.

VII. Selection analyses within NHP lineages

Contributing authors:

Abigail N. Sequeira, Qiuhui Li, Arjun Biddanda, Rajiv McCoy, Michael Schatz, Michael Tassia, Zachary A. Szpiech, Christian D. Huber, Kateryna D. Makova

Methods

Read mapping and variant calling

We performed read alignment and variant calling using 129 samples[2-5] described in the T2T ape sex chromosome study[1]. For the T2T references, we generated karyotype-specific references following a published masking method[10] to improve variant representation in sex chromosome pseudoautosomal regions. Reads from XX and XY samples were aligned to their respective masked T2T reference genomes. Variant identification followed the T2T-chrXY ape paper method using GATK v4.4.0.0 HaplotypeCaller[32] for initial calling, GenotypeGVCFs for joint genotyping, and applying the QC filters to SNPs ("QD < 2.0 || QUAL < 30.0 || SOR > 3.0 || FS > 60.0 || MQ < 40.0") and indels ("QD < 2.0 || QUAL < 30.0 || FS > 200.0"). To further improve variant calling and genotyping accuracy, we restricted our analysis to the 'accessible' genomic regions. Following an established protocol (https://www.illumina.com/science/genomics-research/articles/identifying-genomic-regions-with-high-quality-single-nucleotide-.html), we performed a short-read accessibility mask for the T2T reference genomes. This mask incorporates three alignment metrics: high-quality bases with normalized coverage within 25% of the median autosomal coverage, positions with a mapping quality score of 50 or higher, and regions where ≥90% of base calls have a Q20 score or above.

Haplotype phasing and curation

Haplotype phasing was performed across all primate T2T autosomal genomes using BEAGLE v4.0[33] (*impute=false nthreads=8 burnin=4 iterations=12 seed=42*). In all cases the effective population size (*Ne*) and error parameters were estimated on a per-taxa and per-chromosome level prior to phasing. No reference panel was used during the phasing process (https://github.com/aabiddanda/haplotype-phasing). We filtered the phased VCFs for bi-allelic sites that fell within high-confidence regions, resulting in the removal of less than 1% of called SNPs for each species (**Supplementary Table VII.29**). We ran two different selective sweep detection methods, SweepFinder2[34] and saltiLASSI[35], for 10 great ape taxa: bonobo, Bornean orangutan, central chimpanzee, eastern chimpanzee, eastern lowland gorilla, mountain gorilla, Nigerian chimpanzee, Sumatran orangutan, western chimpanzee, and western lowland gorilla. We excluded the Cross River gorilla from the subsequent analyses as it had a sample size of one.

SweepFinder2

We generated non-reference allele frequency files for each population, excluding positions that were monomorphic or did not contain the non-reference allele from the filtered VCFs. We removed between 32,675 and 2,337,860 variants and retained between 4,467,345 and 24,868,785 bi-allelic SNPs across all 10 taxa (**Supplementary Table VII.30**). We used SweepFinder2 to first calculate the whole-genome site frequency spectrum (SFS) for each population using the whole-genome allele frequency file (*SweepFinder2 -f WG.freq.file SFS*). Then using this pre-calculated SFS, we ran SweepFinder2 to calculate a likelihood ratio score along a 1 kbp grid for each autosome for each population (*SweepFinder2 -lg 1000 Chr.freq.file SFS Out.file*).

saltiLASSI

We computed the saltiLASSI statistic with lassip (v1.2.0) for each taxon in three steps. The first step created window-based spectra files for each autosome following these parameters: a 201 SNP window size, a 100 SNP step size, a *–k* of five or ten to estimate the haplotype frequency spectrum (HFS), *–salti*, and *–unphased*. Because *–k* cannot be larger than the sample size (*n*), if *n* > 10, *k* was set to ten and if *n* < 10, *k* was set to five. The second step calculates the mean genome-wide HFS using the window-based spectra files generated for each autosome. The final step calculates the saltiLASSI likelihood ratio score for each autosome using the genome-wide HFS and the corresponding window-based spectra file. Together, the code was written as such:

```
lassip –vcf taxon.chr.vcf –pop taxon.IDs.txt –winsize 201 –winstep 100 -k 10 –calc-spec
–hapstats –salti –unphased –out taxon.chr.spectra

lassip --spectra taxon.chr*.spectra --avg-spec --out taxon.avg.spec.

lassip --spectra taxon.chr.spectra --salti --null-spec taxon.avg.spec --out
taxon.chr.final.out
```

Determining candidate sweeps

For the raw SweepFinder2 and saltiLASSI results, we filtered out positions for which a likelihood ratio was calculated but fell outside an accessibility mask generated for each reference species. Then, we downloaded gene annotation files for each reference from NCBI (GCF_029281585.2, GCF_029289425.2, GCF_028858775.2, GCF_028885655.2, GCF_028885625.2) and filtered the annotation files for the protein-coding biotype and for entries listed as "gene". For each gene, we added a 50 kbp flank to the start and end position to capture signals in potential regulatory sequence of each gene. Finally, we paired each position for which a likelihood ratio score was calculated with the corresponding gene and found the maximum likelihood score for each gene so that every gene has a single representative score.

To determine significant sweep regions from SweepFinder2, we normalized the gene-specific score distribution according to a procedure described in Souilmi et al[36]. We first log transformed the maximum likelihood statistic for each gene. Next, we binned these scores based on gene

length and performed a robust Z-transformation. Finally, we calculated the p-values for each Z-score, assuming a standard normal distribution. We estimated the false discovery rate (FDR) and corresponding q-values using the R package qvalue[37] (v2.34.0). Next, we defined sweep regions by combining genes that had a q-value of 0.1 or smaller and were within 1 Mbp of each other, to take into account that single sweep signals often span multiple genes. Lastly, we filtered out any sweep region that did not contain at least one gene with a q-value of ≤0.01.

We took an outlier approach for identifying significant sweeps with saltiLASSI. However, because the likelihood statistic in the MHC region for each taxon was substantially higher than other likelihood statistics and was potentially caused by strong balancing selection and not positive selection, we took the top 0.1 percentile of the likelihood statistic before and after filtering out the MHC region. Finally, we combined the two to have one single dataset for each species. We again concatenated genes that were within 1 Mbp of each other to determine sweep regions.

Fst

Fst outlier peaks across the genome often reflect regions evolving under local adaptation. Therefore, we took the top 0.1% of Fst values between central and eastern chimpanzees (Fst = 0.09). We did not further investigate other taxa pairs because they were either fairly diverged (Fst > 0.21) or there were no clear Fst peaks across the genome-wide distribution. We assigned genes to the Fst peak regions and compared these regions to sweep regions identified by SweepFinder2 and saltiLASSI.

Gene enrichment

We performed a gene enrichment analysis with GOWINDA[38] (one-sided permutation test) on each taxon that had candidate sweeps called for SweepFinder2, saltiLASSI, and the top 0.1% Fst regions for central and eastern chimpanzees. GOWINDA requires four files: a whole-genome annotation as a .gtf, a file with the total number of SNPs, a file of candidate SNPs, and a gene set file. However, instead of providing SNP files, we provided the positions of the calculated likelihood ratio scores or the center of the Fst and saltiLASSI windows. Using the python scripts provided by the GOWINDA package, we converted each annotation file from a .gff to a .gtf. We downloaded a human gene set file from FuncAssociate 3.0[39] as recommended by GOWINDA. The gene set file contained the HGNC IDs for each gene associated with their respective GO category. For the total SNP file, we used the raw genome-wide positions (chromosome and position) from SweepFinder2, saltiLASSI, and Fst calculations. The candidate SNP files consisted of the positions for which the likelihood ratio or Fst window midpoint fell within the candidate sweep regions or was a top 0.1% of Fst value. We ran GOWINDA using *–mode gene* and *–gene-definition updownstream50000*. The full command was as follows: *java -Xmx4g Gowinda-1.12.jar –snp-file sel.scan.pos.txt –candidate-snp-file candidate.SNP.txt –gene-set-file funcassociate.go.txt –annotation-file annotation.gtf –simulations 100000 –min-significance 1 –mode gene –min-genes 1 –gene-defintion updownstream50000 –threads 8 –output-file*

out.GO.enrichment.txt. We considered GO terms that had an FDR of ≤0.1 to be significantly enriched.

Summary of results

To identify regions harboring population genetic signatures of adaptation in 10 great ape taxa, we used two complementary methods. SweepFinder2 scans for regions exhibiting distorted allele frequency patterns characteristic of a fixed hard sweep (i.e., an excess of low- and high-frequency alleles), whereas saltiLASSI scans for distorted haplotype frequency patterns indicative of a soft or partial sweep. Across all taxa, we identified 143 and 86 candidate regions using SweepFinder2 and saltiLASSI, respectively. Only two candidate regions overlapped between the two methods (**Supplementary Table VII.32**), consistent with their sensitivities for detecting distinct modes of positive selection.

We next performed a gene-set enrichment analysis for GO terms in the sweep regions. We found significant enrichment for genes involved in pathways related to diet (sensory perception for bitter taste, lipid metabolism, and iron transport), immune function (antigen/peptide processing, MHC-I binding), cellular activity, and oxidoreductase activity in bonobos, central and eastern chimpanzees, and western lowland gorillas.

Selection signatures were strongest in the MHC region, a gene-rich locus previously described as a target of strong selection, especially balancing selection[40]. Earlier studies, however, suggested that an ancient MHC-I sweep in the bonobo and central-eastern chimpanzee ancestor results from an adaptation to simian immunodeficiency virus (SIV)-like retroviruses[41-43]. We found evidence of long-term balancing selection on MHC in multiple great ape lineages, including central and eastern chimpanzees, as well as at least two regions in MHC consistent with positive selection in bonobos and western chimpanzees.

Genes encoding bitter taste receptors in primates have been well documented to have undergone species-specific adaptation, especially in chimpanzees[44,45] and gorillas[46]. In agreement with this, we detected significant enrichment in selection signals for such genes in bonobos (*TAS2R3*, *TAS2R4*, *TAS2R5*) and western lowland gorillas (*TAS2R14*, *TAS2R20*, *TAS2R50*), as well as identified a bitter taste receptor gene (*TAS2R42*) within a sweep region in eastern chimpanzees.

To assess the impact of selective sweeps on genome-wide genetic differentiation, we examined F_{ST} values within and outside of SweepFinder2 sweep regions in recently diverged eastern and central chimpanzee subspecies. Notably, sweep regions in both subspecies exhibited significantly higher differentiation (F_{ST} = 0.21 and 0.15, two-sided Mann-Whitney $p < 0.001$) compared to the genome-wide average (F_{ST} = 0.09). No increased differentiation was observed within saltiLASSI sweep regions (two-sided Mann-Whitney $p > 0.05$). These findings suggest that hard selective sweeps play an important role in shaping genomic variation across eastern and central chimpanzees.

Selection scans

To call selective sweeps, we ran two sweep detection methods: SweepFinder2 and saltiLASSI. SweepFinder2 is an SFS-based tool that can detect hard sweeps, whereas saltiLASSI is based on an HFS and can detect hard, soft, and partial sweeps. For determining SweepFinder2 sweep regions, we used a q-value approach in which we concatenated genes with q-value < 0.1 that are within 1 Mbp of each other and called sweep regions significant if they contained at least one gene with a q-value of 0.01 or lower. SweepFinder2 detected sweeps in five out of 10 analyzed great ape taxa: bonobo, central, eastern, and western chimpanzee, and western lowland gorilla (30, 22, 62, 11, and 18 sweeps, respectively; **Supplementary Table VII.31**). Unsurprisingly, these taxa had larger sample sizes (n = 13, 18, 19, 11, and 27, respectively). We took an outlier approach for determining sweeps with the saltiLASSI method and identified 4-18 selective sweeps across all 10 taxa (**Supplementary Table VII.31**). We found minimal overlap between the sweep regions called by SweepFinder2 and saltiLASSI. However, we found one sweep region called by both SweepFinder2 and saltiLASSI on chromosome 10 in western lowland gorillas, one partially overlapping sweep called by both methods on chromosome 5 in bonobos, and one sweep region called on chromosome 3 in central and eastern chimpanzees (**Supplementary Table VII.32**). Each method also identified at least one sweep region occurring in between the various chimpanzee subspecies; SweepFinder2 classified two sweeps in central and eastern chimpanzees and saltiLASSI classified a sweep in central and Nigerian chimpanzees (**Supplementary Table VII.32**). We compared our sweep regions to those from previous literature by identifying at least one common candidate gene and corroborated sweep signals in bonobos[47], central, eastern, and western chimpanzees[47,48], and western lowland gorillas[46,47]. We identified 75 and 70 novel sweep regions via SweepFinder2 and saltiLASSI, respectively, as well as a total of 43 regions that were previously found in humans[12,47].

Beyond these two selection scan methods, we also assessed the top 0.1% of Fst values computed between central and eastern chimpanzees (**Supplementary Table VII.32**). There were no overlaps between the top 0.1% Fst values and saltiLASSI sweeps, but we found overlaps for five SweepFinder2 sweeps in eastern chimpanzees. These sweeps were located on chromosomes 1, 5, 10, and 18 (**Supplementary Table VII.32**). Furthermore, four out of five of the sweeps contained genes in significantly enriched pathways (see below for more detail).

The MHC, in particular, showcased a complex selection signature (**Supplementary Fig. VII.24-26**). The MHC is a gene-dense region that is subject to heavy selective pressure, especially for balancing selection[40]. Overall, we observed strong saltiLASSI peaks in either the MHC class I or II region in eight out of 10 taxa (central, eastern, and western chimpanzees, all three gorilla subspecies, bonobos, and Bornean orangutans), with six (central, eastern, and western chimpanzees, eastern and western lowland gorilla, and bonobos) being significant and ranking among the highest for peak strength (**Supplementary Fig. VII.24-26**). Moreover, we observed overlapping positive peaks of Tajima's D and nucleotide diversity for bonobos, central, eastern,

and western chimpanzees, western lowland gorillas, and Bornean orangutans (**Supplementary Fig. VII.24-26**). However, in eastern lowland gorillas we observed a negative Tajima's *D* and increased nucleotide diversity and in mountain gorillas we observed a positive Tajima's *D* and a decreased nucleotide diversity, giving conflicting signatures of selection (**Supplementary Fig. VII.25**). Given the results from Tajima's *D* and the nucleotide diversity estimates, it is likely that saltiLASSI was picking up balancing selection signals rather than positive selection in all taxa except for eastern lowland and mountain gorillas. In the case of SweepFinder2, we observed evidence for hard sweeps in bonobos and western chimpanzees (**Supplementary Fig. VII.24D and VII.26A**). Broadly, we find that there is a complex selection signature occurring in the MHC region across the various great ape species where clear balancing selection is observed in many of the great ape lineages and at least two instances consistent with positive selection, similar to previous findings[47,49].

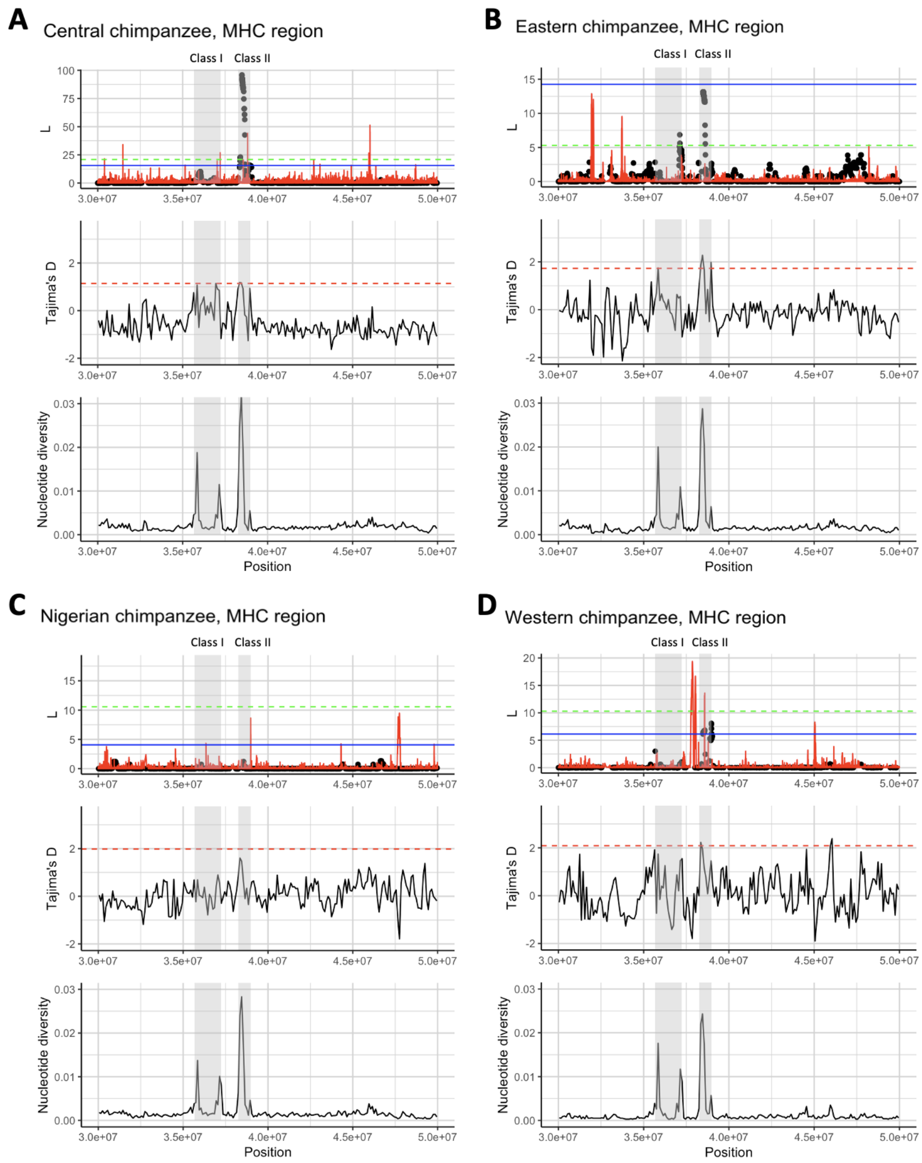

Supplementary Figure VII.24. Selection scans, Tajima's *D*, and nucleotide diversity for the chimpanzee MHC region. (A-D) Gray boxes represent the locations of MHC class I and II genes. Top panel: SweepFinder2 (red solid line) and saltiLASSI (black dots) likelihood ratio scores are plotted along genomic position. SweepFinder2 results for eastern, Nigerian, and western chimpanzees were scaled down by a factor of 10. Central chimpanzee SweepFinder2 results were not scaled down. The blue solid line marks the top 0.1% of saltiLASSI results and the green dotted line marks the top 0.1% of SweepFinder2 peaks, scaled down for eastern, Nigerian, and western chimpanzees. saltiLASSI peaks that are above the blue line were counted as candidate sweeps. Middle panel: Tajima's *D* calculated across 100 kbp windows. The red dotted line represents the top 0.1% of values. Bottom panel: Nucleotide diversity calculated across 100 kbp windows.

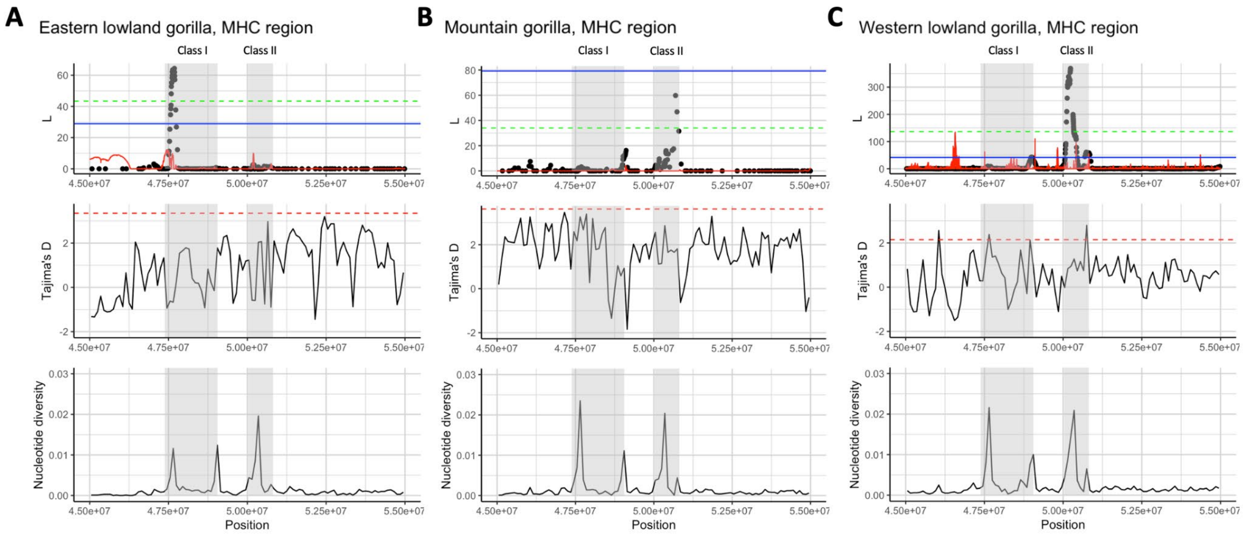

Supplementary Figure VII.25. Selection scans, Tajima's *D*, and nucleotide diversity for the gorilla MHC region. (A-C) Gray boxes represent the locations of MHC class I and II genes. Top panel: SweepFinder2 (red solid line) and saltiLASSI (black dots) likelihood ratio scores are plotted along genomic position. SweepFinder2 results for eastern lowland gorilla and mountain gorilla were scaled down by a factor of 100. Western lowland gorilla SweepFinder2 results were not scaled down. The blue solid line marks the top 0.1% of saltiLASSI results and the green dotted line marks the top 0.1% of SweepFinder2 peaks, scaled down for eastern lowland gorilla and mountain gorilla. saltiLASSI peaks that are above the blue line were counted as candidate sweeps. Middle panel: Tajima's *D* calculated across 100 kbp windows. The red dotted line represents the top 0.1% of values. Bottom panel: Nucleotide diversity calculated across 100 kbp windows.

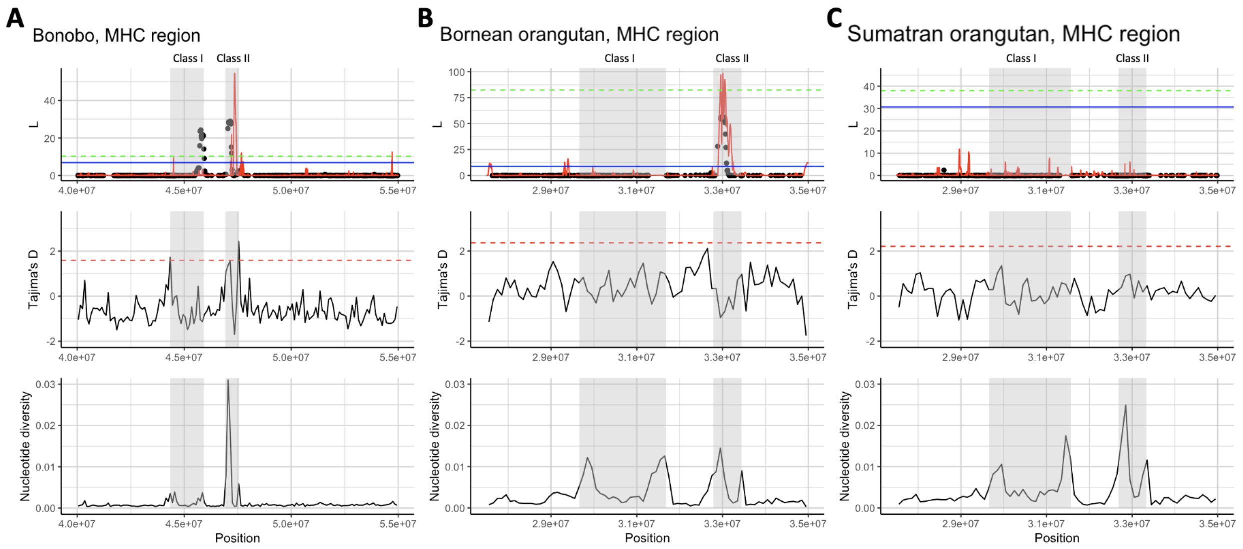

Supplementary Figure VII.26. Selection scans, Tajima's *D*, and nucleotide diversity for the bonobo and orangutan MHC regions. (A-C) Gray boxes represent the locations of MHC class I and II genes. Top panel: SweepFinder2 (red solid line) and saltiLASSI (black dots) likelihood ratio scores are plotted along genomic position. SweepFinder2 results for bonobo, Bornean and Sumatran orangutans were scaled down by a factor of 10. The blue solid line marks the top 0.1% of saltiLASSI results and the green dotted line marks the top 0.1% of SweepFinder2 scaled peaks. saltiLASSI peaks that are above the blue line were counted as candidate sweeps. Middle panel: Tajima's *D* calculated across 100 kbp windows. The red dotted line represents the top 0.1% of values. Bottom panel: Nucleotide diversity calculated across 100 kbp windows.

Following the selection scans, we ran a gene set enrichment analysis via GOWINDA to test if genes with certain GO terms are enriched in the sweep regions. We considered any GO term that had an FDR of 0.1 or smaller to be significantly enriched. Out of the 10 taxa, only bonobos, central and eastern chimpanzees, and western lowland gorillas had significantly enriched GO terms. Sweep regions detected by SweepFinder2 were enriched in bonobos, eastern chimpanzees, and western lowland gorillas while regions identified by saltiLASSI were found to be enriched in central chimpanzees (**Supplementary Table VII.33**). Of the top 0.1% Fst peaks between central and eastern chimpanzees, we find enrichment for genes related to the regulation of epidermal cell division (**Supplementary Table VII.33**). Of note, the two genes in this gene set both fall in the same sweep region detected by SweepFinder2 in eastern chimpanzees, suggesting species-specific differentiation in these two genes between central and eastern chimpanzees. Across all three selection tests, enrichment was found for genes involved in diet (sensory perception for bitter taste, lipid metabolism, and iron transport), immune function (antigen/peptide processing, MHC class I binding), cellular activity, and oxidoreductase activity (**Supplementary Table VII.33**).

Diet-related function

Among the enriched GO terms, we identified several pathways related to diet. Pathways involving lipid metabolism, oxidoreductase activity, and iron transport were found for genes within eastern chimpanzee sweeps (**Supplementary Table VII.33**). Chimpanzees are highly frugivorous omnivores[50-52]. Moreover, several studies, particularly in eastern chimpanzees, have reported them to engage in geophagy (intentional eating of soils) of termite mounds and clay-infused water[53-56]. It is hypothesized that these are adaptive behaviors to either provide protection against plant toxins and parasites or to supplement essential elements such as iron[57]. While these behaviors are not specific to eastern chimpanzees—indeed geophagy is found across a wide variety of taxa among and outside of primates[57,58]—the iron transport pathway was only significantly enriched in eastern chimpanzees, pointing to recent adaptation to dietary iron availability in this subspecies.

The oxidoreductase pathway encompasses a wide array of enzymes that are crucial for maintaining cellular homeostasis, energy production, biosynthesis, detoxification, and signaling. Notably, all but one of the 12 candidate genes that are in the oxidoreductase activity pathway are strongly connected to diet or diet-related disease. These 11 genes are involved with androgen metabolism, aldehyde oxidation, breaking down fatty acid chains, and vitamin metabolism. Furthermore, these have been associated with nonalcoholic fatty liver disease[59,60] and obesity[61,62].

Genes encoding for bitter taste receptors (TAS2Rs or T2Rs) have an interesting evolutionary history. In primates, T2Rs have been well documented to have undergone species-specific modes of selection, especially in chimpanzees[44,45] and gorillas[46]. Upon examining the sweep identified by both SweepFinder2 and saltiLASSI on chromosome 10 in western lowland gorillas, we find three bitter taste receptor genes (*TAS2R14*, *TAS2R20*, and *TAS2R50*) within the sweep region, corroborating previous findings[46]. In chimpanzees, Hayakawa et al.[44] conclude that balancing selection was the main driver for western chimpanzee taste receptor evolution and purifying selection in the human TAS2R cluster in eastern chimpanzees. Notably, our results find a selective sweep pattern in eastern chimpanzees harboring *TAS2R42* (**Supplementary Table VII.31**), indicating that positive selection in bitter taste reception might also play a role in chimpanzees. We also identified one significant sweep region in bonobos that contain TAS2R genes (**Supplementary Table VII.31**). In sum, our results suggest local adaptation at taste receptor genes for western lowland gorillas, eastern chimpanzees, and bonobos.

MHC region and immune-related function

As stated above, we observed complex selection patterns in the MHC region. In the case of bonobos and chimpanzees, previous hypotheses have postulated that an ancient sweep occurred in the ancestor of bonobos and chimpanzees in MHC-I driven by an adaptation to better combat a SIV-like retrovirus[41-43]. However, Pawar et al.[63] did not find evidence for an ancient sweep in the

central-eastern chimpanzee ancestor (possibly due to lack of power), instead suggesting recent balancing selection in the defense against SIV infection in central and eastern chimpanzees[63,64] as the more likely explanation for MHC genetic patterns. Our results show evidence for both positive and balancing selection in bonobos in the MHC class I and II regions (**Supplementary Table VII.29; Supplementary Fig. VII.24A**), but only evidence for balancing selection (see above discussion) for central and eastern chimpanzees (**Supplementary Fig. VII.24A-B**). Similar to Pawar et al.[63], we do not see evidence of positive selection in central and eastern chimpanzees in the MHC-I region but instead two large peaks of diversity at the left and right ends of the region (**Supplementary Fig. VII.24**). However, we do see positive selection signatures in western chimpanzees between the class I and class II gene regions (**Supplementary Table VII.29; Supplementary Fig. VII.24D**).

Gene sets related to MHC protein-binding and antigen processing were significantly enriched sweep regions in bonobos (**Supplementary Table VII.33**). While there were several different genes included in the enriched pathways, *TAP1* and *TAP2* were found in every MHC-related pathway for bonobos (**Supplementary Table VII.33**). *TAP1* and *TAP2* are viral interacting proteins (VIPs) harboring variation associated with risk of respiratory infection (e.g., by influenza)[65] and interact with herpes and pox viruses[66] in humans. In a previous study, Enard et al.[67] reported increased rates of adaptation in VIPs in mammal lineages. This sentiment is echoed by Pawar et al.[63] who found selection on VIPs involved with herpes and influenza, among other viruses, in gorillas. Our results indicate local adaptation at VIPs in bonobos, adding support to the hypothesis that viruses are a major driver of protein adaptation in mammals.

Fst increased within SweepFinder2 sweeps, but not within saltiLASSI sweeps

We compared Fst values outside and within eastern chimpanzee sweep regions and found an increase in Fst within sweep regions (meanout = 0.09, meanwithin = 0.21, two-sided Mann-Whitney, z = -14.35, $p < 2.2e-16$; **Supplementary Fig. VII.27**). We see a similar significant increase in Fst within central chimpanzee sweeps (meanout = 0.09, meanwithin = 0.15, Mann-Whitney, z = -5.44, p = 5.19e-8; **Supplementary Fig. VII.27**), but the increase is not as high. However, eastern chimpanzees have significantly higher Fst values within sweeps compared to central chimpanzees (Mann-Whitney, z = -3.40, $p < 0.001$). Moreover, the maximum Fst value within central chimpanzee sweeps was 0.42 whereas the maximum Fst value for eastern chimpanzees was 0.67. For saltiLASSI sweeps, we observe the opposite pattern with Fst decreasing within eastern chimpanzee sweep regions (meanout = 0.09, meanwithin = 0.07, Mann-Whitney, $p = 0.047$; **Supplementary Fig. VII.28**), but no difference for central chimpanzee sweep regions (meanout = 0.09, meanwithin = 0.09, Mann-Whitney, $p = 0.63$; **Supplementary Fig. VII.28**). Collectively, this suggests that local adaptation plays a large role in subspecies differentiation between central and eastern chimpanzees.

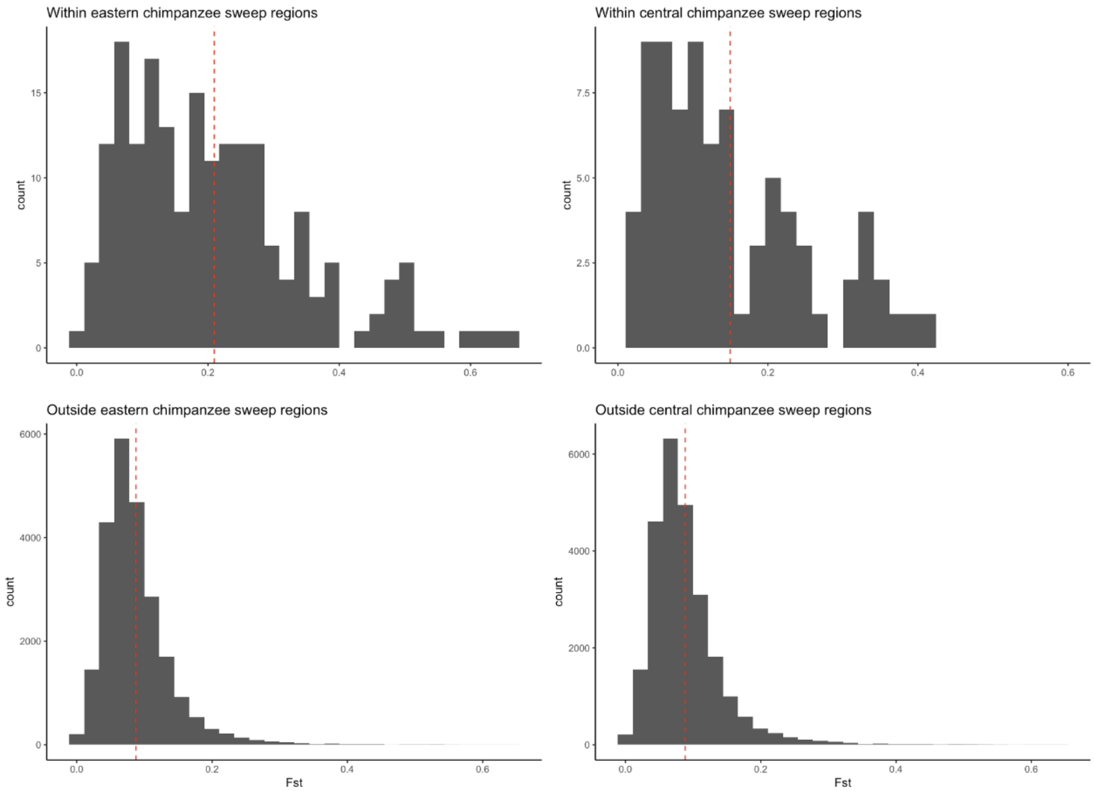

Supplementary Figure VII.27. Central and eastern chimpanzee Fst distribution within and outside of SweepFinder2 sweep regions. Histograms comparing the *Fst* distributions within and outside of central and eastern chimpanzees. The red dashed lines mark the mean *Fst* values. The mean *Fst* for outside sweep regions for both subspecies was the same as the genome-wide average (*Fst* = 0.09). The mean *Fst* within sweeps for central and eastern chimpanzees (*Fst* = 0.15 and 0.21, respectively, $p < 0.001$, two-sided Mann-Whitney test) were significantly larger than outside sweep regions.

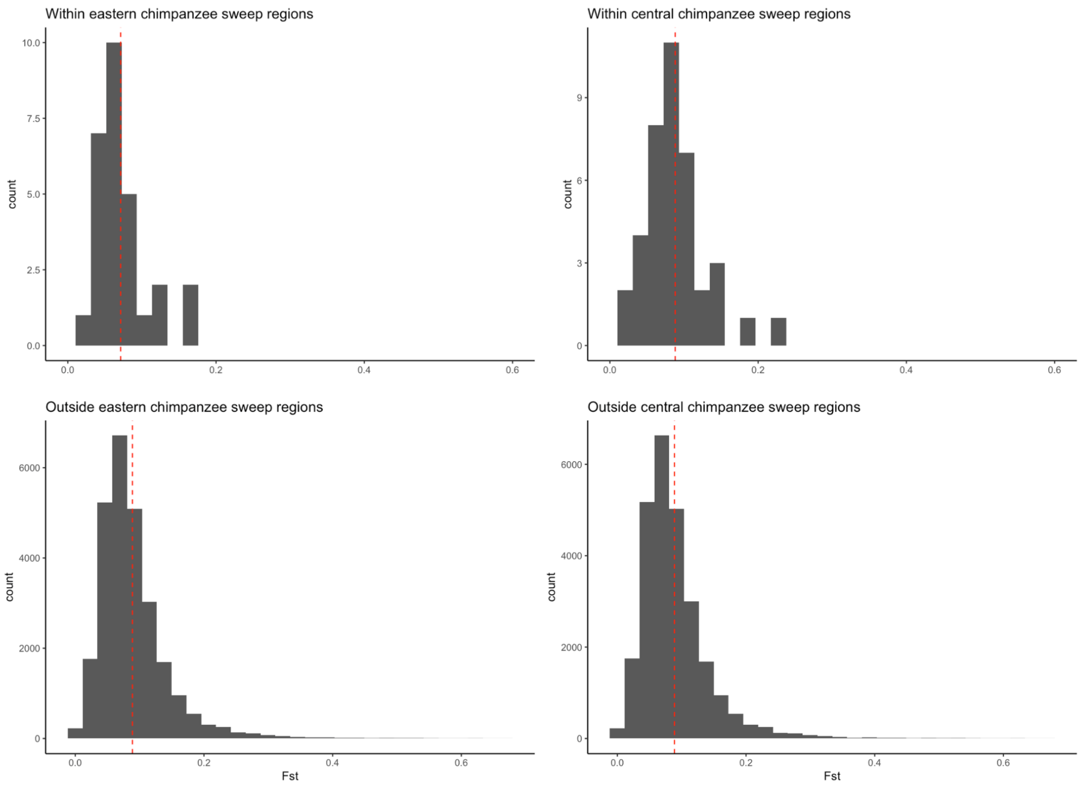

Supplementary Figure VII.28. Central and eastern chimpanzee *Fst* distribution within and outside of saltiLASSI sweep regions. Histograms comparing the *Fst* distributions within and outside of central and eastern chimpanzees. The red dashed lines mark the mean *Fst* values. The mean *Fst* for outside sweep regions for both subspecies was the same as the genome-wide average (*Fst* = 0.09). The mean *Fst* within central chimpanzee sweep regions was not significantly different (*Fst* = 0.09) and the mean *Fst* within eastern chimpanzee sweeps was significantly smaller (*Fst* = 0.07, $p = 0.047$, two-sided Mann-Whitney test).

VIII. Gene annotation

Contributing authors:

Prajna Hebbar, Francisca R. Ringeling, Françoise Thibaud-Nissen, Diana Haddad, Patrick Masterson, Karol Pal, Juan F. I. Martinez, Mark Diekhans, Stefan Canzar, Kateryna D. Makova, Benedict Paten

Methods

RefSeq annotation

The *de novo* gene annotations of the six primate assemblies were performed by the NCBI Eukaryotic Genome Annotation Pipeline (version 10.2) between Feb 27 and Mar 13, 2024, as previously described[1]. Protein-coding and long noncoding genes were predicted based on the alignments to the genome of same-species PacBio Iso-Seq and RNA-seq reads queried from the Sequence Read Archive (SRA), RefSeq human transcripts and proteins, and GenBank Primate proteins. The number of Iso-Seq reads used for the annotation ranged from 16.57 million (*Symphalangus syndactylus*) to 21.99 million (*Pan paniscus*) while the number of RNA-seq reads ranged from 1.34 billion (*Pongo pygmaeus*) to 6.96 billion (*Pan troglodytes*).

CAT gene annotation

Genome annotation was performed using CAT. First, whole-genome alignments between the ape (gorilla, chimpanzee, bonobo, Sumatran orangutan, Bornean orangutan, and siamang) and human GRCh38, and T2T-CHM13v2.0) genomes were generated using Cactus (8-way primary alignment described above). CAT then used the whole-genome alignments to project the UCSC GENCODEv35 CAT/Liftoff v2 annotation set from T2T-CHM13v2 to the primates. CAT was run with transMap, AUGUSTUS, Liftoff[68], AUGUSTUS-PB, and miniprot[69] modes. transMap lifts over gene annotations from the reference onto all the genomes in the cactus alignment. Liftoff lifts over gene annotations from a reference onto a minimap2 alignment between the reference and target genome. The miniprot mode uses protein homology information to improve gene annotations. CAT was given Iso-Seq FLNC data to provide extrinsic hints to the Augustus PB (PacBio) module of CAT, which performs *ab initio* prediction of coding isoforms. CAT then combined these *ab initio* prediction sets with the various human gene projection sets to produce the final gene sets and UCSC assembly hubs used in this project.

RNA-seq reads were aligned using minimap2[70] using the following command:

```
minimap2 -a -x sr --sam-hit-only --secondary=no --eqx -t 4 mmdb/0.mmi
rnaseq_data/0_0.fasta
```

Iso-Seq reads were aligned using minimap2 using the following command:

```
minimap2 -ax splice:hq -uf --sam-hit-only --secondary=no --eqx -t 4 mmdb/0.mmi
isoseq_data/0_0.fasta
```

CAT[69] (https://github.com/ComparativeGenomicsToolkit/Comparative-Annotation-Toolkit) was run using the following command:

```
luigi --module cat RunCat --hal=8-t2t-apes-2023v2.hal --ref-genome=hs1 -- workers=10 -
-config=t2t.apes.config --work-dir t2t_apes_2023v2/cat_work --out-dir
t2t_apes_2023v2/cat_output --local-scheduler --binary-mode local --augustus --augustus-
pb --liftoff –miniprot --maxCores 45 --assembly-hub >& log_t2t_apes_2023v2_CAT.txt
```

with the T2T-CHM13v2 annotation from UCSC GENCODEv35 CAT/Liftoff v2 as input along with a config file with locations to reference gff3, RNA-seq, and Iso-Seq BAM files.

Novel gene annotation and curation of the integrated protein-coding gene annotation set

The annotations generated by CAT were first compared with the gene annotations generated by the NCBI RefSeq pipeline. For protein-coding genes, the two sets displayed a high concordance, with an average Jaccard similarity score of over 0.9. Upon inclusion of pseudogenes and other noncoding genes, the similarity scores drop to 0.78 due to differences in biotype assignment methods between the pipelines. To resolve these differences, we provide a unique and useful gene annotation resource in the form of a consensus gene annotation between the two pipelines. To generate this, a reliable orthologous gene set was first generated. The orthologous genes were identified using the transMap method of CAT, which uses the Cactus alignment to map the orthologs. The cases where genes were mapped to a completely different neighborhood than the one in human were flagged and resolved using mappings from the liftoff mode. Then loci of all the protein-coding genes from this set were compared to the orthologous loci assigned by the NCBI RefSeq pipeline. For genes that were mapped to two completely different loci, the transcripts from both were mapped against human. Depending on the percentage identity of the generated protein to human (>50%), the transcripts were either discarded or assigned as ortholog/novel paralog. The novel gene loci that were annotated by either pipeline were collected and filtered on three levels: length of the protein generated >200 AA, identity with human protein >50%, and Iso-Seq transcript support. These were then merged into the consensus gene set.

Lineage-specific gene family analysis

Using the CAT gene annotations, we find 99.0%-99.63% of human genes annotated on the primates with at least 90% completeness. There are about 185 gene families that have fully intact protein-coding copies present only in humans. We also identified a fraction (2.0%-3.4%) of putative protein-coding genes present in the T2T genomes of the NHPs that were absent in the

human annotation set used. In addition to this, 2.1%-5.0% of transcripts annotated exhibited Iso-Seq-supported splice junctions that are unique to the NHPs. The novel protein-coding genes and exons, which have been identified as gained or lost between humans and NHPs, are documented in **Supplementary Table VIII.34**. There have been a number of gene family expansions in the NHPs, with between 1394-2056 novel gene copies found across the 184-258 families. Around 50% of these overlap with lineage-specific SD regions. A few of these occur in regions of interest.

Segmental duplication (*MAPKBP1*, *JMJD7-PLA2G4B*, *SPTBN5*) in chr1 in gorilla

MAPKBP1 codes for a protein that plays a regulatory role in the JNK and NOD2 pathways. This is a gene present on chromosome 15 in humans. The ortholog of this gene is present in chromosome 16 (homologous to 15) in gorillas. However, there is a significant expansion of this gene in chromosome 1, in tandem with the *JMJD7-PLA2G4B* and *SPTBN5* genes (**Fig. 8**). The three genes form a unit that is repeated eight times in a region spanning 13.5 Mbp near the breakpoint of double tandem inversion specifically found in the gorilla genome. It is important to note that each copy of *MAPKBP1* and *JMJD7-PLA2G4B* are supported by Iso-Seq transcripts and have valid open-reading frames (ORFs). There are also six copies of *PLA2G4B-JMJD7* spanning at least 80% of the homologous human sequence in chimpanzee and three copies in bonobo, all supported by multiple Iso-Seq transcripts (**Supplementary Table VIII.38**).

Expansion (*HERC2*, *GOLGA6/8*, *MCTP2*) in chr16 in *Pongo*

HERC2 is a duplicon that has been associated with the common breakpoint regions of Prader-Willi and Angelman syndrome deletions along with *GOLGA6* and *GOLGA8*. In genomic regions spanning over 20 Mbp in both orangutans, multiple copies of these genes, along with copies of other medically important genes, such as *MCTP2*, have been identified. For the Bornean orangutan, 25 copies of *GOLGA6*, 33 copies of *GOLGA8*, 23 copies of *HERC2*, 5 putative *HERC2-GOLGA* fusion gene copies, and 5 copies of *MCTP2* were located. In Sumatran orangutan, there are 34 copies of *GOLGA6*, 42 of *GOLGA8*, 21 copies of *HERC2*, 11 putative *HERC2-GOLGA* fusion gene copies, and 4 *MCTP2* copies. Every *HERC2* copy annotated in the **Supplementary Table VIII.38** has Iso-Seq support. However, not all *GOLGA* copies have Iso-Seq support.

LRPAP1 expansion in gorilla

LRPAP1 encodes for a protein that interacts with the LDL-receptor protein and is present in Chromosome 4 in humans. *LRPAP1* was also previously known to be associated with dementia and Alzheimer's disease in humans[71,72]. The ancestral copy of this gene is present in Chromosome 3 (homologous to HSA4) in gorillas. This gene family has expanded to 10 copies (1 ancestral and 9 copies), in gorilla across multiple chromosomes, all mapping to lineage-specific SD regions (**Supplementary Table VIII.38**). We also find that *LRPAP1* gene expansion

carried two flanking genes, *DOK7* and *HGFAC*, although expression was only observed for *LRPAP1*. Apart from the ancestral copy in Chromosome 3, four additional copies in Chromosome 12 (copy number 1-4), two in Chromosome 14 (copy 5-6), and one each in Chromosomes 16, 22, and Y (copy 7-9) were identified (**Supplementary Table VIII.38**). It is also to be noted that two copies, one in Chromosome 12 (copy 2) and one in Chromosome 14 (copy 6), exist as solitary *LRPAP1*, without *DOK7* and *HGFAC*.

Examining the protein product sequence of *LRPAP1*, we observed high identity of 98-99.1% of peptide sequence identity for the copy numbers 1, 4, 7, intermediate identity of 83.7-91.3% for copy numbers 5, 8, 9, and relatively low identity of 65.2-79.2% for copy numbers 2, 3, 6, compared to the ancestral copy located in Chromosome 3 (**Supplementary Table VIII.38**). Investigating the gene expression status of *LRPAP1*, we observed expression of the *LRPAP1* copy in Chr12 (copy 1, 2, 4), Chr14 (copy 5, 6), and Chr22 (copy 8; **Supplementary Table VIII.38**). The latter is expressed only in the testes suggesting tissue-specific differentiation. In terms of methylation, we observed hypomethylation of promoter regions without much difference among the different copies.

PSMA5 **expansion in** ***Pan*** **and Gorilla**

PSMA5 gene duplication with SORT1-like pseudogene in chimpanzee, bonobo, and gorilla
PSMA5 is duplicated twice in chimpanzee and bonobo and thrice in gorilla in conjunction with the *SORT1*-like pseudogene right downstream of it in all cases. This is seen in both haplotypes. These duplicated copies occur upstream of the ancestral copy in all of the cases and all copies have Iso-Seq support and valid ORFs. The duplicated *PSMA5* copies are all truncated in the same manner, and the accompanying *SORT1* copies are all pseudogenized. All of these regions overlap with lineage-specific SD regions in the species as well.

In addition, genes that specifically duplicated in orangutans with Iso-Seq support as well as valid ORF were found as follows:

HTATIP2: 2 copies in each haplotype
PRMT3: 2 copies in each haplotype
HEATR6: 2 copies in each haplotype
FAHD2A: 2 copies in each haplotype
COG7: 3 copies in each haplotype
RFX8: 5 copies in each haplotype
RBIS: 5 copies in each haplotype
PIGW: 3 copies in each haplotype
HSFX2: 2 copies in each haplotype
NUTM2B: 2 copies in each haplotype

Analysis of human *Pongo* and *Pan*-specific genes

Novel gene copies that were present only in the specific genus were collected and analyzed for gene ontology enrichment; 185 human-, 212 *Pan*-, and 234 *Pongo*-specific expanded genes families, which qualify by having novel copies with Iso-Seq support and valid ORFs, were used. Gene ontology analysis suggests enrichment of metabolic process in *Pan* genus, as well as signaling and neurogenesis/nervous system development functions in *Pongo* (**Supplementary Table VIII.39**).

Improved Iso-Seq transcript mapping

All long-read samples were aligned using minimap2 v2.24-r1122[70] with parameters recommended for PacBio Iso-Seq cDNA (-ax splice:hq -uf) allowing up to 15 alignments per read (-N 15). Mismatch and indel rates were computed based on primary alignments only using Perbase v0.9.0 (Stadick 2023; https://github.com/sstadick/perbase). Mismatch rates are defined as the sum of all bases covered by at least one read with a base different from the reference, divided by all covered bases. Indel rates correspond to the sum of all bases overlapping an insertion or deletion, divided by all covered bases. Reads for which the total number of soft-clipped bases exceeded 200 bp were counted using a custom awk script.

Transcripts were assembled from aligned reads using StringTie2 v2.2.1[73] with default parameters. We did not provide a reference annotation file to be able to attribute differences in transcript assembly to the quality of the reference sequence alone. To compare transcripts assembled from reads mapped to the T2T reference and from those mapped to previous assemblies, we lifted gene models inferred by StringTie2 for T2T genomes over to previous assemblies using Liftoff v1.6.3[68]. Based on the new coordinates, we then compared transcripts assembled based on T2T and previous genome versions using Gffcompare v0.12.9[74], which defines two transcripts as equal (class code "=") if the coordinates of all donor and acceptor sites match, that is, if they contain the same set of introns.

StringTie2 and most other existing algorithms infer transcripts locus by locus. If not relying on a gene annotation, loci are identified by sets of reads, i.e., read bundles, that together span a (almost) contiguous genomic region. We ran StringTie2 with option -v and parsed the output with custom scripts to collect read bundles that allow for gaps, i.e., genomic regions that are not covered by any reads, of length at most 50 bp. The similarity of bundles formed by reads mapped to T2T and to previous assemblies was measured by the Jaccard index. The Jaccard index, or Jaccard similarity, between two bundles RB_{T2T} and RB_{prev} takes values between 0 and 1 and is defined as $|RB_{T2T} \cap RB_{prev}| / |RB_{T2T} \cup RB_{prev}|$.

To find all bundles in the previous assembly that have a Jaccard similarity to any of the T2T bundles above a threshold of 0.1, we performed an all-pairs similarity search based on an index proposed in Bayardo, Ma, & Srikant[75] and implemented in Python library SetSimilaritySearch (https://github.com/ekzhu/SetSimilaritySearch). For each species, we compiled all genome-wide bundle similarities in a bipartite graph using Python package NetworkX

(https://github.com/networkx/networkx), where the two node sets correspond to bundles found in the two compared genome assemblies, and edge weights equal the Jaccard similarities.

Then when comparing protein-coding genes (**Supplementary Fig. VIII.29f**) or copy number counts (**Supplementary Fig. VIII.29g**) to previous assemblies, we find the read bundle spanning a given gene in T2T ape assemblies by querying an interval tree (https://github.com/chaimleib/intervaltree) that stores all T2T bundles, with the start and end coordinates of the gene. Traversing nodes adjacent to that read bundle in the above similarity graph then yields all similar bundles in the previous assembly. All chromosome map figures depicting bundle similarities were plotted with the aid of the R package chromoMap v4.1.1[76].

To detect multicopy gene families, we used blastp to pairwise compare protein sequences from all protein-coding genes (longest isoform per gene as annotated by NCBI). Homology was defined based on a cutoff of 50% sequence identity and 35% protein[76]. Each set of pairwise homologous genes (under transitive closure) then forms a multicopy gene family. We then represented the gene family by all distinct bundles spanning any of its members. In a second step, we extended families by paralogous loci that might have been missing in the annotation or that were pseudogenized. To this end, we found T2T bundles with Jaccard similarity >0.8 to any of the original family members. To compare copy numbers supported by RNA reads to previous assemblies, we found all bundles in previous assemblies that were similar to any of the T2T family members using the graph-based approach described above.

Supplementary Table VIII.37. Assembly accessions used in this study.

Species	T2T		non-T2T		
	Name	Accession	Name	Accession	Y amended
Gorilla	mGorGor1/Jim_GGO	GCF_029281585.2	Kamilah_GGO	GCF_008122165.1	GCA_015021865.1
Chimpanzee	mPanTro3/AG18354_PTR	GCF_028858775.2	Clint_PTRv2	GCF_002880755.1	*
Bonobo	mPanPan1/PR00251_PPA	GCF_029289425.2	panpan1.1	GCF_000258655.2	GCA_015021855.1
Sumatran Orangutan	mPonAbe1/AG06213_PAB	GCF_028885655.2	Susie_PABv2	GCF_002880775.1	GCA_015021835.1

*Chimpanzee's previous assembly already included a Y chromosome.

Summary of results

We used PacBio Iso-Seq long reads from testis RNA samples[1] of four great apes (chimpanzee, Sumatran orangutan, gorilla, and bonobo) to quantify the potential impact of T2T genome assemblies on read mapping and observed improvements in mappability, soft-clipping, and error rates. Iso-Seq reads were mapped with minimap2 to both T2T assemblies and previous

assemblies (**Supplementary Table VIII.37**). We found 1,075 (0.07%), 1,353 (0.09%), 28,925 (0.7%) and 3,361 (0.1%) reads that were unmapped to previous assemblies but mapped to T2T assemblies in chimpanzee, bonobo, gorilla, and Sumatran orangutan, respectively (**Supplementary Fig. VIII.29a**). Conversely, only a few reads that could not be mapped to T2T mapped to the previous assembly (90, 24, 171 and 769 for chimpanzee, bonobo, gorilla and Sumatran orangutan, respectively). Soft-clipping allows for the mapping of Iso-Seq reads that do not align to the genome end to end. Large soft-clipping events may indicate a missing sequence in the reference genome or a rearrangement in the sequenced individual. We found many more reads with large-scale (>200 bp) soft-clipping when they were aligned to the previous assembly compared to T2T: 29,507 (2%) versus 23,358 (1.5%) in chimpanzee; 67,428 (4.3%) versus 24,192 (1.5%) in bonobo, 89,498 (2%) versus 33,032 (0.7%) in gorilla, and 30,043 (1%) versus 27,250 (0.9%) in Sumatran orangutan (**Supplementary Fig. VIII.29b**). We then looked at mismatch and indel rates and found that Iso-Seq reads mapped to T2T had consistently lower error rates compared to the previous assembly, even though the differences are modest (**Supplementary Fig. VIII.29b**).

We hypothesized that differences in read mapping might lead to discrepancies in transcript assembly and, indeed, we found thousands of transcripts uniquely assembled from reads mapped to T2T or to previous assemblies. While the majority of transcripts assembled with StringTie2 in all analyzed species were identical in T2T and previous assemblies, we found that all species had between 4,930 (in 1,929 loci) and 10,365 (in 3,816 loci) transcripts uniquely assembled from reads mapped to one or the other genome (**Supplementary Fig. VIII.29c**). Differences in mapping statistics (soft-clipping, mismatch and indel rates) were more pronounced in loci with differently assembled transcripts compared to genomic regions where all transcripts agreed (**Supplementary Fig. VIII.29d**), suggesting a larger fraction of correctly inferred transcripts among transcripts unique to T2T than among those unique to previous assemblies.

To identify genomic regions where T2T assemblies have improved mappability of Iso-Seq reads, we searched for regions where different sets of reads mapped in T2T assemblies compared to previous assemblies. Transcript assembly algorithms such as StringTie2 group reads that map to the same locus into so-called ‘bundles’. We used the bundles generated by StringTie2 to identify these discrepant regions without relying on a specific algorithm to process them, and without relying on a high-quality gene annotation of previous assemblies. Measuring the similarity of bundles as the fraction of shared reads (Jaccard similarity, **Methods**), we found that most expressed protein-coding genes in T2T assemblies had a highly similar bundle mapped to the previous assembly (**Supplementary Fig. VIII.29f**). At the same time, we observed many regions in the T2T genome for which no similar bundles of reads mapped to the previous assembly. These regions are spread across all chromosomes, but are more prevalent around centromeric and telomeric regions, highlighting the improved resolution of repetitive regions in the T2T assembly. We also observed that low bundle similarity regions (Jaccard similarity < 0.2) overlap SDs more often than regions with a highly similar read bundle (Jaccard similarity > 0.8) (**Supplementary Fig. VIII.29e**).

We then leveraged the analysis of bundle similarities in T2T and previous assemblies to show how T2T assemblies improve the resolution of multi-copy gene families. Multi-copy gene families are prevalent in great apes and are highly relevant to the study of gene duplication and evolution. Starting from a curated list of gene families (**Methods**) and their corresponding read bundles in T2T assemblies, we looked for all similar read bundles mapping to previous assemblies and compared their numbers. For the vast majority of gene families, we found more gene copies in T2T assemblies compared to previous assemblies (**Supplementary Fig. VIII.29g**).

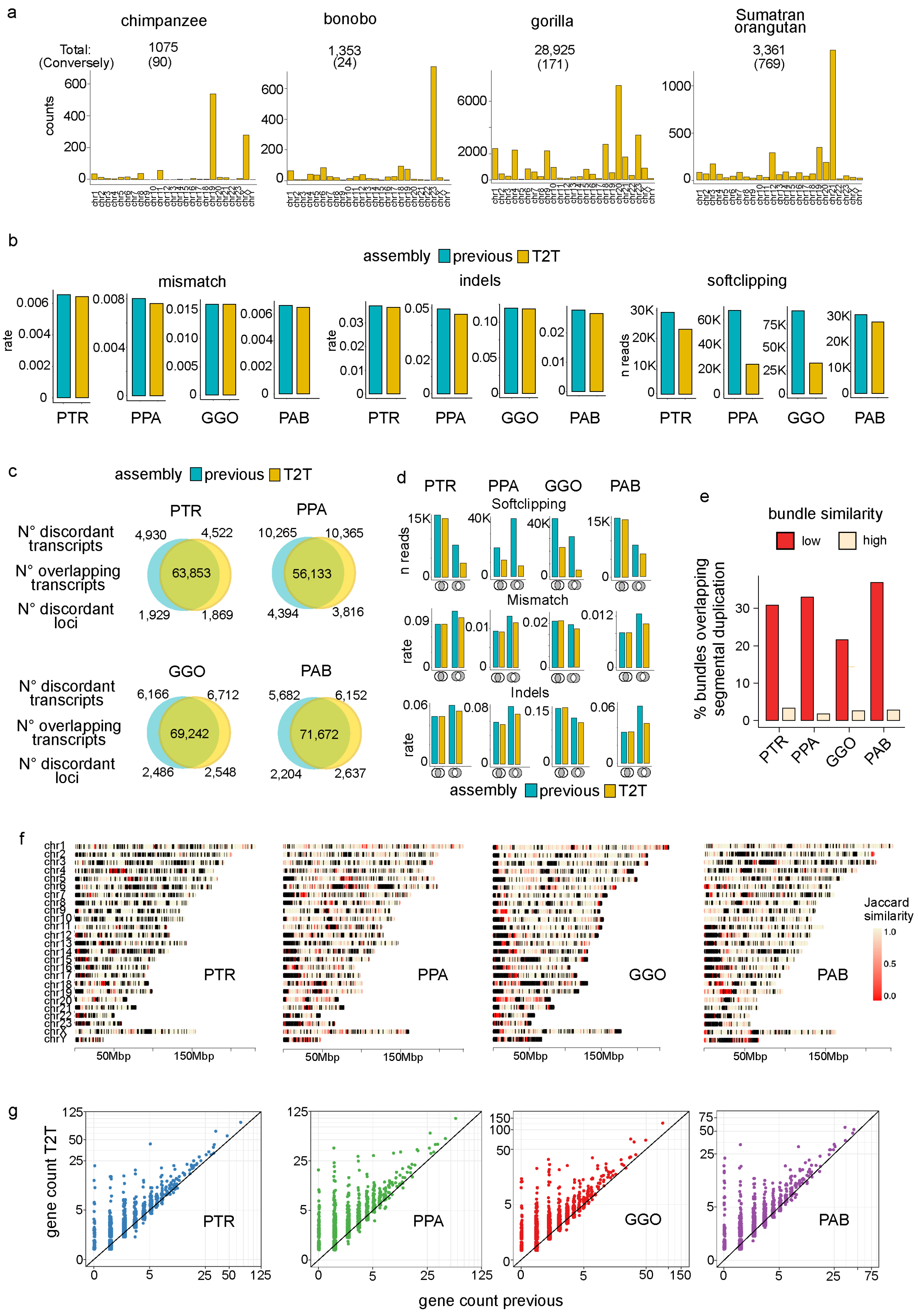
a
chimpanzee
bonobo
gorilla
Sumatran orangutan
Total: (Conversely)
1075 (90)
1,353 (24)
28,925 (171)
3,361 (769)
counts
b
assembly previous T2T
mismatch
indels
softclipping
rate
n reads
PTR PPA GGO PAB
c
N° discordant transcripts
N° overlapping transcripts
N° discordant loci
PTR
4,930
4,522
63,853
1,929
1,869
PPA
10,265
10,365
56,133
4,394
3,816
GGO
6,166
6,712
69,242
2,486
2,548
PAB
5,682
6,152
71,672
2,204
2,637
d
Softclipping
Mismatch
Indels
e
bundle similarity
low
high
% bundles overlapping segmental duplication
f
Jaccard similarity
50Mbp
150Mbp
g
gene count T2T
gene count previous

Supplementary Figure VIII.29. T2T assemblies improve transcript inference. (a) Number of reads that remain unmapped when aligned to previous assemblies but map to T2T assemblies; chimpanzee (PTR), bonobo (PPA), gorilla (GGO), and Sumatran orangutan (PAB). Each plot shows the number of such reads per chromosome. Total number is shown above each plot and in parenthesis the converse number, i.e., number of reads unmapped in T2T mapped to previous assemblies. (b) Mapping statistics for Iso-Seq reads aligned to previous or T2T assemblies. Mismatch rate was calculated as the sum of all bases covered by at least one read with a base different from the reference, divided by all covered bases. Indel rate was calculated as the sum of all bases overlapping an insertion or deletion, divided by all covered bases. For soft-clipping plots, reads for which the total number of soft-clipped bases exceeded 200 bp were counted. (c) Comparison of transcripts assembled from Iso-Seq reads aligned to previous or T2T assemblies. (d) Mapping statistics for genomic regions where transcript predictions from reads aligned to previous assemblies are equal (matching intron chains) to those from reads mapped to T2T assemblies versus regions with non-equal transcripts. (e) Overlap of SDs and genomic regions with low bundle similarity (Jaccard similarity < 0.2) versus regions with high bundle similarity (Jaccard similarity > 0.8). Bar plots show the percentage of regions that overlap in more than 80% of their length with SDs. (f) Ideograms showing T2T chromosomes colored by Jaccard similarities between bundles of reads mapped to T2T assemblies and read bundles on previous assemblies. (g) Scatter plots show number of gene copies per gene family on previous and T2T assemblies.

IX. Repeat annotation

Contributing authors:

Jessica M. Storer, Gabrielle A. Hartley, Mark Loftus, Parithi Balachandran, Panpan Zhang, Edmundo Torres-González, Hailey Loucks, Karen H. Miga, Kateryna D. Makova, Cedric Feschotte, Christine R. Beck, Miriam K. Konkel, Rachel J. O'Neill

Methods

Satellite and repeat annotations

We produced comprehensive repeat annotations across the ape lineage by integrating a combination of known repeats and models identified in human T2T-CHM13[77], T2T-Y[10], ape X/Y chromosomes[1], and *de novo* repeat curation (**Supplementary Table IX.41**). To identify canonical and novel repeats, we utilized the previously described pipeline[77], with modifications to include both the Dfam 3.691 and Repbase (v20181026)[78] libraries for each species during RepeatMasker[79] annotation. An initial RepeatMasker run identified canonical repeats, while a subsequent RepeatMasker run was completed to include repeat models first identified in the analysis of T2T-CHM13, T2T-Y, ape X/Y chromosomes (**Supplementary Table IX.42**), newly defined satellites, and 17 variants of pCht/StSat derived from Cechova, M. et al.[80]; the resulting annotations were merged. Because we previously discovered that prior taxonomic labeling for repeats that was once considered species-specific (i.e., PtERV and pCht, inaccurately labeled in current repeat libraries as *Pan troglodytes* specific) and therefore were previously excluded from the bonobo and gorilla genome repeat annotations[1], an additional RepeatMasker run between the first search for canonical repeats and a subsequent search for novel repeats was performed on the gorilla and bonobo genome assemblies. All of the results were combined as described previously[77].

To identify and curate previously undefined satellites, we utilized additional TRF[81] and ULTRA[82] screening of annotation gaps >10 kbp in length. Potential gaps were identified via BEDTools v2.29.0[83] by subtracting both the repeat and gene annotations for each ape reference sequence. To identify potential redundancy, satellite consensus sequences generated from gaps identified in each species were compared using crossmatch and were used as a RepeatMasker library to search for overlap in the other five analyzed primate species. Consensus sequences were considered redundant if there was a significant annotation overlap in the RepeatMasker output. Repeat consensus sequences were manually curated using RepeatMasker searches to ensure accuracy and identify additional variants.

Species-specific mobile element insertions (MEIs)

Species-specific (SS) non-long terminal repeat retrotransposon (i.e., non-LTR: *Alu*, LINE/L1, and SVA) MEIs were characterized from the unaligned regions of Cactus alignments of the six

great ape assemblies (PonPyg, PonAbe, GorGor, PanTro, PanPan, and hs1) with the siamang (SymSyn) gibbon assembly used as an outgroup. A local RepeatMasker (v4.1.6) installation with a standard library (Dfam v3.8)[84] was first used to identify the repetitive element content of the SS sequences. All SS sequences less than 15 kbp in length and annotated as having a non-zero percentage of repetitive sequence (any kind of repetitive sequence) by RepeatMasker were selected as potential candidates for downstream analyses. For each candidate SS locus, 500 bases of the flanking sequence up and downstream of the insertion coordinate was retrieved and fused. This fused sequence was provided to a local BLAT[85] installation (Standalone BLAT version: 36x2, parameters: -minScore=650, gap size +/- 20% SS sequence length) to query for homologous flanking segments containing similar sequence to the SS locus within all seven genomes. The sequence for each BLAT hit was pulled using SAMtools (version: 1.10) and then cut into *k*-mers (k=14), along with the original SS locus+flanking. Their *k*-mer-profile dissimilarity[86] was calculated to quantify the dissimilarity between the SS locus+flanking and each BLAT hit. Using a conservative approach, the BLAT hit was deemed a match if the *k*-mer-profile dissimilarity was ≤0.5. A candidate SS locus was filtered from the dataset if the SS sequence+flanking was identified in any other species. If a candidate SS locus was deemed unique to a species but duplicated, it was noted and all duplications were counted as only one potential insertion. The remaining 'high-confidence' SS loci were then screened for non-LTR mobile elements (SS sequence element percentage: LINEs/SVA: ≥20% to allow for transductions, Alu elements: ≥80%). These putative SS MEIs were subsequently put through a stringent filtering process screening for A-tails (minimum tail length ≥6 bp), percent divergence (LINEs/SVAs: <15% divergence, Alu: <6%), subfamily (LINEs: L1HS/PA4/PA3/PA2/PA1, Alu elements: AluY and derivatives), element length (LINEs/SVA: ≤10 kbp, Alu: ≤500 bp), and then randomly sampled and manually spot checked for quality control.

Candidate ERVs were identified in each reference genome assembly using RetroTector with scores of ≥300[87] and a Perl script (http://doua.prabi.fr/software/one-code-to-find-them-all). The resulting ERV loci were merged and further filtered based on the presence of two flanking LTRs and a table of ERVs and their associated LTRs, as described by Kojima et al.[88]. To examine species-specific ERVs, we downloaded liftOver files from the human genome to the genomes of five apes and repeated the process for each of the other apes. In total, we used 30 liftOver files from the Genome Archive collection of UCSC Genome Browser[89]. Reciprocal liftOver analysis was conducted to infer the presence/absence of each two LTR ERVs across six primate genomes, using the parameter -minMatch=0.1 (minimum ratio of bases that must remap). Lifted ERVs shared at orthologous genomic positions were deemed ancestral and likely fixed within each species[90] and, thus, were filtered out. These sequences were further analyzed for target site duplications on each side of the ERVs using BLAT. To generate a comprehensive catalog of *gag* (capsid domain) and *pol* (reverse transcriptase and integrase) domains, we performed a six-ORF translation of two LTR ERVs using ORFfinder with the parameters -ml 300 (minimum ORF size 100 codons) and -s 1 (use the standard genetic code but allowing noncanonical start codons)[87]. Short, encoded proteins were concatenated with "N" connection based on coordinates. For the capsid CA domain homology within the *gag*, we utilized hmmscan following a previously described method[91]. We employed HMMER with default parameters, a bit-score threshold of 25,

and a length threshold of 125 amino acids[92]. For reverse transcriptase and integrase domain homology within the *pol*, the domains of all collected proteins were predicted using InterProScan[93]. Additionally, we collected all ERV proteins annotated in Repbase and the Repeat Protein Database and performed InterProScan and CD-BLAST on previously characterized consensus sequences of ERVs.

To determine intact ORFs across lineage-specific full-length L1s, we followed a previously described method[94] to detect intact ORF1p and ORF2p. To account for the millions of years of divergence between species, we lowered the amino acid similarity cut off from 5% to 15%. Once we obtained ORF calls, to curate the lower 15% threshold, we compared intact ORFs from 6-15% divergence across human assemblies and found evidence for downstream ORF2p usage, but these putative proteins retained the function of catalytic domains of ORF2p. Therefore, the thresholds we set are appropriate for proper identification of protein-coding ORFs that may be retrotransposition competent.

Nuclear sequences of mitochondrial DNA origin (NUMT)

NUMTs are not included in the RepeatMasker annotation; therefore, we used a different approach to identify them. We identified NUMTs by aligning the T2T mitochondrial assemblies to nuclear assemblies for each species, including T2T and non-T2T assemblies. We used BLASTN aligner[95] with parameters previously used to study NUMTs in the human T2T-CHM13 assembly (-evalue 0.0001 -gapopen 5 -gapextend 2 -penalty -3 -reward 2 -task blastn)[96]. Overlapping alignments and those within 1 kbp of each other were merged using BEDTools, as they likely represent the same NUMT event. The following non-T2T assemblies were used for comparison: panPan3[30], GRCh38[97], gorGor6, panTro6, and ponAbe3[98]. The panPan3, gorGor6, and ponAbe3 assemblies of female subjects were supplemented with a Y chromosome (NCBI accessions GCA_015021855.1, GCA_015021865.1, and GCA_015021835.1, respectively)[99].

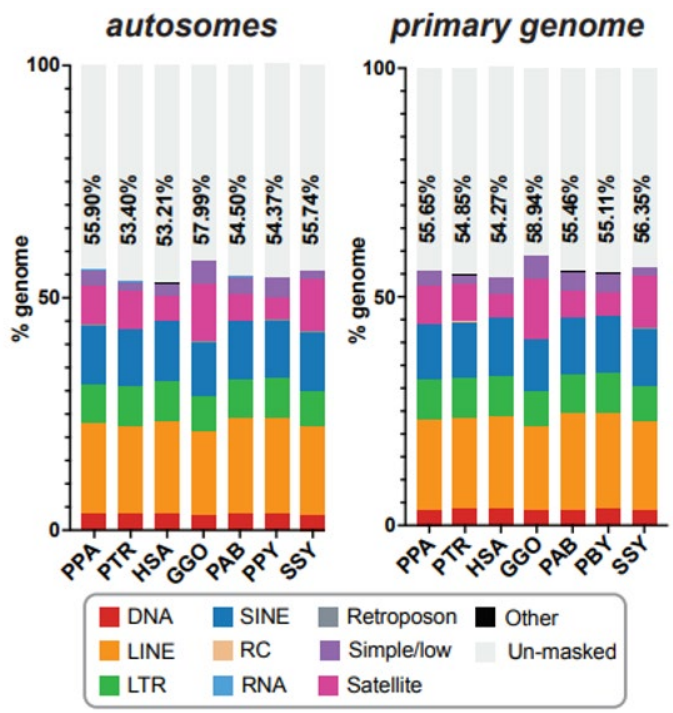

Supplementary Figure IX.30. Total repeat content of ape chromosomes.

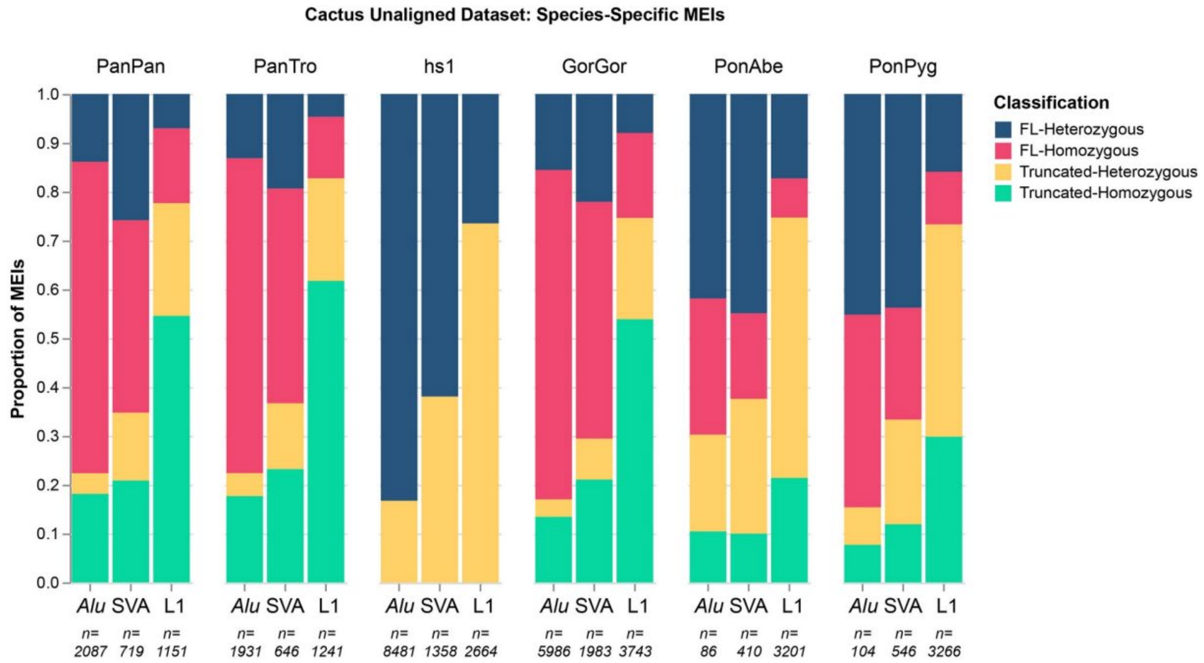

Supplementary Figure IX.31. Species-Specific MEI Classifications. A stacked bar graph showing the proportion of species-specific MEIs that are both full length (FL) and homozygous (red), FL and heterozygous (dark blue), truncated and homozygous (green), and truncated and heterozygous (yellow). MEIs were not checked for homozygosity within humans, only FL or truncated, as hs1 is a haploid genome. The PonAbe (PAB) and PonPyg (PPY) genomes show a larger proportion of heterozygous LINE/L1s compared to the African great apes suggesting an elevated retrotransposition rate of L1s in the *Pongo* lineages.

X. Immunoglobulin annotation and analysis

Contributing authors:

Yana Safonova, Corey T. Watson, Anton Bankevich, Matt Pennell, Yixin Zhu, Swati Saha, William Lees, Eric Engelbrecht, Pavel Pevzner, Ishaan Gupta, Zhenmiao Zhang

Methods

Analyses of the immunoglobulin (IG) heavy (IGH), light chain kappa (IGK) and lambda (IGL), T-cell receptor (TR) beta (TRB), TR alpha/delta (TRA/D), and gamma (TRG) loci in four ape species (bonobo, gorilla, Bornean orangutan, and Sumatran orangutan) for which two complete intact haplotypes were constructed were conducted. Genomes of two species (chimpanzee, siamang gibbon) derived from lymphoblastoid cell lines contain somatic rearrangements driven by V(D)J recombination and were excluded from the analysis.

Annotating germline IG/TR genes and loci

Germline IG and TR variable (V), diversity (D), and joining (J) genes were predicted using the IgDetective[100] and Digger[101] tools. Digger was run using human recombination signal sequences for the IG and TR genes, with output from IgDetective as the starting germline database. Boundaries of IG and TR loci were defined according to the leftmost and rightmost IG genes, extending 20 Kbp on either side. We assessed per base read support of the assemblies spanning the IG and TR loci. To ensure base-level accuracy in the genomic assemblies and read support of the predicted germline IG and TR genes, PacBio HiFi reads were remapped to each T2T genome using minimap2[70], followed by analysis of per-base read support using SAMtools[102] and CloseRead In each of the four species (Bornean orangutan, bonobo, gorilla, and Sumatran orangutan), we found that the mean coverage of remapped HiFi reads to each respective haplotype assembly ranged from 28 to 75. Additionally, 99.9% of assembly bases for the IG and TR loci were supported by at least 80% of the mapped reads, with 100% of IG/TR gene-coding bases supported at this read support level (**Supplementary Data 1**). Given the patterns of divergence and haplotype complexity in the IG/TR loci observed among species, the grouping and assignment of homologous genes among haplotypes and species is nontrivial. We determined that this will require more detailed phylogenetic and comparative genomic analysis of additional haplotypes in each species. Consequently, we determined that the use of standard gene identifiers based on position and cross-species orthology was not valid. Assignments of permanent identifiers to each unique germline sequence in each species is currently under review by the International Union of Immunological Societies TR-IG Nomenclature Review Committee (https://iuis.org/committees/nom/nomenclature-sub-committees/immunoglobulins-ig-t-cell-receptors-tr-and-major-histocompatibility-nomenclature-sc/).

Finding SD blocks

We decomposed IG/TR loci into the alphabet of duplication subunits[103] using a modification of the Sibelia tool[104] that uses iterative de Bruijn graphs for analyzing synteny blocks and SDs. This modification enabled analysis of highly repetitive IG/TR loci that result in particularly complex de Bruijn graphs where the original Sibelia algorithm has limitations. The constructed block decompositions enabled comparison of segmental/tandem duplications in IG/TR loci between different haplotypes of the same species.

Finding units of *IGHV3-30* and *IGHV4-59* tandem units

To assess units of tandem duplications containing *IGHV3-30*-like and *IGHV4-30*-like V genes in the IGH loci across species, SDs found in the bonobo IGH loci and the human IGH T2T locus were identified and aligned to all IGH loci in the other three species; the procedure was repeated until all units were detected. The detected units were numbered according to their order in the corresponding locus/haplotype. The phylogenetic tree of the units was computed using the ClustalW2 tool[105] and visualized using the Iroki tool[106]. The same procedure was applied to tandem duplications containing *IGHV1-58*-like and *IGHV4-59*-like V genes in IGH loci of the Sumatran and Bornean orangutans.

Comparative analysis of IG/TR loci and genes

Pairs of IG/TR germline sequences of all loci were aligned using YASS[107] where the longest non-overlapping alignments were selected to visualize the alignment blocks (as shown in **Extended Fig. 2a**). The repetitiveness of a locus was computed as the fraction of bases among the total bases in that haplotype that were spanned by repetitive sequence of length ≥10 kbp. To define “species-specific” V genes within a locus, the V genes (using the IgDetective gene sets) were combined with known human V genes from the same locus and the nucleotide distances between all pairs of genes were computed. The human set included all *01 alleles for every curated IG and TR in-frame gene without stop codons in the International IMmunoGeneTics Information System (IMGT) database[108] (imgt.org; date downloaded: July 1, 2024). The hierarchical clustering maximizing the number of clusters consisting of at least three nonhuman genes was applied, and genes corresponding to these clusters were reported as species-specific. Similarly, human-specific genes were computed in comparison with bonobo V genes and were identified in clusters consisting of at least two human genes. To estimate the allelic diversity of curated IG/TR genes within a species and locus, for each V gene, the closest V gene (by sequence alignment) from the alternative haplotype of the same locus and the same species was found. Distances between identified pairs of genes were collected across all six loci and four species. The pairs of genes with zero distance were referred to as identical.

Summary of results

Gene annotation

We compiled complete IG/TR gene annotation sets for each species, including annotations for in-frame sequences without stop codons V, D, and J genes/alleles. Annotation sets from IgDetective and Digger are provided in **Supplementary Data 1**, including positions within each haplotype, and in the case of Digger annotations, predicted upstream regulatory sequences, leader sequences, introns, and recombination signal sequences, in addition to the existing annotations available in VDJbase (VDJbase.org)[109].

Locus architecture and gene divergence of the ape IG and TR loci

As shown in **Extended Fig. 2a**, we computed within and between species haplotype alignments for each of the IG and TR loci (**Supplementary Fig. X.32a**). These initial comparisons revealed a greater degree of synteny between species and a higher level of structural variation within and between species in IG loci compared to TR loci (see below). To quantify this, we computed within haplotype SD blocks (see methods above) and calculated the percent of bases covered by SDs in each locus and haplotype. On average, we found that across species, the IG loci had a consistently high fraction of bases spanned by SDs. In contrast, while both TRB and TRG loci had comparable levels of SDs, the fraction of bases covered by SDs in the TRA/D locus was lowest among all species (**Extended Fig. 2b**). IGK showed the greatest degree of variation in this computed fraction and also the greatest degree of inter-haplotype length variation, which was mainly associated with long insertions (**Extended Fig. 2c**). Additionally, several tandem duplication regions were associated with length differences between haplotypes both within and between species in the IG loci. As an example, we conducted inter-haplotype and interspecies analyses of two regions in the IGH locus that harbors expanded tandem duplication blocks. In human, these two distinct regions harbor the genes *IGHV3-30* (and related paralogs) and *IGHV1-58/IGHV4-59* (**Supplementary Fig. X.32a,c**); the *IGHV3-30* region is known to be highly diverse with respect to structural variation in the human population[110]. Of note, these regions exhibited extensive expansion and contraction between ape species (**Supplementary Fig. X.32a,c**). Furthermore, a subset of the identified duplication blocks of human and bonobo clustered into species-specific clades within the phylogenetic trees, revealing the likelihood of complex evolutionary signatures. These patterns indicated that in addition to within species sequence divergence, a combination of species-specific recurrent duplications/deletions alongside sequence conversion have plausibly occurred. Specifically with respect to the identification of and study of human and ape orthologs in these regions, additional haplotypes and phylogenetic analyses will be required to accurately trace their evolutionary histories.

In addition to tandem duplication expansions and contractions, we also noted large SVs, particularly in the IG loci, including insertions and inversions between species and haplotypes. Comparison of two haplotypes of the bonobo IGL locus revealed a 1.4 Mbp inversion (denoted

as INV) flanked by a mosaic tandem repeat consisting of directed and inverted units of two types denoted as A (~58 kbp) and B (~27 kbp) with variable counts of repeat copies (**Supplementary Fig. X.32b**). The maternal haplotype can be represented as $A_1B_1A_2B_2A_2$ + INV + A_3'B_3'A_4' and the paternal haplotype can be represented as $A_5B_4A_5$ + INV' + A_6', where ' refers to the inverted orientation of the block. These observations raised a concern about the assembly accuracy, as the complex repeat structure marks hotspots for possible structural assembly errors. To verify the assembly quality, HiFi reads were assembled using the LJA genome assembler based on construction of highly accurate de Bruijn graphs[111]. Analysis of the de Bruijn graph revealed that the flanking repeat copies A and B from each distinct haplotype were sufficiently diverged from one another, making assembly error practically impossible. Contigs generated by LJA also supported the presence of the inversion within the bonobo IGL locus.

As expected, these large SVs were associated with the presence of species-specific genes (defined in Methods), including examples of IGHV genes residing within tandem SD/repeat expansions and contractions (**Extended Fig. 2a, Supplementary Fig. X.32a,c**). Across all species, the greatest number of species-specific genes were observed in IGH (**Supplementary Fig. X.32d**), which positively correlated with a greater density of long repeats (≥10 kbp) in IGH relative to the other five loci (r=0.51, P=6.95×10–5; **Extended Fig. 2d**). In addition, within species, the IG loci were characterized by higher V gene distances between haplotypes (P=0.013, Kruskal-Wallis test) and lower fractions of identical V gene sequences between haplotypes ($P=3.03\times10^{-13}$, Kruskal-Wallis test) compared to TR loci (**Supplementary Fig. X.32e,f**). While these haplotypes provide clear evidence for rapid divergence in these critical immune loci, it is imperative to note that further sampling of haplotypes for these species will be necessary for characterizing additional SVs and building more complete sets of IG/TR genes and alleles for each species. Even in humans, IG and TR genes continue to be discovered[112].

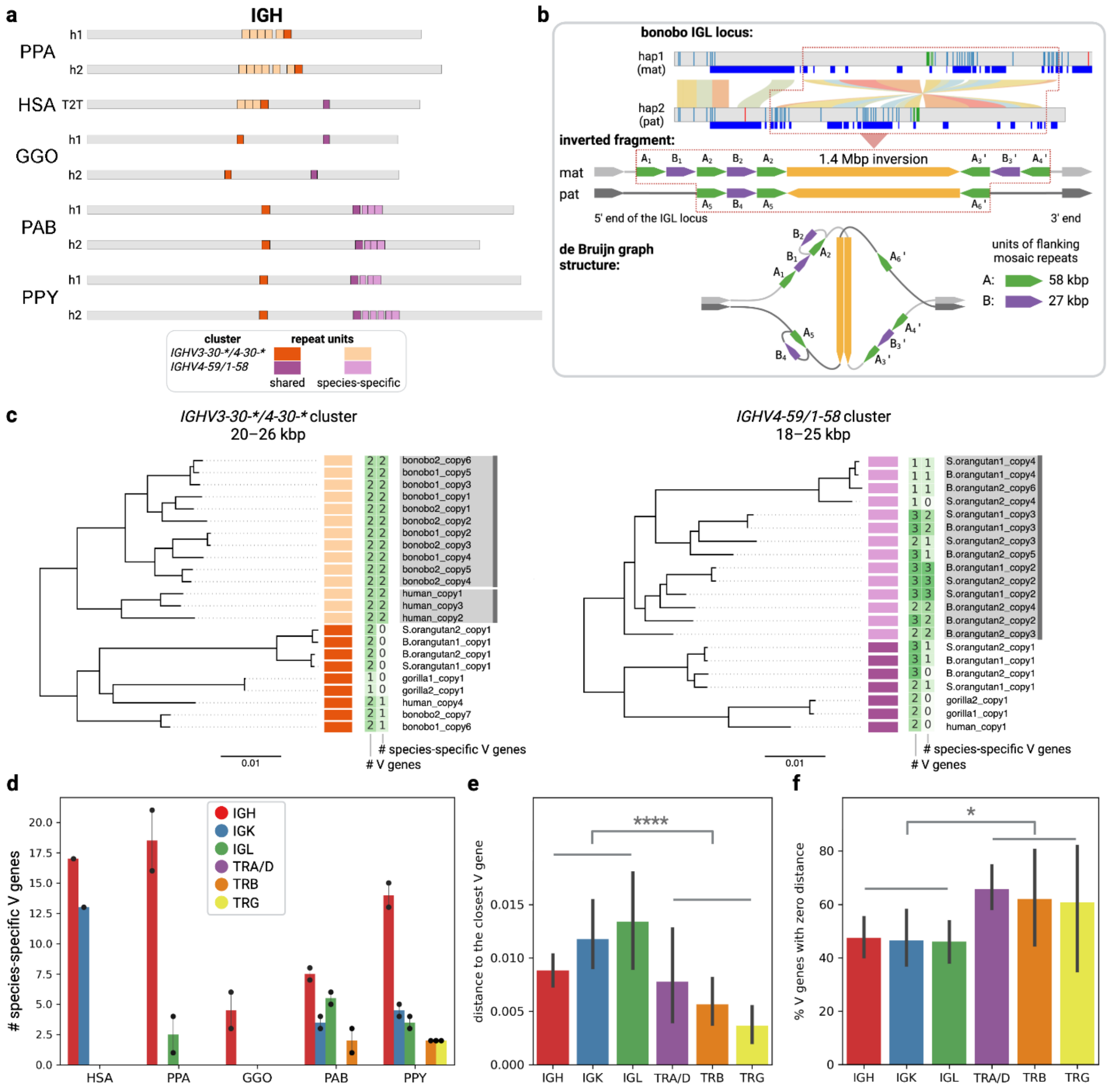

Supplementary Figure X.32. Comparative analysis of IG/TR loci and genes across four great ape species. (a) Diagram showing positions of two tandem repeat units containing IGHV3-30-*/4-30-* genes (orange) and IGHV4-59/1-58 genes (purple) in IGH loci of five ape species; bonobo (PPA), human (HSA), gorilla (GGO), Sumatran and Bornean orangutans (PAB and PPY). Shared and species-specific units are colored in dark and pale colors, respectively.
(b) Genomic analysis of the inversion structure in the bonobo IGL locus. The top part shows an alignment of two haplotypes of the bonobo IGL locus with the inverted fragment shown as a red dashed rectangle. The middle part shows the genomic structure of the inverted fragment that includes a 1.4 Mbp inversion (shown in yellow) and mosaic repeats flanking it (shown in green and purple). The bottom part shows the simplified structure of the de Bruijn graph corresponding

to the inverted fragment. For visualization purposes, bulges representing divergence between haplomes were not shown in the graph. Lengths of genomic blocks and de Bruijn graph edges are not up to scale. (c) Phylogenetic trees of units of the IGHV3-30-*/4-30-* and IGHV4-59/1-58 clusters. Colors of shared and species-specific units are consistent with panel B. Counts of all V genes and species-specific V genes in each repeat unit are shown in green. Subtrees corresponding to bonobo (the left tree), the human (left), and both orangutan species (right) are highlighted in gray. (d) Counts of species-specific V genes across IG/TR loci and five great ape species. (e) The distances between closest pairs of V genes from different haplotypes within the same locus and species collected across IG/TR loci and four ape species (PPA, GGO, PAB, PPY). The distance is computed as the fraction of non-matching positions in the alignment. (f) The fractions of V genes from different haplotypes within the same locus and species with identical gene sequences collected across IG/TR loci and four ape species (PPA, GGO, PAB, PPY).

XI. MHC I and MHC II analyses

Contributing authors:

Joanna Malukiewicz, Britta S. Meyer, Mihir Trivedi, Prajna Hebbar, Tobias L. Lenz

Methods

We defined the MHC genomic region as all loci located between *GABBR1* and *KIFC1,* as previously determined by Shiina et al.[113] This region is located on chromosome six in humans[113]. We first identified the chromosomal location of the MHC genomic region in the ape T2T assemblies of bonobo (*Pan paniscus*; NCBI assembly NHGRI_mPanPan1-v2.0_pri), chimpanzee (*Pan troglodytes*; NCBI assembly NHGRI_mPanTro3-v2.0_pri), western lowland gorilla (*Gorilla gorilla gorilla*; NCBI assembly NHGRI_mGorGor1-v2.0_pr), Bornean orangutan (*Pongo pygmaeus*; NCBI assembly NHGRI_mPonPyg2-v2.0_pri), Sumatran orangutan (*Pongo abelii*; NCBI assembly NHGRI_mPonAbe1-v2.0_pri), and siamang (*Symphalangus syndactylus*; NCBI assembly NHGRI_mSymSyn1-v2.0_pri). The chromosomal locations of each ape MHC genomic region were located by first aligning each respective haplotype of each T2T ape assembly to the human T2T assembly (NCBI assembly T2T-CHM13v2.0) as a reference with minimap2 v2.28[70]. SAMtools[114] was then used to filter resulting SAM files for mapped reads with the "-F4" flag with the view subcommand. Then filtered SAM files were sorted with SAMtools sort subcommand and simultaneously converted to BAM file format. BAM files were indexed with SAMtools index subcommand. Finally, the SAMtools view subcommand was used to subset BAM files from the regions of each ape assembly haplotype that mapped to human chromosome 6 between coordinates 2937649-3331258. These coordinates flank the MHC region of human T2T assembly T2T-CHM13v2.0. Additionally, we applied the same approach to extract MHC region sequences of four previously published great ape genomes for comparison[115]: bonobo (NCBI assembly Mhudiblu_PPA), chimpanzee (NCBI assembly Clint_PTR), western lowland gorilla (NCBI assembly Kamilah_GGO), and Sumatran orangutan (NCBI assembly Susie_PAB).

To annotate putative classical and nonclassical MHC class I and class II genes within the two individual haplotypes of each ape T2T genomic assembly, we used two complementary approaches. First, we used EXONERATE 2.4[116] with the "est2genome" mapping model (https://www.ebi.ac.uk/about/vertebrate-genomics/software/exonerate). EXONERATE was run recursively with functional human HLA gene and CDS annotations from T2T-CHM13v2.0 and the IPD-MHC database (Release 3.12.0.0 build 211; https://www.ebi.ac.uk/ipd/mhc/) gene and CDS annotations for chimpanzee, bonobo, western lowland gorilla, and both orangutan species. For all ape species except siamang, chromosome 5 was the query and for siamang chromosome 23 served as the query and EXONERATE results were filtered to matches of greater than 95%.

Second, we mapped human HLA gene annotations to chromosome 23 of siamang and chromosome 5 for all remaining ape species with minimap2. Results between EXONERATE and minimap2 were compared for concordance. Then annotations were manually verified and curated with ALIVIEW 2.8[117] to retain only a single gene annotation per locus, and all gene annotations were individually compared against all available human and ape species orthologs to confirm proper annotation. Due to the absence of previous gene and CDS annotations for the siamang, we assigned MHC gene annotations for presumed start codon to stop codon based on corresponding human HLA gene and CDS annotations. MHC class I and class II gene names were assigned according to human and ape species orthologs.

The results from this manually curated annotation approach were compared to MHC annotations of the automated genome-wide CAT annotation pipeline. Since these two approaches use different criteria and evidence for annotation, minor discrepancies are expected. **Supplementary Tables XI.57-58** provide a detailed comparison of the annotation results, as well as a consensus call that was reached by phylogenetic comparisons.

Phylogenetic trees were produced for MHC class I loci and MHC class II DRB loci to confirm the identity of MHC genes exhibiting copy number variation. First, separate MHC class I and MHC-DRB multiple sequence alignments were produced with the online version of MAFFT (https://mafft.cbrc.jp/alignment/server/index.html). Both alignments consisted of genomic sequence of annotated MHC genes from ape T2T assemblies, human coding sequence (CDS) and genomic sequence of genes from T2T-CHM13v2.0. Genomic and coding sequences for MHC class II DRB genes (n=71) and MHC class I loci (n=406) were obtained from the IPD-MHC database (https://www.ebi.ac.uk/ipd/mhc/, Release 3.12.0.0 (2024-01) build 211). Multiple sequence alignments were created using MAFFT[118].

To generate phylogenetic trees from sequences from all MHC class I and the MHC class II DRB genes, respectively, the IQ-TREE platform (http://iqtree.cibiv.univie.ac.at) was used with default settings[119]. IQ-TREE automatically selected the best-fitting substitution models and calculated node support for the phylogenetic trees with Ultrafast Bootstrap[120]. Phylogenetic trees were plotted using RStudio (version 2024.04.2+764) with the ggtree[121] and ape[122] packages, retaining bootstrap values above 70.

Dot plots were used to highlight structural variations between the haplotypes of each primate species. Two haplotypes from each species were aligned using NUCMER and show-coords from the MUMmer package v4.0.0rc1[123] to generate delta and coordinates files from the FASTA sequences. These coordinates, along with BED file annotations for coding genes and pseudogenes within the MHC regions, were used to generate and customize the dot plots with the SVbyEye package (https://github.com/daewoooo/SVbyEye)[124] in R, along with ggplot2[125].

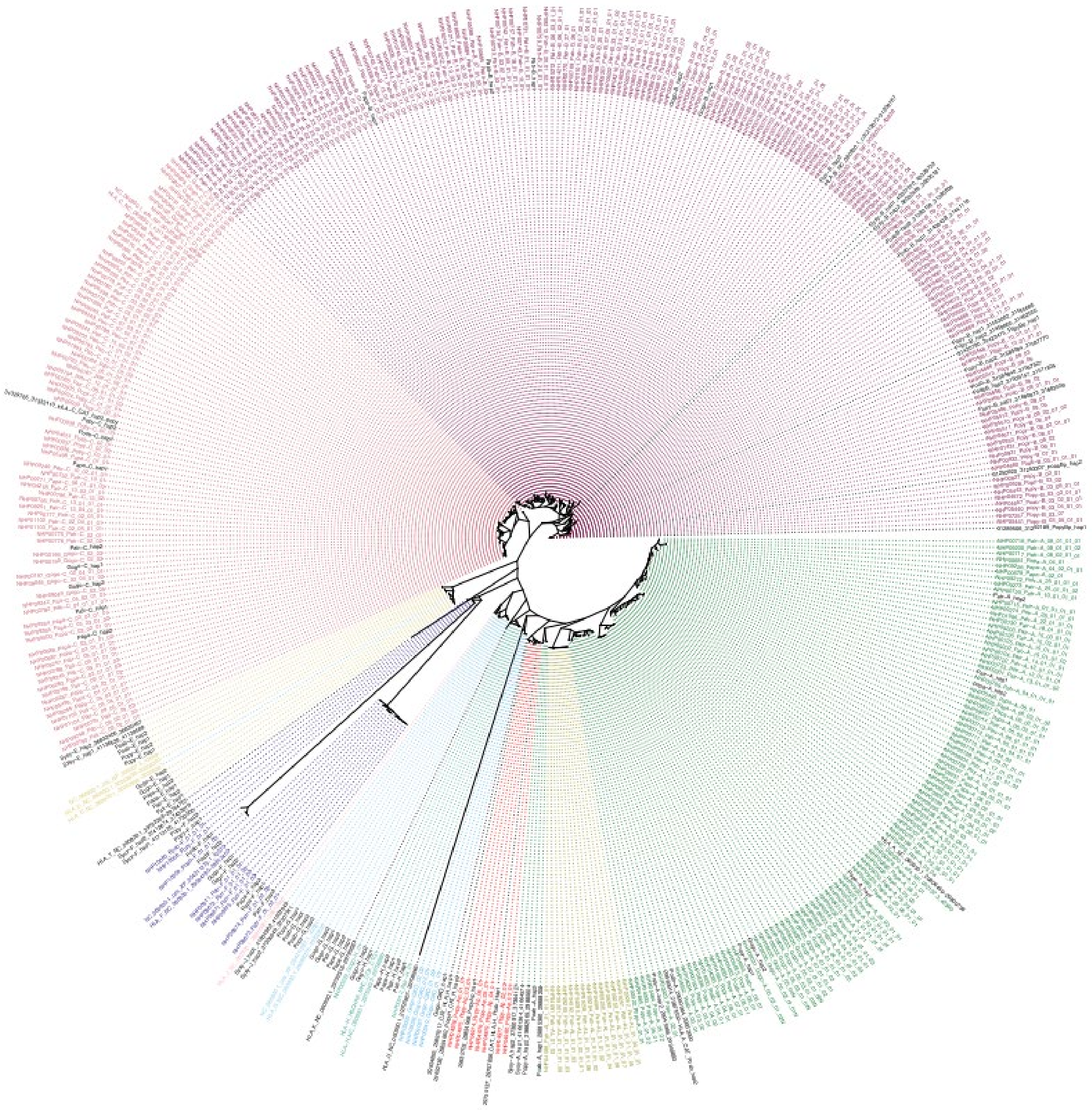

Supplementary Figure XI.33. Unrooted maximum likelihood phylogeny of MHC class I genes. Phylogeny of genomic sequence of human ape MHC class I loci from the six T2T assemblies, genomic sequence of species MHC class I genes obtained from the IMGT MHC database, and annotated MHC class I genes from the human T2T-CHM13v2.0 genomic assembly. NHP gene names are abbreviated according to species (Patr-*Pan troglodytes*, Papa-*Pan paniscus,* Gogo-*Gorilla gorilla*, Popy-*Pongo pygmaeus*, Poab-*Pongo abelii,* Sysy-*Symphalangus syndactylus*). Clusters of orthologous loci are represented by unique colors. Newly annotated MHC I genes from this study are shown in black. IQ-TREE chose GTR+F+I+G4 as the best fitting model for this tree following the BIC criterion.

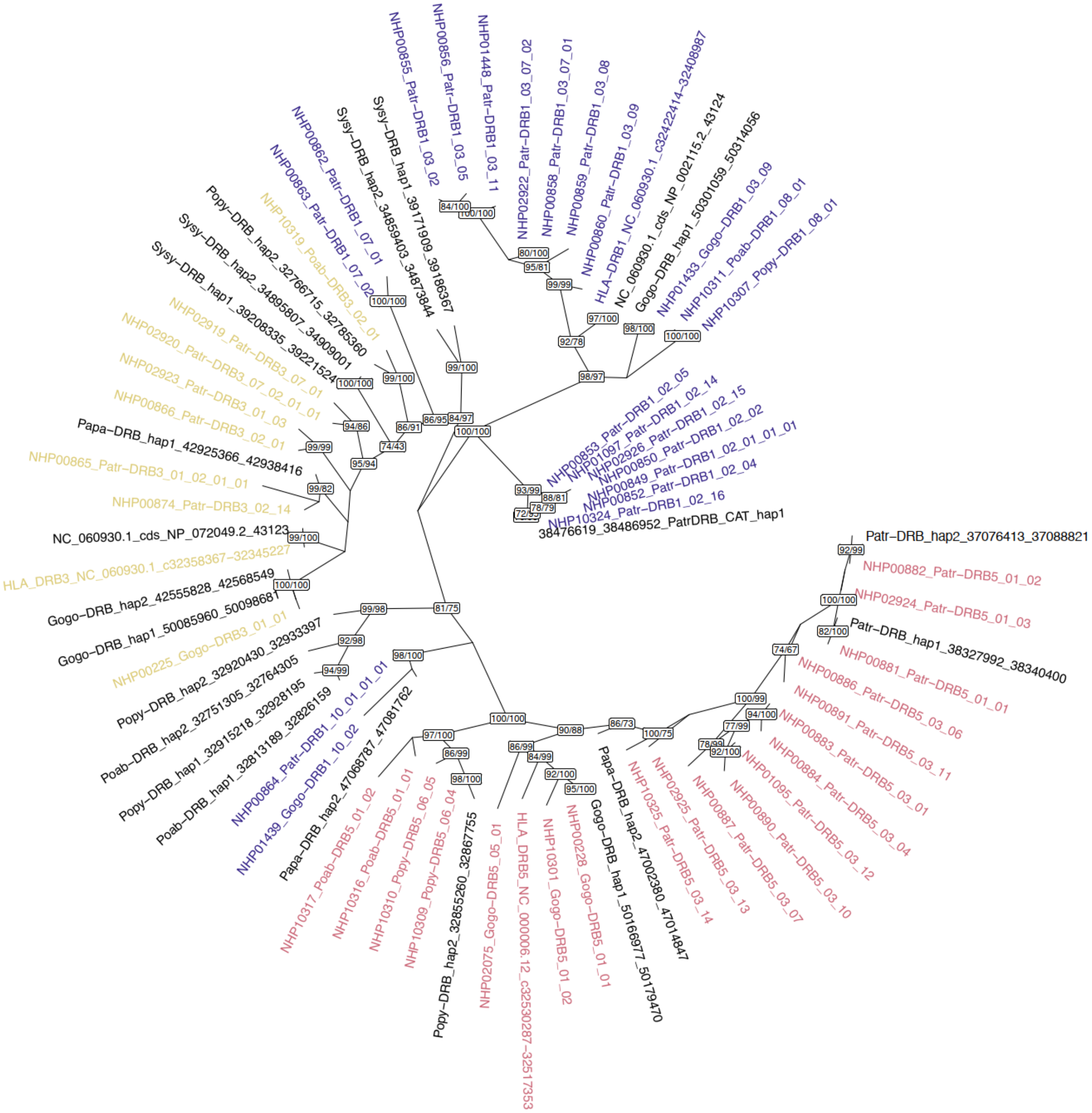

Supplementary Figure XI.34. Unrooted maximum likelihood phylogeny of MHC class II DRB genes. Phylogeny of coding sequence of human and ape MHC class II DRB loci from the six T2T assemblies, genomic sequence of species MHC class II DRB genes obtained from the IMGT MHC database, and annotated MHC class II genes from the human T2T-CHM13v2.0 genomic assembly. NHP gene names are abbreviated according to species (Patr-*Pan troglodytes*, Papa-*Pan paniscus*, Gogo-*Gorilla gorilla*, Popy-*Pongo pygmaeus*, Poab-*Pongo abelii*, Sysy-*Symphalangus syndactylus*). Clusters of orthologous loci are represented by unique colors. Newly annotated MHC II genes from this study are shown in black. IQ-TREE chose K2P+I+G4 as the best fitting model for codons 1 and 2, the HKY+F+I+G4 model for codon 3 following the BIC criterion.

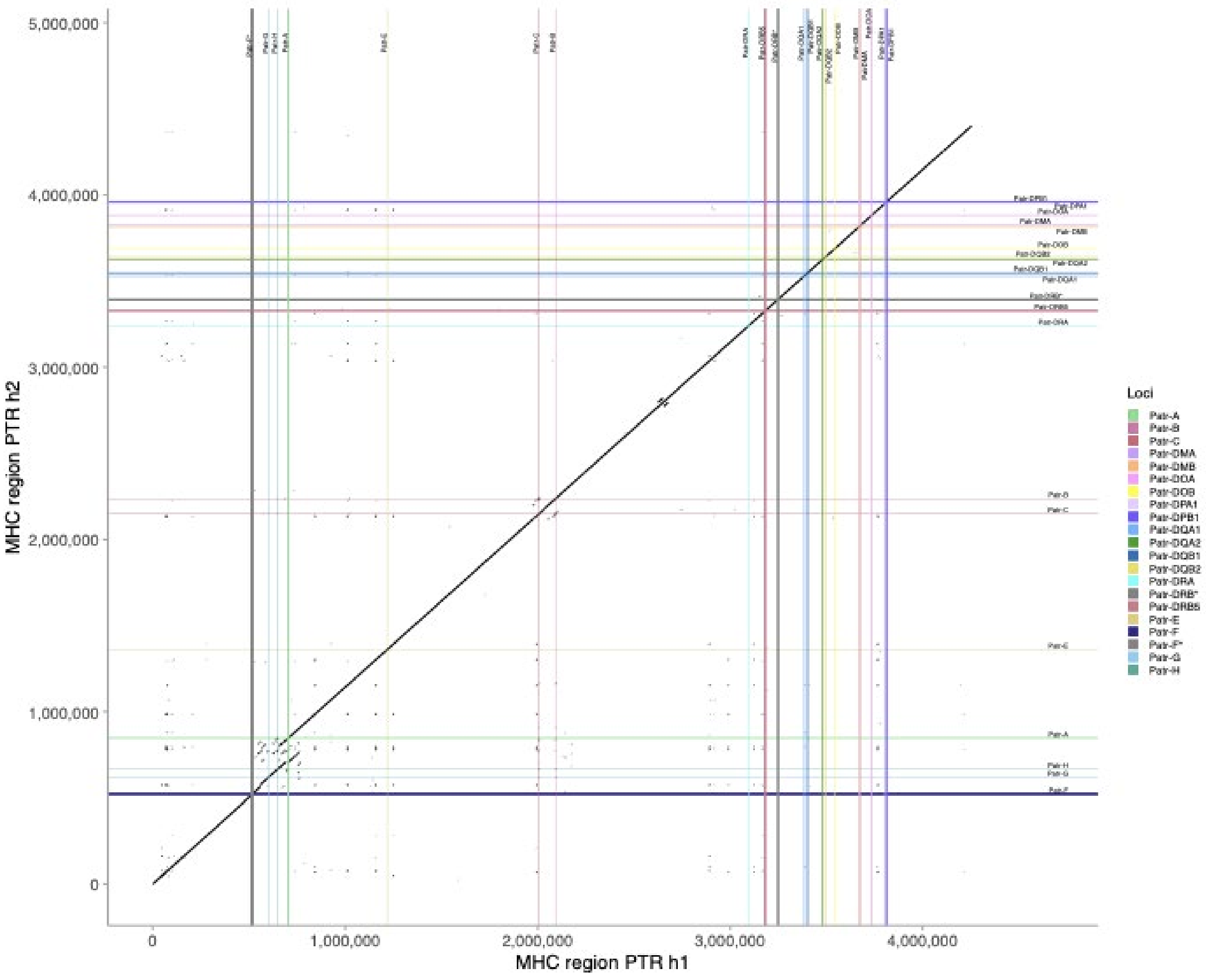

Supplementary Figure XI.35. Dot plot of PTR.h1/h2. Dot plots were made for the two MHC region haplotypes of *Pan troglodytes*. Locations of MHC coding genes and pseudogenes (labeled with *) for haplotypes 1 and 2 are labeled on the dot plot and also represented by horizontal lines (haplotype 1) and vertical (haplotype 2) lines. Unique colors representing each locus match those shown in **Fig. 3b-c** for a given MHC locus. The span of each horizontal and vertical line represents the length of a given gene.

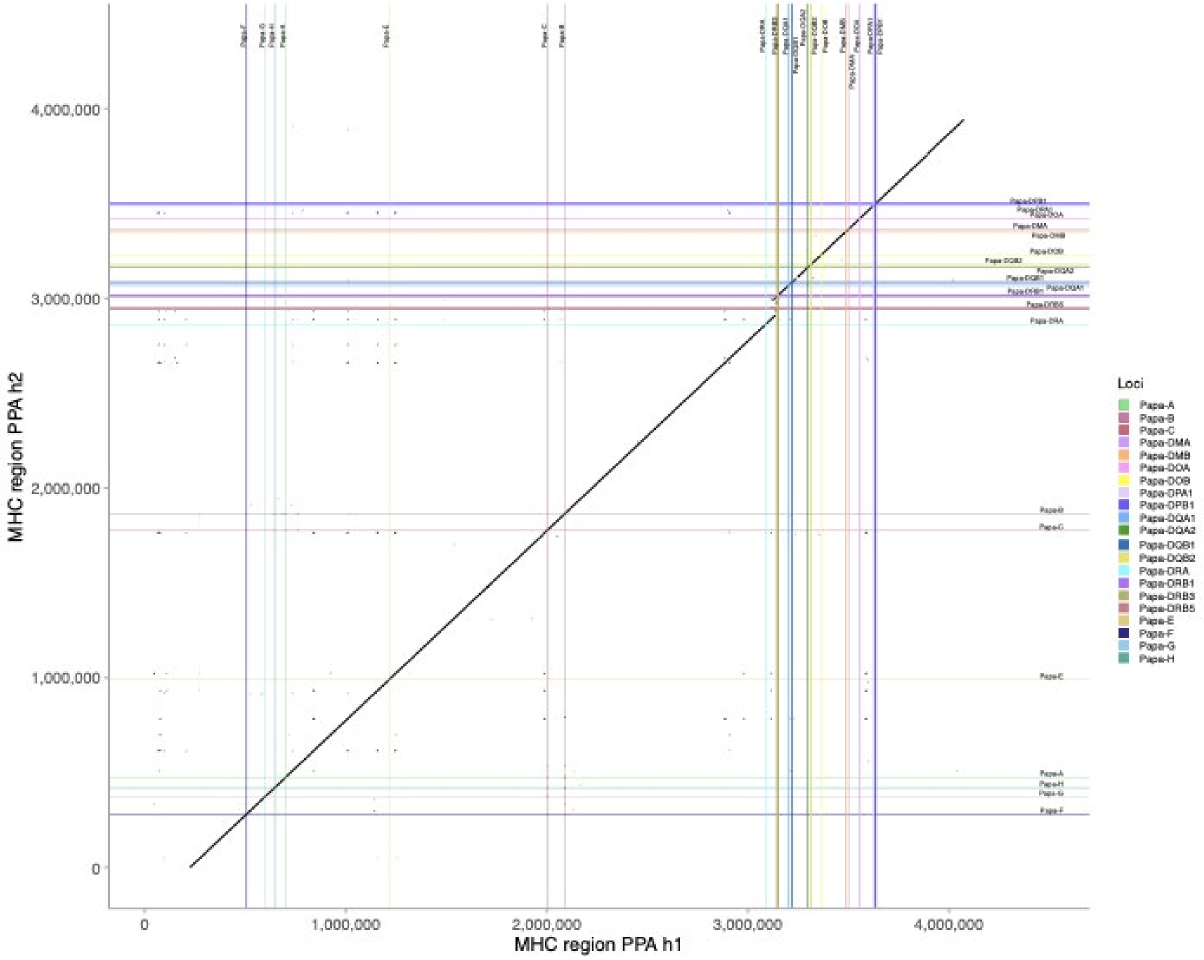

Supplementary Figure XI.36. Dot plot of PPA.h1/h2. Dot plots were made for the two MHC region haplotypes of *Pan paniscus*. Locations of MHC coding genes and pseudogenes (labeled with *) for haplotypes 1 and 2 are labeled on the dot plot and also represented by horizontal lines (haplotype 1) and vertical (haplotype 2) lines. Unique colors representing each locus match those shown in **Fig. 3b-c** for a given MHC locus. The span of each horizontal and vertical line represents the length of a given gene.

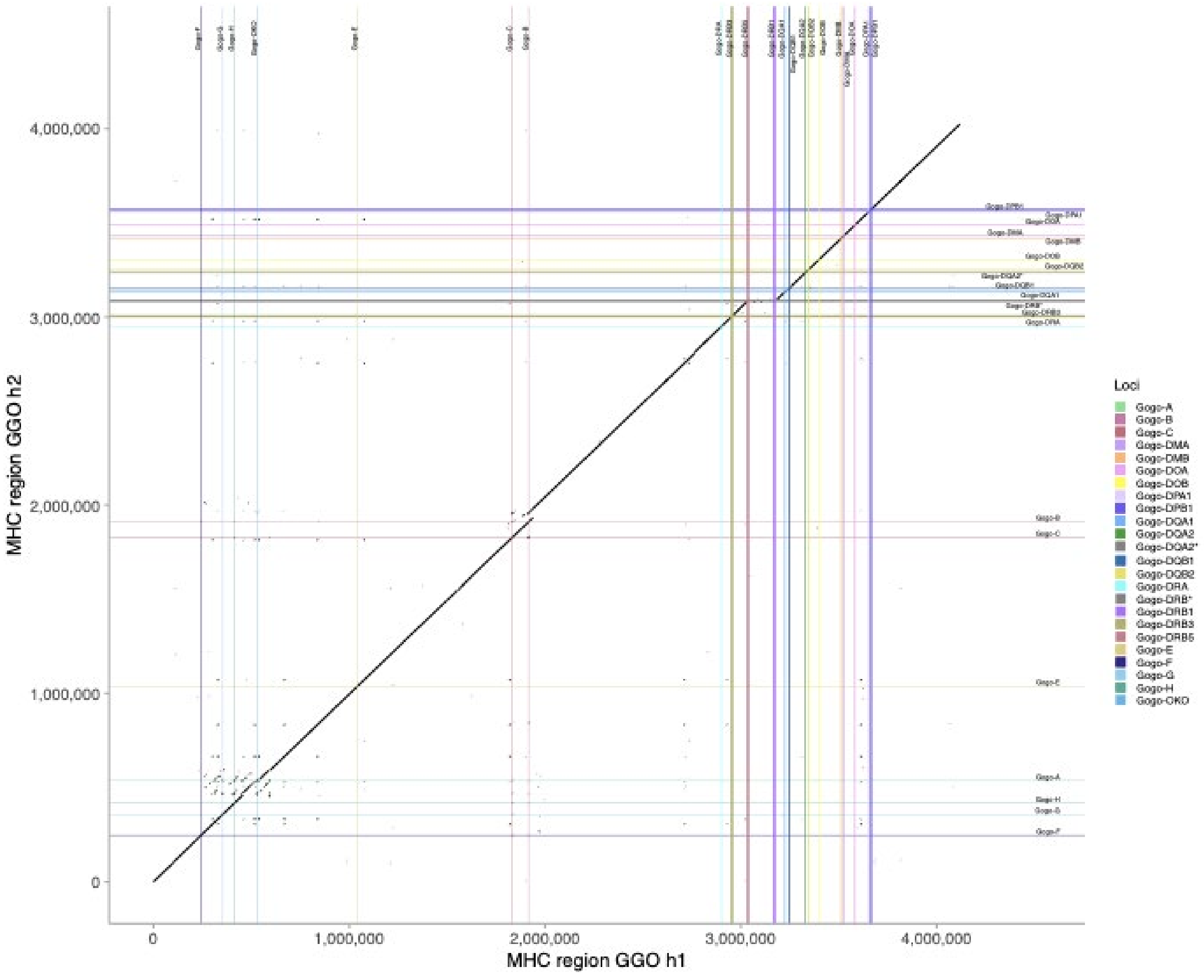

Supplementary Figure XI.37. Dot plot of GGO.h1/h2. Dot plots were made for the two MHC region haplotypes of *Gorilla gorilla*. Locations of MHC coding genes and pseudogenes (labeled with *) for haplotypes 1 and 2 are labeled on the dot plot and also represented by horizontal lines (haplotype 1) and vertical (haplotype 2) lines. Unique colors representing each locus match those shown in **Fig. 3b-c** for a given MHC locus. The span of each horizontal and vertical line represents the length of a given gene.

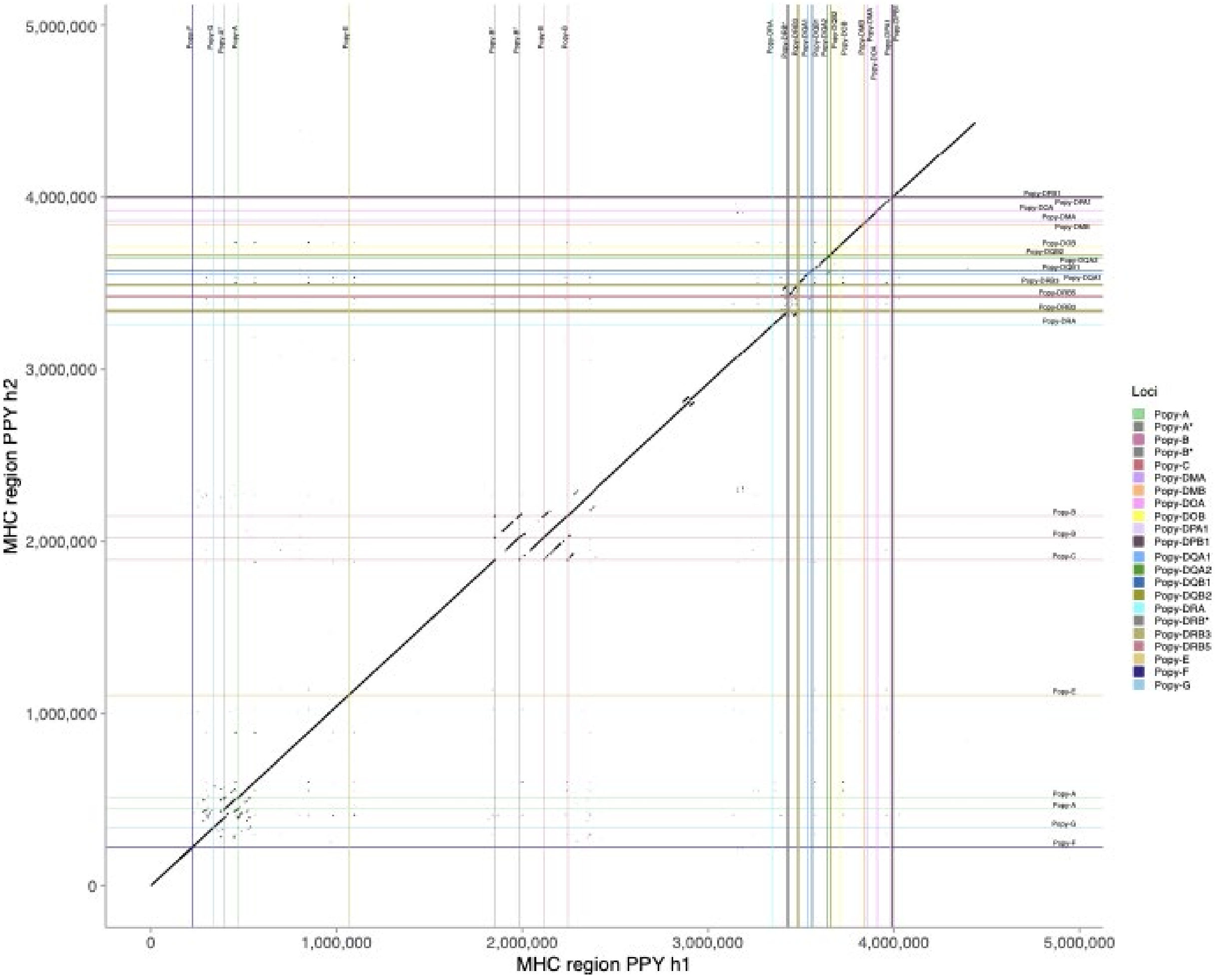

Supplementary Figure XI.38. Dot plot of PPY.h1/h2. Dot plots were made for the two MHC region haplotypes of *Pongo pygmaeus*. Locations of MHC coding genes and pseudogenes (labeled with *) for haplotypes 1 and 2 are labeled on the dot plot and also represented by horizontal lines (haplotype 1) and vertical (haplotype 2) lines. Unique colors representing each locus match those shown in **Fig. 3b-c** for a given MHC locus. The span of each horizontal and vertical line represents the length of a given gene.

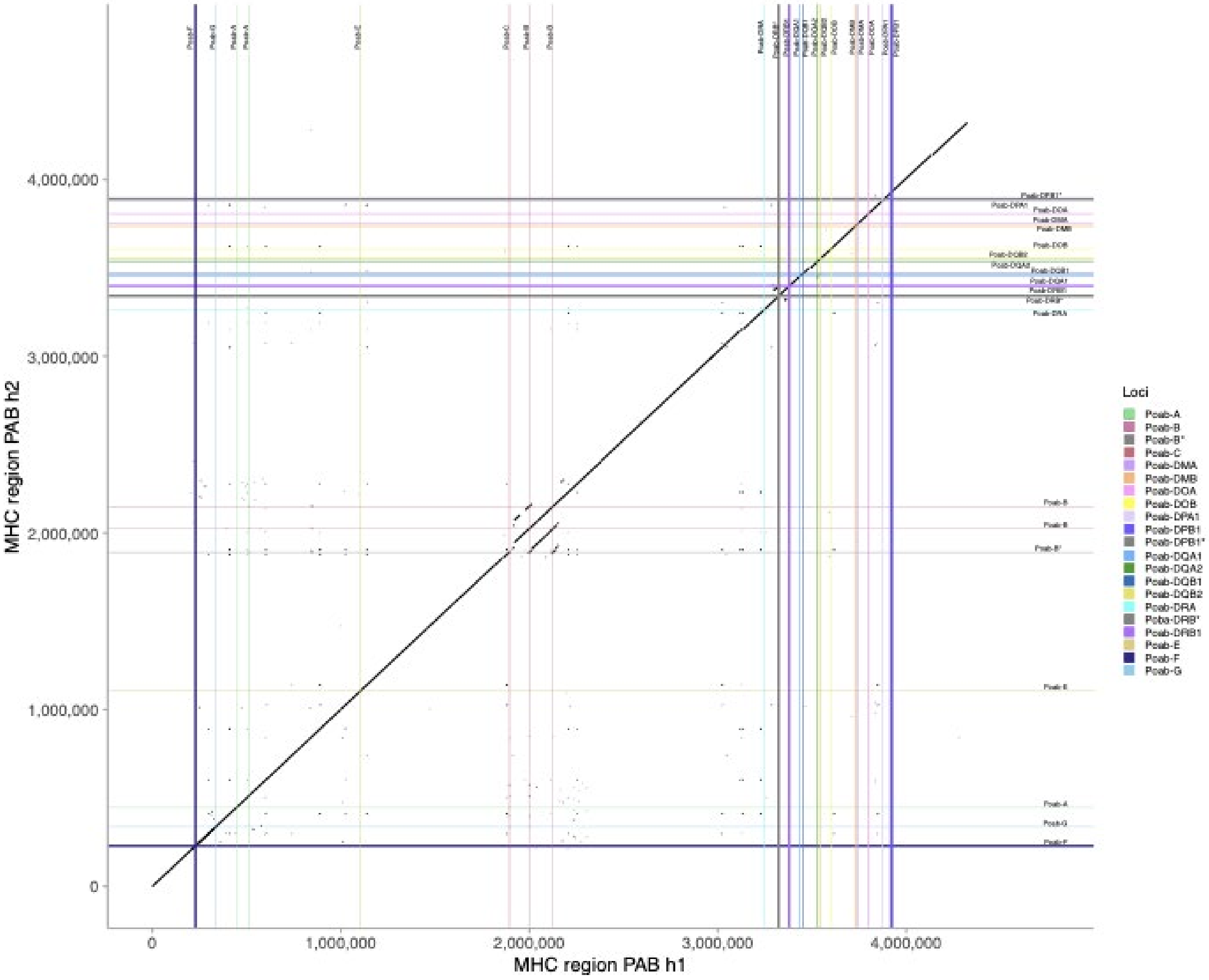

Supplementary Figure XI.39. Dot plot of PAB.h1/h2. Dot plots were made for the two MHC region haplotypes of *Pongo abelii*. Locations of MHC coding genes and pseudogenes (labeled with *) for haplotypes 1 and 2 are labeled on the dot plot and also represented by horizontal lines (haplotype 1) and vertical (haplotype 2) lines. Unique colors representing each locus match those shown in **Fig. 3b-c** for a given MHC locus. The span of each horizontal and vertical line represents the length of a given gene.

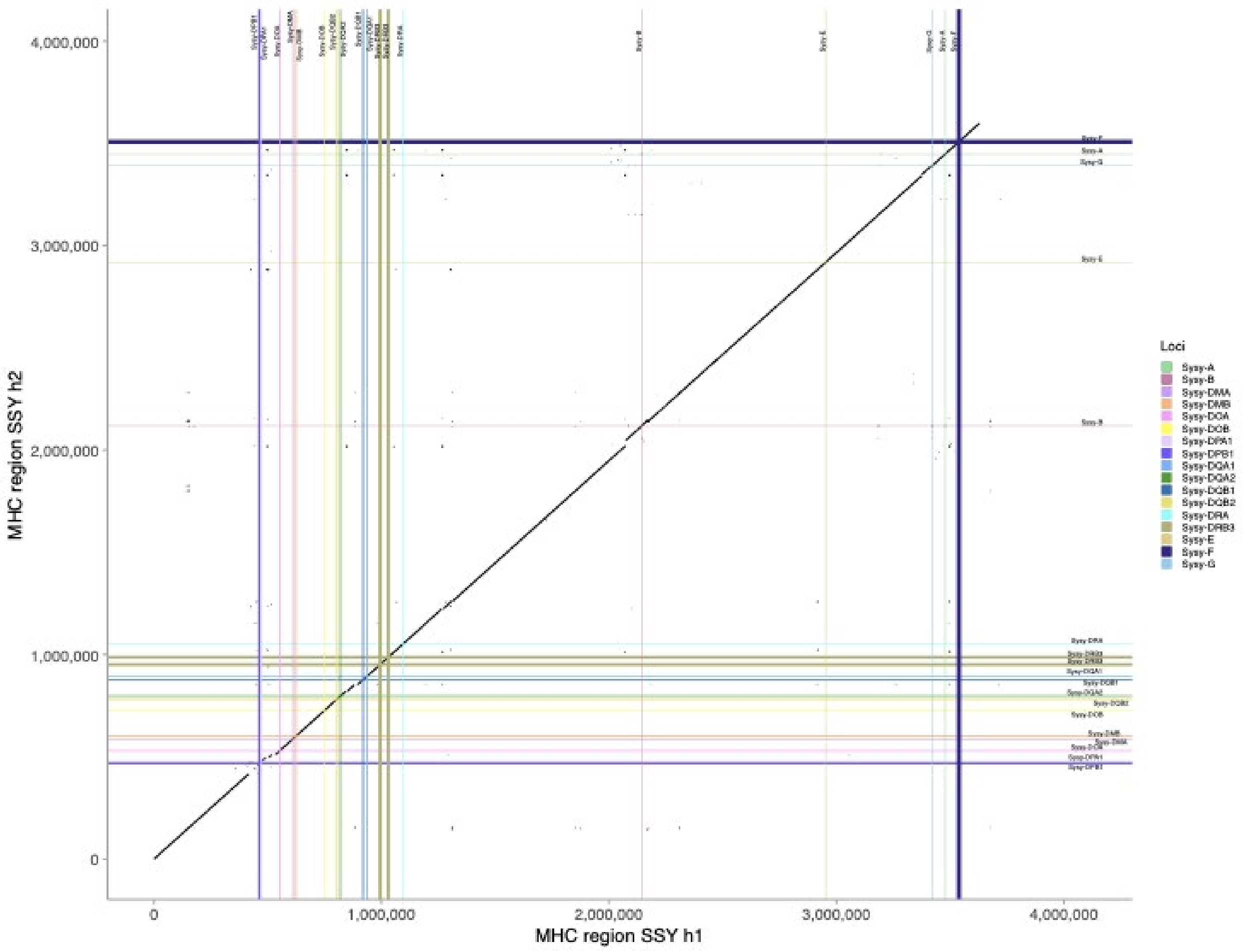

Supplementary Figure XI.40. Dot plot of SSY.h1/2. Dot plots were made for the two MHC region haplotypes of *Symphalangus syndactylus*. Locations of MHC coding genes and pseudogenes (labeled with *) for haplotypes 1 and 2 are labeled on the dot plot and also represented by horizontal lines (haplotype 1) and vertical (haplotype 2) lines. Unique colors representing each locus match those shown in **Fig. 3b-c** for a given MHC locus. The span of each horizontal and vertical line represents the length of a given gene.

Phylogenetic tree reconstruction of MHC region

We created 500 bp nonoverlapping bins from the 5.5 Mbp MHC locus from the human T2T sequence. These human 500 bp bins were pairwise aligned to each haplotype from the sequenced ape species using minimap2[70] to extract the homologous region from all the haplotypes. We also used the HG002 human sequence and did not include siamang in this analysis. We extracted homologous sequences in 500 bp increments and optimized local multiple sequence alignments (8,259 bins for 500 bp) using MAFFT and then concatenated to generate 5 kbp regions (953

bins); the regions with no missing sequences were used retaining 76% of position-wise bins. Maximum likelihood phylogenetic trees were constructed with IQ-TREE2[126] applying an optimized base substitution model and 1000 bootstrap replicates. To compare tree topologies we applied the Robinson-Foulds method, as implemented in the python ete3 package (compare() function)[127]. Each haplotype from the diploid assembly was considered separately, by selectively removing the external nodes pertaining to another haplotype in a tree. We performed hierarchical clustering taking trees and their pairwise distances into account to identify distinct topologies, through hclust and cuttree functions in R. We identified three regions that are enriched by 5 kbp bins with discordant tree topologies (4-19) by merging discordant bins that are within 500 kbp distance from each other (**Supplementary Table XI.59**). For the divergence time estimates, we narrow down to >50 kbp regions after merging discordant 500 bp bins within 10 kbp distance to identify four regions. From these four regions, we estimated the coalescence time. To estimate within species variation, we repeated the analysis after adding more genomes from Mao et al., 2024[115], and also repeating the procedure of defining four subregions by merging them to the nearest 10 kbp of discordant bins. The divergence trees were calibrated by Macaque–Human split in all the four trees and Siamang–Human split in the second tree (II) (**Supplementary Fig. XI.41**). We only used Siamang–Human split once in a single tree, as it was the only topology where Siamang (Jambi_SSY) was the outgroup to African great apes and orangutans.

Following are the 19 topologies constructed during the analysis:

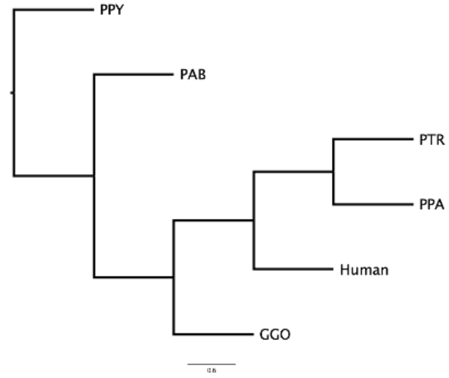

1

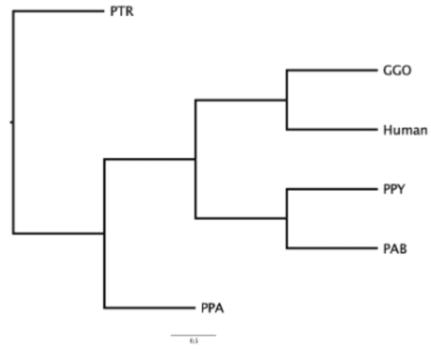

2

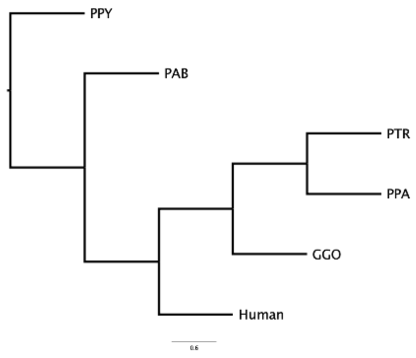

3

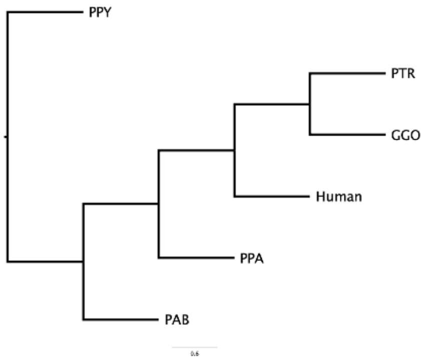

4

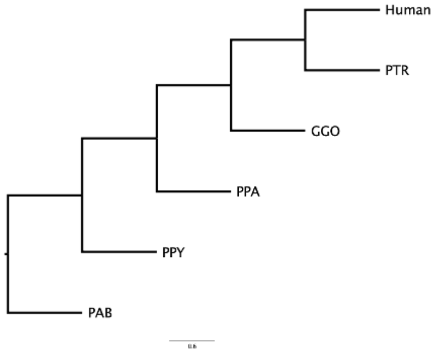

5

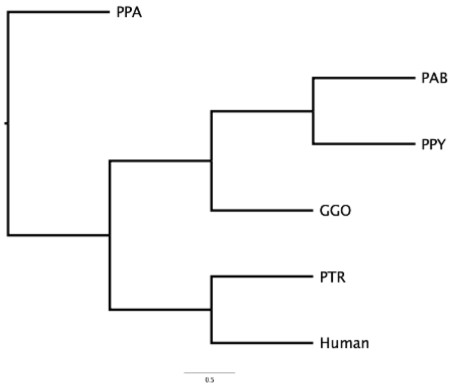

6

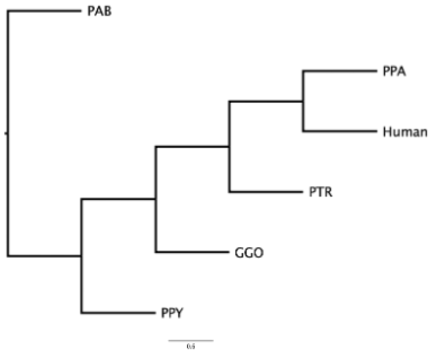

7

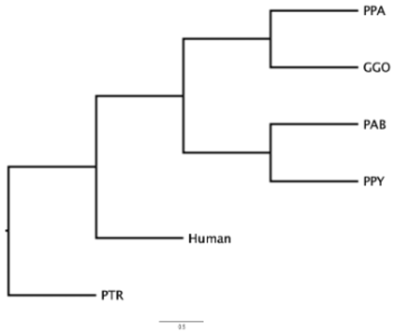

8

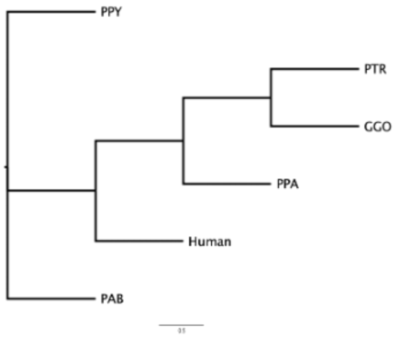

9

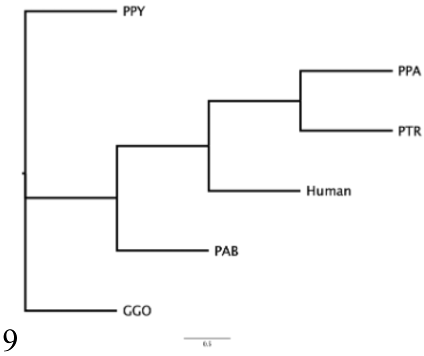

10

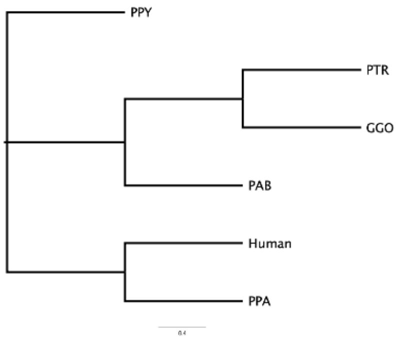

11

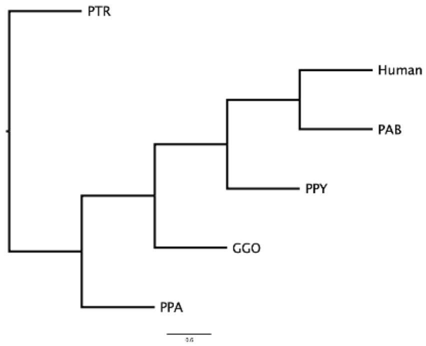

12

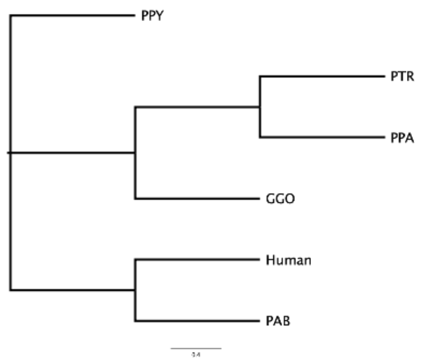

13

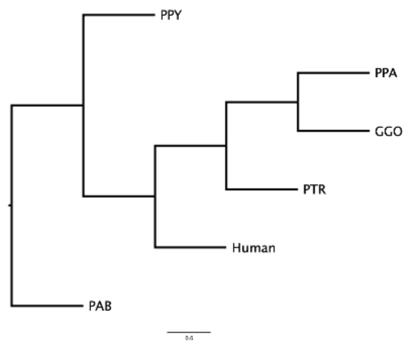

14

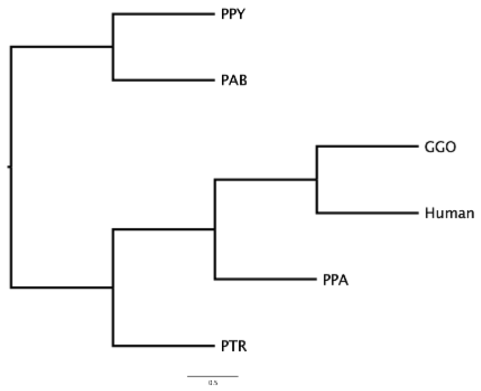

15

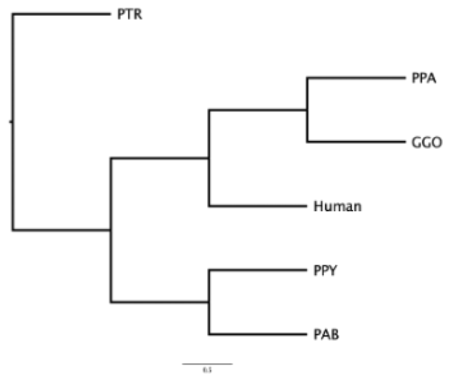

16

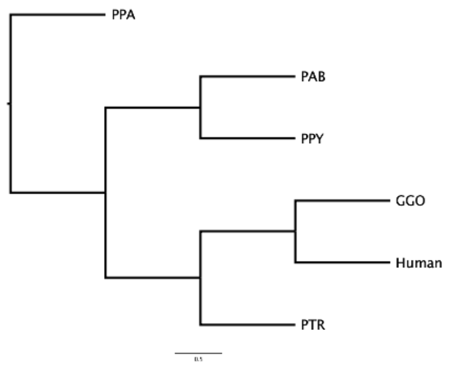

17

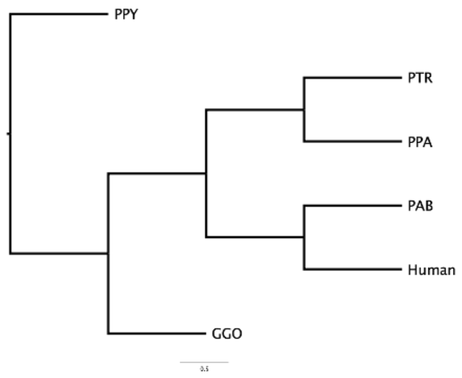

18

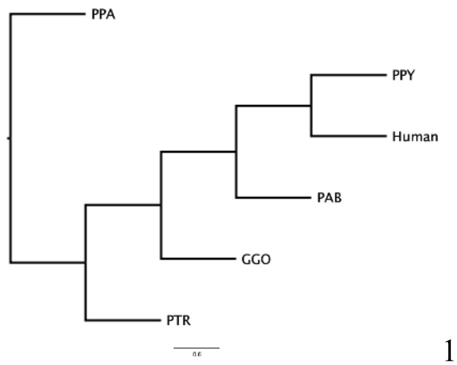

19

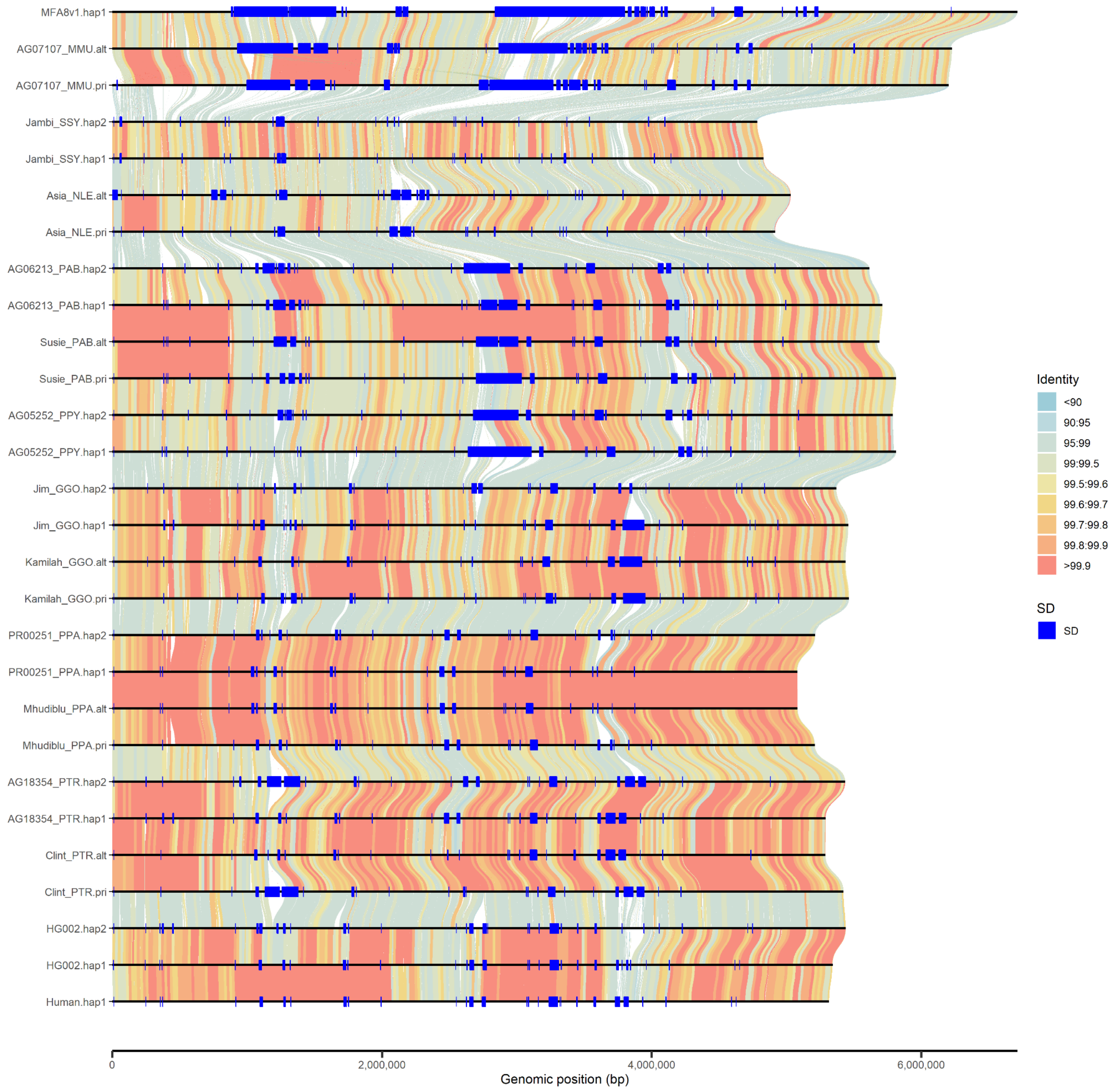

Supplementary Figure XI.41. Alignment of MHC regions with the addition of genomes from previous studies[115].

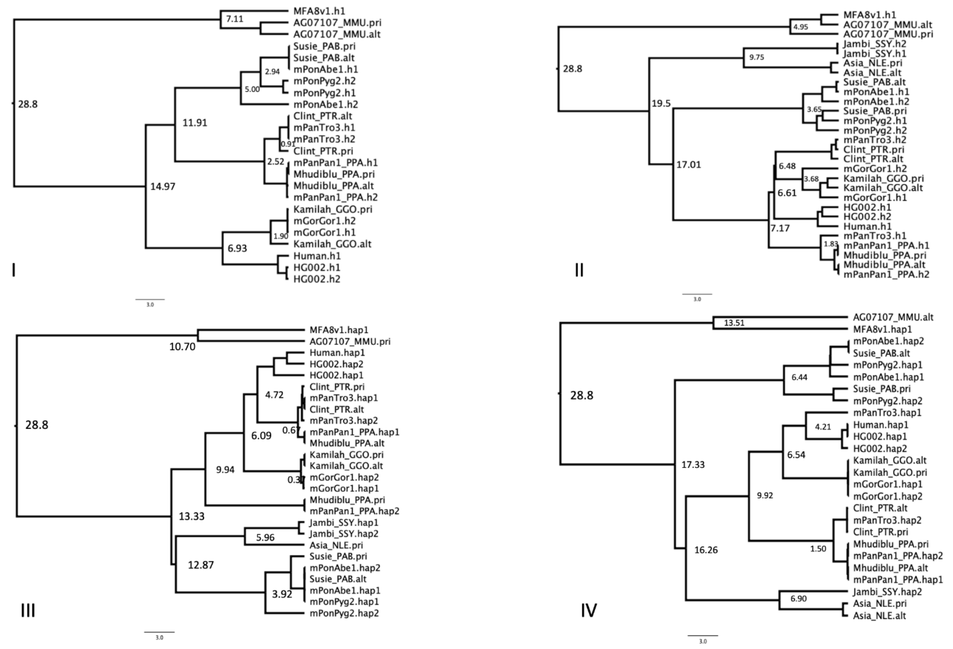

Supplementary Figure XI.42. The tree topology and divergence time estimates of the variable regions (I, II, III and IV) detected in **Extended Fig. 3**, after addition of genomes[115].

Summary of results

We also completely assembled and annotated the 4-5 Mbp region corresponding to 12 ape haplotypes for the MHC loci—a highly polymorphic region among mammals[128] encoding diverse cell surface proteins crucial for antigen presentation, adaptive immunity[129], and in humans disproportionately implicated in disease via genome-wide association[130]. Comparative sequence analyses confirm extraordinary sequence divergence and structural variation, including expansions and contractions associated with MHC genes (**Extended Fig. 3**). Overall, MHC class I genes reveal more extensive structural variation within and among the apes than MHC class II genes (**Extended Fig. 3a-b**, **Supplementary Fig. XI.33-34**). For instance, siamang carries a distinct Sysy-B locus but lacks a distinct Sysy-C locus (**Extended Fig. 3a-b**). Furthermore, an inversion occurred between the MHC-G and MHC-A loci after the divergence of the great apes and humans from the siamang. The MHC I gene content and organization is identical across human, bonobo, and chimpanzee, but we observe relatively high levels of interspecific and intraspecific variability in the other species, including additional genes (e.g., Gogo-OKO (**Extended Fig. 3**), which is related but distinct from Gogo-A[128]). Furthermore, in orangutans, we observed expansion of MHC-A and MHC-B in both the Bornean and Sumatran lineages (**Extended Fig. 3c**). Unlike human, chimpanzee, gorilla, and bonobo, MHC-B is duplicated in both haplotypes of the two orangutan species. Both the Sumatran and Bornean orangutan species

possessed a duplicated MHC-A locus on one haplotype and a single MHC-A locus on the other haplotype. Human and the other great ape species possessed a single MHC-A locus on both haplotypes. Surprisingly, both orangutan species lack the MHC-C locus on one haplotype and have the MHC-C locus on the other haplotype, thus revealing another case of copy number variation of an MHC class I gene in these species. All ape and human species possessed the same identical set of MHC II loci, but there was copy number variation at the interspecific and intra-individual level in the DRB locus among all studied species (**Extended Fig. 3a-b**). We also observed two cases where an MHC locus was present as a functional gene on one haplotype and as a pseudogene on the other haplotype, as exemplified by the Gogo-DQA2 locus in gorilla and the Poab-DPB locus in Sumatran orangutan.

XII. Evolutionary rearrangements and inversion characterization

Contributing authors:

Francesca Antonacci, DongAhn Yoo, Luciana de Gennaro, David Porubsky, Mario Ventura

Methods

SV calling

We used the SYRI (v1.6.3)[131] and PAV (v2.3.2)[94] pipelines to identify SVs (>50 bp), including inversions, in six diploid genomes. This was done using the human T2T genome (T2T-CHM13v2.0) and Bornean orangutan's primary haplotype as the reference to check for effect of reference bias. SYRI inversions in general represented larger inversions, and the PAV-called inversions were added after merging inversion calls that share the same breakpoints (reciprocal coverage of 80%). Alignment artefacts (less than 50% supported by alignment or 1 Mbp of alignment support-minimap2) were further filtered to the merged set of inversion calls by SYRI and PAV. For the comparison to previous studies, inversions with size exceeding 10 kbp were used.

Validation against previous studies

We rigorously compared our identified inversions against an array of previously characterized large-scale inversions from cytogenetic studies and smaller inversions validated through single-cell strand-seq, fluorescence in situ hybridization (FISH), BAC clone sequencing, BAC-end mapping, and PCR[24,30,98,132-152]. This was done by comparing the inversion breakpoints lift overed onto hg38.

Summary of results

Near-T2T ape genome assemblies provide a more comprehensive map of inversions across the ape phylogenetic lineage. We identified a total of 1,175 inversion variants larger than 10 kbp as well as one large-scale chromosome fusion and one translocation (**Supplementary Fig. XII.43**) across six species; the breakdown of identified inversions is as follows: 188 in bonobo, 171 in chimpanzee, 199 in gorilla, 180 in Bornean orangutan, 180 in Sumatran orangutan, and 257 in siamang. Compared with the previously documented inversions, we find 558 represent novel discoveries. Focusing on the larger ones (>1 Mbp), we find that 160 were previously documented inversions and 29 are novel. Furthermore, we find 27 erroneous calls, 23 of which coming from siamang We resolved 40 breakpoints of the Yunis and Prakash large chromosome inversions; all

breakpoints differ by a maximum of ~700 kbp from previous cytogenetic mapping (**Supplementary Table XII.66**). In six instances, boundaries involving the centromere or telomere have been resolved for the first time, and in two instances the T2T genome assemblies revealed a more complex organization than reported by cytogenetics (orangutan chromosomes 2 and 19).

Filtering out the false inversion calls of 1,175 inversions larger than 10 kbp, 1,140 inversion calls remain. Among these, 617 are previously known and 522 are novel. Additionally, our complete assemblies allowed us to refine the breakpoints of 85/617 known inversions and revealed that one event (Jim_GGO.hap1.INVTR_4), which was previously classified as an inversion in gorilla[132] is an inverted transposition. The transposition was confirmed with two FISH experiments on metaphase chromosomes in one gorilla individual (**Fig. 3b**). Out of the 1,140 inversions, 632 are homozygous (found in both haplotypes of the same individual), while 508 are heterozygous and potentially polymorphic in the population. Looking for genes mapping at the breakpoints, we found that 416 inversions have genes mapping at least at one breakpoint, while 724 events are completely devoid of genes at their breakpoints.

Of the 1,140 inversions, 529 have SDs at both breakpoints (46%), with 78% (412 out of 529) having SDs in inverted orientation. Additionally, 195 inversions (17%) have SDs at only one breakpoint, and 416 inversions are devoid of SDs at both breakpoints.

Focusing on just the 632 homozygous inversions, 197 are novel and 435 are previously known. The 197 novel homozygous inversions have an average size of 223 kbp and mostly map within pericentromeric regions and/or SDs. In particular, 72% (141/197) have annotated human SDs at one or both ends of the inversions, versus 43% (186/435) of the known homozygous inversions, which could explain why they were not detected in previous studies. Of the novel homozygous inversions, 78 have annotated genes mapping at least one of the breakpoints with 119 devoid of protein-coding genes (**Supplementary Table XII.67**).

Investigating 100 kbp flanking regions of inversions, we find that inversion breakpoints are typically enriched for T2T human SDs ($p<0.001$) compared to random genomic regions by 4.6-fold (flanking of the randomly shuffled inversions; **Supplementary Fig. XII.44**). We also observed that the enrichment was stronger for inverted duplications ($p<0.001$) with 6.2-fold more overlap compared to null genomic regions. Stronger enrichment was observed restricting the test to larger inversions (>50 kbp).

Of the inversions without SDs, 289 have other types of repeat elements mapping at both breakpoints and 35 have repeats at only one breakpoint, while 92 are completely devoid of any repeats.

Among the 523 novel inversions, we identified 78 in chimpanzee, 99 in bonobo, 96 in gorilla, 71 in Sumatran orangutan, 68 in Bornean orangutan, and 111 in siamang. All the novel inversions are smaller than 5 Mbp, just below the limit of cytogenetic resolution. Nearly half of these novel inversions (227 out of 523) have SDs at one or both breakpoints when mapped to the human

reference genome. This complexity may have contributed to these inversions being overlooked in previous studies. Additionally, 325 out of the 523 novel inversions are heterozygous and potentially polymorphic, which may account for their absence in earlier analyses performed on different individuals (e.g., Porubsky et al.[132]). For 160 novel inversions, genes mapped at one or both breakpoints, while no genes were detected at the breakpoints of the remaining 363 inversions.

Based on the parsimony of homozygous inversions among the apes (comparing the breakpoints with 80% reciprocal overlap), we assigned 64-90% of inversion events (64% in bonobo and up to 90% in siamang) onto phylogeny (**Supplementary Fig. XII.45**) and predict the remaining inversions to be recurrent. The number of inversions were particularly less in certain lineages, including HSA which showed fivefold less than expected number considering the branch length. Among the phylogeny-classified inversions, we find a total of 133 new inversions, with the highest number gained from siamang lineage (n=44). We also find the number of inversion events correlated with phylogeny ($R2 = 0.77$).

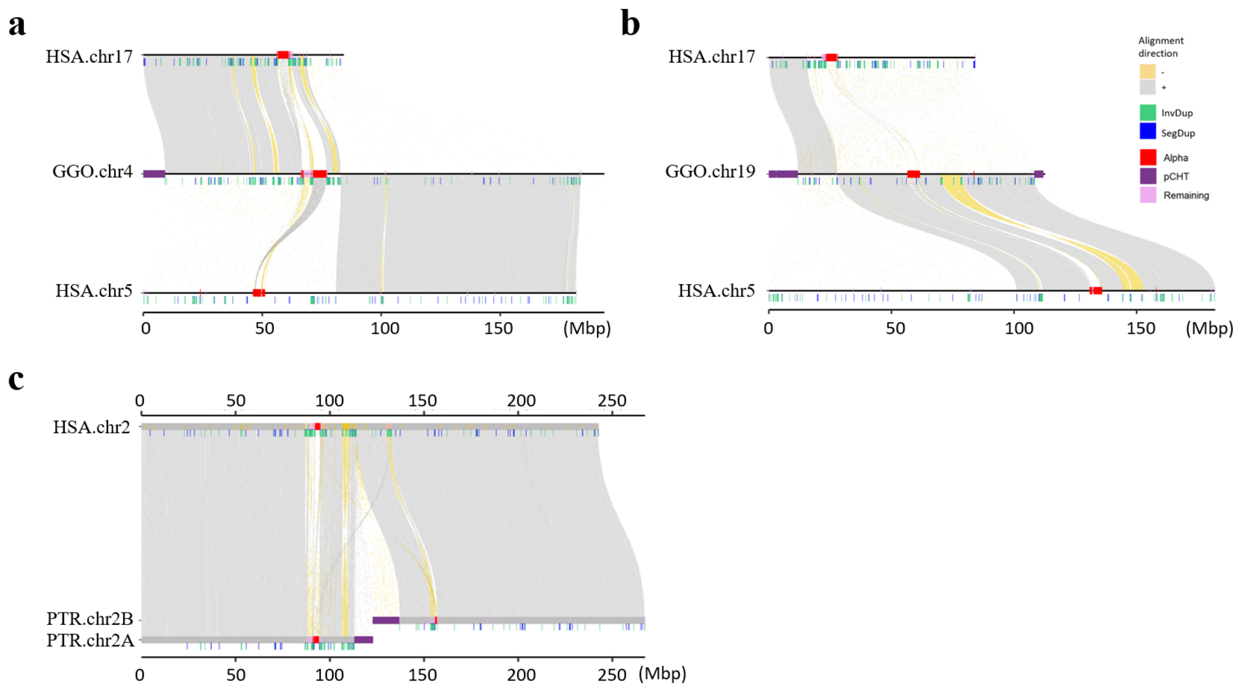

Supplementary Figure XII.43. Chr4/19 translocation in gorilla and chr2 fusion in human. Alignment view of gorilla chr4 (a) and chr19 (b). c) Alignment of human chr2 fusion.

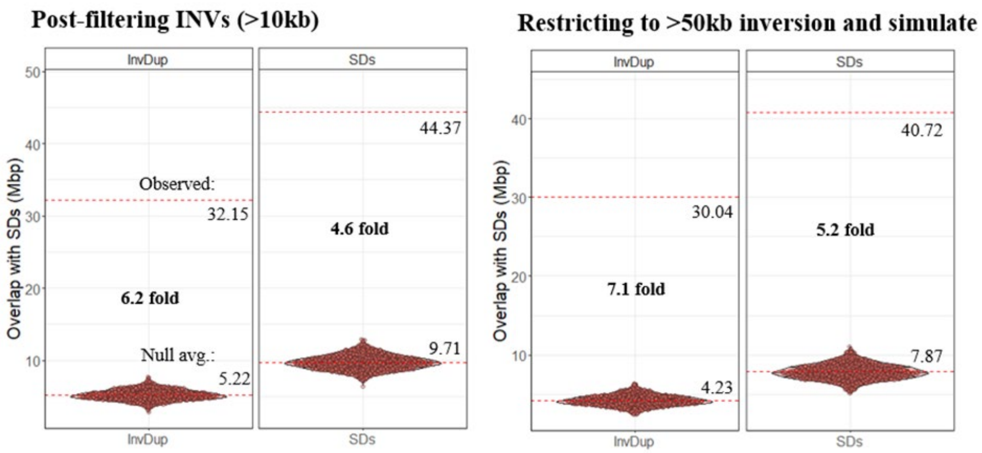

Supplementary Figure XII.44. Enrichment of segmental duplications (SDs) at the breakpoints (100 kbp flanking) of inversions. Left shows the enrichment for the inversions (INVs) in size >10 kbp, while the right shows the enrichment for >50 kbp inversions.

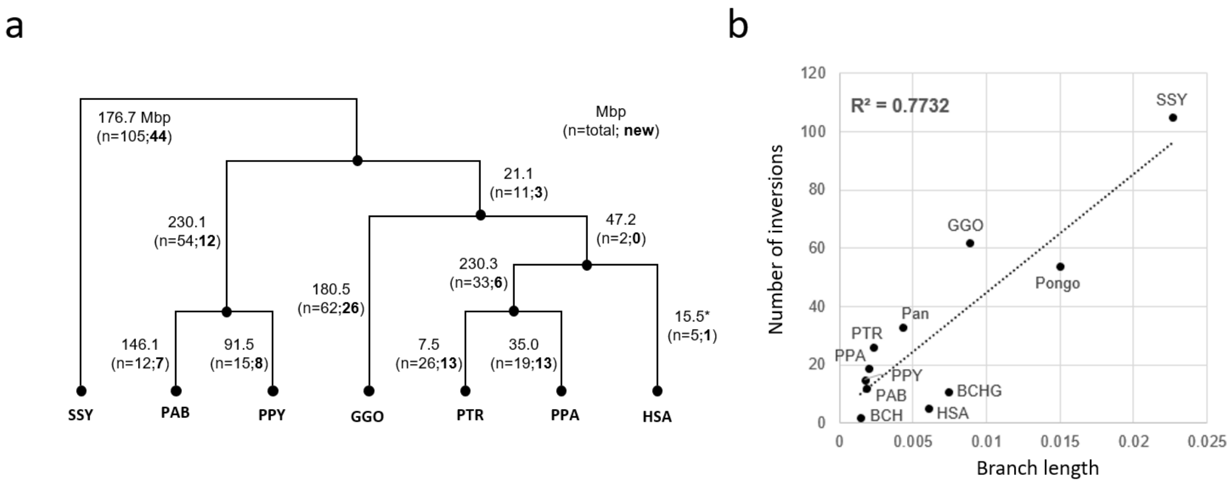

Supplementary Figure XII.45. Inversion assignment into phylogeny and correlation of inversions with phylogenetic branch lengths. Indicated by "*" are the human-specific inversion calls which are found in inverted orientation in all other apes compared to human.

XIII. AQER

Contributing authors:

Yanting Luo, Manolis Kellis, Riley J. Mangan, Sarah A. Zhao, Chul Lee, Youngho Lee, Erich D. Jarvis, Craig B. Lowe

Methods

We defined highly divergent regions in four great ape lineages by calculating the number of mutations that likely occurred in 500 bp regions between inferred ancestral nodes and extant genomes. The four lineages we analyzed were the inferred human–chimpanzee ancestor to the human (hs1), chimpanzee (primary haplotype), and bonobo (primary haplotype) genomes, as well as the inferred human–gorilla ancestor to the gorilla (primary haplotype) genome. We term these regions ancestor quickly evolved regions (AQERs), with the individual sets being (human) HAQERs, chimp-AQERs, bonobo-AQERs, and gorilla-AQERs. The identification of AQERs is not limited to conserved regions, but rather screens the entire genome, including elements that descended from previously neutral regions. This distinguishes HAQERs from many other searches for the genetic underpinnings of uniquely human traits, which focused on human-specific divergence in conserved genomic regions and reflect modifications of existing functional elements. AQERs were identified in three steps. First, we aligned both haplotypes from the nearly complete great ape assemblies (https://github.com/marbl/Primates) to the T2T human assembly (hs1) with LASTZ[23]. We chained the local alignments together with the utilities used by the UCSC Genome Browser group[153], but with stricter parameters[154]. We used stricter scoring and filtering parameters because our method is sensitive to false-positive alignments; misalignments can often appear as regions of rapid divergence. We filtered these chained alignments to retain alignment fragments scoring greater than 60,000 (approximately 20 kbp of matches). We then used these single-coverage chains to generate a multi-species genome-wide alignment with MultiZ[155]. For each of the four lineages being analyzed for highly divergent regions, we inferred the sequence of the ancestral node in a conservative fashion. Specifically, we used the gene annotations made by NCBI RefSeq for hs1 (https://hgdownload.soe.ucsc.edu/goldenPath/hs1/bigZips/genes/hs1.ncbiRefSeq.gtf.gz) to extract fourfold degenerate codon sites and estimate branch lengths for a fixed-topology tree using a Jukes-Cantor model of evolution by maximum likelihood. For each position in the alignment, a base was determined to be present in the ancestral node according to the tree, i.e., a base is present in at least two species on two independent lineages connected to the ancestral node (lineages to parent node and two child nodes). This is equivalent to treating aligned bases as having a common origin. For bases that are present at a node, we reconstructed the probabilities of A, C, G, and T in the ancestral node using the estimated branch lengths and the value of the base position in extant species[156]. To assign a single base to the ancestral node from these four probabilities, positions were assigned the value observed in the extant species unless the probabilities of the other three base values summed to greater than 0.8. This ensures that we

only count substitutions where we are confident of the change occurring on the lineage being analyzed. Finally, the number of mutations that separate the inferred ancestral node and the extant genome was counted by sliding windows of 500 bp. Windows with significantly more divergences than expected were identified as HAQERs, chimp-AQERs, bonobo-AQERs, and gorilla-AQERs. To conservatively estimate significance, we use the fastest divergence rate in a 10 Mbp window of the genome as the estimate of the expected divergence rate when defining HAQERs. For the other lineages, we scale this conservative divergence rate relative to the ratio of branch lengths between the human branch and the branch length of the other lineages from fourfold degenerate sites for: chimp-AQERs, bonobo-AQERs, and gorilla-AQERs. The p-value of obtaining at least the number of divergences found in each 500 bp window along the assembly was calculated using a binomial distribution, where the number of trials is 500, and the probability of success is the expected divergence rate. This raw p-value was adjusted using the Benjamini-Hochberg procedure by counting the number of 500 bp windows in the genome and ranking their raw p-values. Windows with adjusted p-values of less than 3e-7 were identified as AQERs, which is equivalent to at least 29 mutations in a 500 bp window for most lineages analyzed (i.e., HAQERs, chimp-AQERs, and bonobo-AQERs), and at least 34 mutations in a 500 bp window for gorilla-AQERs, due to the longer phylogenetic branch from the human–gorilla ancestor to gorilla. We used the maximum rate of evolution (mutations/bp) in a 10 Mbp window as the expected rate of evolution. If this window size were to decrease, to say 1 Mbp, the maximum rate (mutations/bp) will increase since the fastest 1 Mbp window must be at least as fast as the fastest 10 Mbp window. The goal is to identify forces shaping the divergence of short genomic regions (e.g., 500 bp) that is unique compared to the forces shaping large genomic regions (i.e., 10 Mbp). Decreasing from a 10 Mbp window size to 5 Mbp, 1 Mbp, or 500 kb would have increased the threshold for calling HAQERs from 29 mutations to 31, 38, or 39. Additionally, we ensured that chimp-AQERs, bonobo-AQERs, and gorilla-AQERs represent continuous sections of both the human and NHP genomes. Mangan, R. J. et al. offers a more detailed description of the methods we used[20]. We generated additional HAQER sets at different levels of spatial resolution by changing the window size of our sliding window-based HAQER ascertainment program. We generated additional HAQER sets at window sizes of 25, 50, 100, 200, 300, 400, 500, 750, 1k, 2k, 5k, 10k, and 20k bp. There are varying levels of statistical power across these sets to detect a deviation from a set rate of divergence. The larger windows have more statistical power and greater sensitivity to detect a deviation from the set rate of divergence. Furthermore, we identified a repeat-free subset of 500 bp window size T2T HAQERs, which excludes HAQERs that overlap centromeres/satellites, SDs, simple repeats, or RepeatMasker annotations. Complementary to the repeat-free subset of HAQERs is the repeat subset of HAQERs, which consists of HAQERs that have any overlaps with these repeat annotations.

To analyze the relationship between AQER elements and regulatory elements, we calculated binomial-based overlap enrichments between AQERs and chromatin state annotations derived from the Roadmap Epigenomics Consortium[157,158]. We calculated similar enrichments against RepeatMasker and gene annotations developed for each assembly. Finally, to analyze the relationship between HAQERs and regions of ILS, we calculated one-sided binomial-based enrichments between HAQERs and regions of the human genome with a posterior probability of at least 0.6 for each TRAILS hidden state.

For *ADCYAP1*, to assemble methylation profiles of six ape species for downstream analyses of vocal-learning related genes, public raw PacBio long reads generated with 5-base HiFi sequencing with kinetics and methylation tags were downloaded from Human Pangenome Reference Consortium (HPRC, https://humanpangenome.org/data/) for human and GenomeArk (https://www.genomeark.org/t2t-all/) for chimpanzee, gorilla, Bornean orangutan, Sumatran orangutan, and siamang gibbon. First, all reads were merged into a single file for each species by SAMtools version 1.16.1 using SAMtools merge. Then, the reads were aligned to T2T genome assemblies for each of the respective species by pbmm2 version 1.10.0 using pbmm2 align. Aligned reads were sorted and indexed by SAMtools using SAMtools sort and SAMtools index, respectively. Pre-included methylation tags of the raw reads were called using align_bam_to_cpg_scores.py provided pb-CpG-tools version 2.3.2 (https://github.com/PacificBiosciences/pb-CpG-tools). Then, modification probability of all CpGs of genomes was calculated based on tags of reads mapped to the corresponding sites and adjusted based on overall distributions of modification scores using a machine-learning model pileup_calling_model.v1.tflite provided by pb-CpG-tools. After pileup of the modification probabilities, we classified CpGs with scores over 75% as hypermethylated, between 25-75% as heteromethylated, and under 25% as hypomethylated.

Summary of results

Nearly complete great ape assemblies resolve multi-scale divergence landscapes across human and great ape genomes

We investigated how the availability of nearly complete genome assemblies for humans and great apes improves the identification of highly divergent regions within the great apes. Previous searches for highly divergent regions on the human lineage in conserved regions have revealed functional modifications in regulatory elements[159] and divergent elements in unconstrained sequence are associated with *de novo* functional elements[154].

We identified 13,128 AQERs across four great ape lineages (3,268 HAQERs, 4,001 chimp-AQERs, 4,231 bonobo-AQERs, and 1,628 gorilla-AQERs) (**Supplementary Fig. XIII.46a, Methods**). There are 920 gapped HAQERs that overlap any T2T HAQERs lifted to the gapped assembly (**Supplementary Fig. XIII.46b**). It is worth noting that there are a number of differences between the underlying data used to identify the previously defined set of HAQERs and the set of T2T HAQERs beyond gapped and T2T assemblies. An important difference is that the T2T human assembly represents a different set of human haplotypes, often with different ancestry, from those used in hg38. Therefore, the 661 HAQERs that were not re-identified have some divergent mutations that are not fixed in humans, such that these same regions do not cross the significance threshold for calling a HAQER when using another sampling of human haplotypes. The 2,348 T2T HAQERs that were not previously discovered, are a mixture of those that do not replicate broadly across human haplotypes (similar to the 661) and those in regions of the genome that could not previously be analyzed due to assembly gaps in humans and/or other great apes.

Particular classes of repetitive elements are enriched or depleted for these highly diverged sequences across all four lineages (**Supplementary Fig. XIII.46c**). All four AQER sets show enrichments for SDs (5-fold in HAQERs, p<1e-30 one-sided binomial test) and simple repeats (2-fold, p<1e-30, one-sided binomial test); however, all the lineages also show depletions for mobile element insertion (MEIs), including SINEs, LINEs, and LTRs. There are some particular MEI families that show enrichments, such as SVAs due to their internal VNTR region. There are also shared features among the four sets, such as them all being enriched for overlapping promoters in their respective genomes at between 1.4- and 1.6-fold (human p<1e-6, chimp p<1e-6, bonobo p<1e-3, gorilla p<1e-2, one-sided binomial test)

Having nearly complete great ape genomes has greatly expanded the original set of 1,581 HAQERs derived from gapped assemblies to 3,268. These T2T HAQERs are more highly enriched in SDs, simple repeats, and centromeric satellite regions, which were previously difficult to assemble (**Supplementary Fig. XIII.46c**). These new regions from T2T genomes also show evidence of gene regulatory function based on location in the genome and their overlap with epigenomic annotations in the EpiMap dataset[160]. Of these new HAQERs, 464 show evidence of promoter function through being located within 2 kbp upstream or 500 bp downstream of a transcription start site, or overlapping epigenetic modifications associated with promoters, and 684 new HAQERs show evidence of enhancer function based on their overlap with chromatin states associated with enhancer function.

We investigated whether rapid sequence divergence is associated with particular classes of functional elements. While there is limited functional genomic data for chimpanzees, bonobos, and gorillas, we were able to analyze the HAQERs in the context of epigenomes from the Roadmap Epigenomics Consortium and EpiMap[157]. Both T2T and Gapped HAQERs exhibit enrichments for bivalent chromatin states across diverse tissues, with the strongest enrichment being for bivalent promoters (p<1e-35, one-sided binomial test) (**Fig. 4a**). Bivalent domains are thought to contain gene regulatory elements that exhibit precise spatiotemporal activity patterns in the context of development and environmental response[161]. Notably, we observed more pronounced enrichments in the shared set of Gapped and T2T HAQERs. By analyzing HAQERs in assemblies from different individuals within each species, this union set may partially control for intraspecific variation, thereby biasing the union set towards regions with fixed differences. Bivalent chromatin state enrichments were observed to a lesser degree on the chimpanzee, bonobo, and gorilla lineages (p<1e-19, p<1e-30, p<1e-12 respectively, one-sided binomial test) (**Fig. 4a**). This may reflect limited cross-species transferability of epigenomic annotations in these regions, potentially due to functional divergence in these regions of elevated sequence divergence.

We define HAQERs as the most divergent regions of the human genome, agnostic to the underlying force, or forces, of divergence. There is a non-independence between mutation rate and selection. Many regions of high mutation rates, which are deprioritized in other divergence-based studies, can be under strong selective pressure[162]. Importantly, many HAQERs may be the product of a mixture of these mutually confounding forces. Thus, we consider HAQERs to be a heterogeneous set, where the rapid divergence of some elements may be related to selection,

while others are the product of elevated local mutation rates. To emphasize this point, we analyzed the relationship between HAQERs and genomic regions that are likely to have local increases to the expected number of mutations. The three ways we approached this was to investigate the overlap between HAQERs and: repeats, GC-content, and incomplete lineage sorting. Repeat regions, such as simple repeats, tandem repeats, and SDs, can have elevated mutation rates[163,164]. GC-rich HAQERs may have locally elevated mutation rates due to error-prone polymerases, polymerase slippage, or a greater density of CpG dinucleotides[165]. ILS can also have local effects on the expected number of mutations. We identified a repeat-free subset of HAQERs, which excludes HAQERs that overlap centromeres/satellites, SDs, simple repeats, or mobile elements. This subset should have a reduced contribution from the repeat-associated increase in mutation rate. The repeat-free subset shows a stronger enrichment for overlapping the functional categories of bivalent enhancers and promoters, and the complementary set shows reduced enrichment for these gene regulatory states (**Supplementary Fig. XIII.46d**). These results are consistent with the repeat-free HAQERs having a lesser contribution from elevated mutation rates, and potentially a greater contribution from selection acting on their functional effects. Compared to the genome-wide distribution of GC-content, HAQERs show an overall bias towards regions of greater GC-content (one-sided Wilcoxon rank sum test, $p<1e-16$; **Supplementary Fig. XIII.46h**). HAQERs also show more variation in their GC-content by being fourfold enriched for occurring in GC-poor (<25%) regions ($p<1e-16$), and twofold enriched for occurring in GC-rich (>75%) regions ($p<1e-16$). Many of these GC-poor and GC-rich HAQERs overlap AT-rich or GC-rich VNTRs (64% of the GC-poor HAQERs and 29% of GC-rich HAQERs). For this reason, the GC-poor regions may still have elevated mutation rates from the VNTRs, even though their GC-content is low. The GC-rich subset of HAQERs shows a stronger enrichment for overlapping the functional categories of bivalent enhancers and promoters, and GC-poor subset shows reduced enrichment for these gene regulatory states (**Supplementary Fig. XIII.46d**). The overall bias for HAQERs to occur in regions of greater GC-content may contribute to them having an overall greater rate of mutation and also be associated with their enrichment for occurring in promoter regions. Regarding ILS, we used the TRAILS analysis to identify genomic segments associated with the four states: V0, corresponding to the standard great ape topology; V1, which shares a topology with V0 but identifies regions with deep coalescence times; V2, which captures human–gorilla ILS; and V3, which captures chimp–gorilla ILS. A genomic segment is associated with one of the four states if it has a posterior probability of 0.6 or greater for that state. We observed a significant depletion between HAQERs and the V0 state and an enrichment in the V1 state (**Supplementary Fig. XIII.46e**). This suggests that nonuniformity in coalescence times across the genome may be an additional consideration for analyzing human divergence rates. However, we note that the V1 state does not measure imbalances between the branch length separating the human from the human–chimp ancestor and separating the chimp from the human–chimp ancestor, which would reflect human-specific divergence. We note only minor enrichments between HAQERs and regions with nonstandard topology (**Supplementary Fig. XIII.46e**). This may be a consequence of our conservative thresholding for ancestral state inference, which ignores position with substantial uncertainty in the human–chimp ancestral state, as would be expected in positions

associated with ILS. Taken together, these two analyses show that many forces and scenarios can contribute to elevated interspecies divergence.

HAQERs were initially identified by scanning the genome for regions of elevated local divergence in 500 bp windows. To determine if functional annotation enrichments in HAQERs were dependent on this ascertainment window size, we generated HAQER sets at a range of sliding window sizes, from 25 bp to 20 kbp, to create multi-scale divergence landscapes of the human genome (**Supplementary Fig. XIII.46f**). This analysis revealed some rapidly evolving elements are only detectable at specific window sizes (**Supplementary Fig. XIII.46f**, left and right), and that many larger-scale rapidly evolving elements are composites of multiple, constituent regions at smaller scales (**Supplementary Fig. XIII.46f**, middle). While bivalent gene regulatory and heterochromatin state enrichments for HAQERs are heavily dependent on the ascertainment window size, HAQERs were consistently depleted from transcribed regions across window sizes (**Supplementary Fig. XIII.46g**).

We have not included the orangutan in this AQER analysis. To analyze the lineage leading to orangutan, we need to be able to confidently estimate the human–orangutan ancestor, which we could not accomplish to the same degree as we could for the human–chimp or human–gorilla ancestor. The lineage leading to orangutan is more than twice as long as the other four lineages examined. Already on the human lineage some regions, such as centromeres change so rapidly that they cannot be aligned with current methods, and therefore cannot be included in our analyses. This would be exaggerated in orangutan-AQERs, making it difficult to compare across the sets Additionally, only 73% of the bases in the orangutan assembly align to a base in the gibbon assembly, which is needed to confidently infer the base at the human–orangutan ancestral node. This is in comparison to the human lineage, where 92% of human bases have a base from an outgroup species present. Overall, we do not think our alignment and ancestral inference methods are currently at the point where we can estimate the human–orangutan ancestor to the same degree of confidence that we can for the human–chimp or human–gorilla ancestral nodes.

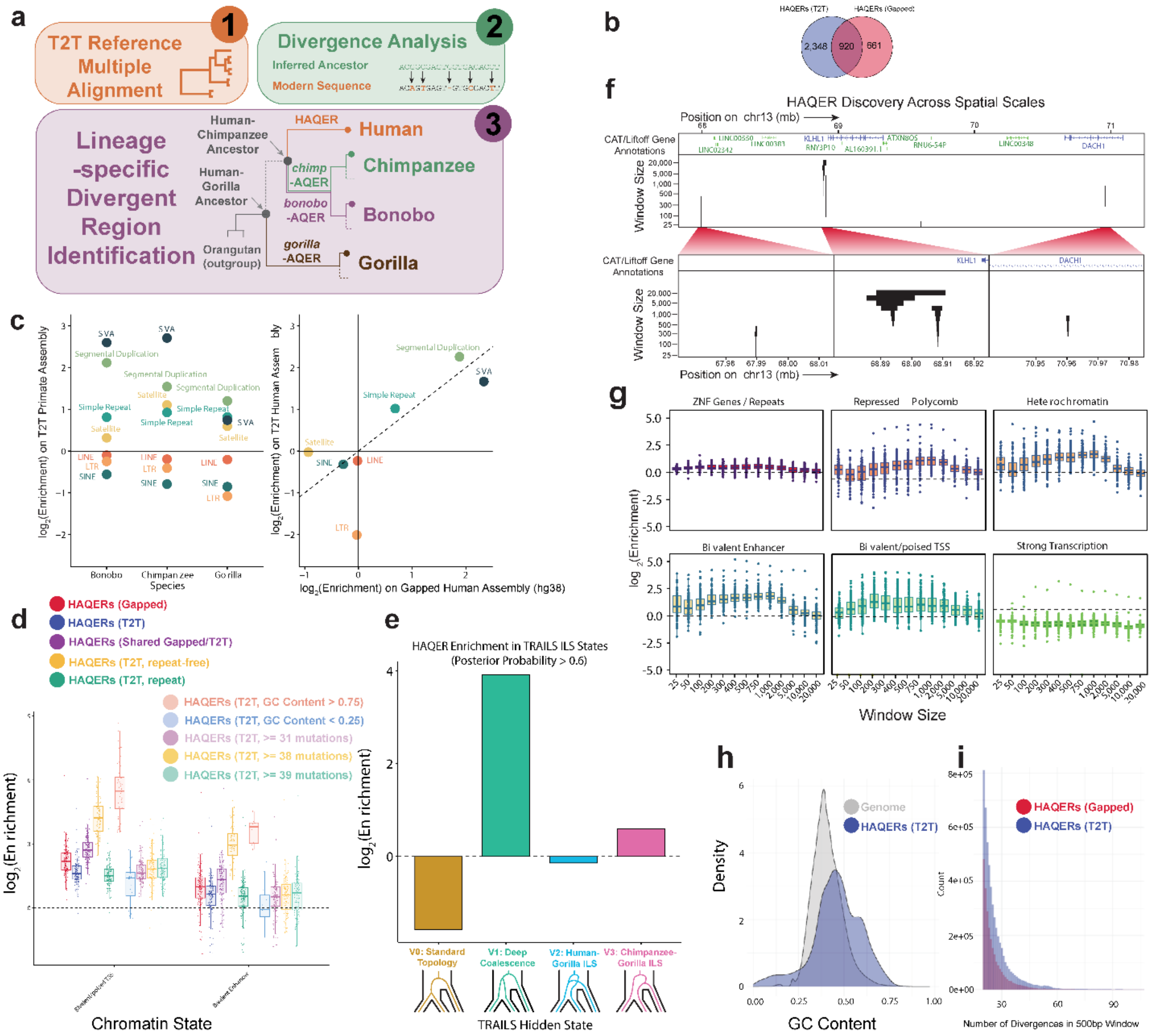

Supplementary Figure XIII.46. Great ape T2T assemblies resolve highly divergent genomic regions. (a) We ascertained HAQERs (human ancestor quickly evolved regions), and similarly fast-evolving regions in other great ape species, by identifying regions of elevated sequence divergence in 500 bp windows. (b) There are 920 gapped HAQERs that overlap any T2T HAQERs lifted to the gapped hg38 assembly. (c) AQERs are enriched in SVAs, simple repeats, and SDs, but not across the general classes of SINEs, LINEs, or LTRs. With T2T genomes, the set of HAQERs previously defined using gapped genome assemblies became even more enriched for simple repeats and SDs (right). (d) The HAQER sets based on both gapped and T2T assemblies show enrichments for bivalent gene regulatory elements across 127 cell types and tissues (n=127 biologically independent samples for each chromatin state. Boxes show interquartile range and median, with whiskers showing data points within 1.5 times the standard deviation). The set of HAQERs shared between the gapped and T2T sets shows an even stronger enrichment for this functional state. The repeat-free subset of T2T HAQERs, which excludes HAQERs that overlap centromeres/satellites, SDs, simple repeats, or MEIs, shows a stronger bivalent enrichment, and the complementary set shows reduced bivalent enrichments. GC-rich

HAQERs show stronger enrichments than GC-poor HAQERs. As the threshold is increased from 29 to 31, 38 or 39 mutations in a 500 bp window, the set of HAQERs shows a slightly greater enrichment for the bivalent state. These thresholds correspond to using the fastest 10 Mbp, 5 Mbp, 1 Mbp, or 500 kb as the expected rate of divergence in a 500 bp window. (e) The T2T HAQER set is significantly depleted in the V0 state (standard great ape topology) and enriched in the V1 state (standard great ape topology regions with long branches). We note only minor enrichments between HAQERs and regions with nonstandard topologies V2 (human–gorilla ILS) and V3 (chimp–gorilla ILS). (f) HAQER genomic locations, observed across spatial scales, in a 4 Mbp region of chr13. Bottom: 50 kbp zoomed-in inserts. (g) The influence of divergence scale on chromatin state enrichment (n=127 biologically independent samples for each chromatin state. Boxes show interquartile range and median, with whiskers showing data points within 1.5 times the standard deviation). (h) The plot compares the GC-content in 500 bp windows across the human genome (randomly sampling 1%) compared to sites defined as HAQERs. HAQERs have anonymously high GC-content. (i) T2T primate genomes have increased our ability to identify highly divergent regions of the human genome. Here we show the distribution of divergence for windows with 24 or more mutations on the human lineage (29 is the HAQER threshold) that could be identified with incomplete genomes (Gapped) or T2T genomes (T2T). For these high levels of divergence visualized in the plot, there are consistently more windows detected with T2T genomes.

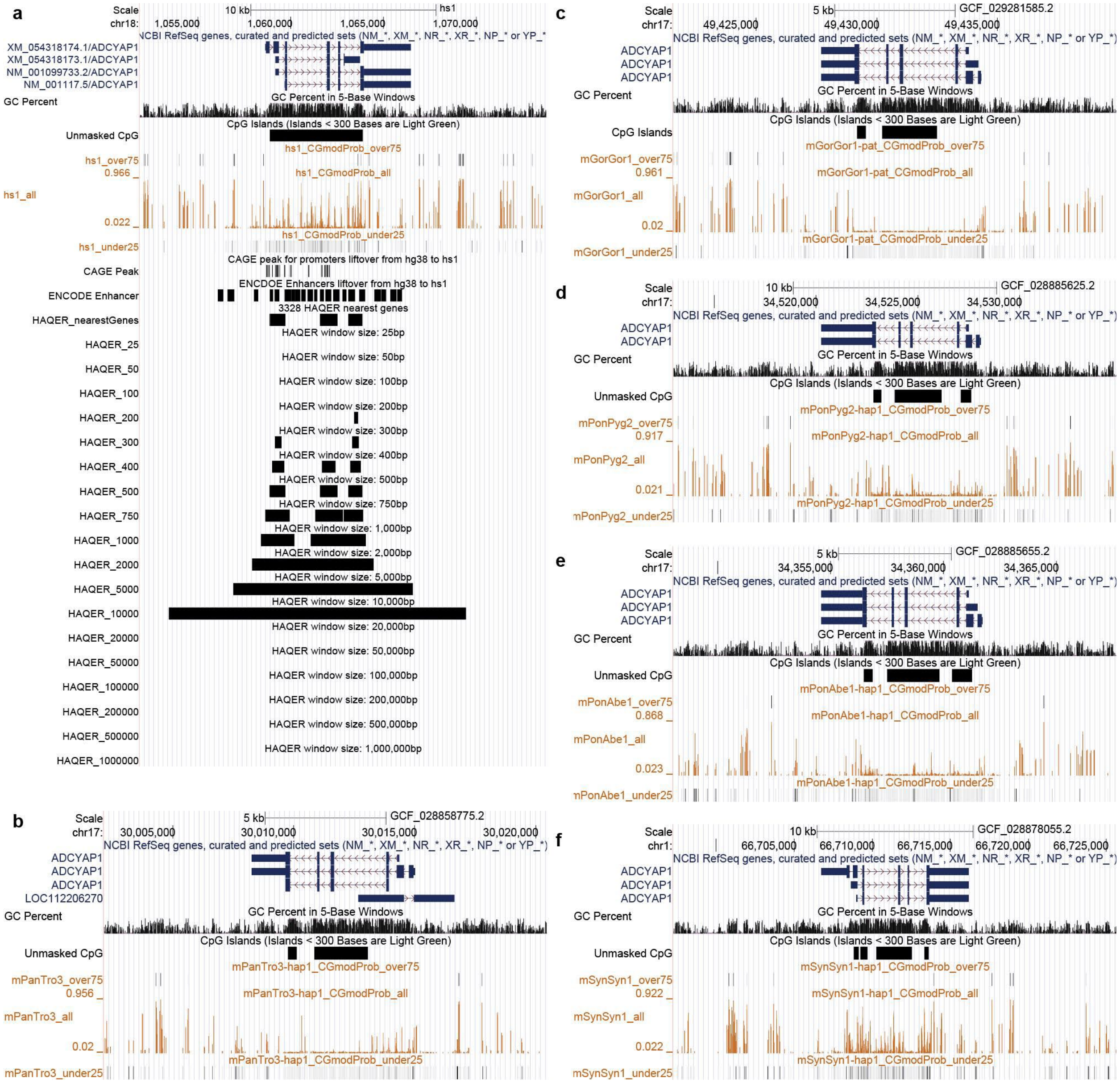

Supplementary Figure XIII.47. DNA hypomethylation pattern on *ADCYAP1* gene bodies of apes. (a) Human, (b) chimpanzee, (c) gorilla, (d) Bornean orangutan, (e) Sumatran orangutan, and (f) siamang gibbon. All panels show NCBI annotation, GC percent, CpG islands, DNA hyper-methylated loci (probability > 75%), DNA methylation probabilities on CpG, and DNA hypo-methylated loci (probability < 25%). The methylation probabilities estimated from PacBio HiFi reads. CAGE peaks and ENCODE enhancers were lifted over from hg38 to hs1. HAQERs were identified in different windows (25 bp-10 Mbp) and HAQER nearest genes were classified by considering transcription start sites of each gene.

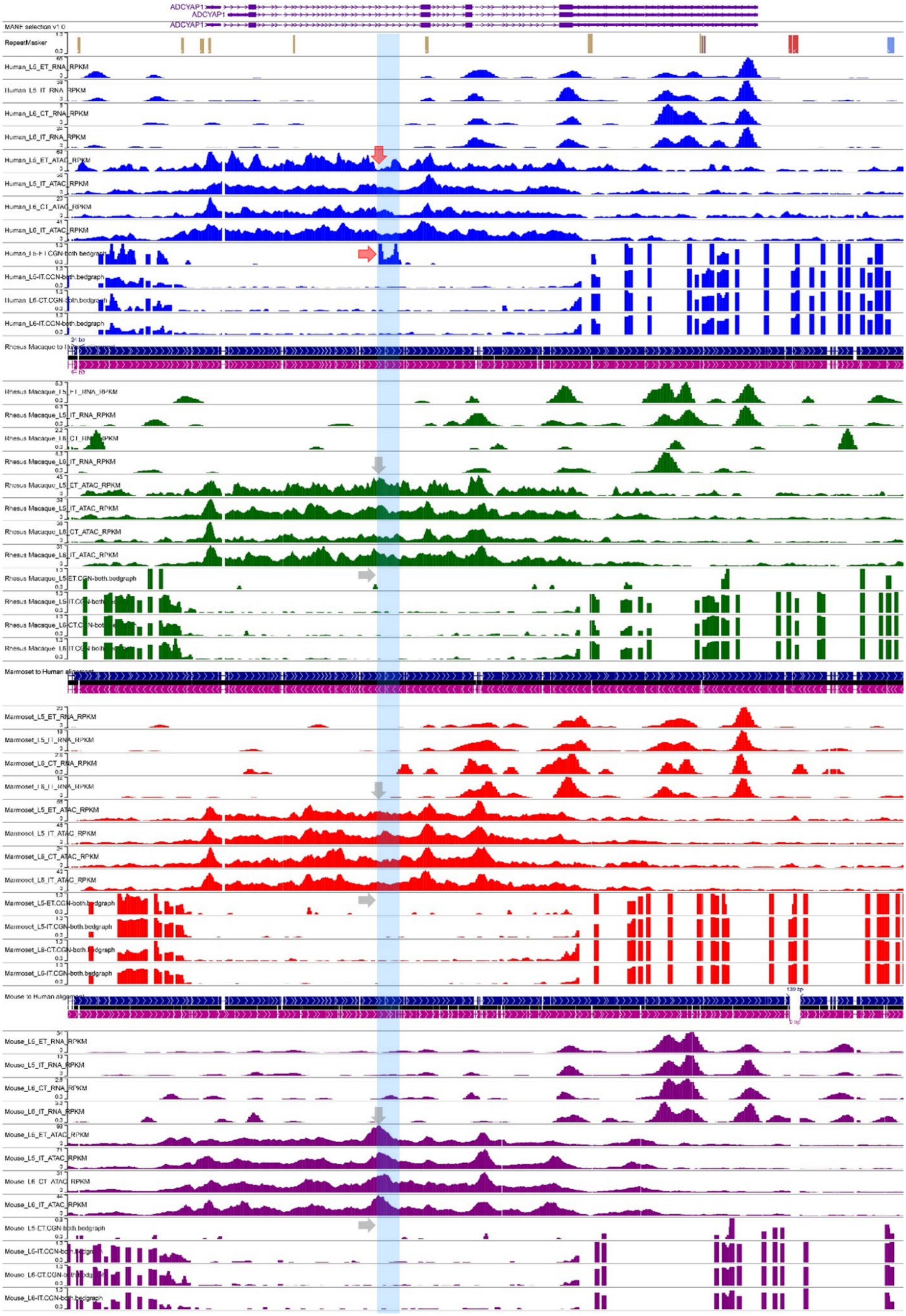
ADCYAP1
ADCYAP1
ADCYAP1
MANE selection v1.0
RepeatMasker
Human_L5_ET_RNA_RPKM
Human_L5_IT_RNA_RPKM
Human_L6_CT_RNA_RPKM
Human_L6_IT_RNA_RPKM
Human_L5_ET_ATAC_RPKM
Human_L5_IT_ATAC_RPKM
Human_L6_CT_ATAC_RPKM
Human_L6_IT_ATAC_RPKM
Rhesus Macaque_L5_IT_RNA_RPKM
Rhesus Macaque_L6_IT_RNA_RPKM
Rhesus Macaque_L5_ET_ATAC_RPKM
Rhesus Macaque_L5_IT_ATAC_RPKM
Rhesus Macaque_L6_CT_ATAC_RPKM
Rhesus Macaque_L6_IT_ATAC_RPKM
Rhesus Macaque_L5ET.CGN-both.bedgraph
Marmoset_L5_ET_RNA_RPKM
Marmoset_L6_CT_RNA_RPKM
Marmoset_L5_ET_ATAC_RPKM
Marmoset_L5_IT_ATAC_RPKM
Marmoset_L6_CT_ATAC_RPKM
Marmoset_L6_IT_ATAC_RPKM
Marmoset_L5-IT.CGN-both.bedgraph
Marmoset_L6-IT.CGN-both.bedgraph
Mouse_L5_ET_RNA_RPKM
Mouse_L6_CT_RNA_RPKM
Mouse_L6_IT_RNA_RPKM
Mouse_L5_ET_ATAC_RPKM
Mouse_L5_IT_ATAC_RPKM
Mouse_L6_IT_ATAC_RPKM
Mouse_L6-IT.CGN-both.bedgraph

Supplementary Figure XIII.48. Human-unique epigenetic patterns different from macaque, marmoset, and mouse. Each four rows show scRNA seq read depths, scATAC-seq read depths, and DNA methylation probabilities of layer 5 ET / IT and layer 6 CT / IT neurons of primary motor cortexes of human, macaque, marmoset, and mouse. Modified from Comparative Epigenomics browser (https://epigenome.wustl.edu/BrainComparativeEpigenome/).

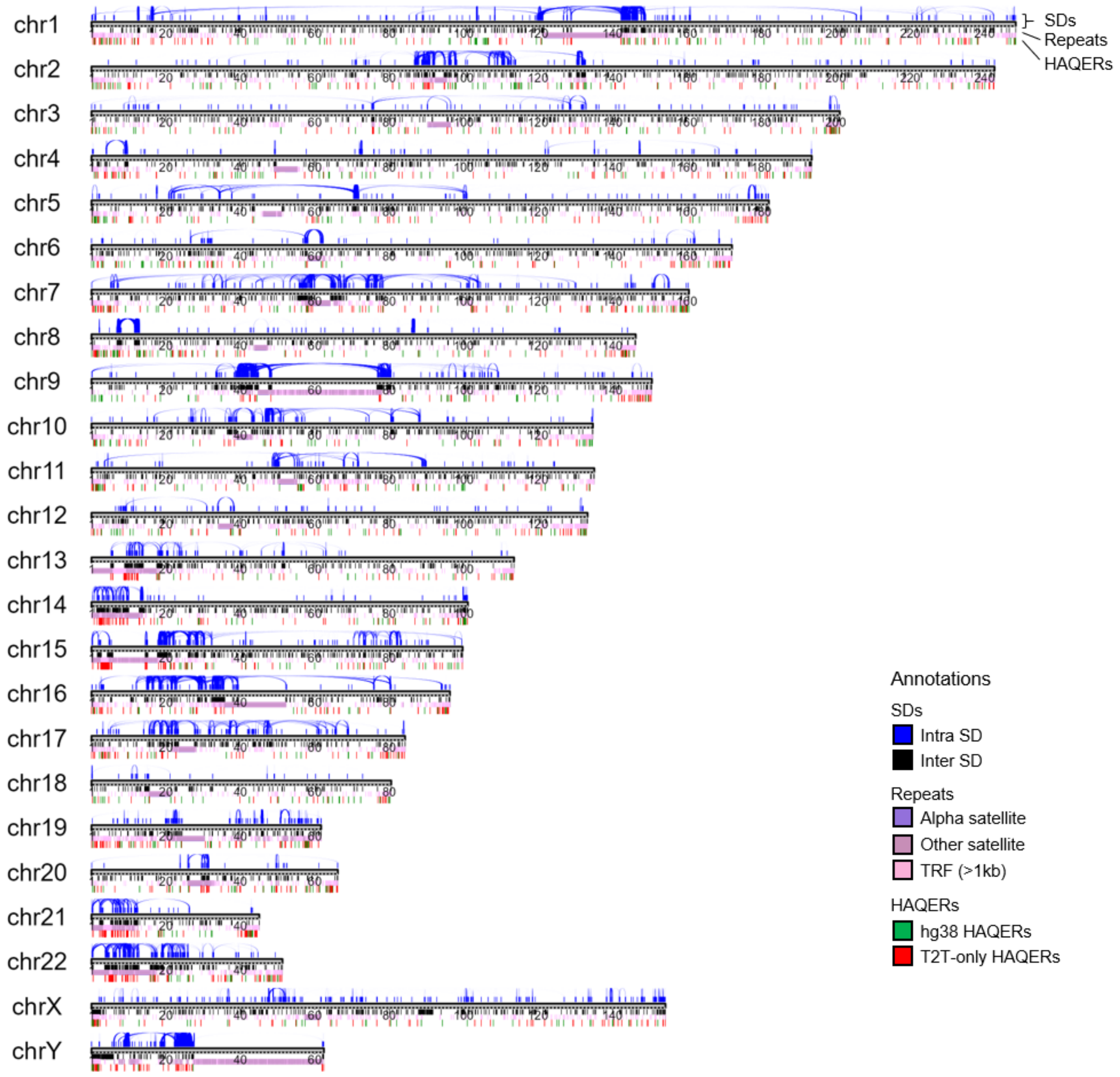

Supplementary Figure XIII.49. Genome-wide distribution of HAQERs (the hg38 shared ones in green and T2T-only ones in red). SDs (intrachromosomal and interchromosomal in blue and black, respectively) and the satellite-rich sequences (pinks and purples) are indicated to show regional bias of the newly discovered HAQERs.

XIV. TOGA analysis

Contributing authors:

Agnes Chan, Michael Hiller, Nicholas J. Schork

Method

TOGA (Tool to infer Orthologs from Genome Alignments) inference resource[166] was used to identify human gene loci that were reported as "absent" across more than 40 primate species. The pipeline was applied to the current six ape T2T assemblies to identify and refine genes lost during ape evolution.

Summary of results

TOGA human-specific genes

We identified six examples of candidate genes that are likely unique to the human lineage and 19,148 (out of 19,244, 99.5%) protein-coding genes that are present in two or more of the six ape T2T assemblies. TOGA predictions for the primate assemblies (group 1) and the ape pre-T2T assemblies (group 2) as of 2023 were collected from Kirilenko et al.[166] The existing ape assemblies from 2023 included silvery gibbon, northern white-cheeked gibbon, Sumatran orangutan, western lowland gorilla, pygmy chimpanzee, and chimpanzee. New TOGA predictions were generated in this study for six ape T2T assemblies. Human-specific gene candidate sequences were identified based on TOGA predictions that reported gene absence across over 80% of the assemblies analyzed. A summary of human genes selected from each assembly group based on the "over 80% absence" criteria are shown in bold in **Supplementary Table XIV.75**. Additional evidence was collected from the T2T-CHM13 UCSC Genome Browser, including whole-genome alignments of the ape T2T assemblies against T2T-CHM13, and SD predictions from this study. For one of the candidate genes, *FOXO3B*, we carried out in-depth validations using nucleotide comparison including flanking sequences and confirmed its absence across five of the ape T2T primate genomes except gorilla, and its unique genome architecture embedded in a clinically relevant locus in the human genome.

We describe an example of human-specific gene sequences related to the major longevity gene *FOXO3*, which encodes a transcription factor with prevalent functional roles in regulating apoptosis, autophagy, and metabolism[167]. The association of *FOXO3* with longevity was first reported in a Japanese-American Hawaiian cohort[167] and subsequently replicated in multiple European cohorts (e.g., Flachsbart et al.[168]). Proposed mechanisms for the longevity phenotype of *FOXO3* included a *FOXO3* haplotype-induced chromatin hub spanning multiple transcription factor binding sites, and its transcript isoforms[169,170]. Identifying human gene loci that were reported as "absent" across more than 40 primate species included in the TOGA analysis

(**Supplementary Fig. XIV.50a**) revealed *FOXO3B* as one of the top-ranked genes that were not detected across almost all primate species analyzed. In terms of gene structure, *FOXO3B* is a chimeric gene that arose as a fusion of three exons of a zinc finger gene and a processed pseudogene copy of *FOXO3* that makes up its last exon. *FOXO3B* mRNA expression by RT-qPCR has been reported across multiple tissues, including the cerebellum, fetal brain, and neural progenitor cells and cell lines[171].

Using the high-quality, long-read derived genome assemblies from the ape T2T project, we investigated the presence of *FOXO3B* in NHPs. Across the ape T2T species, *FOXO3B* sequence was not found in chimpanzee, bonobo, orangutan, or gibbon that seem to lack the *FOXO3* processed gene insertion and thus the last *FOXO3B* exon. *FOXO3B* was also not found in the New World monkey marmoset. Of note, the complete *FOXO3B* gene was detected in the gorilla genome, where it encodes an intact reading frame. It should be noted that the genomic regions flanking FOXO3B in the gorilla genome have rearrangements compared with the human reference assemblies (i.e., T2T-CHM13, GRCh38) (**Supplementary Fig. XIV.50b**). CHM13 Iso-Seq data supports expression of *FOXO3B* locus.

We investigated the genomic context of the human *FOXO3B* locus and revealed a complex structure involving five local low-copy repeats or SDs of ~200 kbp at chromosome 17 near the centromere. We mapped the location of the *FOXO3B* locus to one of the low-copy repeats embedded within a 4 Mbp region at 17p11.2 (**Supplementary Fig. XIV.50c**). Previous studies have shown that microdeletion or microduplication of this region is associated with Smith-Magenis syndrome (SMS) and Potocki-Lupski syndrome (PLS), respectively[172]. The clinical impacts of SMS and PLS include developmental delay and cognitive impairment. The causal gene of SMS and PLS is believed to be *RAI1* located within 17p11.2, but whether *FOXO3B* could be involved in cognitive impairment requires further analysis.

It is worth noting that complex co-regulation networks of a parent gene and its pseudogene counterpart involving coding and noncoding mechanisms have been reported in transcription factors with significant roles in tumor suppression (e.g., *PTEN* and the pseudogene *PTENP1*), and embryonic and stem cell development (e.g., *POU5F1/OCT4* and five pseudogenes *POUR5F1P1* to *POUR5F1P5*)[173].

In summary, we discovered the special evolutionary history of the *FOXO3B* gene locus through comparative analysis of human and primate assemblies and ortholog annotation. Many questions remain, such as: the functional significance of *FOXO3B* in longevity, apoptosis, etc., whether *FOXO3B* is translated into a protein in human and gorilla, whether *FOXO3* and *FOXO3B* could be subjected to co-regulation networks, and, if so, in which tissues/cell types and developmental stages, and the ancestral origin and evolution of the *FOXO3B* locus across humans and the great apes.

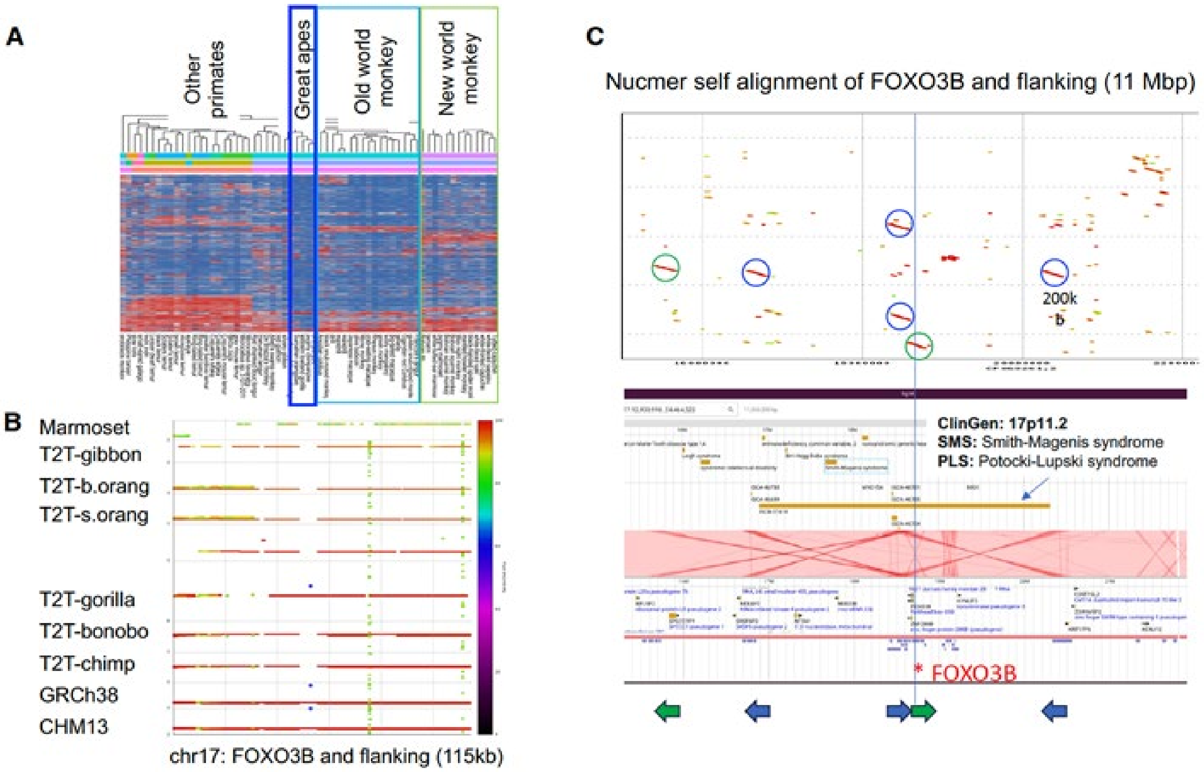

Supplementary Figure XIV.50. Human-specific gene sequences encoding FOXO3-like sequences. (A) Predicted gene loss (red) across over 40 primate species as reported by TOGA. (B) The 7 kbp *FOXO3B* (asterisk) was only detected in the human (CHM13, GRCh38) and T2T gorilla genome assemblies, yet absent from other T2T primate genomes. C) *FOXO3B* resides with a 4 Mbp clinically significant locus known to link to cognitive impairment.

XV. Non-B DNA annotations

Contributing authors:

Kaivan Kamali, Linnéa Smeds, Edmundo Torres-González, Kateryna Makova

Methods

Non-B DNA motifs were annotated in each species using gfa (https://github.com/abcsFrederick/non-B_gfa). We used default settings, except the flag --*skipGQ*, to annotate A-phased repeats, direct repeats, inverted repeats, mirror repeats, short tandem repeats, and Z-DNA. Triplex motifs were extracted from mirror repeats ('subset=1' in gfa, containing mirror repeats with a maximum spacer length of 8 bp and a minimum purine/pyrimidine content of 10%). G-quadruplexes were annotated using Quadron[174]. For each motif type, the output was converted to bed format, and mergeBed from BEDTools[83] was used to merge any overlapping annotations.

Alignments to old assembly versions

For each species that had a corresponding older non-T2T assembly, the new T2T assembly was aligned to its predecessor (panPan3 for bonobo, panTro6 for chimpanzee, gorGor6 for gorilla and ponAbe3 for Sumatran orangutan; Bornean orangutan and siamang were excluded from this analysis because they were sequenced here for the first time and thus their previous assemblies are unavailable). Each chromosome pair (new vs. old) was aligned separately with Winnowmap v2.03[175]. According to the recommendations, we first generated a set of high-frequency *k*-mers with meryl v.14.1[7] using k=19. To assess regions in the T2T assemblies that were previously unassembled, we merged all the aligned sequences, and then extracted everything that did *not* align to the previous assembly using BEDTools complement. This is from now on referred to as the 'new' sequence.

Enrichment of non-B DNA in new sequence

To investigate the density of non-B motif annotations in new versus previously assembled sequence, we intersected the non-B annotations of each motif type separately with the new regions defined above, as well as to the previously assembled regions, using BEDTools intersect and the flag -wao that outputs the number of overlapping base pairs. The overlaps with new and previous sequence were summed up for each motif type separately, and the fold enrichment was calculated as non-B density in the new sequence divided by non-B density in previously assembled sequence.

XVI. Methylation

Contributing authors:

Dongmin R. Son, Yong Hwee Eddie Loh, Soojin V. Yi

Methods

DNA methylation analysis

To generate CpG methylation calls, ONT data were base-called using Guppy v6.3.7 with the model "dna_r9.4.1_450bps_modbases_5hmc_5mc_cg_sup_prom.cfg". Counts of modified bases at each cytosine position were generated using modbam2bed v0.10.0 after minimap2 v2.26 mapping to the genome. Given that 5mC in the CpG context is symmetrical, read counts of two cytosines in the CpG context were combined to represent a single CpG site. The fractional methylation level at each CpG site was calculated for CpG sites, which had at least five read counts.

We generated a list of confident orthologous protein-coding promoters for comparative studies on promoter methylation level changes across species. We first defined human promoter regions as 1000 bp upstream to 500 bp downstream of the transcription start site of protein-coding genes as annotated by the CuratedRefSeqv5.1 annotation track on the human HG002v1.0.1 paternal genome. We used halLliftOver against a 16-way Cactus alignment to identify orthologous promoters in NHP primary genomes. Additionally, we restricted our analysis to genes that are detected as single-copy and intact according to TOGA (where the middle 80% of the CDS was present and exhibited no gene-inactivating mutations) based on the TOGA hg38 gene annotations of NHPs. We calculated the distance between the center of orthologous promoters and the 5' end of genes using the RefSeq annotation track for each NHP, filtering out genes with the distances over 2.5 kbp. Out of these 8,256 confident orthologous protein-coding promoters between humans and six NHPs, 8,177 promoters had at least six CpGs, and 8,174 also had available expression levels (**Supplementary Tables XVI.76-77**).

We calculated the mean 5mC values per promoter. We normalized these values within each species using the z-score transformation to scale between 0 and 1, using scikit-learn MinMaxScaler[176]. We then developed a comparative method to assess whether promoters exhibited consistent methylation patterns or species-specific differences. This approach was based on pairwise 5mC level comparisons between species, with significance thresholds determined using the distribution of promoter 5mC level differences for each species pair, as guided by the Central Limit Theorem. The threshold values are shown in **Supplementary Fig. XVI.51A**. Analyses were performed separately for lymphoblastoid and fibroblast cell lines. Promoters were classified as consistently methylated if all pairwise 5mC differences between

species fell within two standard deviations (2*SD), while those exceeding this threshold were classified as variably methylated. Additionally, we categorized these promoters as hypermethylated and hypomethylated using a cutoff of 0.5, as in previous papers[177]. We show that the majority of promoters are consistently methylated across the studied species. In addition, while the majority of consistently methylated promoters are hypomethylated, hypermethylated promoters are enriched in variably methylated promoters (P<0.001 for both cell lines, Fisher's exact tests). Species-specific 5mC gain in a promoter was defined as a significant increase in 5mC levels in the species of interest, exceeding the 2SD threshold in all relevant pairwise comparisons, while differences in other species remained relatively stable within the 2SD thresholds. Conversely, species-specific 5mC loss was characterized by a significant decrease in 5mC levels in the species of interest, where the decrease exceeded the 2SD threshold in all pairwise comparisons, with differences in other species remaining within the 2SD thresholds (**Supplementary Tables XVI.76-77**). We visualize the methylation levels of promoters identified as species-specific 5mC gain and loss from this analysis. We then examined DNA methylation levels (5mC), level of gene expression and CpG counts of consistently methylated and variably methylated promoters in **Supplementary Fig. XVI.52**. We also assessed species-specific and shared MEIs in **Supplementary Fig. XVI.53**.

A

Lymphoblastoid

Pairwise comparison	Mean	Lower threshold (Mean - 2*SD)	Upper threshold (Mean + 2*SD)
Human - Chimpanzee	-0.0082	-0.180	0.163
Human - Siamang	-0.0281	-0.237	0.181
Chimpanzee - Siamang	-0.0199	-0.207	0.167

Fibroblast

Pairwise comparison	Mean	Lower threshold (Mean - 2*SD)	Upper threshold (Mean + 2*SD)
Bonobo-Gorilla	-0.0066	-0.218	0.204
Bonobo-SOrang	-0.0443	-0.272	0.184
Bonobo-BOrang	-0.0387	-0.259	0.182
Gorilla - SOrang	-0.0377	-0.187	0.111
Gorilla - BOrang	-0.0322	-0.184	0.119
SOrang - BOrang	0.0056	-0.095	0.106

B

Lymphoblastoid

Consistency		# of promoters	Total
Consistent	Hypomethylated	6830	7252
	Hypermethylated	422	
Variable	Hypomethylated	518	922
	Hypermethylated	404	

Species-specific Gain/Loss 5mC	# of promoters		
	Human	Chimpanzee	Siamang
Gain	145	49	145
Loss	26	66	70

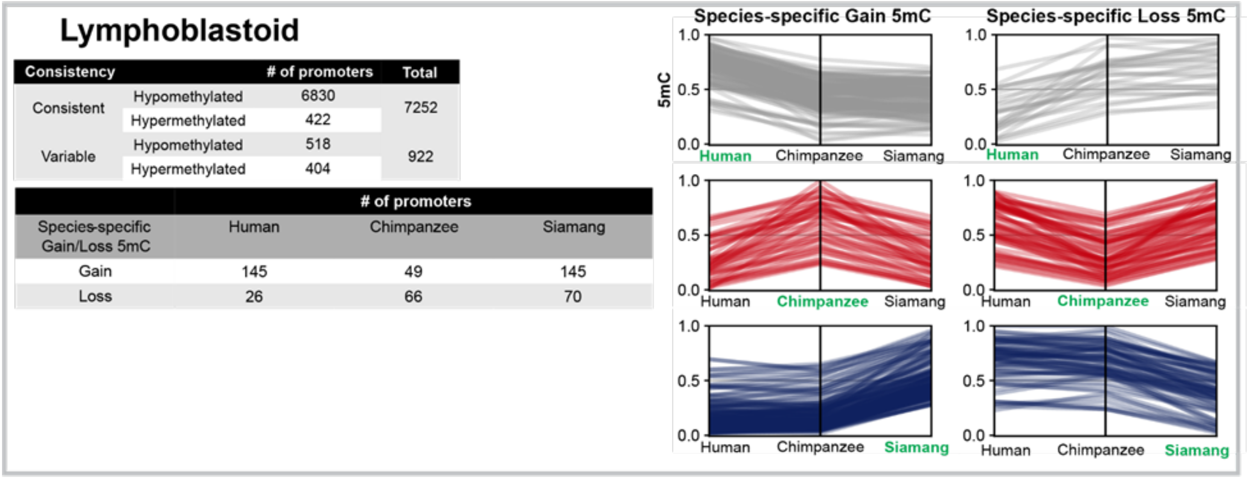

C

Fibroblast

Consistency		# of promoters	Total
Consistent	Hypomethylated	6812	7139
	Hypermethylated	327	
Variable	Hypomethylated	519	1035
	Hypermethylated	516	

Species-specific Gain/Loss 5mC	# of promoters			
	Bonobo	Gorilla	Sumatran Orangutan	Bornean Orangutan
Gain	65	26	23	15
Loss	129	24	10	7

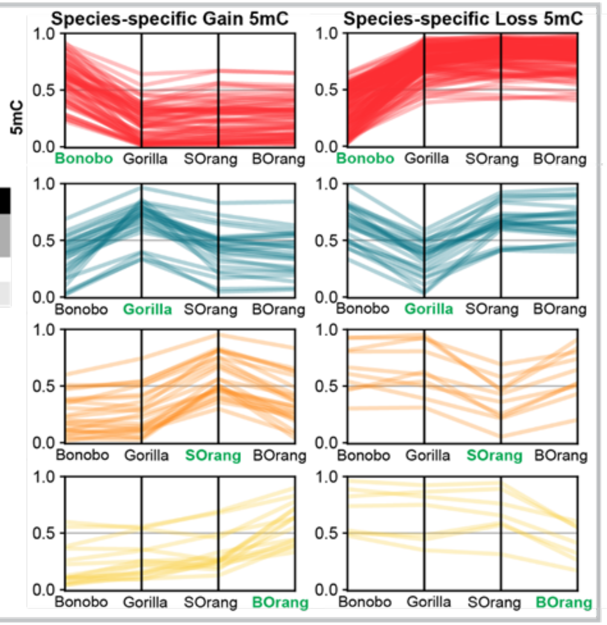

Supplementary Figure XVI.51. (A) The thresholds used to assess significant methylation variation or consistency between species. (B) Results for Lymphoblastoids. The number of consistently or variantly methylated promoters among species and the number of promoters that show significant species-specific 5mC gain or loss. Plots show the DNA methylation of species-specific 5mC gain and loss promoters across the compared species. (C) Same results for Fibroblasts.

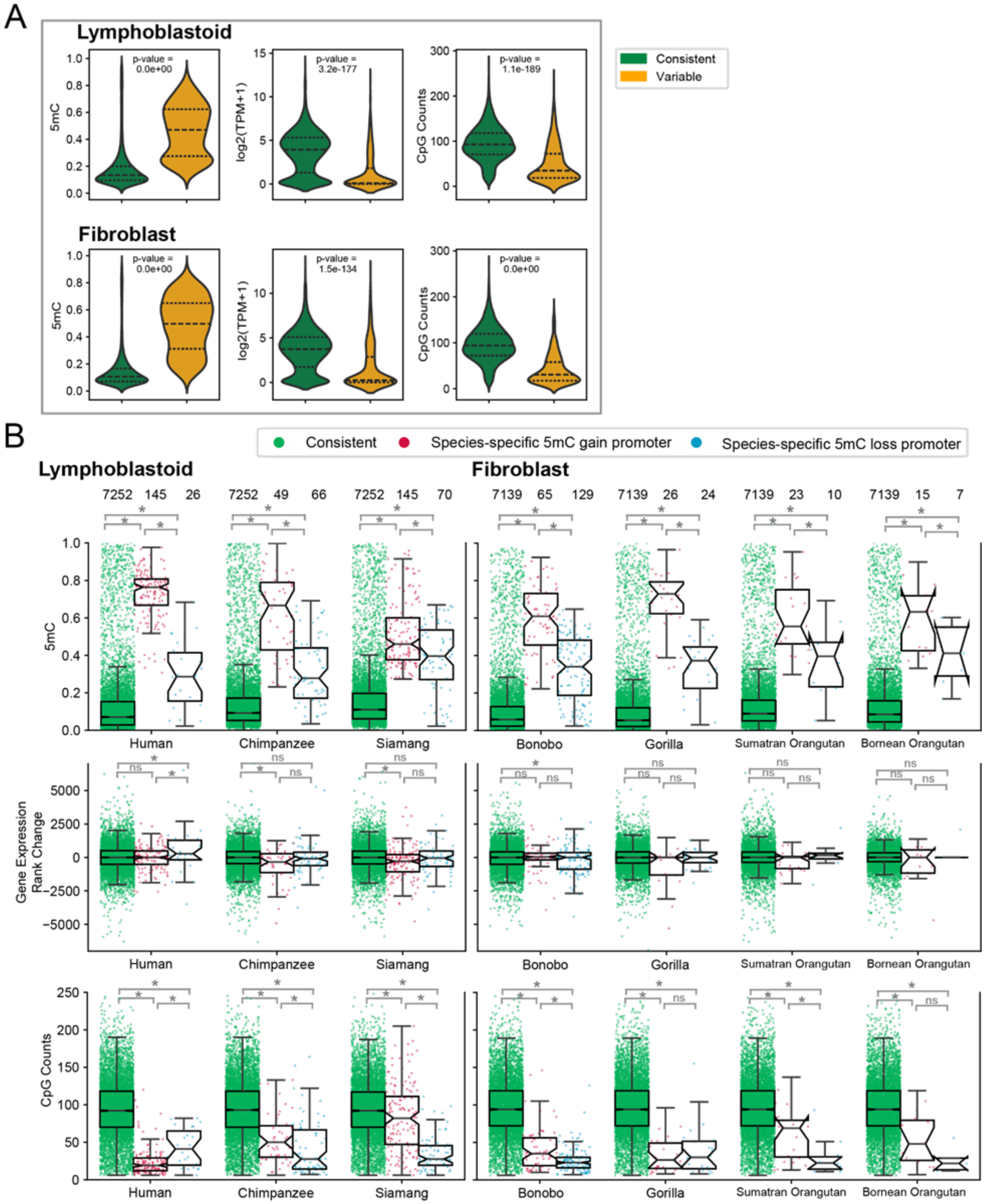

Supplementary Figure XVI.52. DNA methylation analysis of 8,174 orthologous promoters between humans and nonhuman primates (NHPs), examining the divergent characteristics of variably and consistently methylated promoters. The data were analyzed separately for lymphoblastoid and fibroblast cell lines. (a) Consistently methylated promoters tend to be lowly

methylated, more highly expressed, and have a higher density of CpG sites compared to variably methylated promoters. P-values based on the two-sided Mann–Whitney U test are shown, and all are significant. (b) Comparisons of promoters exhibiting species-specific 5mC gain and loss versus those consistently methylated across NHP species. Each dot represents a promoter. P-values are determined using the two-sided Mann–Whitney U test ($p<0.05$: '*', $p<0.01$: '**', or 'ns'). The top panel shows that promoters that display species-specific DNA methylation change tend to be more highly methylated than consistently methylated promoters, regardless of whether they gain or lose DNA methylation. In the middle panel, the ranks of each gene's expression (ranging from 0 for the lowest expressed genes to 8,174 for the highest expressed genes within each species) were determined and each gene's rank change in a species compared to the ranks of the genes in other species was plotted. While consistently methylated promoters show a distribution around 0 (meaning that their expression ranks remained constant), promoters with species-specific 5mC gain tend to show negative values below 0 in gene expression rank changes, indicating that their expression rank decreased compared to in other species. In contrast, those with 5mC loss tend to show positive values above 0 in gene expression rank changes, indicating a trend of higher expression in the species compared to in other species. However, these trends were not always significant, as the numbers of 5mC gain or loss promoters were relatively small. In the bottom panel, we show that promoters that have species-specific DNA methylation change tend to have fewer CpG sites compared to consistently methylated promoters, underscoring the importance of sequence backgrounds in the evolution of epigenome.

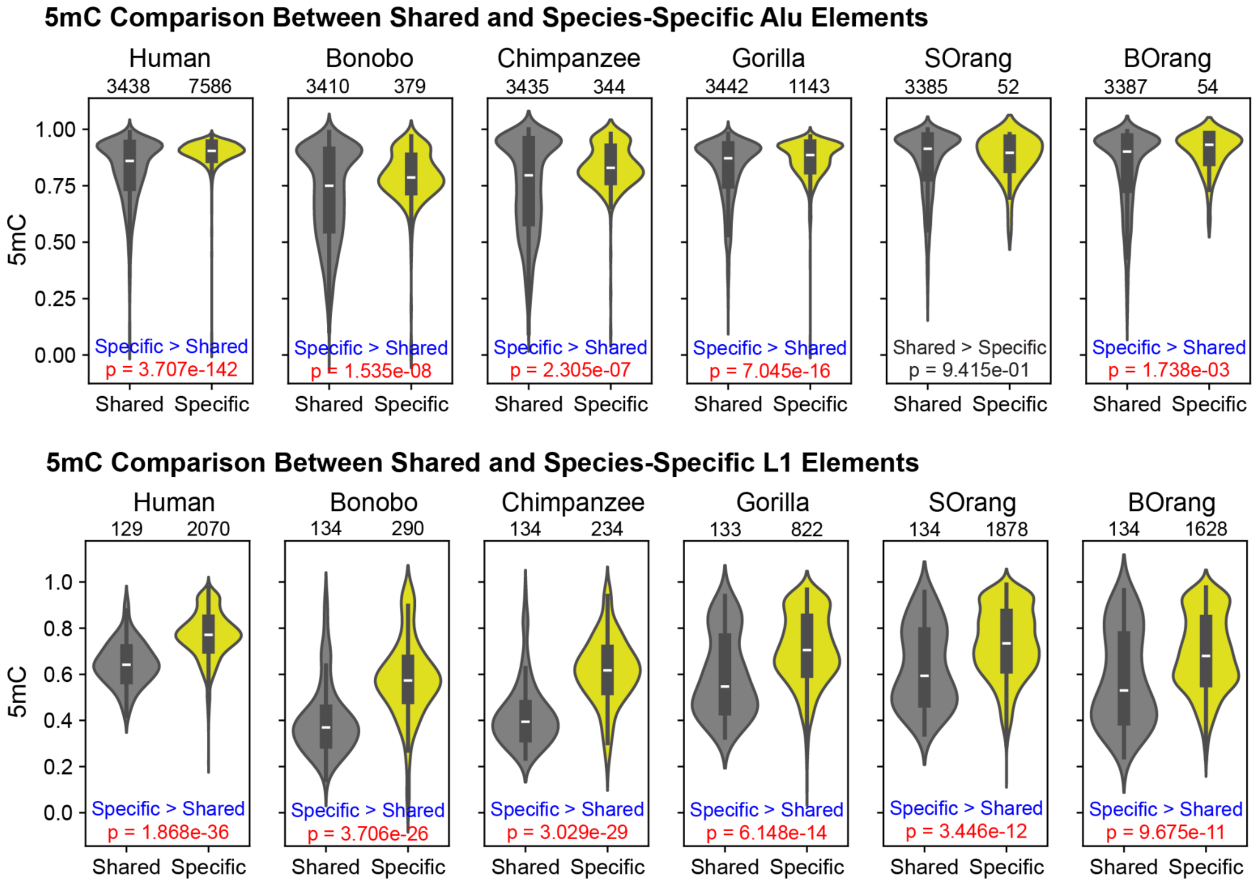

Supplementary Figure XVI.53. DNA methylation differences between species-specific elements and elements shared between all species. Results from (top) Alus and (bottom) L1 elements demonstrate that species-specific elements are significantly more highly methylated compared to shared elements (two-sided Mann-Whitney tests, P-values show in each panel).

XVII. Replication timing

Contributing authors:

Jian Ma, Muyu Yang, Yang Zhang, David Gilbert, Takayo Sasaki, Gabrielle A. Hartley, Emry Brannan, Rachel J. O'Neill

Methods

Cross-species replication timing profiling and evolutionary patterns identification

Replication timing profiling of primate species followed the same procedure described previously[178]. Repli-seq was processed based on a published workflow[179]. Cutadapt (version 4.2) removed the remaining adapters on the reads with parameters "-q 0 -O 1 -m 0", using the adapter sequence AGATCGGAAGAGCACACGTCTG. Reads were mapped to the human (T2T-CHM13v2.0 and NHP assemblies with bwa mem (version 0.7.17)[180] with default parameters. PCR duplicates were removed using SAMtools rmdup tool.

The genomes were segmented into nonoverlapping 5 kbp bins, and mapped reads were counted and normalized to RPKM. Bins with less than 0.1 RPKM across both early and late fractions were removed. The log2 ratio of early to late fractions was calculated and normalized using interquartile range (IQR) normalization, i.e., (value – median)/IQR. Ratios were smoothed with the loess method with 300 kbp window size.

For cross-species comparison, reciprocal liftOver converted NHP replication timing profiles to the human T2T genome (chm13, version 2.0). The human genome was segmented into nonoverlapping 5 kbp bins and mapped to NHPs using liftOver[181], retaining only reciprocally mapped bins. Next, Phylo-HMGP[178], a continuous-trait probabilistic model, inferred evolutionary states for functional genomic signals, identifying 20 states representing distinct evolutionary patterns of replication timing profiles across primate species.

Summary of results

Evolutionary patterns of replication timing

To characterize changes in replication timing across the primate species, we applied Phylo-HMGP[178], focusing on lymphoblastoid cell lines from human, chimpanzee, Bornean orangutan, and siamang. Phylo-HMGP identified 20 states with distinct evolutionary patterns (**Supplementary Fig. XVII.54A**). These states were classified into five categories based on the

replication timing signal values and the variability across the species: early (E), weakly conserved early (WE), late (L), weakly conserved late (WL), and non-conserved (NC). We observed that 31.5% of the genome is in conserved early state and 21.5% in the conserved late state. Lineage-specific states were mainly reflected by weakly conserved and NC states. For example, State 13 has a unique early replication timing specific to chimpanzees, while State 4 shows a distinct late replication timing unique to Bornean orangutans. The distribution of replication timing signals for each state, organized by category, is displayed in **Supplementary Fig. XVII.54B-C** shows an example of replication timing profiles and Phylo-HMGP states on the genome browser.

Correlation between SDs and replication timing evolutionary patterns

We analyzed the relationship between SDs and replication timing, focusing on lineage-specific, nonhomologous sequence elements. The genome was divided into 5 kbp bins, and replication timing signals were calculated for SDs and the genome-wide background. A one-sided Wilcoxon rank-sum test assessed the differences between SDs and the background. We found that ancient SDs shared across many species (e.g., BCHGO and BCHGOS) tend to replicate early (p-value < 1e-20), while more recent SDs (e.g., O and BC) and species-specific SDs tend to replicate late (p-value < 1e-50). Examples of the replication timing distribution are shown in **Supplementary Fig. XVII.55**.

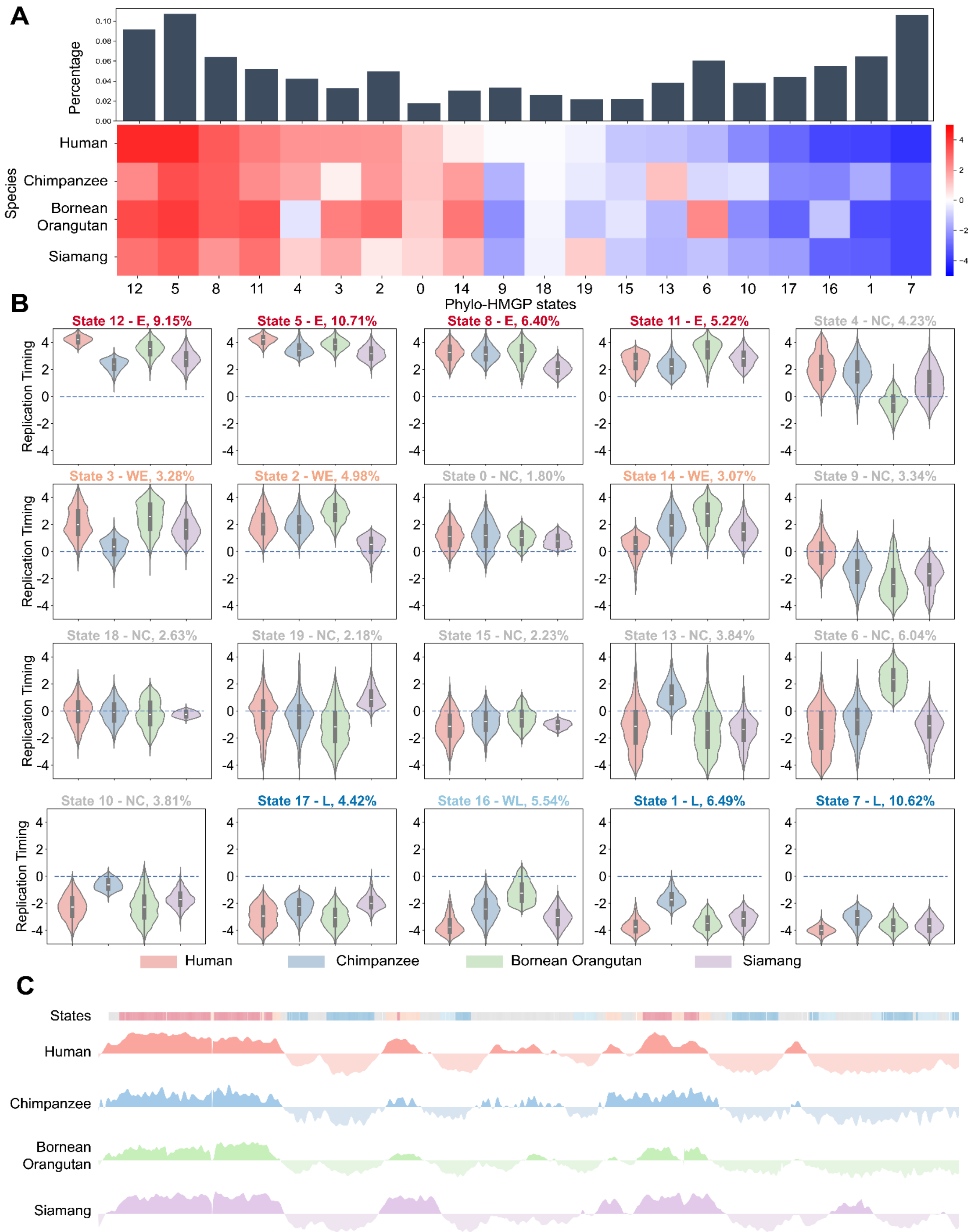

Supplementary Figure XVII.54. Phylo-HMGP identifies 20 states with distinct evolutionary patterns of replication timing in lymphoblastoid cells from four primary species. (A) The top panel shows the proportion of each state across the entire genome, while the bottom panel displays the average replication timing signals in each state, with columns representing different states ordered by the average human replication timing signals. (B) The 20 Phylo-HMGP states are categorized into five groups: early (denoted as E), weakly conserved early (WE), late (L),

weakly conserved late (WL), and non-conserved (NC). Violin and boxplots show the replication timing distributions for each state, organized by category. (C) Visualization of replication timing patterns and state annotations in the genome browser (chr1:50,000,000-75,000,000 in human T2T genome version 2.0).

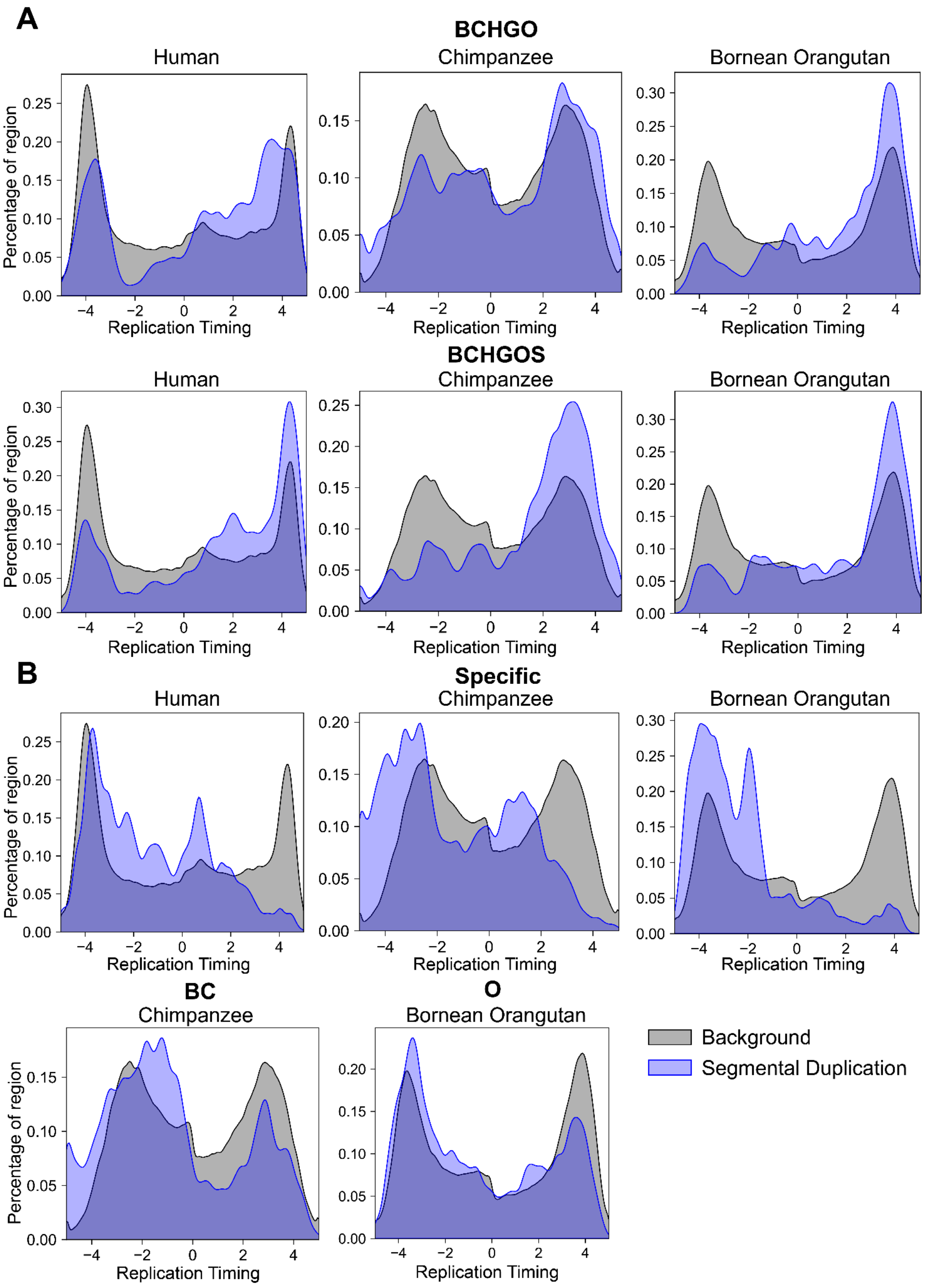

Supplementary Figure XVII.55. Correlation between segmental duplications (SDs) and replication timing (RT). The blue density curves represent the distribution of RT signals in SD regions, and the gray density curves represent the background RT signals. (A) Distribution of RT signals in more ancestral SDs, including BCHGO (shared among bonobo, chimpanzee, human, gorilla, and orangutan) and BCHGOS (shared among bonobo, chimpanzee, human, gorilla, orangutan, and siamang). (B) Distribution of RT signals in more recent SDs, including Specific (species-specific), BC (shared between bonobo and chimpanzee), and O (shared between two types of orangutans).

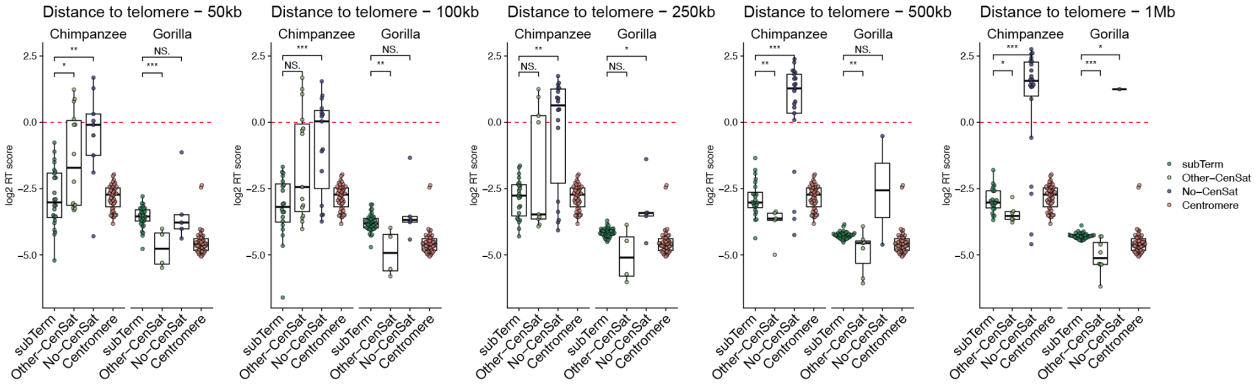

Supplementary Figure XVII.56. Boxplots showing the replication timing of telomeric regions of each chromosomal arm at varying distances from the chromosome ends (i.e., 50 kbp, 100 kbp, 250 kbp, 500 kbp, and 1 Mbp). The significance of p-values from the one-sided Wilcoxon test is indicated: * denotes p<0.05, ** denotes p<0.01, *** denotes p<0.001, NS. indicates nonsignificant results. The box and the horizontal line indicate interquartile range and median while whiskers indicate datapoints within 1.5-fold the interquartile range.

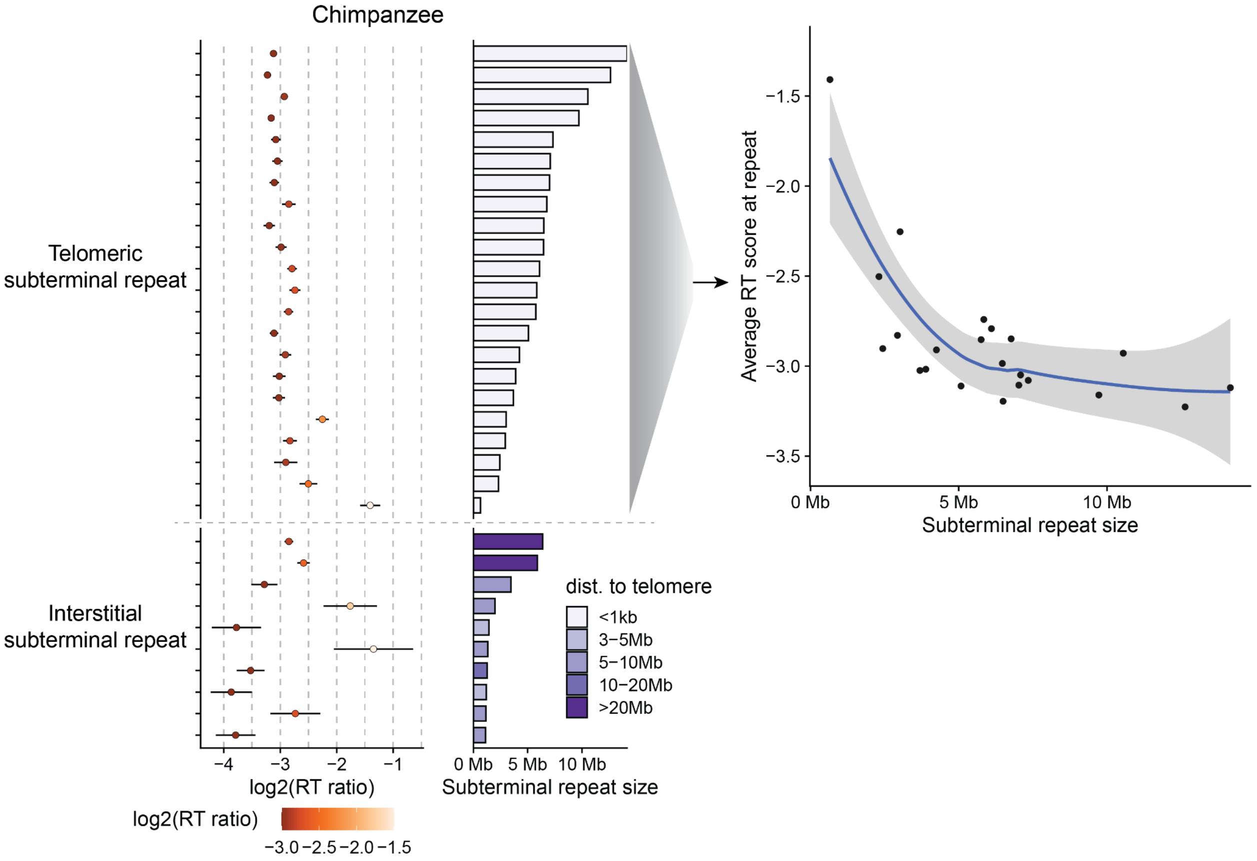

Supplementary Figure XVII.57. The dot plot on the left shows the replication timing of each subterminal repeat in chimpanzee. The horizontal line indicates the standard error of replication timing. Subterminal repeats are categorized into telomeric subterminal repeat (top) and interstitial subterminal repeat (bottom). The bar plot shows the length of each subterminal repeat, ordered by the size. The scatter plot on the right shows the relationship between the average

replication timing and the length of the telomeric subterminal repeats. The blue curve and gray shaded area are calculated using the LOESS smoothing method.

XVIII. Acrocentric region analysis

Contributing authors:

Steven J. Solar, Alexander P. Sweeten, Graciela Monfort Anez, Matthew Borchers, Tamara Potapova, Jennifer L. Gerton, Adam M. Phillippy

Methods

Identifying rDNA locations

To identify rDNA-containing chromosomes and array orientations, a reference human 45S unit (GenBank accession KY962518) was mapped against all primate genomes using mashmap v3.1.1:

```
mashmap -r $primate_ref -q 45S.fa -t $cpus --pi 90 -s 13332 --filter_mode none -o
$primate.45S.mashmap
```

The resulting mappings were manually validated, filtering out lower identity pseudogenes that were not part of intact arrays, and all chromosomes containing rDNA arrays were noted. These results were further confirmed by FISH.

Chromosome spreads and FISH

For the preparation of chromosome spreads, cells were blocked in mitosis by the addition of Karyomax colcemid solution (0.1 µg/ml, Life Technologies) for 6-7h and collected by trypsinization. Collected cells were incubated in hypotonic 0.4% KCl solution for 12 min and prefixed by addition of methanol:acetic acid (3:1) fixative solution (1% total volume). Pre-fixed cells were collected by centrifugation and then fixed in Methanol:Acetic acid (3:1). Spreads were dropped on a glass slide and incubated at 65°C overnight. Before hybridization, slides were treated with 0.1mg/ml RNAse A (Qiagen) in 2xSSC for 45 minutes at 37°C and dehydrated in a 70%, 80%, and 100% ethanol series for 2 minutes each. Slides were denatured in 70% formamide/2X SSC solution pre-heated to 72°C for 1.5 min. Denaturation was stopped by immersing slides in 70%, 80%, and 100% ethanol series chilled to -20°C. Labeled DNA probes were denatured separately in a hybridization buffer by heating to 80°C for 10 minutes before applying to denatured slides. Fluorescently labeled probe for human rDNA (BAC clone RP11-450E20) was obtained from Empire Genomics. Fluorescently labeled whole chromosome paints for chromosomes 2, 9, 13, 14, 15, 18, 21, and 22 were obtained from Applied Spectral Imaging. Human CenSat probe for D14Z1/D22Z1 was obtained from Cytocell. The probe for labeling distal junction (DJ) regions was prepared from the BAC CH251-351B7 (Eichler lab) and labeled with Biotin-16-dUTP using the nick translation kit (Enzo Life Sciences). Specimens were hybridized to the probes under a glass coverslip or HybriSlip hybridization cover (GRACE

Biolabs) sealed with the rubber cement or Cytobond (SciGene) in a humidified chamber at 37°C for 48-72hours. After hybridization, slides were washed in 50% formamide/2X SSC 3 times for 5 minutes per wash at 45°C, then in 1x SSC solution at 45°C for 5 minutes twice, and at room temperature once. For biotin detection, slides were incubated with streptavidin conjugated to Cy5 (Thermo) for 2-3 hours in PBS containing 0.5% Triton X-100 and 5% bovine serum albumin (BSA), and then washed 3 times for 5 minutes with PBS/0.5% Triton X-100. Slides were mounted in Vectashield containing DAPI (Vector Laboratories). Wide-field images were acquired on the Nikon TiE microscope equipped with 100x objective NA 1.4 and Prime 95B sCMOS camera (Photometrics). Z-stack images were acquired on the Nikon TiE microscope equipped with 100x objective NA 1.45, Yokogawa CSU-W1 spinning disk, and Flash 4.0 sCMOS camera (Hamamatsu).

Estimating rDNA copy number from FISH images

Image processing was performed in FIJI and Python. Primate rDNA-containing chromosomes were identified as homo sapiens (HSA) homologs based on the labeling with human chromosome paints and morphological features. For chimpanzee and bonobo, painting HSA 14 and HSA 21 was sufficient to identify all rDNA-containing chromosomes, and for gorilla, HSA 22 paint alone was sufficient. For Sumatran and Bornean orangutans, all rDNA-containing chromosomes were painted on separate slides, and these data were aggregated across all slides. For human HG002 spreads, labeling centromeric satellite 14/22 was sufficient to identify all rDNA-containing chromosomes. Chromosome Y was identified by morphology.

For manual image quantifications performed for chromosome spreads from chimpanzee, bonobo and gorilla cells, sum intensity projections of confocal Z-planes were generated, and individual rDNA arrays were segmented based on threshold applied to the entire image. The fluorescence intensity of the regions of the same chromosomes that did not contain the rDNA was used to subtract the local background. The background-subtracted integrated intensity was measured for each array. For semi-automated quantification performed for chromosome spreads from Sumatran orangutan, Bornean orangutan, and human HG002 cells, wide-field single Z-plane images were used. rDNA-containing chromosomes were segmented using a Cellpose model trained on 2-channel images, including the DAPI and rDNA signals. rDNA regions were also segmented using a trained Cellpose model. The chromosome segmentations were examined and, if necessary, curated manually in Napari. rDNA intensities for each array were measured after subtracting the fluorescence background for the respective chromosomes. Custom Python and scripts are deposited in GitHub: https://github.com/jouyun/2024_Primate_rDNA.

The sum of all intensities of all rDNA loci represented the total amount of rDNA per cell, and the fraction of this total signal was calculated for each rDNA array. The total rDNA copy number was estimated from Illumina sequencing data (see "Estimating rDNA copy number from *k*-mer coverage"). The fraction of the total rDNA fluorescence intensity was used as a proportion

of the total rDNA copy number to determine the number of rDNA copies on specific chromosomes in each chromosome spread.

Estimating rDNA copy number from *k*-mer coverage

Genomic DNA from primate cell lines was isolated using QIAamp DNA Micro Kit (Qiagen) according to the manufacturer's protocol. PCR-free DNA-seq libraries were constructed using NEBNext Ultra II DNA Library Prep Kit and sequenced on AVITI System (Element Biosciences) outputting 150 bp paired-end reads. Data for HG002 was obtained from Baid et al.[182], using the PCR-free 40× coverage whole-genome sequencing sample. rDNA copy numbers were estimated from *k*-mer frequencies in the whole-genome sequencing data. A reference 18S sequence was set for each species by choosing a single representative unit from rDNA loci identified by mashmap. The 18S copy number served as a proxy for the greater 45S unit, as each unit contains a single 18S segment. A custom pipeline counted *k*-mers of size 31 from the 18S consensus in short-read Illumina sequencing data and normalized it to counts of 31mers from G/C matched windows elsewhere in the rDNA containing chromosomes. The matched windows were of similar size to the 18S, and 30 were randomly selected per rDNA-containing chromosome. Any *k*-mers that also occurred outside the matched windows were removed to ensure that counts were exclusively from the matched windows. *k*-mer sets were filtered to remove those with whole-genome sequencing counts greater than three standard deviations from the mean of the set, or those that were missing entirely, and counts were divided by their genomic multiplicity. Finally, the median count from the 18S *k*-mers was divided by the median count of the matched windows to yield a copy number approximation. Three replicates of this process were done for each primate, as the G/C matching step had a random component, and the integer-rounded mean of the three replicates was taken as the copy number. A pipeline referred to as CONKORD (version 7) was used for this process. A slightly different version of CONKORD was used for HG002, which did not filter *k*-mers at high standard deviations or zero counts (version 5). CONKORD contains a set of custom Python and Bash scripts, which are deposited in GitHub (https://github.com/borcherm/primate_rdna_cn).

Plotting acrocentric short arms

To dot plot the short arms, the short arms of all chromosomes seen containing an NOR in at least one haplotype were extracted:

```
samtools faidx $primate_ref:$chr:1-$cen_start > $primate.$chr.p_arm.fa
```

For the purposes of plotting, the 1 Mbp rDNA gap in each assembly was replaced with the human reference rDNA unit duplicated as many times as indicated by the combined FISH and Illumina copy number quantification. These new sequences were then plotted with ModDotPlot v0.8.4 using scripts in the linked GitHub, where the axis limits were set to just shy of 55 Mbp

based on the largest rDNA-gap-filled HSA22 short arm, which was mPanPan1 chr23_mat_hsa22.

To quantify satellite compositions, BED files were defined denoting the short arms of all the chromosomes where at least one haplotype contained an NOR in that primate, then used to filter the satellite annotation BED files to only contain records up to the start of the centromere. Next, for each satellite class total base pairs were counted from these files. rDNA bases were defined by multiplying the total copy number per species by 45 kbp, an estimated length of an rDNA unit. This is inexact, given rDNA units vary in size within and across species and individuals, and is meant to be taken as an estimate.

Quantifying short arm synteny

To quantify syntenic and non-syntenic bases on the acrocentric chromosomes, each T2T-CHM13 acrocentric (chr22 shown in **Supplementary Fig. XVIII.61**) was mashmapped to each of the primate haplotypes separately, and all hits within 1% of the best hit were retained.

```
mashmap -q chm13.$chr.fa -r $primate_ref -t $cpus -M --pi 80 -s 10000 --filter_mode
none -o $primate.chm13_$chr.mashmap
```

Then colors were assigned according to what each CHM13 segment hit in the primates based on the key in the figure. Siamang was not included in this analysis due to its mosaic synteny relative to human chromosomes. Both haplotypes' mappings were combined to assess syntenic and non-syntenic bases on the short and long arms.

Hits to multiple haplotypes of the same chromosome were combined, and then each window was checked for all its hits within 1% of the best mapping. Next, colors were assigned according to the list of best hits based on the key in the figure. If the segment singly mapped to a chromosome that is acrocentric in humans, it was colored accordingly. A single-mapper to a non-acrocentric was colored black. Multimappers were colored light tan if all best hits were to acrocentric chromosomes, and brown if any hits were to non-acrocentric chromosomes. Siamang was not included as described above. Both haplotypes' mappings were combined to assess syntenic and non-syntenic bases on the short and long arms.

Identifying distal junctions

To identify distal junction (DJ) locations and orientations in the primate assemblies, a reference DJ was extracted from T2T-CHM13 as defined by Sluis et al.[183] extending from the end of the CER block at T2T-CHM13v2.0 chr13:5,424,523 to the beginning of the rDNA array at chr13:5,770,548. This reference DJ was aligned to the primate assemblies using minimap2 v2.28 and filtered for hits ≥ 100 kbp:

This process was repeated aligning the DJ just chrY to look for possible remnant DJs using the same commands.

The DJ palindrome was identified by dot plotting the reference DJ using MUMmer v4.0.0 and manually identifying the maxmatches that defined the start and end of each palindromic arm (chr13:5,436,542-5,549,309 and chr13:5,555,214-5,670,026).

A second gorilla NOR on both haplotypes of HSA22 was identified by this DJ mapping and confirmed by viewing the inverted duplication with ModDotPlot. To assess rDNA copy number and activity in the second NOR, the mapped human 45S location was viewed in IGV along with ONT read alignments containing methylation data. Notably, only a single arm of this palindrome was identified in siamang, with the orientation of these sequences inverted between the two haplotypes.

Analyzing rDNA unit conservation

To assess rDNA conservation across species, representative units were extracted from the assemblies. The human 45S gene was minimapped to the assembly. Then, the sequence from the start of one 45S to the start of the next was extracted using BEDTools (v2.29.0) in each primate and flipped when necessary to match the transcriptional direction, to serve as a representative unit, acknowledging that intraspecies variation will exist. 45S and intergenic spacer (IGS) sequences were extracted using the coordinates from the original alignment. Multiple sequence alignments of rDNA units, 45S sequences, and the IGS were generated using Mafft-linsi v7.526. This was used to compute pairwise gap-excluded percent identities for each region with a simple python script.

rDNA units were dot plotted against each human consensus sequence and themselves using ModDotPlot v0.8.4 with the following command:

```
moddotplot static --compare -r 100 -a 45000 -f $rdna_1 $rdna_2 -o
$species_1.$species_2
```

As expected, the 45S gene looks most highly conserved, with more divergence in the IGS.

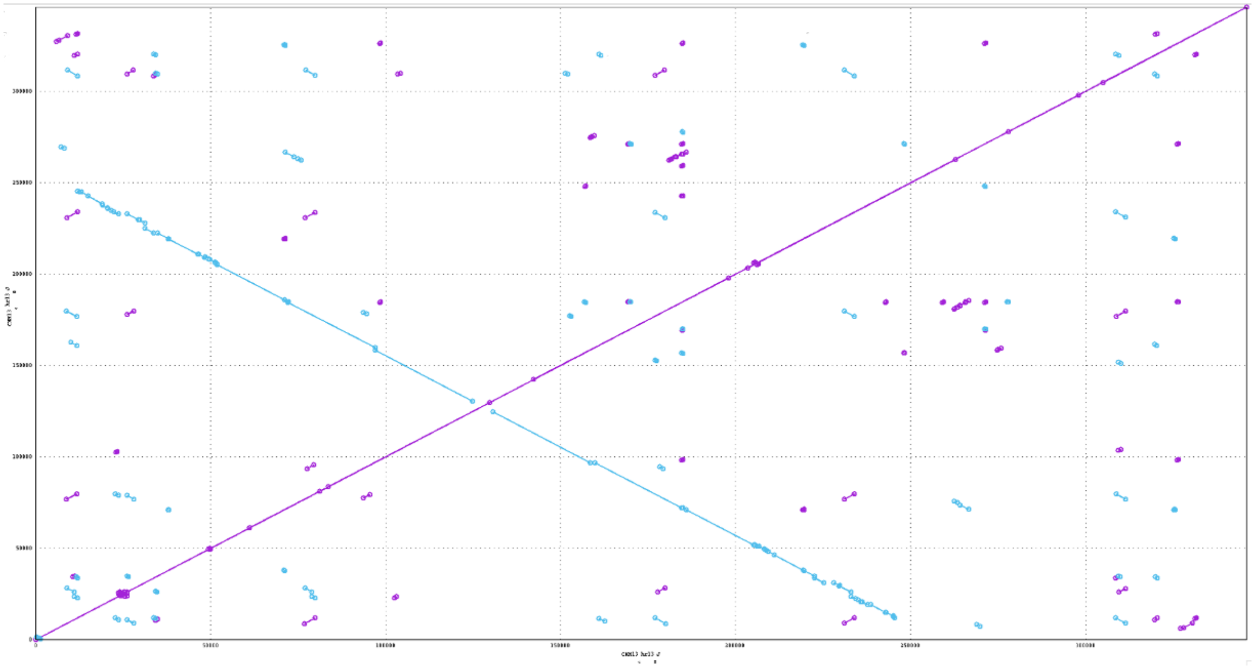

Supplementary Figure XVIII.58. Structure of the human distal junction (DJ). A plot of maximal exact self-matches of at least 20 bp in the CHM13 chr13 DJ. Forward matches are indicated in purple and reverse matches in blue. The large X shape indicates the presence of an inverted repeat, in this case the characteristic DJ palindrome encoding the long ncRNA. The precise boundaries of this palindrome were extracted.

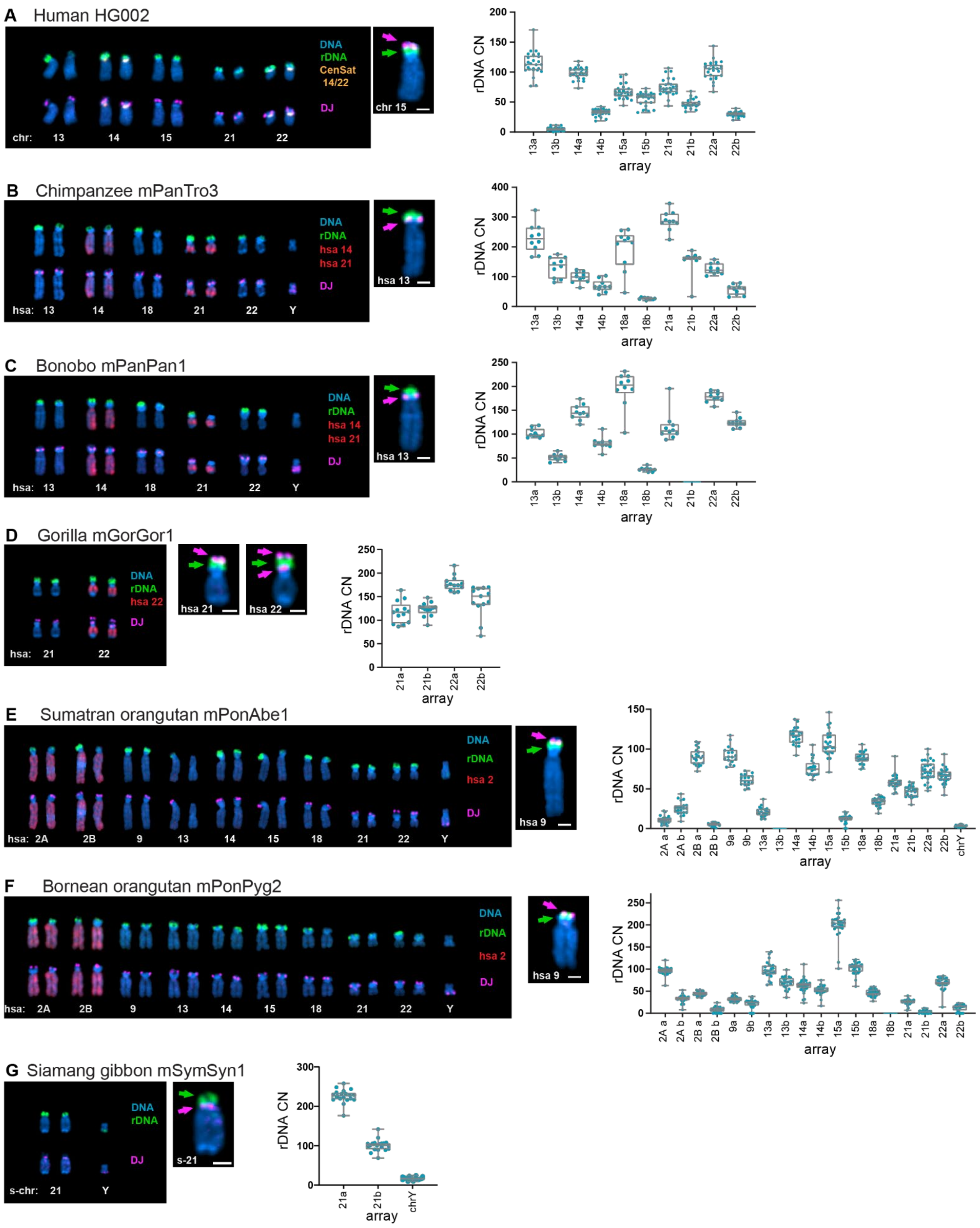

Supplementary Figure XVIII.59. Representative karyograms of NOR+ chromosomes in cells from human (A), chimpanzee (B), bonobo (C), western lowland gorilla (D), Sumatran orangutan (E), Bornean orangutan (F) and Siamang gibbon (G). Primate chromosomes were identified as homo sapien (HSA) homologs except Siamang-specific rDNA-containing chromosome 21. The

top chromosome rows show FISH labeling with the rDNA probe (green) and chromosome identification markers. For chromosome identification, human CenSat 14/22 probe (orange) or indicated human whole chromosome paints (red) were used. The bottom rows show labeling with the DJ-region probe (magenta). DNA was counter-stained with DAPI. Side panels show overlayed images of representative individual chromosomes. Corresponding quantifications of rDNA copy number are shown on the right. The boxes represent the IQR, with the edges indicating the upper and lower quartiles. The line inside the boxes indicates the median. Whiskers show the range from minimum to maximum values. Ten or more spreads were quantified for each specimen. All individual data points are shown. Estimated total numbers of rDNA units and rDNA units per haplotype for each species are listed in **Supplementary Table XVIII.78**.

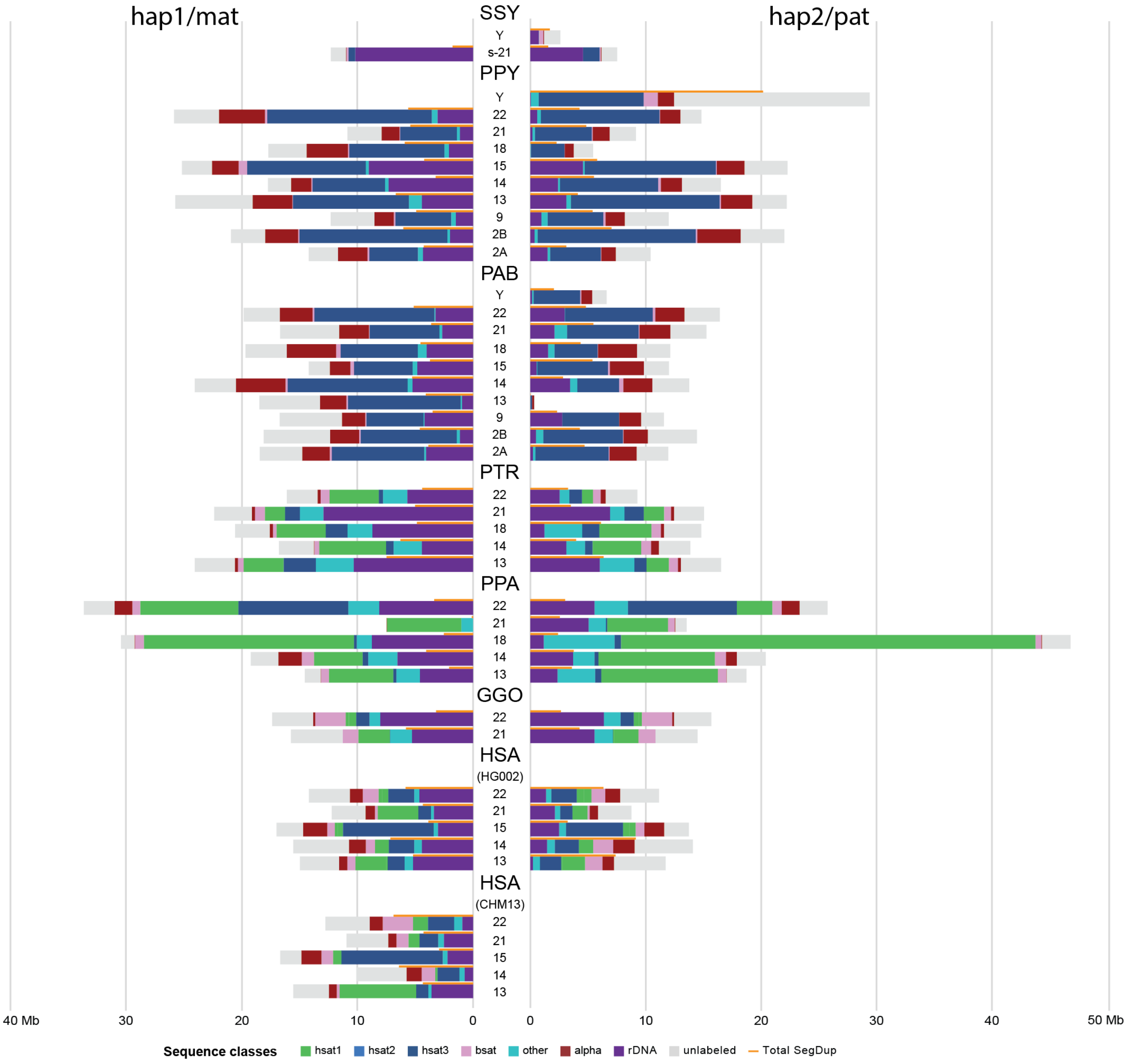

Supplementary Figure XVIII.60. Satellite content of all short arms. Sum of satellite and rDNA sequence across each short arm where one haplotype is NOR+ in each species. "Unlabeled" indicates sequences without a satellite annotation, which mostly comprise SDs. Total SD bases are given for comparison, with some overlap between regions annotated as SDs and satellites. Total satellite content on haplotypes of the same chromosome can vary as much as 22.5 Mbp (PPA HSA18), or as little as 100 kbp (PPY HSA21). Of the two truncated short arms, one appears much shorter than the other (PAB HSA13, 1.2 Mbp vs. PPA HSA21, 7.5 Mbp) mainly due to large HSAT1A expansions on the bonobo truncated HSA21.

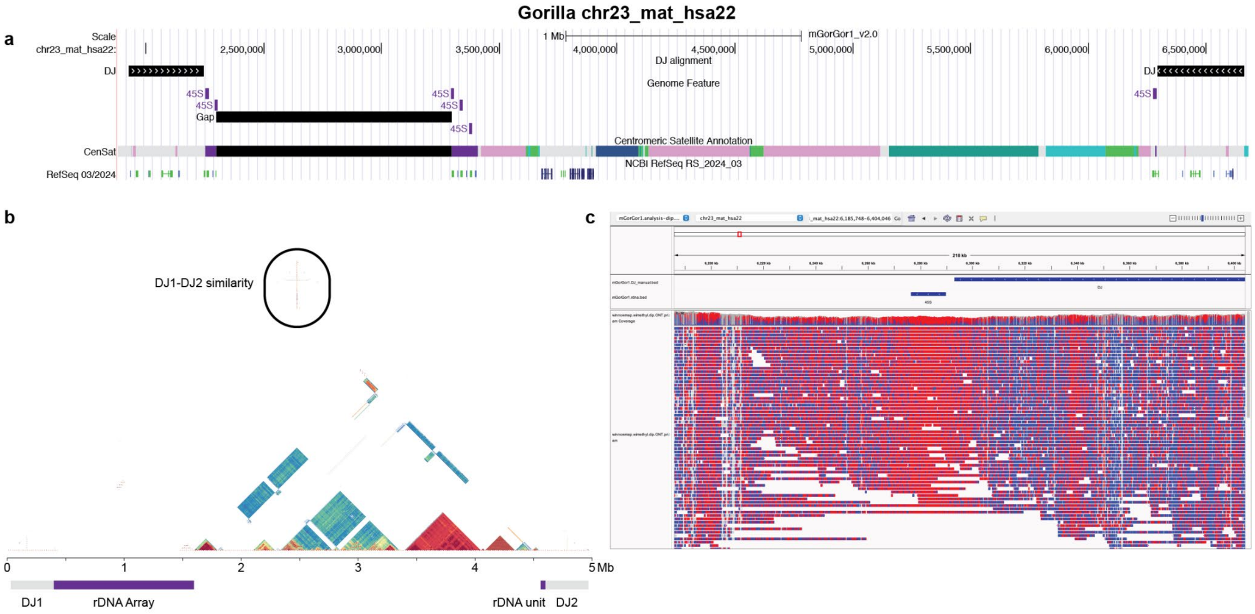

Supplementary Figure XVIII.61. NOR structure of gorilla HSA22. (a) Viewing of alignments of the human DJ and 45S gene to gorilla's maternal HSA22 indicates the presence of two NORs. One faces toward the centromere (as in HSA21) and contains a full rDNA array; the other faces towards the telomere and contains a single rDNA unit. This is also the case in the paternal haplotype. (b) A self-ModDotPlot of the same region shows high similarity between the two DJs (labeled DJ1 and DJ2). (c) Whole-genome alignments of ONT reads show that the single rDNA unit associated with DJ2 is hypermethylated (red) in both haplotypes, which is known to indicate inactivity[1].

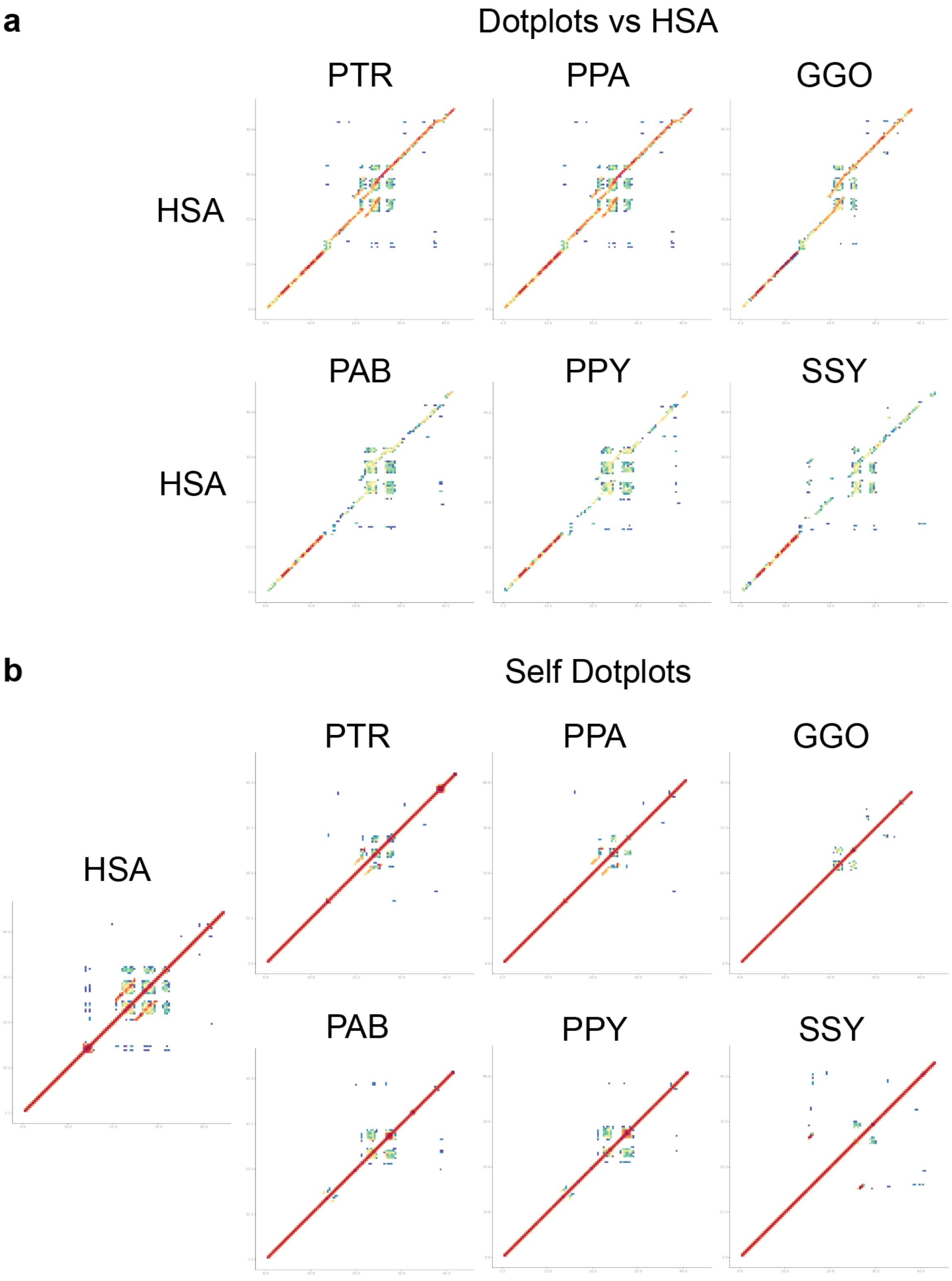

Supplementary Figure XVIII.62. Structure of representative rDNA units. (a) rDNA units from each primate were compared to the human reference rDNA unit KY962518 with ModDotPlot (https://www.ncbi.nlm.nih.gov/nuccore/KY962518) to identify similarities in structure.
(b) rDNA units from each primate were self-dot plotted to identify satellites and internal repeat structures.

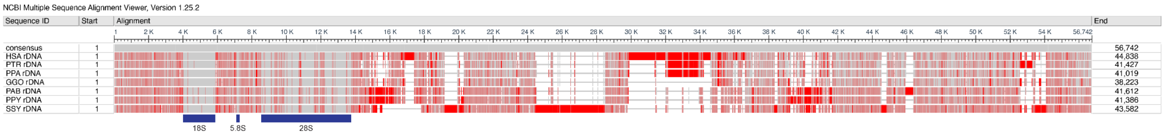

Supplementary Figure XVIII.63. Interspecies variation within the rDNA. Multiple sequence alignment of the representative rDNA units for each species generated with MAFFT shows high conservation within the genic regions (particularly the 18S and 5.8S) relative to the intergenic spacer, which shows significant structural variation.

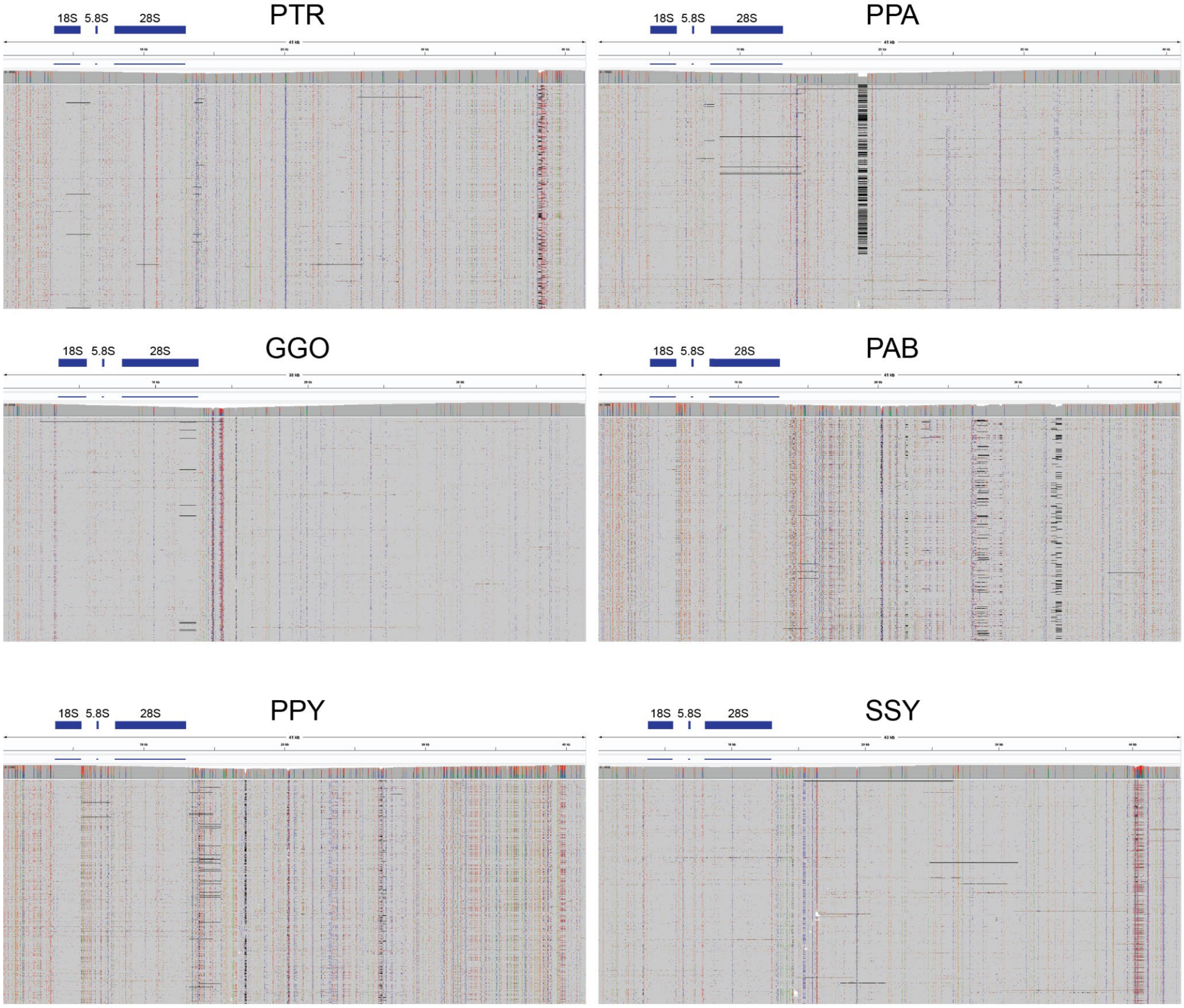

Supplementary Figure XVIII.64. Intraspecies variation within the rDNA. rDNA-specific HiFi reads were extracted for each species and aligned to the representative rDNA unit. Again, the genic regions, particularly the 18S and 5.8S, show higher conservation relative to the intergenic spacer. Certain SNVs and SVs appear relatively common in the population of rDNA units, as does variation in the size of CT-microsatellites.

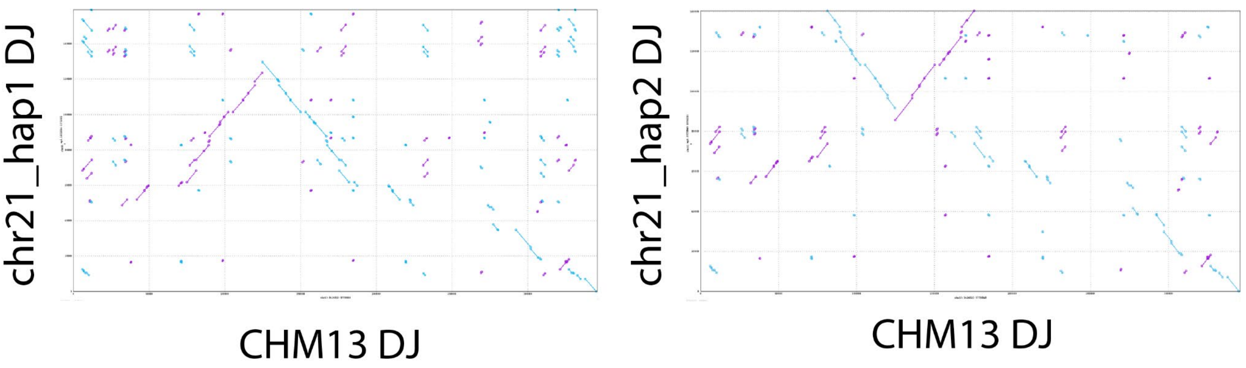

Supplementary Figure XVIII.65. Comparison of two gibbon DJs to human reference. The gibbon DJs on both haplotypes of chr21 were extracted. A plot of maximal exact matches to human of at least 20 bp indicates that gibbon has polymorphically lost one arm of the DJ palindrome. With human CHM13 chr13 DJ on the x-axis, the dot plots indicate that chr21_hap1 retained the first arm of the palindrome, while chr21_hap2 retained the second arm.

XIX. Centromere analyses

Contributing authors:

Glennis A. Logsdon, Hailey Loucks, Karen Miga

Methods

Centromere identification and annotation

To identify the centromeric regions within each NHP genome, we first aligned the whole-genome assemblies to the T2T-CHM13v2.0 reference genome[184] using minimap2 (v2.24)[70] with the following parameters: -I 10G -a --eqx -x asm20 -s 5000. We filtered the alignments to only those regions that traversed each human centromere, from the p- to the q-arm, using SAMtools (v1.9)[102] and then ran RepeatMasker (v4.1.0)[79] to identify regions containing α-satellite sequences, marked by "ALR/Alpha". Once we identified the regions of the assemblies containing α-satellite repeats, we ran Hum-AS-HMMER (https://github.com/fedorrik/HumAS-HMMER_for_AnVIL) using the hmmer-run_SF.sh script and the AS-SFs-hmmer3.0.290621.hmm Hidden Markov Model. This generated a BED file with each α-satellite suprachromosomal family (SF) designation and its organization along the centromere. We used the α-satellite SF BED file to visualize the organization of the α-satellite higher-order repeat (HOR) arrays with R (v1.1.383)[185] and the ggplot2 package[125].

Validation of centromeric regions

We validated the construction of each centromeric region by first aligning native PacBio HiFi and ONT data from the same source genome to each whole-genome assembly using pbmm2 (v1.1.0) (for PacBio HiFi data; https://github.com/PacificBiosciences/pbmm2) or Winnowmap (v1.0) (for ONT data)[175]. We, then, assessed the assemblies for uniform read depth across the centromeric regions via IGV[186] and for collapses, duplications, and misjoins via NucFreq[187]. Centromeres that were found to have a misassembly were flagged and are indicated in the figures.

Estimation of α-satellite HOR array length

To estimate the length of the α-satellite HOR arrays of each centromere in the NHP genome assemblies, we first ran Hum-AS-HMMER (https://github.com/fedorrik/HumAS-HMMER_for_AnVIL) on the centromeric regions using the hmmer-run_SF.sh script and the AS-SFs-hmmer3.0.290621.hmm Hidden Markov Model. Then, we used the α-satellite SF BED file to calculate the length of the α-satellite HOR arrays by taking the minimum and maximum coordinate of continuous stretches of α-satellite SFs and plotting their lengths with GraphPad Prism (v9).

Pairwise sequence identity heatmaps

To generate pairwise sequence identity heatmaps of each centromeric region, we ran StainedGlass (v6.7.0)[188] with the following parameters: window=5000, mm_f=30000, and mm_s=1000. We normalized the color scale across the StainedGlass plots by binning the % sequence identities equally and recoloring the data points according to the binning.

CpG methylation analysis and CDR definition

To determine the CpG methylation status of each NHP centromere, we aligned ONT reads >30 kbp in length from the same source genome to the relevant whole-genome assembly via Winnowmap (v1.0) and then assessed the CpG methylation status of the centromeric regions with Epi2me modbam2bed (https://github.com/epi2me-labs/modbam2bed; v0.10.0) and the following parameters: -e -m 5mC --cpg. We converted the BED file to a bigWig using the bedGraphToBigWig tool (https://www.encodeproject.org/software/bedgraphtobigwig/) and then visualized the file in IGV. To determine the size of hypomethylated region (termed "centromere dip region", or CDR[189] in each centromere, we used CDR-Finder (https://github.com/arozanski97/CDR-Finder). This tool first bins the assembly into 5 kbp windows, computes the median CpG methylation frequency within windows containing α-satellite (as determined by RepeatMasker (v4.1.0)), selects bins that have a lower CpG methylation frequency than the median frequency in the region, merges consecutive bins into a larger bin, filters for merged bins that are >50 kbp, and reports the location of these bins.

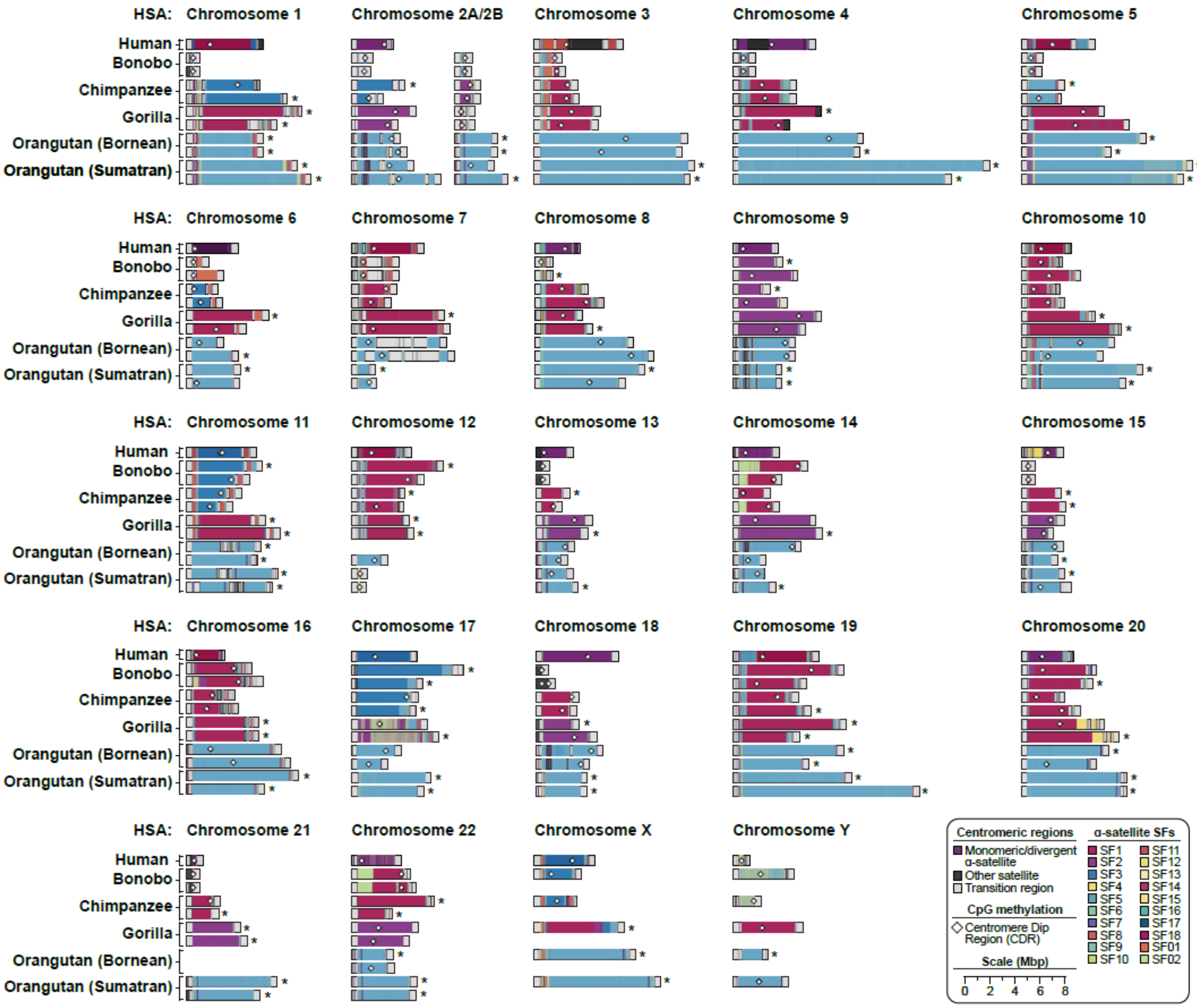

Supplementary Figure XIX.66. Sequence and structure of 237 contiguous centromeres from five NHPs. Maps of the active α-satellite HOR arrays from the human (CHM13), bonobo, chimpanzee, gorilla, Bornean orangutan, and Sumatran orangutan chromosome centromeres, with the α-satellite suprachromosomal family (SF), indicated for each centromere. Centromeres with an error in their assembly are indicated with an asterisk. Assemblies without any errors have the location of the centromere dip region (or CDR) indicated with a white diamond.

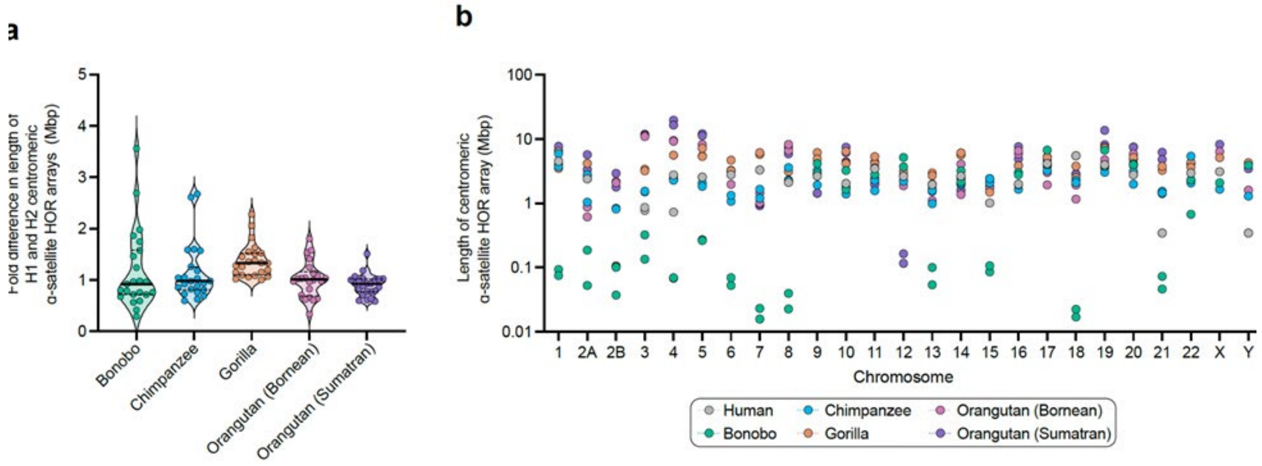

Supplementary Figure XIX.67. Haplotypic and cross-species variation in centromeric α-satellite HOR array lengths. (a) Fold difference in α-satellite HOR array lengths between haplotypes for each NHP. (b) Differences in α-satellite HOR array lengths for each chromosome among human and five NHPs. Chromosomes are named according to HSA nomenclature to permit cross-species comparisons, and the lengths of the α-satellite HOR arrays are shown on a log scale.

XX. Subterminal satellite

Contributing authors:

DongAhn Yoo, Evan E. Eichler

Methods

Subterminal satellites or pCht repeats present in African great ape species (chimpanzee, bonobo and gorilla) were identified using BLASTN (v2.12.0)[95] with the consensus sequence (len = 32bp): "gatatttccatgtttatacagatagcggtgta". The blast hit with longer than 90% of the consensus (>28 bp) was recovered. The individual pCht unit was classified into different types based on the variants (small INS, DEL or substitution). The siamang genome, which contains subterminal α-satellite, was investigated using RepeatMasker (v4.1.5)[79].

In addition to the subterminal satellites, spacer SDs interrupting the satellites were investigated. This was done by subtracting the subterminal satellite arrays from the entire subterminal satellites regions obtained by "*bedtools merge -i [pCht/α-satellite] -d 1000000*". Size distribution was obtained from subtraction of satellites loci from the merged region blocks. Examining distribution of the spacer sequences and their size, 32 kbp highly conserved sequence was identified in *Pan* lineage and 34 kbp independently in gorilla; on the other hand, multiple modal lengths were identified for siamang spacer with 57 kbp spacers being the most abundant. Using a copy of spacer sequence, spacers were identified using BLASTN (n=793, 800 in chimpanzee and bonobo, and n=974 in gorilla). Methylation status of spacer and subterminal satellites were investigated via ONT long-read alignment against each of the ape genome assembly using Epi2me modbam2bed (https://github.com/epi2me-labs/modbam2bed; v0.10.0; "*-e -m 5mC --cpg*".

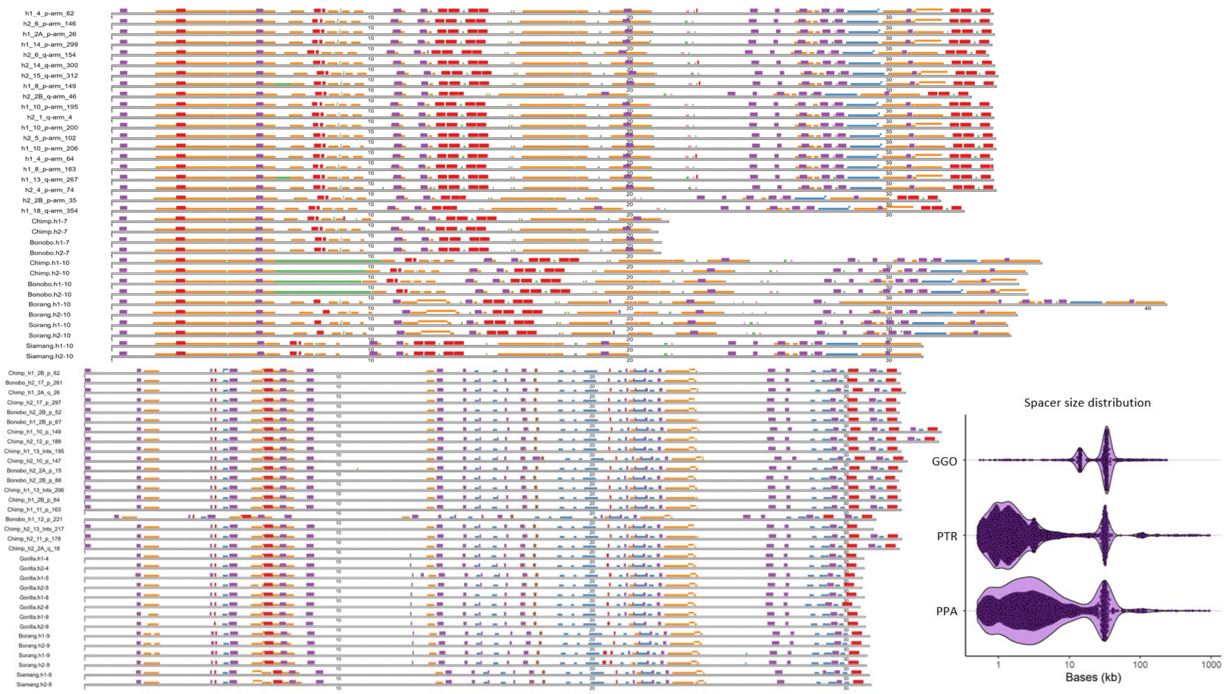

Supplementary Figure XX.68. Sequence organization of the subterminal spacer SDs and their ortholog copies. The top shows the stacked plot of gorilla spacer SDs, followed by the ancestral orthologous/paralogous copies of other species below. Below is the subterminal *Pan* lineage spacer SDs followed by the orthologous/paralogous copies of other species. The color scheme represents different classes of repeats; red: LTR, blue: DNA, green: simple repeat, purple: SINE, orange: LINE, yellow: single recognition particle RNA, brown: retrotransposon, gray: low complexity. The bottom right shows the distribution of the spacer SD sizes identified across the diploid genomes.

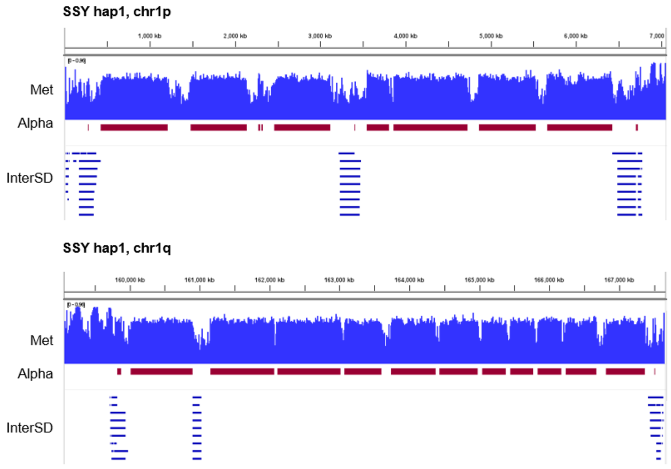

Supplementary Figure XX.69. Epigenetic property of spacers, forming hypomethylated pockets, similar to African great ape spacers. The example Chr1 p- and q-arm spacer SDs are shown as the genome browser format. For each view, top shows the CpG methylation track indicating % of CpGs in the locus methylated; below that shows α-satellite arrays indicated by the red track followed by interchromosomal SDs.

XXI. Segmental duplications

Contributing authors:

DongAhn Yoo, David Porubsky, Hyeonsoo Jeong, Evan E. Eichler

Methods

SDs were called via SEDEF (v1.1)[190] based on soft-masked genome assemblies - TRF v.4.1.0[81], RepeatMasker v.4.1.5 [79], and WindowMasker (v2.2.22)[191]. The SD calls with sequence identity >90%, length >1 kbp, and satellite content <70% were kept. Lineage-specific SDs were defined by chaining SDs within 100 kbp distance and comparing the putative homologous SD loci, defined as containing 100 kbp syntenic sequence flanking the SD. In addition, the SDs that are homologous by location were further checked for the contents using pairwise alignment (minimap2 v2.26)[70]. The SDs with sequence content (coverage >20%) changed were considered as specific, and SDs with expanded length (>2-fold) were identified. Thus, the SDs with 1) no homologous SDs of other species by position, 2) sequence content changed, and 3) expanded size were quantified. Homozygous and heterozygous genotypes were determined by comparing the two haplotypes. Polymorphic SDs within congeneric species were also investigated by making four-way comparisons for *Pan* and *Pongo* lineages. To check if the copy number of genes located in polymorphic SDs are also variable in additional ape genomes, we utilized Illumina short-read whole-genome sequencing data[2] processed by a previous study[115]. Homologous SDs shared by different apes were classified into phylogenetic branches based on maximum parsimony. The correlation of SDs (Mbp) versus branch length (mya) was computed using linear regression, excluding siamang and macaque, which contain significantly lower number of SDs compared to other species (two-tailed Wilcox ranked sum test) and BCHGOS ancestral node affected by siamang. Examining Cook's distance also identifies significant deviation (D>4/n) in the nodes.

Candidate lineage-specific genes expanded for chr1 GGO double inversion and *Pongo* chr16 expansions were identified by aligning of the gene copies using minimap2 (v2.26) (*-cx asm20 -f 5000 -k15 -w10 -p 0.05 -N 200 -m200 -s200 -z10000 --secondary=yes –eqx*) to find the mapping with >75% of query sequence coverage and >75% of percentage identity. We further screened for Iso-Seq support (indicating read count > 3) and assessment of ORF whether peptide sequence predicted by TransDecoder (https://github.com/TransDecoder/TransDecoder) covers at least >75% of the blastp best hit peptide sequence and percentage identity >80% (the longest transcript). Divergence time among the variable copies was estimated by aligning the complete gene sequence using MAFFT (v7.525)[118] and least square dating of IQTREE2 (v 2.1.2)[126] using chimpanzee–human divergence of 6.4 mya.

Visualization of the alignment of lineage-specific SDs in chr1 GGO double inversion and *Pongo* chr16 expansions was done by alignment of sequences using a PAF file generated by minimap2 (v2.26; options *"-secondary=no -x asm20 -c –eqx"*), followed by breaking of the sequence

alignment blocks containing insertions/deletions larger than 100 bp in size using Rustybam ("*rb break-paf -m 100*"), with the SVbyEye R package (https://github.com/daewoooo/SVbyEye)[124] on the resulting PAF file.

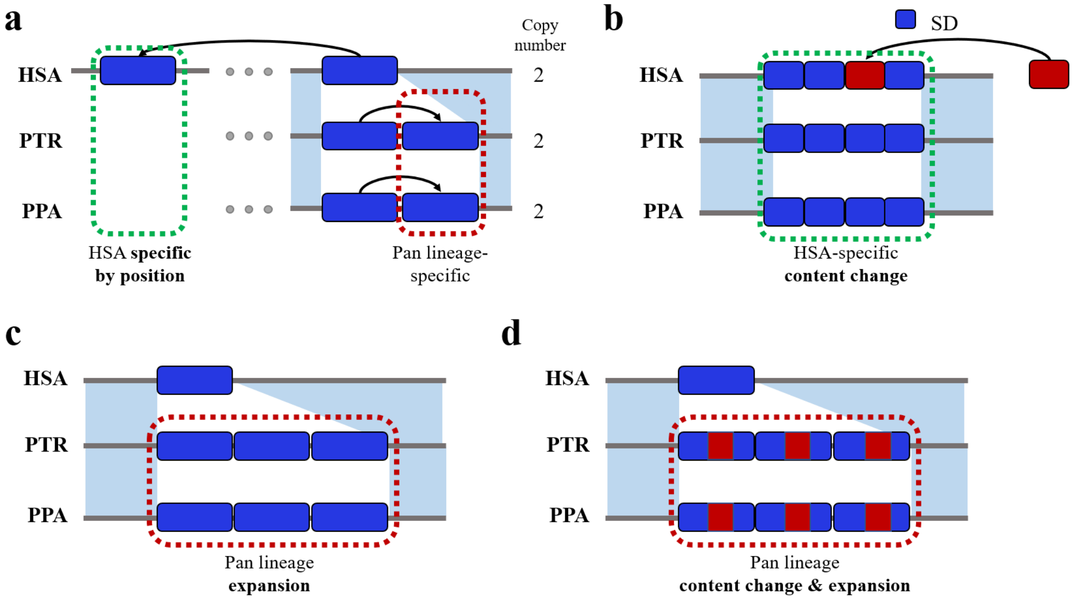

Supplementary Figure XXI.70. Examples of lineage-specific SDs. (a) location, (b) content, (c) length, and (d) both content and length.

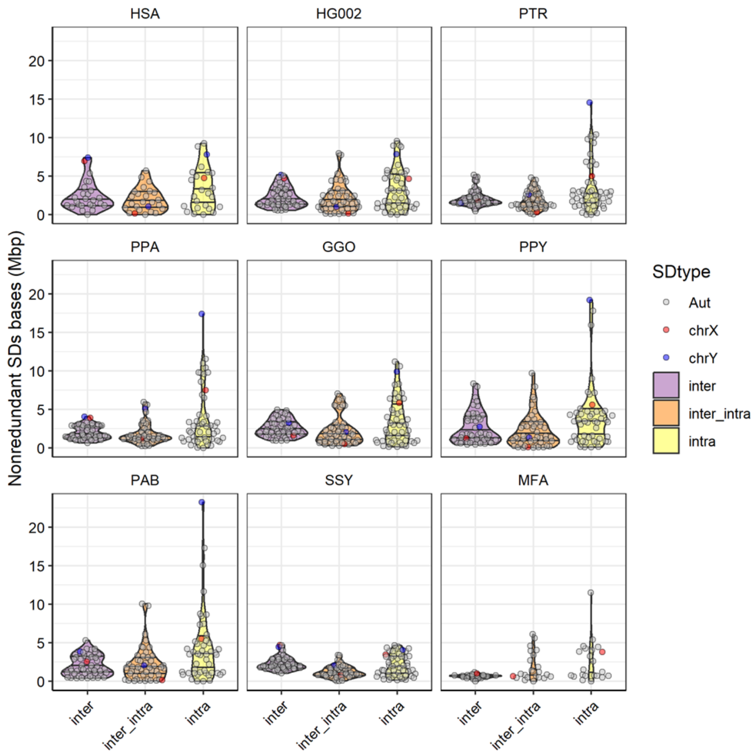

Supplementary Figure XXI.71. Comparison of SD content in sex chromosomes and autosomes.

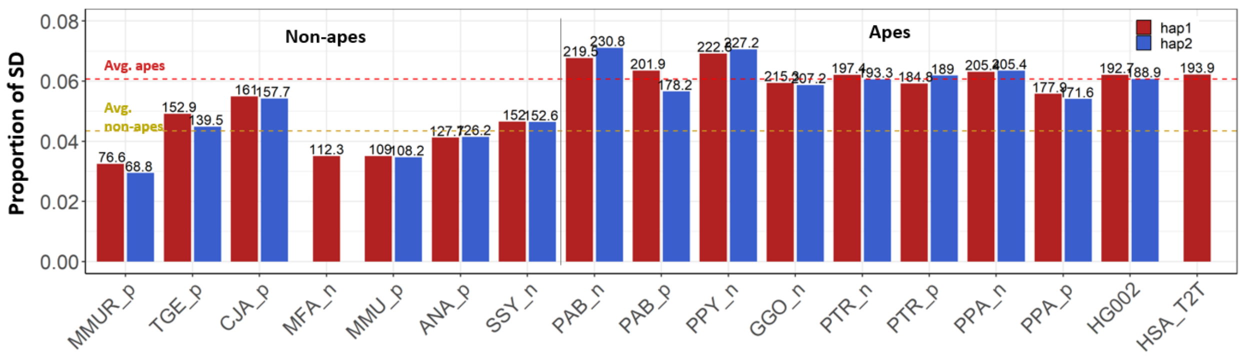

Supplementary Figure XXI.72. Total number of SD bases across apes and non-apes.

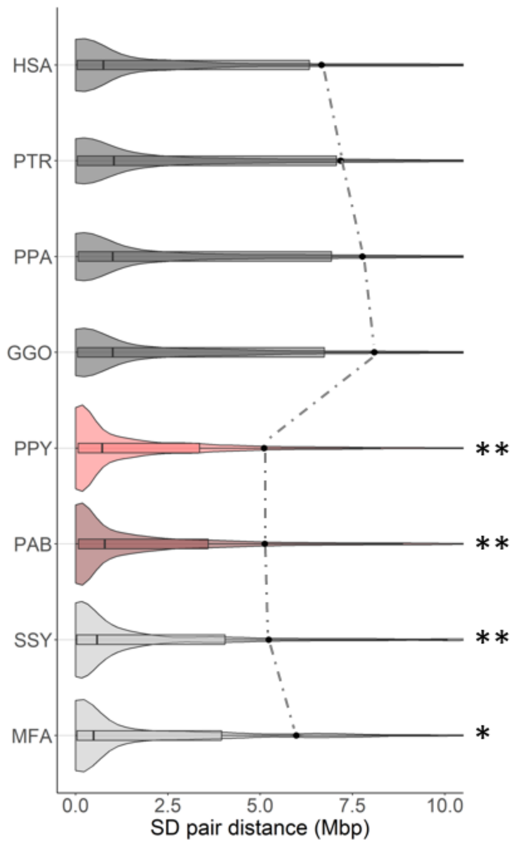

Supplementary Figure XXI.73. A violin plot distribution of pairwise SD distance to closest paralog. The median (black line) and mean (dashed line) are compared for different apes. The box indicates interquartile range. One-sided Wilcoxon rank sum test against human as the reference showed p-value of 1.0 for PTR, PPA, and GGO while showing $<2.2\times10^{-16}$ for PPY, PAB and SSY and 1×10^{-4} for MFA (pair of SDs, n = 17800, 17703, 21313, 19211, 19979, 21066, 13161 and 8543 for HSA, PTR, PPA, GGO, PPY, PAB, SSY and MFA, respectively).

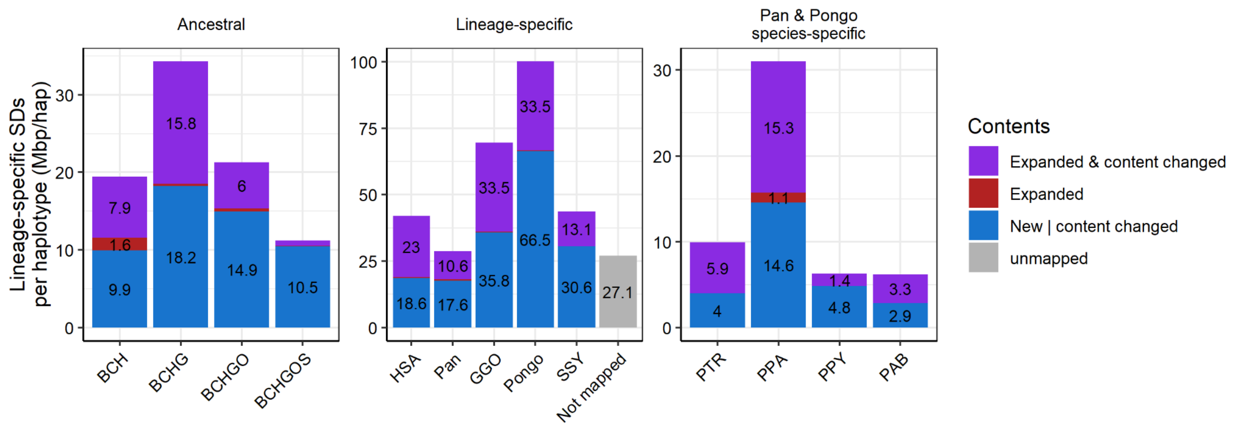

Supplementary Figure XXI.74. Summary of average lineage-specific SDs detected per haplotype. Note the quantification excludes acrocentric SDs. The left panel shows ancestral SDs that are shared among apes, representing the inner ancestral nodes. The center panel summarizes lineage-specific SDs. Species-specific SDs for *Pan* and *Pongo* are shown on the right panel, separately due to relatively short divergence times to latest common ancestors.

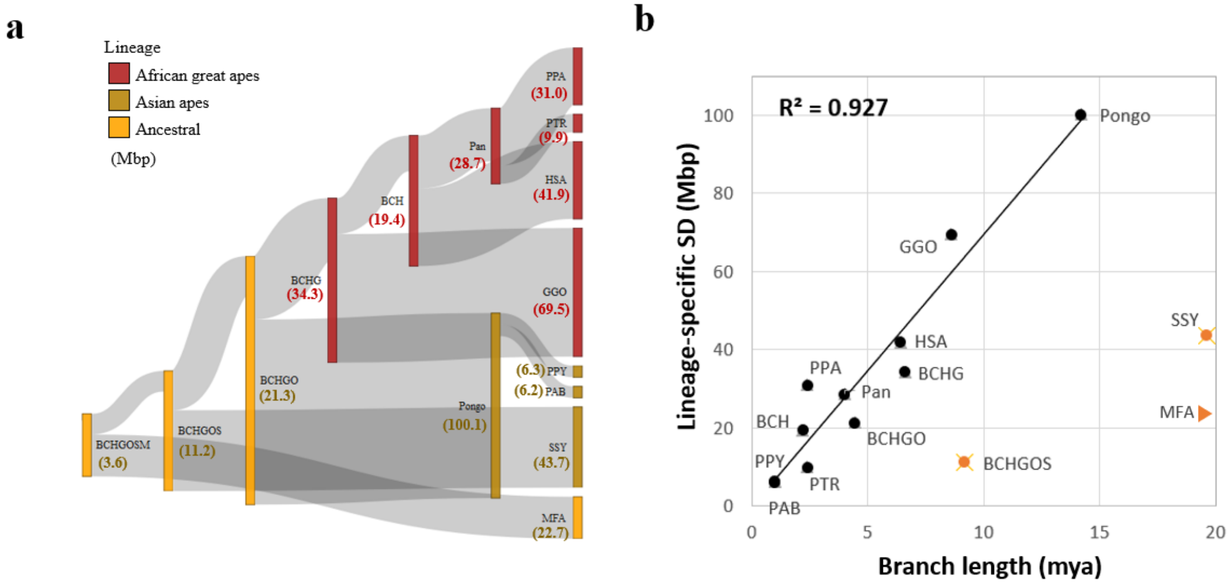

Supplementary Figure XXI.75. Identification of lineage-specific SDs. (a) Assignment of SDs (in Mbp) to ancestral and lineage-specific terminal branches based on content, location, and copy number differences (**Supplementary Fig. XXI.70**). This excludes acrocentric SDs. Asian (dark yellow) and African (red) apes are compared using macaque (MFA) (lighter yellow) as an outgroup. (b) Estimated divergence time (based on SNVs) correlates with SD accumulation (R^2=0.927) with notable outliers including siamang, macaque, and ancestral branches (e.g., BC= bonobo/chimpanzee, HBC= human/bonobo/chimpanzee ancestral node, etc.).

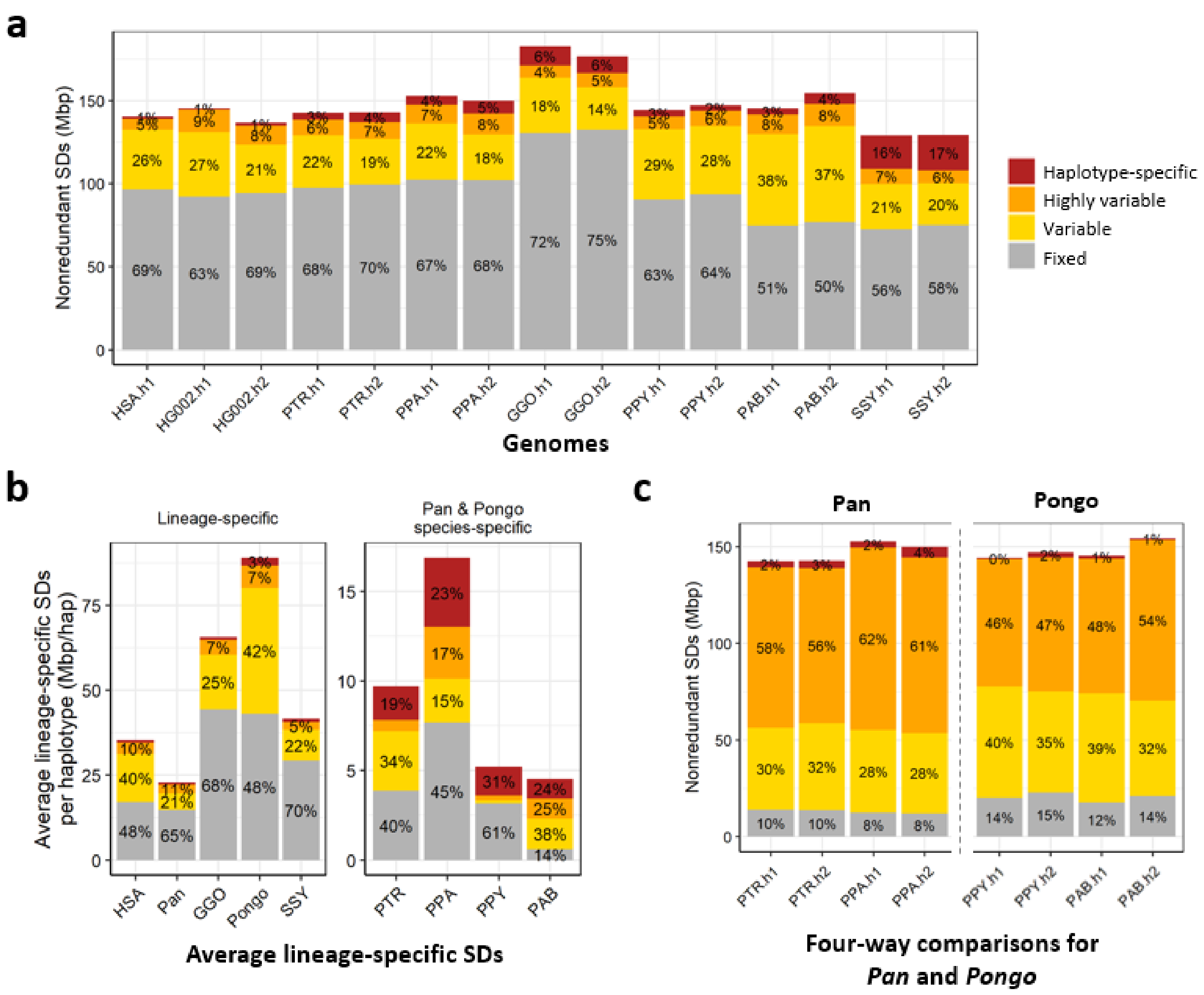

Supplementary Figure XXI.76. Within-species variability of SDs observed between two haplotypes. (a) Classification of "fixed", "haplotype-specific", "highly variable length", and "variable length" SDs with respect to the alternative haplotype. (b) Average inter-haplotype variability within each species observed for the lineage-specific SDs. Within species-specific SDs that correspond under the *Pan* and *Pongo* genera are summarized separately due to relatively short divergence time between the two. (c) Four-way comparison among haplotypes of the *Pan* and *Pongo* lineages to quantify SD variability between haplotypes of less diverged species.

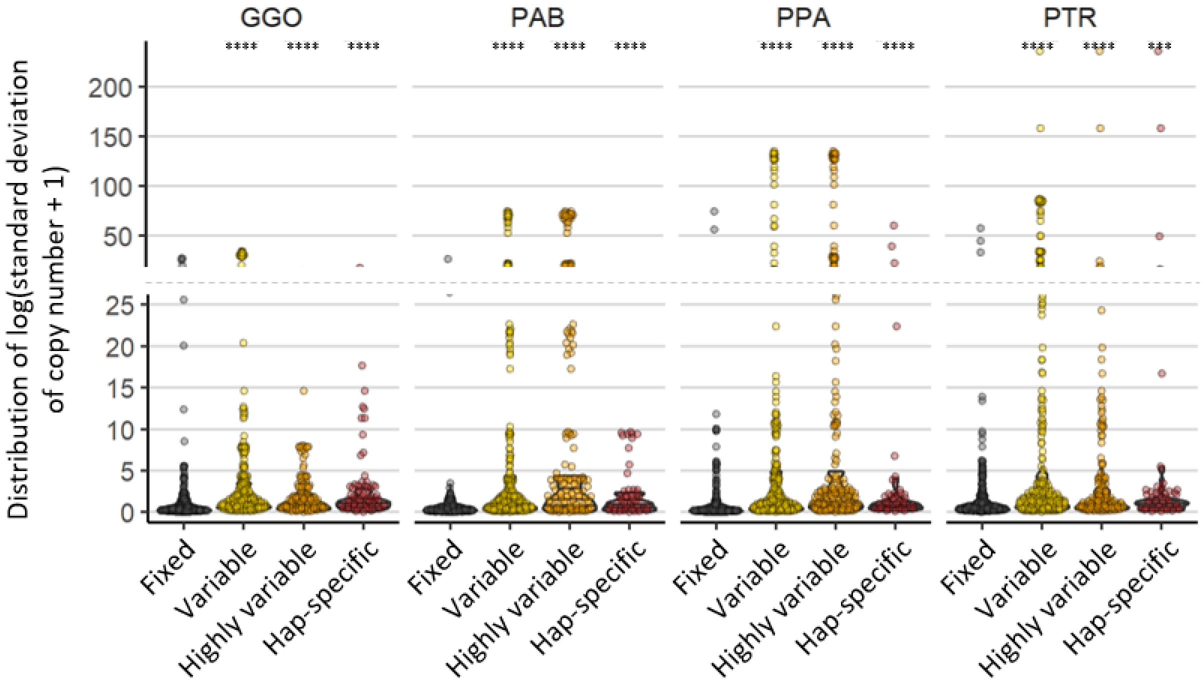

Supplementary Figure XXI.77. Distribution of log(standard deviation of copy number + 1) across 10 additional individuals of gorilla, Sumatran orangutan, bonobo, and chimpanzee for the four categories of variable status. The standard deviation of copy number found for SD genes flagged as fixed between two haplotypes is significantly smaller (two-tailed Wilcox ranked sum test, indicated on the top ***: p<0.001 ****:p<0.0001) than variable SDs.

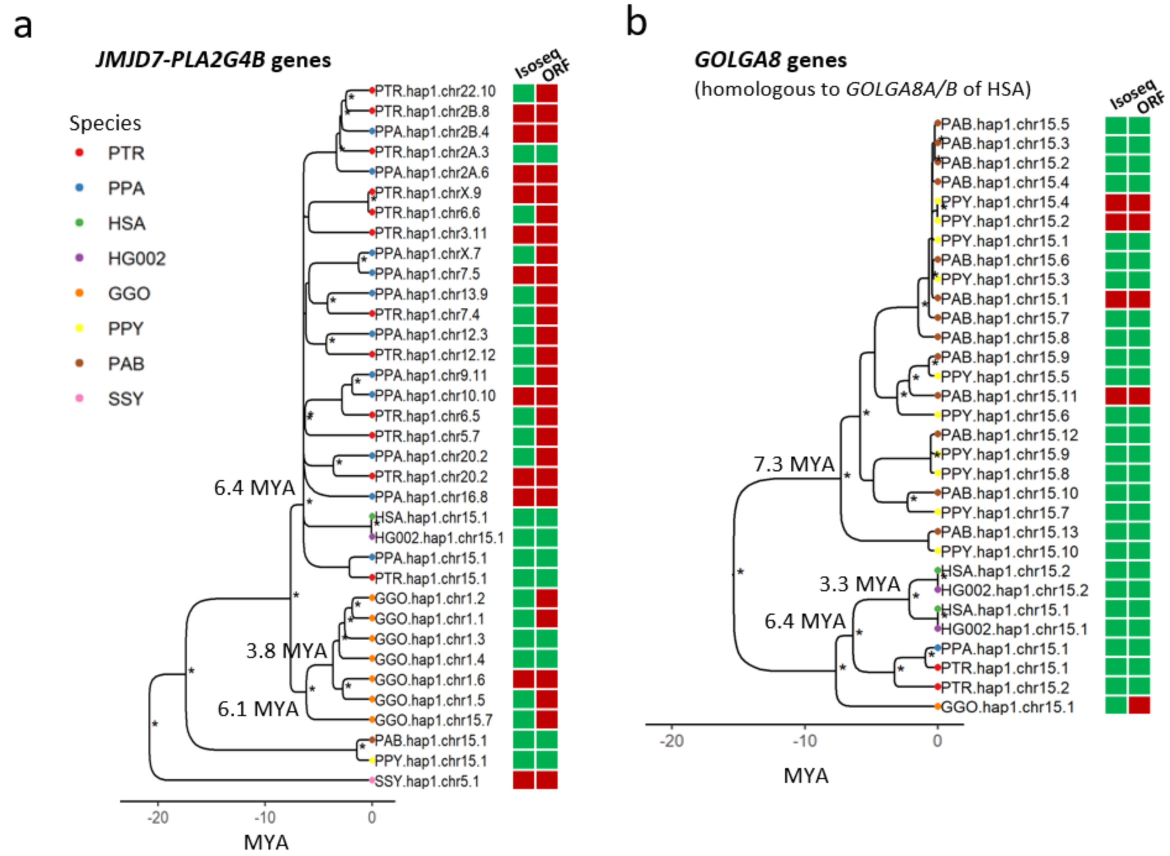

Supplementary Figure XXI.78. Phylogeny of the expanded genes. (a) *JMJD7-PLA2G4B* of GGO chr1 double inversion. (b) *GOLGA8* expansion of *Pongo* chr16q. Iso-Seq support and valid ORF (allowing for at least 70% of full-length peptide sequence) are indicated in green.

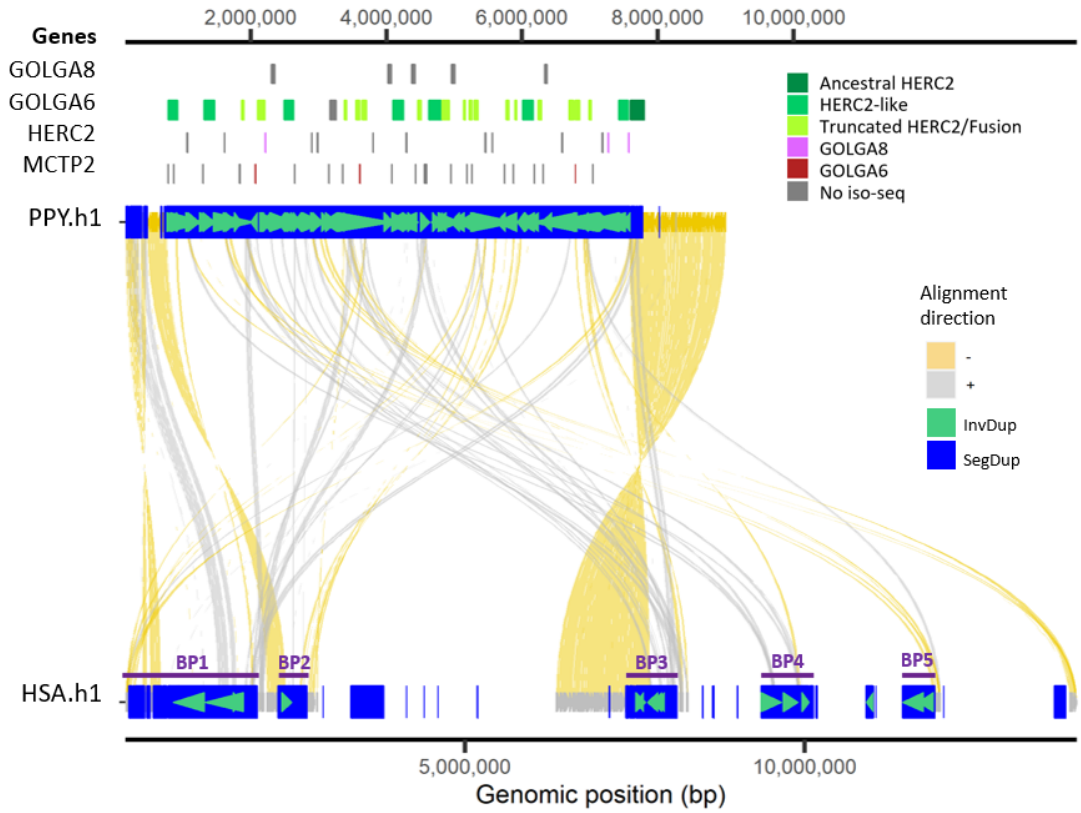

Supplementary Figure XXI.79. Zoomed-in view of the largest SD expansion in *Pongo* chr16.

REFERENCES

1 Makova, K. D. et al. The complete sequence and comparative analysis of ape sex chromosomes. Nature, 1-11 (2024).

2 Prado-Martinez, J. et al. Great ape genetic diversity and population history. Nature 499, 471-475 (2013).

3 De Manuel, M. et al. Chimpanzee genomic diversity reveals ancient admixture with bonobos. Science 354, 477-481 (2016). https://doi.org/10.1126/science.aag2602

4 Pawar, H. et al. Ghost admixture in eastern gorillas. Nat Ecol Evol 7, 1503-1514 (2023). https://doi.org/10.1038/s41559-023-02145-2

5 Xue, Y. et al. Mountain gorilla genomes reveal the impact of long-term population decline and inbreeding. Science 348, 242-245 (2015). https://doi.org/10.1126/science.aaa3952

6 Rautiainen, M. et al. Telomere-to-telomere assembly of diploid chromosomes with Verkko. Nature Biotechnology 41, 1474-1482 (2023).

7 Rhie, A., Walenz, B. P., Koren, S. & Phillippy, A. M. Merqury: reference-free quality, completeness, and phasing assessment for genome assemblies. Genome biology 21, 1-27 (2020).

8 Rautiainen, M. & Marschall, T. GraphAligner: rapid and versatile sequence-to-graph alignment. Genome biology 21, 253 (2020).

9 Mc Cartney, A. M. et al. Chasing perfection: validation and polishing strategies for telomere-to-telomere genome assemblies. Nature methods 19, 687-695 (2022).

10 Rhie, A. et al. The complete sequence of a human Y chromosome. Nature 621, 344-354 (2023).

11 Formenti, G. et al. Merfin: improved variant filtering, assembly evaluation and polishing via k-mer validation. Nature methods 19, 696-704 (2022).

12 Pickrell, J. K. et al. Signals of recent positive selection in a worldwide sample of human populations. Genome research 19, 826-837 (2009).

13 Lejeune, J. et al. A proposed standard system of nomenclature of human mitotic chromosomes. The Lancet 275, 1063-1065 (1960).

14 Levan, A., Fredga, K. & Sandberg, A. A. Nomenclature for centromeric position on chromosomes. (1964).

15 Li, H. & Rong, J. Bedtk: finding interval overlap with implicit interval tree. Bioinformatics 37, 1315-1316 (2021).

16 Siepel, A. et al. Evolutionarily conserved elements in vertebrate, insect, worm, and yeast genomes. Genome research 15, 1034-1050 (2005).

17 Secomandi, S. et al. A chromosome-level reference genome and pangenome for barn swallow population genomics. Cell reports 42 (2023).

18 Hickey, G. et al. Pangenome graph construction from genome alignments with Minigraph-Cactus. Nature biotechnology 42, 663-673 (2024).

19 Liao, W.-W. et al. A draft human pangenome reference. Nature 617, 312-324 (2023).

20 Armstrong, J. et al. Progressive Cactus is a multiple-genome aligner for the thousand-genome era. Nature 587, 246-251 (2020).

21 The 1000 Genomes Project Consortium, G. P. A global reference for human genetic variation. Nature 526, 68 (2015).

22 Herrero, J. et al. Ensembl comparative genomics resources. Database 2016, bav096 (2016).

23 Harris, R. S. Improved pairwise alignment of genomic DNA. (The Pennsylvania State University, 2007).

24 Locke, D. P. et al. Comparative and demographic analysis of orang-utan genomes. Nature 469, 529-533 (2011).

25 Mattle-Greminger, M. P. et al. Genomes reveal marked differences in the adaptive evolution between orangutan species. Genome biology 19, 1-13 (2018).

26 Shao, Y. et al. Phylogenomic analyses provide insights into primate evolution. Science 380, 913-924 (2023).

27 Schiffels, S. & Wang, K. MSMC and MSMC2: the multiple sequentially markovian coalescent. Statistical population genomics. Humana. 147-165 (2020).

28 Wang, K., Mathieson, I., O'Connell, J. & Schiffels, S. Tracking human population structure through time from whole genome sequences. PLoS genetics 16, e1008552 (2020).

29 Rivas-González, I. et al. Pervasive incomplete lineage sorting illuminates speciation and selection in primates. Science 380, eabn4409 (2023).

30 Mao, Y. et al. A high-quality bonobo genome refines the analysis of hominid evolution. Nature 594, 77-81 (2021).

31 Carbone, L. et al. Gibbon genome and the fast karyotype evolution of small apes. Nature 513, 195-201 (2014).

32 Poplin, R. et al. Scaling accurate genetic variant discovery to tens of thousands of samples. bioRxiv. 2018: 201178. DOI 10, 201178 (2018).

33 Browning, B. L. & Browning, S. R. Genotype imputation with millions of reference samples. The American Journal of Human Genetics 98, 116-126 (2016).

34 DeGiorgio, M., Huber, C. D., Hubisz, M. J., Hellmann, I. & Nielsen, R. SweepFinder2: increased sensitivity, robustness and flexibility. Bioinformatics 32, 1895-1897 (2016).

35 DeGiorgio, M. & Szpiech, Z. A. A spatially aware likelihood test to detect sweeps from haplotype distributions. PLoS genetics 18, e1010134 (2022).

36 Souilmi, Y. et al. Admixture has obscured signals of historical hard sweeps in humans. Nature Ecology & Evolution 6, 2003-2015 (2022).

37 Storey, J. D. The positive false discovery rate: a Bayesian interpretation and the q-value. The annals of statistics 31, 2013-2035 (2003).

38 Kofler, R. & Schlötterer, C. Gowinda: unbiased analysis of gene set enrichment for genome-wide association studies. Bioinformatics 28, 2084-2085 (2012).

39 Berriz, G. F., Beaver, J. E., Cenik, C., Tasan, M. & Roth, F. P. Next generation software for functional trend analysis. Bioinformatics 25, 3043-3044 (2009).

40 Hedrick, P. W. Balancing selection and MHC. Genetica 104, 207-214 (1998).

41 De Groot, N. G. et al. Evidence for an ancient selective sweep in the MHC class I gene repertoire of chimpanzees. Proceedings of the National Academy of Sciences 99, 11748-11753 (2002).

42 De Groot, N. G. et al. Pinpointing a selective sweep to the chimpanzee MHC class I region by comparative genomics. Molecular ecology 17, 2074-2088 (2008).

43 de Groot, N. G., Heijmans, C. M. & Bontrop, R. E. AIDS in chimpanzees: the role of MHC genes. Immunogenetics 69, 499-509 (2017).

44 Hayakawa, T. et al. Eco-geographical diversification of bitter taste receptor genes (TAS2Rs) among subspecies of chimpanzees (Pan troglodytes). PLOS ONE e43277 (2012).

45 Parry, C. M., Erkner, A. & le Coutre, J. Divergence of T2R chemosensory receptor families in humans, bonobos, and chimpanzees. Proceedings of the National Academy of Sciences 101, 14830-14834 (2004).

46 McManus, K. F. et al. Inference of gorilla demographic and selective history from whole-genome sequence data. Molecular biology and evolution 32, 600-612 (2015).

47 Cagan, A. et al. Natural selection in the great apes. Molecular biology and evolution 33, 3268-3283 (2016).

48 Nye, J., Mondal, M., Bertranpetit, J. & Laayouni, H. A fully integrated machine learning scan of selection in the chimpanzee genome. NAR Genomics and Bioinformatics 2, lqaa061 (2020).

49 Fortier, A. L. & Pritchard, J. K. Ancient Trans-Species Polymorphism at the Major Histocompatibility Complex in Primates. bioRxiv, 2022.2006. 2028.497781 (2022).

50 Abwe, E. E. et al. Dietary ecology of the Nigeria–Cameroon Chimpanzee (Pan troglodytes ellioti). International Journal of Primatology 41, 81-104 (2020).

51 Carvalho, J. S., Vicente, L. & Marques, T. A. Chimpanzee (Pan troglodytes verus) diet composition and food availability in a human-modified landscape at Lagoas de Cufada Natural Park, Guinea-Bissau. International Journal of Primatology 36, 802-822 (2015).

52 Watts, D. P., Potts, K. B., Lwanga, J. S. & Mitani, J. C. Diet of chimpanzees (Pan troglodytes schweinfurthii) at Ngogo, Kibale National Park, Uganda, 1. Diet composition and diversity. American Journal of Primatology 74, 114-129 (2012).

53 Reynolds, V. et al. Mineral acquisition from clay by Budongo forest chimpanzees. PLoS One 10, e0134075 (2015).

54 Pebsworth, P. A. et al. Geophagy among East African Chimpanzees: consumed soils provide protection from plant secondary compounds and bioavailable iron. Environmental geochemistry and health 41, 2911-2927 (2019).

55 Reynolds, V., Pascual-Garrido, A., Lloyd, A. W., Lyons, P. & Hobaiter, C. Possible mineral contributions to the diet and health of wild chimpanzees in three East African forests. American Journal of Primatology 81, e22978 (2019).

56 Mahaney, W. C. et al. Soils consumed by chimpanzees of the Kanyawara community in the Kibale Forest, Uganda. International Journal of Primatology 26, 1375-1398 (2005).

57 Pebsworth, P. A., Huffman, M. A., Lambert, J. E. & Young, S. L. Geophagy among nonhuman primates: A systematic review of current knowledge and suggestions for future directions. American Journal of Physical Anthropology 168, 164-194 (2019).

58 Panichev, A. M., et al. Rare earth elements as a causal factor of geophagy among herbivorous animals. Doklady Earth Sciences. Vol. 499 pp. 599-603 (2021)

59 Wang, M.-X. & Peng, Z.-G. 17β-hydroxysteroid dehydrogenases in the progression of nonalcoholic fatty liver disease. Pharmacology & Therapeutics 246, 108428 (2023).

60 Ding, J. et al. HADHA alleviates hepatic steatosis and oxidative stress in NAFLD via inactivation of the MKK3/MAPK pathway. Molecular Biology Reports 50, 961-970 (2023).

61 Dourmashkin, J. et al. Different forms of obesity as a function of diet composition. International journal of obesity 29, 1368-1378 (2005).

62 Ding, Y. et al. The vitamin K epoxide reductase Vkorc1l1 promotes preadipocyte differentiation in mice. Obesity 26, 1303-1311 (2018).

63 Pawar, H., Ostridge, H. J., Schmidt, J. M. & Andrés, A. M. Genetic adaptations to SIV across chimpanzee populations. PLoS Genetics 18, e1010337 (2022).

64 Schmidt, J. M., de Manuel, M., Marques-Bonet, T., Castellano, S. & Andrés, A. M. The impact of genetic adaptation on chimpanzee subspecies differentiation. PLoS Genetics 15, e1008485 (2019).

65 Kelly, A. & Trowsdale, J. Genetics of antigen processing and presentation. Immunogenetics 71, 161-170 (2019).

66 Ressing, M. E., Luteijn, R. D., Horst, D. & Wiertz, E. J. Viral interference with antigen presentation: trapping TAP. Molecular immunology 55, 139-142 (2013).

67 Enard, D., Cai, L., Gwennap, C. & Petrov, D. A. Viruses are a dominant driver of protein adaptation in mammals. elife 5, e12469 (2016).

68 Shumate, A. & Salzberg, S. L. Liftoff: accurate mapping of gene annotations. Bioinformatics 37, 1639-1643 (2021).

69 Li, H. Protein-to-genome alignment with miniprot. Bioinformatics 39, btad014 (2023).

70 Li, H. Minimap2: pairwise alignment for nucleotide sequences. Bioinformatics 34, 3094-3100 (2018).

71 Pandey, P., Pradhan, S. & Mittal, B. LRP-associated protein gene (LRPAP1) and susceptibility to degenerative dementia. Genes, Brain and Behavior 7, 943-950 (2008).

72 Singh, N. K. et al. APOE and LRPAP1 gene polymorphism and risk of Parkinson's disease. Neurological Sciences 35, 1075-1081 (2014).

73 Shumate, A., Wong, B., Pertea, G. & Pertea, M. Improved transcriptome assembly using a hybrid of long and short reads with StringTie. PLoS computational biology 18, e1009730 (2022).

74 Pertea, G. & Pertea, M. GFF utilities: GffRead and GffCompare. F1000Research 9 (2020).

75 Bayardo, R. J., Ma, Y. & Srikant, R. Scaling up all pairs similarity search. Proceedings of the 16th international conference on World Wide Web (2007).

76 Anand, L. & Rodriguez Lopez, C. M. ChromoMap: an R package for interactive visualization of multi-omics data and annotation of chromosomes. BMC bioinformatics 23, 33 (2022).

77 Hoyt, S. J. et al. From telomere to telomere: The transcriptional and epigenetic state of human repeat elements. Science 376, eabk3112 (2022).

78 Bao, W., Kojima, K. K. & Kohany, O. Repbase Update, a database of repetitive elements in eukaryotic genomes. Mobile Dna 6, 1-6 (2015).

79 Tempel, S. Using and understanding RepeatMasker. Mobile genetic elements: protocols and genomic applications, 29-51 (2012).

80 Cechova, M. et al. High satellite repeat turnover in great apes studied with short-and long-read technologies. Molecular biology and evolution 36, 2415-2431 (2019).

81 Benson, G. Tandem repeats finder: a program to analyze DNA sequences. Nucleic acids research 27, 573-580 (1999).

82 Olson, D. & Wheeler, T. in Proceedings of the 2018 ACM International Conference on Bioinformatics, Computational Biology, and Health Informatics. 37-46 (2018).

83 Quinlan, A. R. & Hall, I. M. BEDTools: a flexible suite of utilities for comparing genomic features. Bioinformatics 26, 841-842 (2010).

84 Storer, J., Hubley, R., Rosen, J., Wheeler, T. J. & Smit, A. F. The Dfam community resource of transposable element families, sequence models, and genome annotations. Mobile DNA 12, 1-14 (2021).

85 Kent, W. J. BLAT—the BLAST-like alignment tool. Genome research 12, 656-664 (2002).

86 Bray, J. R. & Curtis, J. T. An ordination of the upland forest communities of southern Wisconsin. Ecological monographs 27, 326-349 (1957).

87 Sperber, G. O., Airola, T., Jern, P. & Blomberg, J. Automated recognition of retroviral sequences in genomic data—RetroTector©. Nucleic acids research 35, 4964-4976 (2007).

88 Kojima, K. K. Human transposable elements in Repbase: genomic footprints from fish to humans. Mobile DNA 9, 2 (2018).

89 Clawson, H. et al. GenArk: towards a million UCSC genome browsers. Genome Biology 24, 217 (2023).

90 Johnson, W. E. Origins and evolutionary consequences of ancient endogenous retroviruses. Nature Reviews Microbiology 17, 355-370 (2019).

91 Henriques, W. S. et al. The diverse evolutionary histories of domesticated metaviral capsid genes in mammals. Molecular Biology and Evolution 41, msae061 (2024).

92 Finn, R. D., Clements, J. & Eddy, S. R. HMMER web server: interactive sequence similarity searching. Nucleic acids research 39, W29-W37 (2011).

93 Jones, P. et al. InterProScan 5: genome-scale protein function classification. Bioinformatics 30, 1236-1240 (2014).

94 Ebert, P. et al. Haplotype-resolved diverse human genomes and integrated analysis of structural variation. Science 372, eabf7117 (2021).

95 Chen, Y., Ye, W., Zhang, Y. & Xu, Y. High speed BLASTN: an accelerated MegaBLAST search tool. Nucleic acids research 43, 7762-7768 (2015).

96 Tao, Y., He, C., Lin, D., Gu, Z. & Pu, W. Comprehensive Identification of Mitochondrial Pseudogenes (NUMTs) in the Human Telomere-to-Telomere Reference Genome. Genes 14, 2092 (2023).

97 Schneider, V. A. et al. Evaluation of GRCh38 and de novo haploid genome assemblies demonstrates the enduring quality of the reference assembly. Genome research 27, 849-864 (2017).

98 Kronenberg, Z. N. et al. High-resolution comparative analysis of great ape genomes. Science 360, eaar6343 (2018).

99 Cechova, M. et al. Dynamic evolution of great ape Y chromosomes. Proceedings of the National Academy of Sciences 117, 26273-26280 (2020).

100 Sirupurapu, V., Safonova, Y. & Pevzner, P. A. Gene prediction in the immunoglobulin loci. Genome research 32, 1152-1169 (2022).

101 Lees, W. D., Saha, S., Yaari, G. & Watson, C. T. Digger: directed annotation of immunoglobulin and T cell receptor V, D, and J gene sequences and assemblies. Bioinformatics 40, btae144 (2024).

102 Li, H. et al. The sequence alignment/map format and SAMtools. bioinformatics 25, 2078-2079 (2009).

103 Jiang, Z. et al. Ancestral reconstruction of segmental duplications reveals punctuated cores of human genome evolution. Nature genetics 39, 1361-1368 (2007).

104 Minkin, I. & Medvedev, P. Scalable multiple whole-genome alignment and locally collinear block construction with SibeliaZ. Nature communications 11, 6327 (2020).

105 Sievers, F. & Higgins, D. G. Clustal Omega for making accurate alignments of many protein sequences. Protein Science 27, 135-145 (2018).

106 Moore, R. M., Harrison, A. O., McAllister, S. M., Polson, S. W. & Wommack, K. E. Iroki: automatic customization and visualization of phylogenetic trees. PeerJ 8, e8584 (2020).

107 Noé, L. & Kucherov, G. YASS: enhancing the sensitivity of DNA similarity search. Nucleic acids research 33, W540-W543 (2005).

108 Lefranc, M.-P. et al. IMGT®, the international ImMunoGeneTics information system®. Nucleic acids research 37, D1006-D1012 (2009).

109 Lees, W. et al. OGRDB: a reference database of inferred immune receptor genes. Nucleic acids research 48, D964-D970 (2020).

110 Rodriguez, O. L. et al. Genetic variation in the immunoglobulin heavy chain locus shapes the human antibody repertoire. Nature communications 14, 4419 (2023).

111 Bankevich, A., Bzikadze, A. V., Kolmogorov, M., Antipov, D. & Pevzner, P. A. Multiplex de Bruijn graphs enable genome assembly from long, high-fidelity reads. Nature biotechnology 40, 1075-1081 (2022).

112 Rodriguez, O. L., Silver, C. A., Shields, K., Smith, M. L. & Watson, C. T. Targeted long-read sequencing facilitates phased diploid assembly and genotyping of the human T cell receptor alpha, delta, and beta loci. Cell Genomics 2 (2022).

113 Shiina, T., Blancher, A., Inoko, H. & Kulski, J. K. Comparative genomics of the human, macaque and mouse major histocompatibility complex. Immunology 150, 127-138 (2017).

114 Danecek, P. et al. Twelve years of SAMtools and BCFtools. Gigascience 10, giab008 (2021).

115 Mao, Y. et al. Structurally divergent and recurrently mutated regions of primate genomes. Cell 187, 1547-1562. e1513 (2024).

116 Slater, G. S. C. & Birney, E. Automated generation of heuristics for biological sequence comparison. BMC bioinformatics 6, 1-11 (2005).

117 Larsson, A. AliView: a fast and lightweight alignment viewer and editor for large datasets. Bioinformatics 30, 3276-3278 (2014).

118 Katoh, K. & Standley, D. M. MAFFT multiple sequence alignment software version 7: improvements in performance and usability. Mol Biol Evol 30, 772-780 (2013). https://doi.org/10.1093/molbev/mst010

119 Trifinopoulos, J., Nguyen, L.-T., von Haeseler, A. & Minh, B. Q. W-IQ-TREE: a fast online phylogenetic tool for maximum likelihood analysis. Nucleic acids research 44, W232-W235 (2016).

120 Hoang, D. T., Chernomor, O., Von Haeseler, A., Minh, B. Q. & Vinh, L. S. UFBoot2: improving the ultrafast bootstrap approximation. Molecular biology and evolution 35, 518-522 (2018).

121 Yu, G., Smith, D. K., Zhu, H., Guan, Y. & Lam, T. T. Y. ggtree: an R package for visualization and annotation of phylogenetic trees with their covariates and other associated data. Methods in Ecology and Evolution 8, 28-36 (2017).

122 Paradis, E. & Schliep, K. ape 5.0: an environment for modern phylogenetics and evolutionary analyses in R. Bioinformatics 35, 526-528 (2019).

123 Marçais, G. et al. MUMmer4: A fast and versatile genome alignment system. PLoS computational biology 14, e1005944 (2018).

124 Porubsky, D. et al. SVbyEye: A visual tool to characterize structural variation among whole-genome assemblies. bioRxiv, 2024.2009. 2011.612418 (2024).

125 Villanueva, R. A. M. & Chen, Z. J. ggplot2: elegant graphics for data analysis. Taylor & Francis 160-167 (2019).

126 Minh, B. Q. et al. IQ-TREE 2: New Models and Efficient Methods for Phylogenetic Inference in the Genomic Era. Mol Biol Evol 37, 1530-1534 (2020). https://doi.org/10.1093/molbev/msaa015

127 Huerta-Cepas, J., Serra, F. & Bork, P. ETE 3: reconstruction, analysis, and visualization of phylogenomic data. Molecular biology and evolution 33, 1635-1638 (2016).

128 Heijmans, C. M., de Groot, N. G. & Bontrop, R. E. Comparative genetics of the major histocompatibility complex in humans and nonhuman primates. International Journal of Immunogenetics 47, 243-260 (2020).

129 Radwan, J., Babik, W., Kaufman, J., Lenz, T. L. & Winternitz, J. Advances in the evolutionary understanding of MHC polymorphism. Trends in Genetics 36, 298-311 (2020).

130 Lenz, T. L., Spirin, V., Jordan, D. M. & Sunyaev, S. R. Excess of deleterious mutations around HLA genes reveals evolutionary cost of balancing selection. Molecular Biology and Evolution 33, 2555-2564 (2016).

131 Goel, M., Sun, H., Jiao, W.-B. & Schneeberger, K. SyRI: finding genomic rearrangements and local sequence differences from whole-genome assemblies. Genome biology 20, 1-13 (2019).

132 Porubsky, D. et al. Recurrent inversion toggling and great ape genome evolution. Nature genetics 52, 849-858 (2020).

133 Yunis, J. J. & Prakash, O. The origin of man: a chromosomal pictorial legacy. Science 215, 1525-1530 (1982).

134 Müller, S., Finelli, P., Neusser, M. & Wienberg, J. The evolutionary history of human chromosome 7. Genomics 84, 458-467 (2004).

135 Kehrer-Sawatzki, H., Szamalek, J. M., Tänzer, S., Platzer, M. & Hameister, H. Molecular characterization of the pericentric inversion of chimpanzee chromosome 11 homologous to human chromosome 9. Genomics 85, 542-550 (2005).

136 Carbone, L., Ventura, M., Tempesta, S., Rocchi, M. & Archidiacono, N. Evolutionary history of chromosome 10 in primates. Chromosoma 111, 267-272 (2002).

137 Cardone, M. F. et al. Evolutionary history of chromosome 11 featuring four distinct centromere repositioning events in Catarrhini. Genomics 90, 35-43 (2007).

138 Kehrer-Sawatzki, H., Sandig, C., Goidts, V. & Hameister, H. Breakpoint analysis of the pericentric inversion between chimpanzee chromosome 10 and the homologous chromosome 12 in humans. Cytogenetic and Genome Research 108, 91-97 (2004).

139 Kehrer-Sawatzki, H. et al. Molecular characterization of the pericentric inversion that causes differences between chimpanzee chromosome 19 and human chromosome 17. The American Journal of Human Genetics 71, 375-388 (2002).

140 Cardone, M. F. et al. Hominoid chromosomal rearrangements on 17q map to complex regions of segmental duplication. Genome biology 9, 1-11 (2008).

141 Goidts, V., Szamalek, J. M., Hameister, H. & Kehrer-Sawatzki, H. Segmental duplication associated with the human-specific inversion of chromosome 18: a further example of the impact of segmental duplications on karyotype and genome evolution in primates. Human genetics 115, 116-122 (2004).

142 Misceo, D. et al. Evolutionary history of chromosome 20. Molecular Biology and Evolution 22, 360-366 (2005).

143 Ventura, M. et al. Gorilla genome structural variation reveals evolutionary parallelisms with chimpanzee. Genome research 21, 1640-1649 (2011).

144 Capozzi, O. et al. A comprehensive molecular cytogenetic analysis of chromosome rearrangements in gibbons. Genome Research 22, 2520-2528 (2012).

145 Catacchio, C. R. et al. Inversion variants in human and primate genomes. Genome Research 28, 910-920 (2018).

146 Maggiolini, F. A. M. et al. Single-cell strand sequencing of a macaque genome reveals multiple nested inversions and breakpoint reuse during primate evolution. Genome research 30, 1680-1693 (2020).

147 Mercuri, L. et al. A high-resolution map of small-scale inversions in the gibbon genome. Genome Research 32, 1941-1951 (2022).

148 Nuttle, X. et al. Emergence of a Homo sapiens-specific gene family and chromosome 16p11. 2 CNV susceptibility. Nature 536, 205-209 (2016).

149 Paparella, A. et al. Structural Variation Evolution at the 15q11-q13 Disease-Associated Locus. International Journal of Molecular Sciences 24, 15818 (2023).

150 Zody, M. C. et al. Evolutionary toggling of the MAPT 17q21. 31 inversion region. Nature genetics 40, 1076-1083 (2008).

151 Maggiolini, F. A. et al. Genomic inversions and GOLGA core duplicons underlie disease instability at the 15q25 locus. PLoS Genetics 15, e1008075 (2019).

152 Antonacci, F. et al. Characterization of six human disease-associated inversion polymorphisms. Human molecular genetics 18, 2555-2566 (2009).

153 Kent, W. J., Baertsch, R., Hinrichs, A., Miller, W. & Haussler, D. Evolution's cauldron: duplication, deletion, and rearrangement in the mouse and human genomes. Proceedings of the National Academy of Sciences 100, 11484-11489 (2003).

154 Mangan, R. J. et al. Adaptive sequence divergence forged new neurodevelopmental enhancers in humans. Cell 185, 4587-4603. e4523 (2022).

155 Blanchette, M. et al. Aligning multiple genomic sequences with the threaded blockset aligner. Genome research 14, 708-715 (2004).

156 Yang, Z., Kumar, S. & Nei, M. A new method of inference of ancestral nucleotide and amino acid sequences. Genetics 141, 1641-1650 (1995).

157 Kundaje, A. et al. Integrative analysis of 111 reference human epigenomes. Nature 518, 317-330 (2015).

158 Au, E. H. et al. Gonomics: uniting high performance and readability for genomics with Go. Bioinformatics 39, btad516 (2023).

159 Whalen, S. et al. Machine learning dissection of human accelerated regions in primate neurodevelopment. Neuron 111, 857-873. e858 (2023).

160 Boix, C. A., James, B. T., Park, Y. P., Meuleman, W. & Kellis, M. Regulatory genomic circuitry of human disease loci by integrative epigenomics. Nature 590, 300-307 (2021).

161 Bernstein, B. E. et al. A bivalent chromatin structure marks key developmental genes in embryonic stem cells. Cell 125, 315-326 (2006).

162 Xie, K. T. et al. DNA fragility in the parallel evolution of pelvic reduction in stickleback fish. Science 363, 81-84 (2019).

163 Nesta, A. V., Tafur, D. & Beck, C. R. Hotspots of human mutation. Trends in Genetics 37, 717-729 (2021).

164 Vollger, M. R. et al. Increased mutation and gene conversion within human segmental duplications. Nature 617, 325-334 (2023).

165 Kirilenko, B. M. et al. Integrating gene annotation with orthology inference at scale. Science 380, eabn3107 (2023).

166 Kirilenko, B. M. et al. Integrating gene annotation with orthology inference at scale. Science 380, eabn3107 (2023).

167 Willcox, B. J. et al. FOXO3A genotype is strongly associated with human longevity. Proceedings of the National Academy of Sciences 105, 13987-13992 (2008).

168 Flachsbart, F. et al. Association of FOXO3A variation with human longevity confirmed in German centenarians. Proceedings of the National Academy of Sciences 106, 2700-2705 (2009).

169 Donlon, T. A. et al. FOXO 3 longevity interactome on chromosome 6. Aging Cell 16, 1016-1025 (2017).

170 Frankum, R. et al. Extreme longevity variants at the FOXO3 locus may moderate FOXO3 isoform levels. GeroScience 44, 1129-1140 (2022).

171 Santo, E. E. & Paik, J. A splice junction-targeted CRISPR approach (spJCRISPR) reveals human FOXO3B to be a protein-coding gene. Gene 673, 95-101 (2018).

172 Park, S.-S. et al. Structure and evolution of the Smith-Magenis syndrome repeat gene clusters, SMS-REPs. Genome research 12, 729-738 (2002).

173 Poliseno, L. et al. A coding-independent function of gene and pseudogene mRNAs regulates tumour biology. Nature 465, 1033-1038 (2010).

174 Sahakyan, A. B. et al. Machine learning model for sequence-driven DNA G-quadruplex formation. Scientific reports 7, 14535 (2017).

175 Jain, C. et al. Weighted minimizer sampling improves long read mapping. Bioinformatics 36, i111-i118 (2020).

176 Pedregosa, F. et al. Scikit-learn: Machine learning in Python. the Journal of machine Learning research 12, 2825-2830 (2011).

177 Keller, T. E., Han, P. & Yi, S. V. Evolutionary transition of promoter and gene body DNA methylation across invertebrate–vertebrate boundary. Molecular biology and evolution 33, 1019-1028 (2016).

178 Yang, Y. et al. Continuous-trait probabilistic model for comparing multi-species functional genomic data. Cell systems 7, 208-218. e211 (2018).

179 Marchal, C. et al. Genome-wide analysis of replication timing by next-generation sequencing with E/L Repli-seq. Nature protocols 13, 819-839 (2018).

180 Li, H. & Durbin, R. Fast and accurate short read alignment with Burrows–Wheeler transform. bioinformatics 25, 1754-1760 (2009).

181 Hinrichs, A. S. et al. The UCSC genome browser database: update 2006. Nucleic acids research 34, D590-D598 (2006).

182 Baid, G. et al. An extensive sequence dataset of gold-standard samples for benchmarking and development. bioRxiv, 2020.2012. 2011.422022 (2020).

183 van Sluis, M. et al. Human NORs, comprising rDNA arrays and functionally conserved distal elements, are located within dynamic chromosomal regions. Genes & Development 33, 1688-1701 (2019).

184 Nurk, S. et al. The complete sequence of a human genome. Science 376, 44-53 (2022).

185 R Core Team, R. (R foundation for statistical computing Vienna, Austria, 2013).

186 Thorvaldsdóttir, H., Robinson, J. T. & Mesirov, J. P. Integrative Genomics Viewer (IGV): high-performance genomics data visualization and exploration. Briefings in bioinformatics 14, 178-192 (2013).

187 Vollger, M. R. et al. Long-read sequence and assembly of segmental duplications. Nature methods 16, 88-94 (2019).

188 Vollger, M. R., Kerpedjiev, P., Phillippy, A. M. & Eichler, E. E. StainedGlass: interactive visualization of massive tandem repeat structures with identity heatmaps. Bioinformatics 38, 2049-2051 (2022).

189 Gershman, A. et al. Epigenetic patterns in a complete human genome. Science 376, eabj5089 (2022).

190 Numanagić, I. et al. Fast characterization of segmental duplications in genome assemblies. Bioinformatics 34, i706-i714 (2018).

191 Morgulis, A., Gertz, E. M., Schäffer, A. A. & Agarwala, R. WindowMasker: window-based masker for sequenced genomes. Bioinformatics 22, 134-141 (2006).
