# Supplementary material for: Complete sequencing of ape genomes
Source: Nature. 2025 Apr 9;641(8062):401–18. doi: 10.1038/s41586-025-08816-3 (PMC12058530; doi:10.1038/s41586-025-08816-3)
Supplement: Supplementary file 2 — Reporting Summary [file 41586_2025_8816_MOESM2_ESM.pdf]

nature portfolio

Corresponding author(s): Evan E. Eichler, Adam M. Phillippy, Kateryna D. Makova

Last updated by author(s): Jan 30, 2025

Reporting Summary

Nature Portfolio wishes to improve the reproducibility of the work that we publish. This form provides structure for consistency and transparency in reporting. For further information on Nature Portfolio policies, see our Editorial Policies and the Editorial Policy Checklist.

Statistics

For all statistical analyses, confirm that the following items are present in the figure legend, table legend, main text, or Methods section.

n/a	Confirmed	
☐	☒	The exact sample size (*n*) for each experimental group/condition, given as a discrete number and unit of measurement
☐	☒	A statement on whether measurements were taken from distinct samples or whether the same sample was measured repeatedly
☐	☒	The statistical test(s) used AND whether they are one- or two-sided<br>*Only common tests should be described solely by name; describe more complex techniques in the Methods section.*
☒	☐	A description of all covariates tested
☐	☒	A description of any assumptions or corrections, such as tests of normality and adjustment for multiple comparisons
☐	☒	A full description of the statistical parameters including central tendency (e.g. means) or other basic estimates (e.g. regression coefficient) AND variation (e.g. standard deviation) or associated estimates of uncertainty (e.g. confidence intervals)
☐	☒	For null hypothesis testing, the test statistic (e.g. *F*, *t*, *r*) with confidence intervals, effect sizes, degrees of freedom and *P* value noted<br>*Give P values as exact values whenever suitable.*
☐	☒	For Bayesian analysis, information on the choice of priors and Markov chain Monte Carlo settings
☒	☐	For hierarchical and complex designs, identification of the appropriate level for tests and full reporting of outcomes
☒	☐	Estimates of effect sizes (e.g. Cohen's *d*, Pearson's *r*), indicating how they were calculated

Our web collection on statistics for biologists contains articles on many of the points above.

Software and code

Policy information about availability of computer code

Data collection

The software used to collect sequencing data include Pacific Biosciences Sequel IIe instrument, using version 2.0 sequencing reagents (control software version 10.1.0.119549), SMRT Link v12.

Data analysis

The custom scripts used in this study are available by the following:

Overall scripts: (https://github.com/marbl/Primates), polishing of the genome (https://github.com/arangrhie/T2T-Polish), assembly QC (https://github.com/EichlerLab/assembly_eval), implicit graph & pangenome (https://github.com/T2T-apes/ape_pangenome), Cactus alignment (https://cglgenomics.ucsc.edu/february-2024-t2t-apes), assessment of ancestral sequence (https://github.com/shanksc/ancestral_state), AQER analysis (https://github.com/vertgenlab/vglDocumentation/tree/master/primateT2T), population genome processing and selection analysis (https://github.com/aabiddanda/haplotype-phasing), acrocentric/rDNA analyses (https://github.com/jouyun/2024_Primate_rDNA, https://github.com/borcherm/primate_rdna_cn), species-specific MEI analysis (https://github.com/Markloftus/t2t-ape-MEIs), non-B DNA annotation and NUMT detection, (https://github.com/makovalab-psu/T2T_primate_autosomes), and transcript comparison (https://github.com/canzarlab/apes_transcriptome_analysis).

In addition to the custom scripts, the following codes were used: Alignment (https://github.com/arangrhie/T2T-Polish/tree/master/winnowmap (v2.03/v1.0), lastz (v1.04), minimap2(v2.24/v2.26/v2.28), blastn (v2.12.0), blastp (v2.12.0), https://github.com/waveygang/wfmash, https://github.com/marbl/MashMap v3.1.1, NUCMER), alignment processing (https://github.com/AndreaGuarracino/paf2chain, http://github.com/wjwei-handsome/wgatools, https://github.com/mrvollger/rustybam v0.1.29, https://github.com/sstadick/perbase), conservation score calculation (PhastCons v1.5), pan-genome graph (https://github.com/pangenome/impg), further assembly QC (https://github.com/mobinasri/flagger v0.3.3), non-B DNA annotation (https://github.com/abcsFrederick/non-B_gfa), gene annotation (https://github.com/ComparativeGenomicsToolkit/Comparative-Annotation-Toolkit, IgDetective, Digger, Exonerate v2.4), repeat annotation

(Repeatmasker v4.1.0/v4.1.5/v4.1.6, TRF v4.1.0, ULTRA, windowmasker v2.2.22, http://doua.prabi.fr/software/one-code-to-find-them-all), transcriptome data alignment (StringTie2 v2.2.1), phylogenetic tree (IQ-TREE v2.1.2) ILS (https://github.com/rivasiker/trails, https://github.com/stschiff/msmc2), selection signature scans (Sweepfinder2, saltiLASSI), TOGA (https://github.com/hillerlab/TOGA), replication timing (https://github.com/ma-compbio/Phylo-HMGP), structural variation calling (https://github.com/schneebergerlab/syri v1.6.3, https://github.com/EichlerLab/pav v2.3.2), segmental duplication (https://github.com/vpc-ccg/sedef v1.1), alpha satellites higher order array prediction (https://github.com/fedorrik/HumAS-HMMER_for_AnVIL), data visualization (https://github.com/daewoooo/SVbyEye, https://github.com/mrvollger/StainedGlass v0.5).

For manuscripts utilizing custom algorithms or software that are central to the research but not yet described in published literature, software must be made available to editors and reviewers. We strongly encourage code deposition in a community repository (e.g. GitHub). See the Nature Portfolio guidelines for submitting code & software for further information.

Data

Policy information about availability of data

All manuscripts must include a data availability statement. This statement should provide the following information, where applicable:

- Accession codes, unique identifiers, or web links for publicly available datasets
- A description of any restrictions on data availability
- For clinical datasets or third party data, please ensure that the statement adheres to our policy

The raw genome sequencing data generated by this study are available under NCBI BioProjects, PRJNA602326, PRJNA976699–PRJNA976702, and PRJNA986878–PRJNA986879 and transcriptome data are deposited under BioProjects, PRJNA902025 (UW Iso-Seq) and PRJNA1016395 (UW and PSU Iso-Seq and short-read RNA-seq). The genome assemblies are available from GenBank under accessions: GCA_028858775.2, GCA_028878055.2, GCA_028885625.2, GCA_028885655.2, GCA_029281585.2 and GCA_029289425.2. Genome assemblies can be downloaded via NCBI (https://www.ncbi.nlm.nih.gov/datasets/genome/?accession=GCF_028858775.2,GCF_029281585.2,GCF_028885625.2,GCF_028878055.2,GCF_028885655.2,GCF_029289425.2). Convenience links to the assemblies and raw data are available on GitHub (https://github.com/marbl/Primates) along with a UCSC Browser hub (https://github.com/marbl/T2T-Browser). The UCSC Browser hub includes genome-wide alignments, CAT annotations, methylation, and various other annotation and analysis tracks used in this study. The T2T-CHM13v2.0 and HG002v1.0 assemblies used here are also available via the same browser hub, and from GenBank via accessions GCA_009914755.4 (T2T-CHM13), GCA_018852605.1 (HG002 paternal), and GCA_018852615.1 (HG002 maternal). The alignments are publicly available to download or browse in HAL118 MAF and UCSC Chains formats (https://cglgenomics.ucsc.edu/february-2024-t2t-apes).

Additional public data also include 22 genome assemblies summarized in Table ASM.S11, as well as the human reference genome, GRCh38. Previous genome annotation files include gorilla (GCF_029281585.2), bonobo (GCF_029289425.2), chimpanzee (GCF_028858775.2), Sumatran orangutan (GCF_028885655.2), and Bornean orangutan (GCF_028885625.2) from NCBI. For the pangenome graph, public human genome assemblies (https://github.com/human-pangenomics/HPP_Year1_Data_Freeze_v1.0) were used. The short-read genome sequencing data for great ape species were obtained from SRP018689, ERP001725, ERP016782, and ERP014340. The public methylation data used in this study include the raw PacBio HiFi reads with kinetics and methylation tags for human (https://humanpangenome.org/data/) and for chimpanzee, gorilla, Bornean orangutan, Sumatran orangutan, and siamang gibbon (https://www.genomeark.org/t2t-all/).

Research involving human participants, their data, or biological material

Policy information about studies with human participants or human data. See also policy information about sex, gender (identity/presentation), and sexual orientation and race, ethnicity and racism.

Reporting on sex and gender	N/A
Reporting on race, ethnicity, or other socially relevant groupings	N/A
Population characteristics	N/A
Recruitment	N/A
Ethics oversight	N/A

Note that full information on the approval of the study protocol must also be provided in the manuscript.

Field-specific reporting

Please select the one below that is the best fit for your research. If you are not sure, read the appropriate sections before making your selection.

☒ Life sciences ☐ Behavioural & social sciences ☐ Ecological, evolutionary & environmental sciences

For a reference copy of the document with all sections, see nature.com/documents/nr-reporting-summary-flat.pdf

Life sciences study design

All studies must disclose on these points even when the disclosure is negative.

Sample size	One cell line per species was used (out of six species: chimpanzee, bonobo, gorilla, B. orangutan, S. orangutan and siamang). To determine polymorphic status, we further used one additional near-T2T assembly per species, summarized in Table ASM.S11.
Data exclusions	No data were excluded.
Replication	For the FISH experiment to validate inversion (hsa16 inversion; Fig. 3b), we repeated for three times and 10 metaphase spreads with the corresponding fluorochromes captured for each experiment.
Randomization	N/A; randomization was not applicable as the samples were obtained from previous research (for additional data generation). Also, with n=1 for each species, makes it impossible to control selection bias.
Blinding	N/A; sample size equal to one technically makes blinding ineffective to control for bias.

Reporting for specific materials, systems and methods

We require information from authors about some types of materials, experimental systems and methods used in many studies. Here, indicate whether each material, system or method listed is relevant to your study. If you are not sure if a list item applies to your research, read the appropriate section before selecting a response.

Materials & experimental systems

n/a	Involved in the study	
☒	☐	Antibodies
☐	☒	Eukaryotic cell lines
☒	☐	Palaeontology and archaeology
☒	☐	Animals and other organisms
☒	☐	Clinical data
☒	☐	Dual use research of concern
☒	☐	Plants

Methods

n/a	Involved in the study	
☒	☐	ChIP-seq
☒	☐	Flow cytometry
☒	☐	MRI-based neuroimaging

Eukaryotic cell lines

Policy information about cell lines and Sex and Gender in Research

Cell line source(s)	KB8711 or PR00251 (San Diego Zoological Society), AG18354 (Coriell), AG06213 (Coriel), AG05252 (San Diego Zoological Society), KB3781 or Jim (San Diego Zoological Society), Jambi (Oregon Health and Science University) (detailed in Table Assembly.S1).
Authentication	Each cell line was authenticated as described in Makova et al. 2024 (Note S1 - for orangutan and S2 - for chimpanzees). In addition to the documentation available (Studbook number), multiple analyses were performed to authenticate our samples. The previous study by Makova et al., observed species-specific genomic signatures, including the nucleolar organizing region of Y chromosome in Sumatran orangutan, and the mitochondrial genome analyses suggesting the Bornean or Sumatran orangutan origins in the respective samples.<br><br>Between chimpanzee and bonobo, the previous study also authenticated the respective species by mitochondrial genome. In addition to this, we also further validated species by comparing with the short-read genome-wide SNP PCA (Fig. Sequencing.S1-S5). We further found average genome-wide sequence identity between two Sumatran orangutan assemblies (one previous and T2T) and one Bornean orangutan assembly (T2T), to identify greater sequence identity among the respective species compared to between two species, as expected (Fig. Sequencing S6).
Mycoplasma contamination	The cell lines were tested negative for mycoplasma contamination.
Commonly misidentified lines (See ICLAC register)	No commonly misidentified cell lines were used.

Plants

Seed stocks	N/A; no plants were used in this study.
Novel plant genotypes	N/A
Authentication	N/A
